# Activation of fluoride anion as nucleophile in water with data-guided surfactant selection†

**DOI:** 10.1039/d3sc06311a

**Published:** 2024-03-19

**Authors:** Krishna Sharma, Alison McCorry, Samuel Boobier, James Mottram, Rachel Napier, Ian W. Ashworth, A. John Blacker, Nikil Kapur, Stuart L. Warriner, Megan H. Wright, Bao N. Nguyen

**Affiliations:** a School of Chemistry, University of Leeds Woodhouse Lane LS2 9JT UK b.nguyen@leeds.ac.uk; b Chemical Development, Pharmaceutical, Technology and Development Operations, AstraZeneca Macclesfield SK10 2NA UK; c School of Mechanical Engineering, University of Leeds Woodhouse Lane LS2 9JT UK

## Abstract

A principal component *surfactant_map* was developed for 91 commonly accessible surfactants for use in surfactant-enabled organic reactions in water, an important approach for sustainable chemical processes. This map was built using 22 experimental and theoretical descriptors relevant to the physicochemical nature of these surfactant-enabled reactions, and advanced principal component analysis algorithms. It is comprised of all classes of surfactants, *i.e.* cationic, anionic, zwitterionic and neutral surfactants, including designer surfactants. The value of this *surfactant_map* was demonstrated in activating simple inorganic fluoride salts as effective nucleophiles in water, with the right surfactant. This led to the rapid development (screening 13–15 surfactants) of two fluorination reactions for β-bromosulfides and sulfonyl chlorides in water. The latter was demonstrated in generating a sulfonyl fluoride with sufficient purity for direct use in labelling of chymotrypsin, under physiological conditions.

## Abstract 2

A principal component *surfactant_map* was developed for 91 commercially surfactants for use in surfactant-enabled organic reactions in water. The map led to activation of fluoride anion, usually strongly hydrated, as an active nucleophile in water.

## Graphical abstract

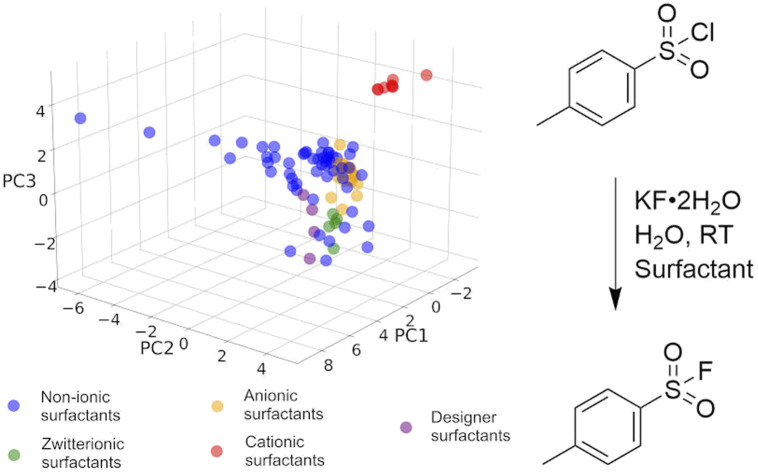

## Introduction

**Fig. 1:**
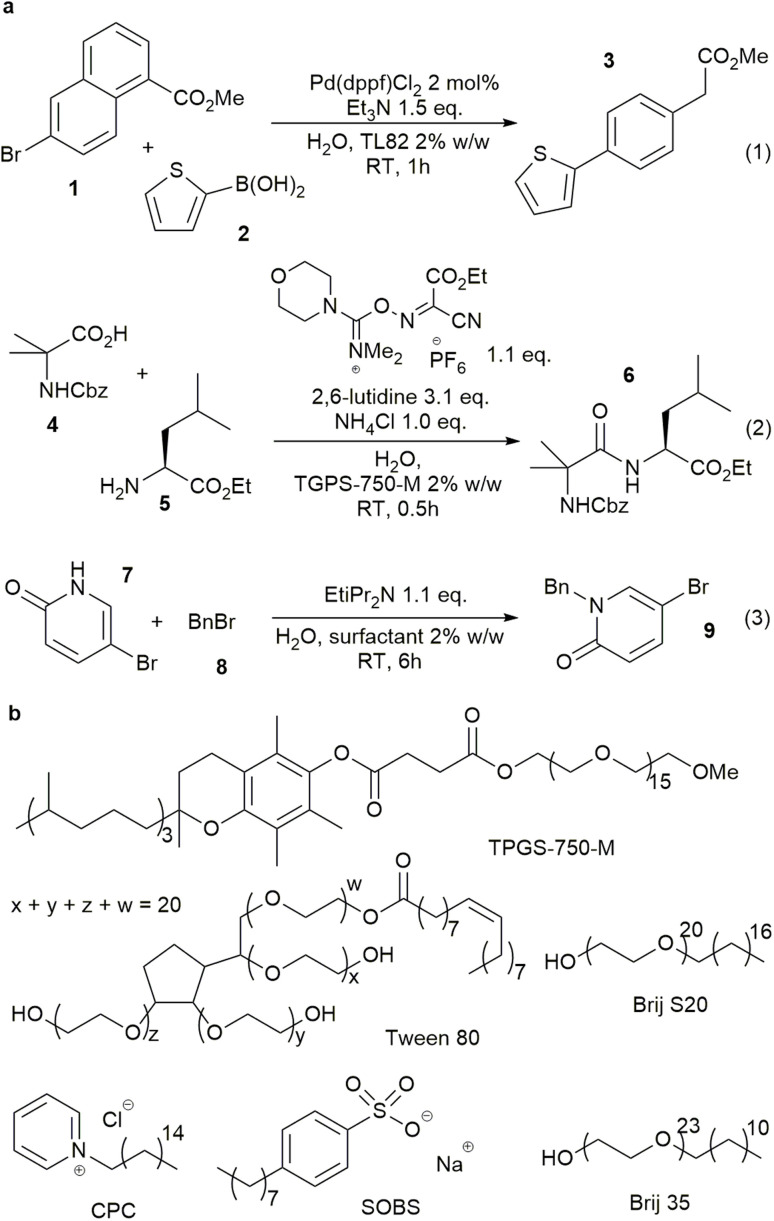
Example of organic reactions in water enabled by surfactants, (a) representative reactions enabled by traditional and designer surfactants, TL82 is a mixture of Tween 80/lecithin 8 : 2, (b) representatives of different classes of surfactants.

Surfactant-enabled organic reactions in water were first reported in the 1970s,^1,2^ wherein the surfactants were thought to stabilise organic emulsions and the charged hydrophilic terminus may have a role in stabilizing the reaction transition states.^3^ ⁠Due to the sustainable nature of water as a reaction medium, there has been a resurgence of interest in these reactions,^3–6^ particularly in the use of neutral designer surfactants.⁠^4^ These surfactants have been effectively implemented in supporting various organic and cross-coupling reactions,^7–9^ including those which employ reactive reagents which may rapidly decompose in water.^10^ ⁠Some of these reactions are shown in Fig. 1a.^11,12^ However, fundamental understanding of how these surfactants work and how to rationally select suitable surfactants from the hundreds of commercially available surfactants remains a key challenge in the field, due to the complex nature of the reaction mixtures.^13–17^

In this paper, we report the first surfactant map (*surfactant_map*) constructed for organic reactions. It was built by Principal Component Analysis (PCA) of surfactant properties, which were selected based on physicochemical understanding of the surfactant-enabled reactions in water. It allows rapid and rational selection of the optimal surfactant for any organic reaction, which in many cases are not designer surfactants. We demonstrated this powerful data-based approach by employing the *surfactant_map* to turn on the nucleophilicity of fluoride in water. Fluoride anion is well-known to be extensively hydrated in water, suppressing its ability to act as a nucleophile in competition with water or hydroxide anion.^18^ On the other hand, fluoride salts are often poorly soluble in organic solvents, necessitating the use of elaborate fluorination agents, such as Me_4_NF·*t*AmylOH,^19^ at high temperature or the highly corrosive KHF_2_ for nucleophilic fluorination.^20,21^ The use of a surfactant allows fine tuning of the fluoride anion hydration at the water–organic interface, successfully revealing the nucleophilicity of fluorides in water and enabling nucleophilic fluorination reactions using simple, relatively safe and readily available KF·2H_2_O as the source of fluoride anion.

## Results and discussion

### Surfactant map for organic reactions

The successes of designer surfactants in enabling organic reactions in aqueous media, with or without a small amount of organic solvent to overcome solubility limits,^3,4^ can potentially lead to many sustainable chemical manufacturing processes. This minimises the use of volatile and toxic organic solvents, particularly the difficult to replace dipolar aprotic solvents. In addition, the surfactants may modify the reactivity of organic compounds and reagents at the organic–water interface,^25^ giving rise to new reactivity which may not be accessible otherwise. The relative high cost and limited supply of designer surfactants, compared to the many readily available commercial, food-grade surfactants, is currently a barrier for their wider adoption. On the other hand, many researchers have established that other surfactants can work just as well, and sometimes better than designer surfactants.^13,26,27^ In this study we aim to develop a data-based tool for rational, rapid surfactant selection, from a pool of 100 common and commercially available surfactants for organic reactions. This tool, which we call *surfactant_map*, is based on the proven PCA approach utilised in solvent selection for organic reactions and ligand selection in catalysis.^28–31^ Due to the length of the study, the latest designer surfactants, *i.e.* Savie,^32^ and APGS-2000-M,^33^ are not included but will be in the next iteration of the map.

**Fig. 2:**
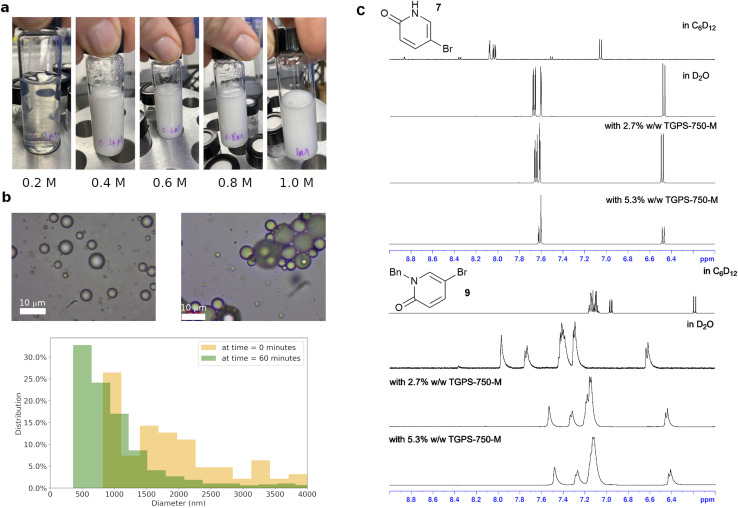
Physicochemical behaviours of surfactant-enabled reactions and the data-based *surfactant_map*, (a) visual observations of TPGS-750-M 2% w/w in water at 0.2, 0.4, 0.6, 0.8 and 1.0 M effective toluene loading, (b) microscope images of toluene in TPGS-750-M 2% w/w and reaction mixture (3) at 0 and 60 minutes, and their particle size distributions, (c) chemical shifts of 7 and 9 in D_2_O, *d*_12_-cyclohexane and D_2_O in the presence of a surfactant.

In order to capture the relevant data on surfactant-enabled reactions, the physicochemical aspects of these reactions were investigated. Whilst this type of reaction is colloquially referred to as ‘micellar catalysis’, the nature of the system can be complex, particularly at higher effective organic concentration (>0.2 M) and in the presence of commonly employed inorganic bases, which increase the ionic strength of the aqueous phase and affect concentrations of species at emulsion surface (Fig. 2a). Dynamic light scattering measurements of surfactant TPGS-750-M in water (0.5% w/w) showed that the system moved away from being micelles at 0.2 M loading of toluene, accompanied by visible change to a milky emulsion. These were confirmed by optical microscopic measurements, which gave a distribution of organic droplet size in the range of 0.2–6 microns O.D. A similar droplet size distribution was observed in the reaction (3) (Fig. 2b), performed with surfactant TPGS-750-M, with increasing aggregation of droplets as the reaction proceeded.

Another uncertainty is the partition of the reactants between the organic (inside the emulsions) and aqueous phases.^34^ While this has an obvious impact on reaction rate, the partition is highly dependent on organic compounds and surfactant. The extent of partition, and where the reaction happens, are critical pieces of information to explain how the surfactant influences the reaction transition state and enables the transformation. Thus, ^1^H NMR experiments were performed to evaluate the partition of the starting materials and product of reaction (3) in the presence of a surfactant in D_2_O by comparing their chemical shifts with the corresponding peaks in D_2_O or *d*_12_-cyclohexane (Fig. 2c, S29–S46†). Reaction (3) was selected due to the two tautomeric forms of starting material 7 with very different chemical shifts in water and organic solvents. Six surfactants, TPGS-750-M, Tween 80, Brij S-20, Brij 35, CPC and SOBS (Fig. 1b), of different classes, *e.g.* neutral, cationic and anionic, were studied at 2.7% w/w loading in D_2_O. Starting material 7 was consistently found to be in an aqueous-like environment (except with SOBS), while starting material 8 was in an organic-like environment. The partition of product 9 was less clear-cut and was surfactant dependent (Section 1.3.3 of ESI†). In TPGS-750-M solutions, the chemical shifts of 9 suggested a dynamic exchange between two environments, *i.e.* organic and aqueous. The chemical shifts of both 7 and 9 change in small degrees when the amount of surfactant was doubled, indicating observable interaction between each compound and the surfactant molecules (Section 1.3 of ESI†). In addition, DOSY experiments showed different changes in diffusion coefficients of 7 (6.3 × 10 to 10 m^2^ s^−1^ to 4.5 × 10 to 10 m^2^ s^−1^) and 8 (4.0 × 10 to 10 m^2^ s^−1^ to 4.1 × 10 to 11 m^2^ s^−1^) in D_2_O upon inclusion of 2.7% w/w of TPGS-750-M. These suggested inclusion of 8 inside the TPGS-750-M micelles/emulsions, and weak interaction of 7 with the interface of these micelles/emulsions, in agreement with the changes in ^1^H NMR chemical shifts above. Similar DOSY interaction between a catalyst and anionic surfactants was also observed by Scarso and Strukul in a surfactant-enabled Baeyer–Villiger reaction.^35^ Taken all together, the NMR evidence suggested that the reaction likely happened at the organic–water interface, assisted by the surfactant.

**Fig. 3:**
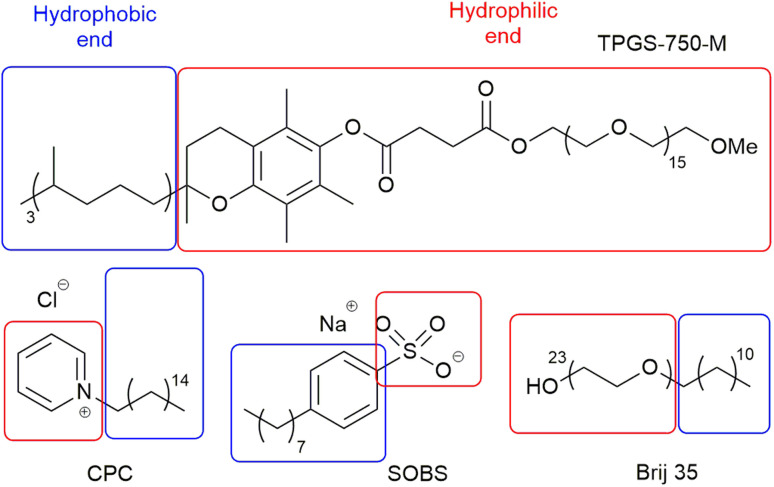
Example of hydrophobic and hydrophilic fragments for surfactants.

Based on the observations above, a number of descriptors, which represent the computational properties of the surfactant molecule (2D and 3D structural information, *e.g.* SASA and number of double bonds, divided into hydrophobic and hydrophilic fragments (Fig. 3) to reflect emulsion properties by hydrophilic–lipophilic balance),^36,37^ its interaction with water (number of OH groups, *e.g.* Brij S20 (Fig. 1b), Δ*G*_solv_, HOMO and LUMO energies, *etc.*), its micellar properties (critical micelle concentration, aggregation number and micelle size), and its emulsion properties (zeta potential, contact angle, and hydrophilic lipophilic balance), were selected to build the *surfactant_map* for 100 common surfactants (Table S13,† selected based solely on commercial availability in order to expand the pool of surfactants for synthetic reactions). While the reaction mixtures are often emulsions, these micellar properties do represent surfactant–surfactant interactions, which are relevant to emulsions. The micellar properties were curated from the literature, while the emulsion properties were measured experimentally. The computational descriptors were derived using either the *rdkit* cheminformatics package or PM6 molecular modelling calculation.^22^ One of the most difficult aspects of numerically representing surfactants in emulsions/micelles is how to represent the charge of the surfactant species, which is a discrete rather than continuous variable, without artificially clustering cationic, anionic and neutral surfactants in the *surfactant_map*. Thus, the intrinsic Hirshfeld charge of the atom with the most negative charge of the hydrophilic end of the surfactant molecule was used for each surfactant instead.^38^ While the majority of the hydrophobic chains are linear alkyl chains, a small number of them include cis double bonds, which can have significant impact on the packing of the surfactant molecules in micelles and emulsions, and consequently the dynamic rate of material exchange at the organic/water interface. Therefore, a descriptor for the number of cis double bonds in the hydrophobic part of each surfactant was included in the dataset. The full list of descriptors is summarised in Table 1.

**Table 1:** Descriptors for *surfactant_map* and their sources^a^

Descriptor	Representing
**Critical micelle concentration** b	Surfactant–surfactant interactions
**Aggregation number range** b	Surfactant–surfactant interactions
**Micelle size range** b	Surfactant–surfactant interactions, homogeneity
*Contact angles (left and right)* c	Surface tension and wettability
*Zeta potential* c	Charge environment around micelles, emulsions
*Hydrophilic Lipophilic Balance (HLB)*	Emulsion stability
Hydrophilic fragment rotatable bonds^d^	Flexibility of surfactant molecules and emulsion flexibility/stability
Hydrophobic fragment rotatable bonds^d^	Flexibility of surfactant molecules and emulsion flexibility/stability
Hydrophilic fragment longest chain length^d^	Size of the interface layer between organic and aqueous phases
Hydrophobic fragment longest chain length^d^	Capability for stabilizing organic phase inside emulsions
Hydrophilic fragment volume^d^	Packing of surfactant and stability of emulsion
Hydrophobic fragment volume^d^	Packing of surfactant and stability of emulsion
Hydrophilic fragment surface area^d^	Packing of surfactant and stability of emulsion
Hydrophobic fragment surface area^d^	Packing of surfactant and stability of emulsion
Hydrophobic fragment number of C=C bonds	Flexibility of surfactant molecules and emulsion flexibility/stability
Hydrophilic fragment number of OH groups	Capability of H-bonding at the emulsion interface
Hydrophilic fragment Δ*G*_solv_^d^	Stability of emulsion
Hydrophilic fragment dipole moment^d^	Stability of emulsion
Hydrophilic fragment HOMO energy^d^	H-bonding capability and interactions with transition states
Hydrophilic fragment LUMO energy^d^	H-bonding capability and interactions with transition states
Hydrophobic fragment dipole moment^d^	Stability of emulsion
Hirshfeld charge for most negative heteroatom^d^	Interactions with transition states

a Micellar properties are in bold; emulsion properties are in italic; and molecular properties are in normal font.

b Experimental (literature).

c Experimental (measured).

d *rdkit*, PM6.^23,24^

**Fig. 4:**
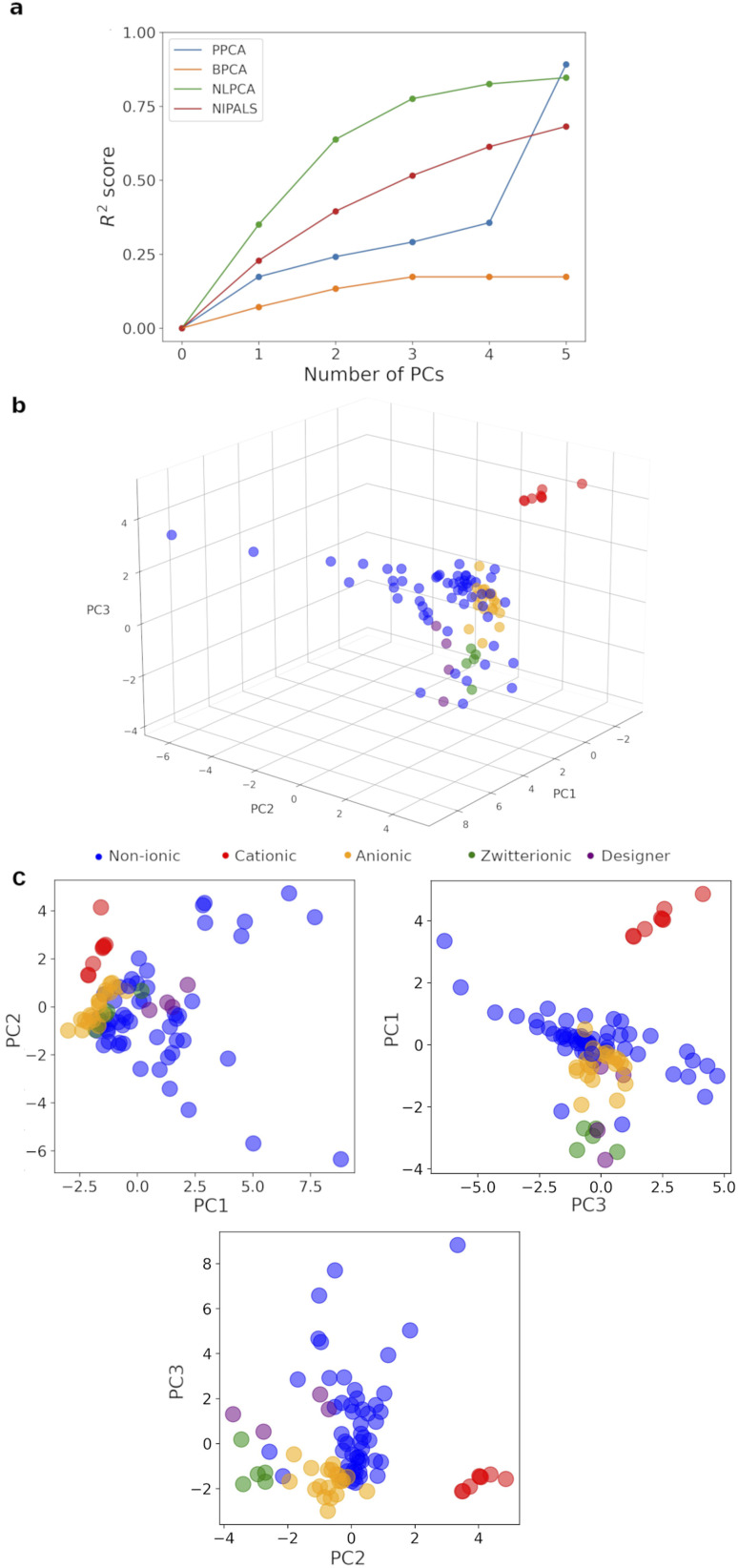
(a) Variance captured with different PCA algorithms *vs.* number of PCs. (b) NIPALS-derived 3D *surfactant_map*. (c) 2D projections of the *surfactant_map* showing the information in each PC.

Whilst experimental data are highly valuable, the inclusion of experimental descriptors led to a significant amount of missing data, *i.e.* 263 entries, accounting for 10.5% of the total 2500 (100 surfactants × 25 descriptors). This prevents the use of a standard PCA algorithm. Removing experimental data completely risks losing reliable information on intermolecular interaction between surfactant molecules and with solvent. Consequently, four modified-PCA algorithms: PPCA (estimate/impute missing values with Gaussian probability),^39^ BPCA (estimate/impute missing values with Bayesian probability and expectation–maximization repetitive algorithm),^40^ NLPCA (artificial neural networks which convert a dataset to principal components and reconstruct it to impute missing data),^41^ and NIPALS (iterative method which skips the missing data, see Section 2.5.1 of ESI† for more complete explanation of the algorithms),^42^ which have been demonstrated to work with varying degrees of missing data, were compared based on their capture of data variance with up to 5 principal components (PCs, Fig. 4a). BPCA did not cope well with the missing data. PPCA gave the best result with *R*^2^ = 0.89 for 5 PCs, but very poor capture of variation in the first 4 PCs. For visualization purposes, NLPCA was the best method, achieving *R*^2^ = 0.78 with only 3 PCs, and NIPALS was the next best choice. However, the PCs generated by NLPCA were found to vary significantly between different runs (the difference in PC1 (0.01 ± 0.13 over 100 surfactants) was −0.176 to 2.317 between 2 runs). This was due to due to the reconstruction stage of the algorithm and the large amount of missing experimental data for 9 surfactants, which consistently lack >5 experimental descriptors, leading to randomization of the produced PCs. Varying the number of cycles of optimization/imputation between 100 and 10 000 did not improve the reproducibility, and averaging 100 different runs did not produce sensible results, *i.e.* most surfactants were pushed close together in 3*D* space. This is a known issue with NLPCA algorithm, which works best with randomly missing data instead of large amount of missing data on certain rows.^43^ On the other hand, NIPALS algorithm produced identical PCs for each surfactants in repeated runs on all 100 surfactants. Careful examination of the 9 surfactants with significant missing experimental data showed that they were placed close to surfactants with which they share little similarity within the 3D map with PC1–3. Consequently, these 9 surfactants were removed, leaving 91 surfactants for the final analysis, and reducing the percentage of missing data from 10.6% to 8.8% (Table S14†). A 3D map based on 3 PCs generated with NIPALS for these 91 surfactants was built and shown in Fig. 4b as our *surfactant_map*.

**Fig. 5:**
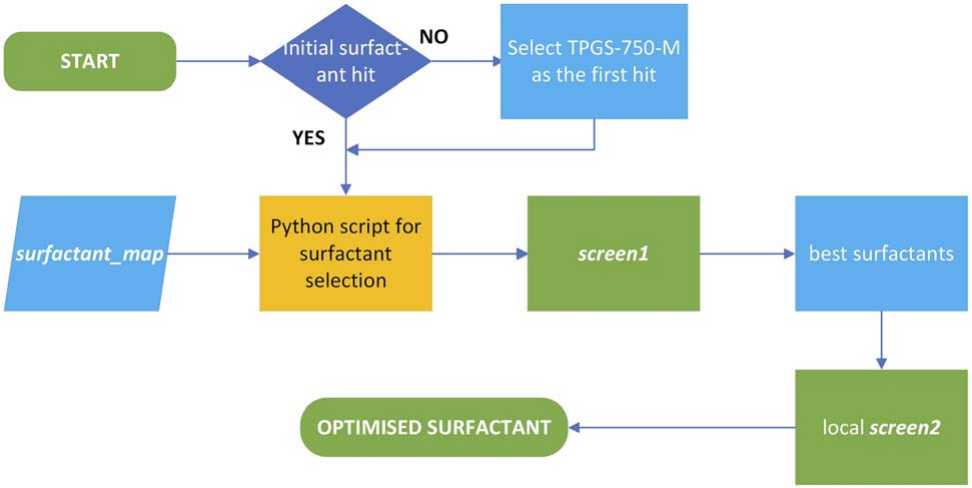
Workflow employing the *surfactant_map* for optimising surfactant-enabled reactions.

This *surfactant_map* showed some degree of the expected clustering of cationic, anionic and zwitterionic surfactants. Crucially, the most numerous neutral surfactants (blue) occupy the most space in the PCA map, mixing well with the anionic (orange) and zwitterionic surfactants (green) but not the cationic surfactants (red). Zwitterionic surfactants and cationic surfactants are positioned close to others in the same class, suggesting that they behave similarly to each other. Analysis of the map suggests that PC3 is significantly influenced by nominal charge of the surfactant molecule; cationic surfactants have higher PC3 values and zwitterionic surfactants have lower PC3 values; and neutral surfactants cover a very wide range of PC1 and PC2 values. These are consistent with the loadings for PC1–3 (Table S15†), wherein zeta potential, solvation energy and the most negative atomic charge on the hydrophilic fragment contribute the most to PC3. The most significant contributors to PC1 are centred on the volume, surface area and flexibility of the hydrophobic and hydrophilic fragments of each surfactant, *i.e.* hydrophobic/hydrophilic balance. PC2 was mainly derived from a combination of contact angles, zeta potential and volume/area/flexibility properties of the hydrophobic fragment. Importantly, the designer surfactants (purple) occupy a relatively small, but central portion of surfactant space compared to the rest of the surfactants (Fig. 4c). This explains the relative success of these surfactants in surfactant-enabled reactions and, at the same time, highlights the risk of exclusively focusing on designer surfactants when screening surfactants for a given reaction. A small number of outliers, *e.g.* Croduret 25-LQ, and Croduret 50-SS, can be attributed to their unique structures and properties, *i.e.* PEG-25 or PEG-40 hydrogenated castor oil. Interestingly, the surfactant IGEPAL CO-720, which is structurally very similar to IGEPAL CA-720 and Triton-X-102, was placed closest to these surfactants in the map, showcasing the ability of NIPALS algorithm to cope with missing data for IGEPAL CA-720 and Triton-X-102, if there is significant similarity in the remaining descriptors between surfactants. The *surfactant_map* and workflow led to rapid identification of sulfobetain-16 and TTAB as the best surfactants for reaction (3), giving 95% yield (5% of *O*-alkylated product) in 20 minutes at 45 °C, in comparison to 64% yield with TPGS-750-M (Fig. 5). A similar surfactant screen for the reaction between 7 and allyl bromide, instead of benzyl bromide, also identified sulfobetain-16 and TTAB as the optimal surfactant (100% for *N*-allylated product with sulfobetain-16). Nevertheless, the true usefulness of this *surfactant_map* needs to be demonstrated in enabling new chemical reactions.

### Nucleophilic fluorination with fluoride anion

**Fig. 6:**
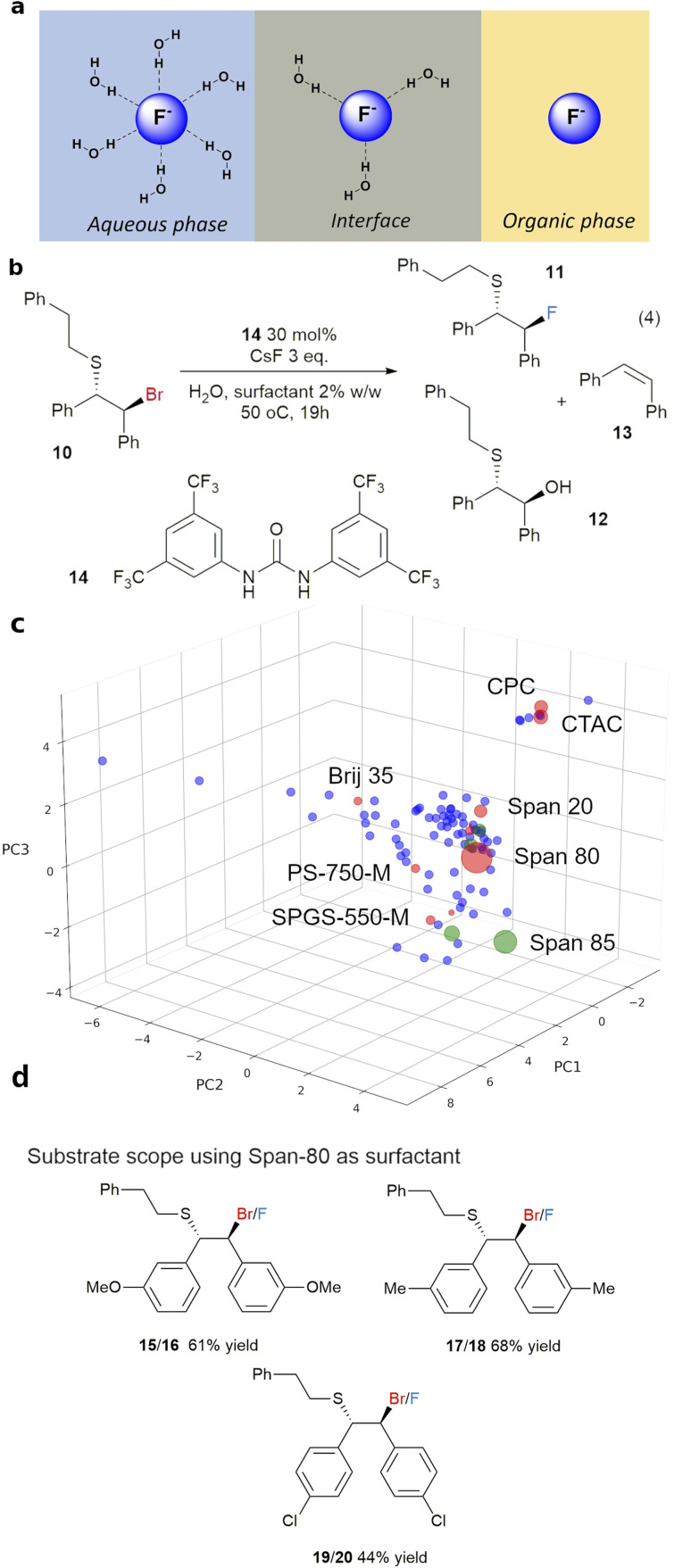
(a) Modification of the nucleophilicity of fluoride anion at the organic/water interface, (b) fluorination of β-bromosulfides in water with CsF and surfactant in *screen1*, (c) results from *screen1* (red) and *screen2* (green) for reaction (4) showing the local area with best selectivity, after 19 hours at 50 °C, the tested surfactants are in red, and the size of the marker represent the 11 : 12 ratio, (d) substrate scope and ^1^H NMR yields.

While the use of fluoride as a nucleophile in mixture of organic solvents and water have been reported in recent years,^44,45^ often at elevated temperature, reactions in water alone are rare due to the very strong hydration of fluoride anion in water. The only example without a co-solvent in the literature employs KHF_2_, which is corrosive against glass vials and reactors, as the source of fluoride.^20^ While the nucleophilicity of fluoride anion in organic solvent is much improved, the high lattice energy of readily available inorganic fluorides means that their solubility is often very low in these solvents. Previous solutions for this conundrum included the use of phase transfer catalyst and water/organic solvent combinations,^46–48^ and elaborate fluoride reagent such as NMe_4_F and NMe_4_F·*t*Amyl-OH,^19^ which have better solubility in organic solvents, or SuFEx reagents,^49^ which generate *in situ* fluoride anion in organic solvents. An alternative, yet unexplored, solution is to modify the environment around the fluoride anion, and thus its reactivity, at the organic/water interface through the use of surfactant (Fig. 6a). This has the benefit of circumventing the solubility limitation, while fine tuning the nucleophilicity and basicity of fluoride anion to achieve selective reactions. Thus, the *surfactant_map* was used to select the surfactants which can activate fluoride anion as a nucleophile in water for synthesis. To achieve this, a selection of 8–10 surfactants (*screen1*, sufficient to cover most areas of the map) were made using a Python script which randomises the selection of surfactants while maximizing their coverage of the *surfactant_map*. The origin point can be set for the first hit surfactant which enables reaction, if known, or TPGS-750-M, which locates approximately in the center of the surfactant space. The best results obtained with *screen1* will lead to the selection of an additional 5–6 surfactants in the region around the best surfactants for *screen2*, leading to the optimised surfactant for a given reaction. This workflow is described in Fig. 5.

This workflow was first demonstrated with the fluorination of episulfoniums, generated *in situ* from β-bromosulfides (4), reported by Gouverneur and co-workers,^47^ and the results are summarised in Fig. 6. A non-chiral catalyst, 1,3-bis[3,5-bis(trifluoromethyl)phen-yl]urea 14, was used for simplicity, and the competition between the fluoride anion and water as nucleophiles, giving products 11 and 12, was used to evaluate the effectiveness of the surfactant in improving the reactivity of the fluoride anion (Fig. 6b). Initial reaction using 3 equivalents of CsF in a water/toluene (9 : 1) mixture without a surfactant at room temperature gave little conversion and an unexpected product, alkene 13 (entry 1, Table 2). Formation of alkene *via* nucleophilic attack of a fluoride anion on the S atom of an episulfonium, instead of the C atoms, has previously been reported by Helmkamp.^50^ Changing the organic solvent to the more polar 1,2-dichloroethane (DCE) led to improved reactivity, although not necessarily a significant change in products ratio (entry 2). The best results, 57%, of 11 and the best combined yield for products of fluoride nucleophilic attack on the C and S atoms, *i.e.* 92% for 11 and 13, was obtained with Span 80 as the surfactant in *screen1*, starting from Span 20 as the first hit surfactant and performed with gentle heating (50 °C). This was followed by *screen2* using Span 85, Span 60, Span 40 and Tween 85, which are near Span 80 in the *surfactant_map*. Nevertheless, Span 80 remained the best performing surfactant. The surfactants had a clear impact on the distribution of products between hydrolysis (12), nucleophilic attack on C (11) and on S (13) (Table S18 of the ESI†). More hydrolysis product 12 was observed with surfactants with shorter hydrophobic chain lengths (C10–12: SDS, lauryl betaine, PS-750-M and Brij 35). Selectivity between 11 and 13 is relatively poor, with high yields for 13 often associated with high yields of the desired product 11. The lowest 11 : 13 ratios were observed lauryl betaine and Brij 52, straight chain surfactants with short hydrophilic fragment. Further optimization enabled replacement of CsF with the much cheaper KF·2H_2_O (6 eq.) without significant change to reaction performance. Attempts at replacing DCE with toluene led to a lower selectivity, likely *via* changes to the stability of the emulsions, giving 40% of the desired product 11 (entry 9). The standard reaction conditions, using Span 80 and 6.0 eq. of KF·2H_2_O were successfully applied to three derivatives of 10 as substrates, giving moderate to good yields of the desired fluorides (Fig. 6d).

**Table 2:** Surfactant optimization for fluorination of β-bromosulfide 10^a^

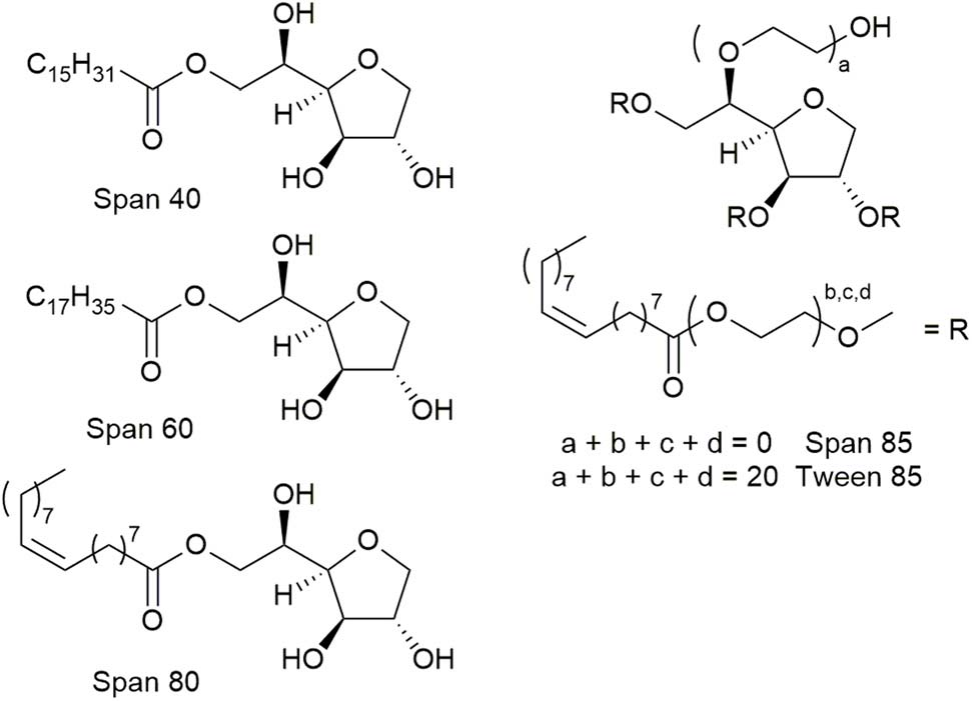					
No.	Solvent	Fluoride	Surfactant	10 : 11 : 12 : 13^b^	11 + 13 (%)
1^c^^,^^d^	Toluene : H_2_O (10 : 90)	CsF	None	81 : **6** : 7 : 6	12
2^c^^,^^d^	DCE : H_2_O (10 : 90)	CsF	None	69 : **10** : 9 : 12	22
3	DCE : H_2_O (10 : 90)	CsF	Span 80	0 : **57** : 8 : 35	92
4	DCE : H_2_O (10 : 90)	CsF	Span 85	0 : **52** : 13 : 35	87
5	DCE : H_2_O (10 : 90)	CsF	Span 40	0 : **38** : 32 : 30	68
6	DCE : H_2_O (10 : 90)	CsF	Span 60	0 : **39** : 31 : 30	69
7	DCE : H_2_O (10 : 90)	CsF	Tween 85	0 : **44** : 25 : 31	75
8^e^	DCE : H_2_O (10 : 90)	KF·2H_2_O	Span 80	0 : **58** : 13 : 29	87
9	Toluene : H_2_O (10 : 90)	KF·2H_2_O	Span 80	0 : **40** : 28 : 32	72
10^d^	DCE : H_2_O (10 : 90)	TBAF	None	75 : **0** : 6 : 18	18
11^e^	DCE : H_2_O (10 : 90)	KF·2H_2_O	Dibenzo-18-crown-6	18 : **16** : 50 : 16	32

a Standard conditions [10] = 0.15 M, 3.0 eq. of fluoride, 30 mol% of catalyst 14, 2% w/w surfactant in water, 19 h at 50 °C.

b Determined by ^1^H NMR.

c [10] = 0.08 M.

d At room temperature.

e 6.0 eq. of KF·2H_2_O. The full list of surfactants and experiments is in Table S17.

To rule out a simple solubility effect, the reaction was performed without surfactant, using either tetrabutylammonium fluoride (TBAF) at room temperature or a combination of KF·2H_2_O/dibenzo-18-crown-6 ether (entry 10 and 11). Both conditions increase the concentration of fluoride anion in the organic phase. No product 11 was observed with TBAF, while a 1 : 3.13 ratio of 11 : 12 was observed with KF·2H_2_O/dibenzo-18-crown-6 ether. These data support our hypothesis of reactivity attenuation at the water/organic interface by surfactant.

**Fig. 7:**
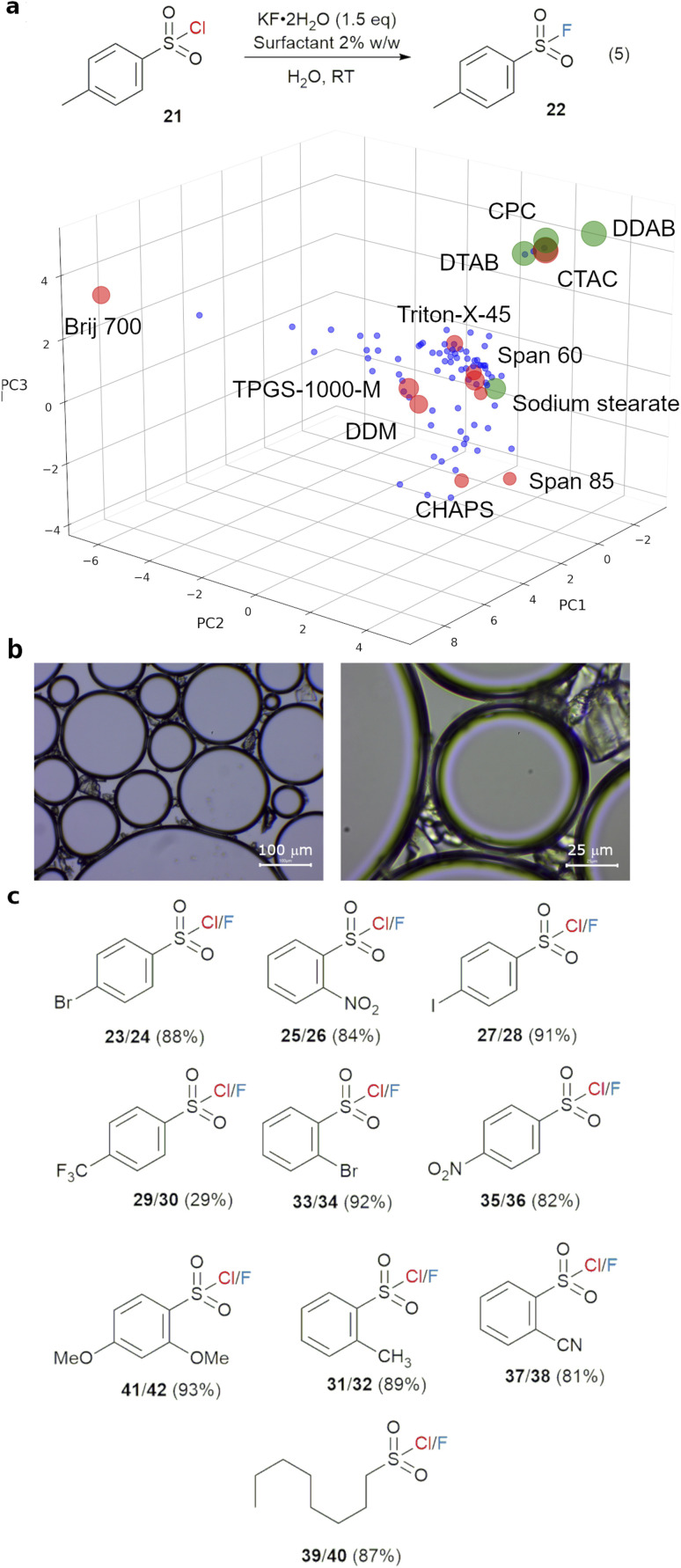
(a) Surfactant screen results for reaction (5) showing the local area with best reaction yield after 3 hours at room temperature, the tested surfactants from *screen1* are in red and from *screen2* are in green, and the size of the marker represents the yield of 22, (b) microscope images of reaction mixtures without KF·2H_2_O; (c) substrate scope and yield of fluorinations of sulfonyl chlorides in water with KF (3.0 eq.) and CTAC as surfactant.

Sulfonyl fluorides have been widely employed as reactive probes in chemical biology, thanks to their biocompatibility and specific reactivity toward reactive serine, threonine, lysine, tyrosine cysteine and histidine residues.^51,52^ Their synthesis often involves activation of sulfonamides, deoxygenation of sulfonic acids or electrochemical oxidation of thiols under harsh or highly reactive conditions.^51^ Direct fluorination of sulfonyl chlorides under aqueous conditions is highly desirable, as the sulfonyl fluorides may be readily used in peptide labelling without the need for purification with flash chromatography. This has been shown possible with the help of a phase transfer catalyst or in a combination of MeCN and water, albeit using the highly corrosive KHF_2_ reagent.^20,21,49^ Given our success with fluorination of β-bromosulfides, we hypothesised that the use of a surfactant will enable the use of a simple fluoride salt. Thus, a surfactant screen (*screen1*, using TPGS-750-M as the starting point) using 10 surfactants was carried out for reaction (5) from *p*-toluenesulfonyl chloride with 1.5 eq. of KF·2H_2_O at room temperature in water (Fig. 7a). The best result was obtained with CTAC, giving 96% yield after 3 hours, compared to 13% yield without any surfactant (Table 3, entries 1 and 11). Thus, the surfactant space around CTAC was explored in *screen2* with CPC, CTAB, DDAB and DTAB (entries 3–7).

**Table 3:** Surfactant *screen2* for fluorination of 21 to 22^a^

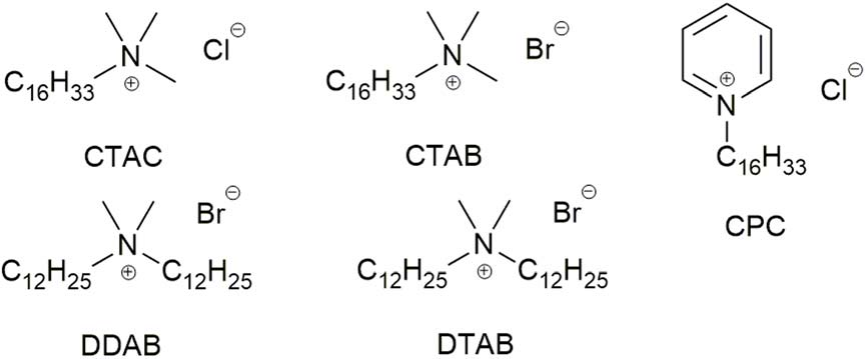		
No.	Surfactant	^1^H NMR yield of 22 (%)
1	None	13
2	Span 60	57
3	Span 80	25
4	Span 85	25
5	Triton-X-45	41
6	Brij-700	46
7	TPGS-1000-M	59
8	1-Dodecanesulfonic acid sodium salt	35
9	PS-750-M	49
10	CHAPS	29
11	CTAC	96
12	CPC	94
13	CTAB	90
14	DDAB	96
15	DTAB	88
16	Sodium stearate	65
17	18-Crown-6	77

a 0.15 M substrate, 1.5 eq. of KF·2H_2_O, 2% w/w surfactant at room temperature, 3 h reaction time.

The best performing class of surfactant are cationic surfactants, which consistently gave high yields (88–96%) of 22. CTAC and DDAB are the best performing surfactants, both giving 96% yield of 22. As these cationic surfactants can also act as phase transfer catalysts, the reaction mixtures of 21 and CTAC (without KF·2H_2_O) were examined with a microscope. Clear emulsions (50–500 μm), which resisted pressure, were observed. Surprisingly, compound 21 persisted as observable crystals in this system, instead of being completely dissolved as an organic phase inside the emulsions (Fig. 7b). The lack of an organic phase, and the large size of the emulsions suggested that these are bilayer emulsions, representing a completely different reaction system compared to the standard view of ‘micellar catalysis’ (Section 5.3 of ESI†). Strong interfacial interaction between these crystals and the emulsions led to clustering, and a fluorination reaction which happens at the interface of the emulsions, crystalline 21 and the aqueous phase. To separate the roles of the surfactant in stabilising emulsions and facilitating phase transfer, 18-crown-6, a known phase transfer catalyst for potassium cation, was used instead. This led to a good conversion of 77%, highlighting the dual nature of the surfactant in this reaction.^53^ As CTAC is significantly cheaper and more readily available than DDAB, it was selected as the surfactant of choice for substrate scope study (Fig. 7c). Excellent isolated yields (81–93%), *via* a simple filtration with most aryl sulfonyl chlorides (23–37), were obtained with a wide range of aryl and alkyl sulfonyl chlorides. ^1^H and ^19^F NMR spectra of the crude reaction mixture indicated complete conversion in most cases, with partial solubility of the sulfonyl fluorides in water being the reason behind imperfect isolation yields in some cases. The reaction tolerates a wide range of substituents on the phenyl group at *o*- and *p*-positions. The only poor yield was obtained with a strongly electron-withdrawing *p*-CF_3_ substituent, with complete consumption of the sulfonyl chloride. This can be attributed to the low stability of the electron-poor sulfonyl fluorides against hydrolysis.^54^

**Fig. 8:**
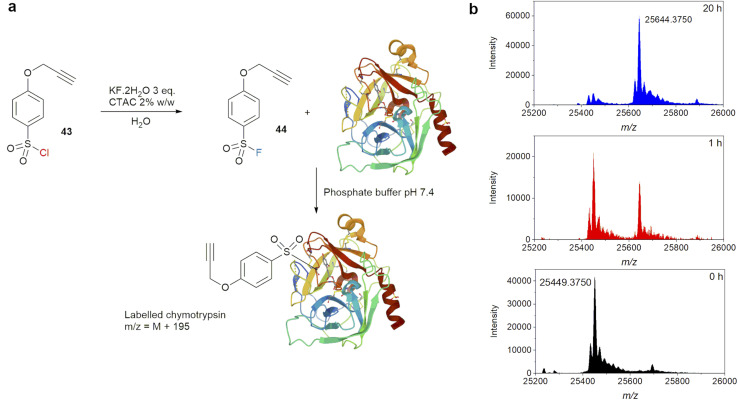
(a) Labelling experiments of chymotrypsin with sulfonyl fluoride generated by nucleophilic fluorination of sulfonyl chloride in water, (b) MS evolution of labelling experiment.

The effectiveness of this new synthetic protocol for sulfonyl fluorides was demonstrated in a labelling experiment with the serine hydrolase chymotrypsin (Fig. 8). Chymotrypsin is known to react with sulfonyl fluoride-containing protease inhibitor through the reactive active site serine.^55,56^ 4-(Prop-2-yn-1-yloxy)-benzene-1-sulfonyl chloride (43) was chosen as the starting material for conversion to sulfonyl fluoride 44 at room temperature. Substrate 43 is an active sulfonyl chloride, which can readily react with methyl glycolate in the presence of a base at 0 °C, and is thus susceptible to hydrolysis.^57^ It also contains an alkyne functional group which allow for further functionalization *via* ‘click’ reaction. Thus, it presents a good test for fluorination *vs.* hydrolysis with a complex substrate. Reaction between 43 and KF·2H_2_O was carried out using the optimised conditions above, and the product was purified by extraction with DCM. Once isolated, one equivalent of 44 in DMSO (calculated based on 100% conversion of 43) was added to a solution of bovine chymotrypsin in phosphate buffer pH 7.4 at a final concentration of 10 μM. LC-MS of the reaction mixture was compared to a control sample without 44 at 1 and 20 hours (Fig. 8b). MS data showed a significant new set of peaks corresponding to a single labelling event, *i.e.* [M + 195]^+^ for [chymotrypsin + C_9_H_7_O_3_S]^+^, even after just 1 hour. At 20 hours, the labelling was judged complete.

## Conclusions

In conclusion, we demonstrated that surfactant-enabled organic reactions in water are complex and diverse physiochemical systems, in which the desired reaction often occurs at the interface and can be influenced by the choice of surfactants. Given this complexity, the *surfactant_map* we developed, based on physicochemical understanding of these emulsified systems, can enable rapid screening of surfactants which enable organic reactions in water. The effectiveness of the map was demonstrated in fine-tuning the reactivity of simple fluoride salts at the organic–water interface, enabling fluoride anion as a nucleophile in reactions with β-bromosulfides and sulfonyl chlorides while suppressing hydrolysis of the substrate as side reactions. These outcomes were demonstrated in the simple synthesis of a complex sulfonyl fluoride and subsequent successful labelling of chymotrypsin. Work to expand surfactant-enabled nucleophilic fluorinations with simple fluorides is on-going in our group and will be disseminated in due course. Importantly, this *surfactant_map* underpins a rational and data-based approach to surfactant selection in surfactant-enabled organic reactions in water, a rapidly growing area of green chemistry in both academia and industry.

## Data availability

The data for the surfactant map and R code are available at: https://zenodo.org/record/7979537.

## Author contributions

BNN, AJB, NK and IA provided direction and supervision in the project. MHW and SLW planned and supervised the labelling experiments of chymotrypsin. AMC and KS planned and carried out the experiments. SB carried out PM6 calculations. JM performed microscope measurements. AMC and KS contributed equally to this work.

## Conflicts of interest

The authors declare the following conflict of interest: IA is an employee of AstraZeneca and may hold share options and shares in AstraZeneca.

## Supplementary Material

SC-015-D3SC06311A-s001
