# Supplementary material for: Activation of fluoride anion as nucleophile in water with data-guided surfactant selection
Source: Chem Sci. 2024 Mar 19;15(15):5764–74. doi: 10.1039/d3sc06311a (PMC11023051; doi:10.1039/d3sc06311a)
Supplement: SC-015-D3SC06311A-s001 [file SC-015-D3SC06311A-s001.pdf]

Electronic Supplementary Material (ESI) for Chemical Science.
This journal is © The Royal Society of Chemistry 2024

Activation of Fluoride Anion as Nucleophile in Water with Data-Guided Surfactant Selection

Krishna Sharma, Alison McCorry, Samuel Boobier, James Mottram, Rachel Napier, Ian W. Ashworth, A. John Blacker, Nikil Kapur, Stuart L. Warriner, Megan H. Wright, Bao N. Nguyen*

Supplementary Information

Table of contents

Table of contents 2

1 Chemophysical studies of surfactant-enabled reactions 6

1.1 Studies of surfactants in water with organic loading 6

1.1.1 Visual observations 6

1.1.2 Dynamic light scattering measurements 9

1.1.3 Cryo-SEM 14

1.1.4 Microscope imaging 15

1.1.5 Size comparison between different techniques 17

1.2 Studies of reaction (3) 20

1.2.1 Microscope imaging of reaction (3) with TPGS-750-M surfactant 20

1.3 NMR studies of reaction (3) 22

1.3.1 ^{1}H NMR spectra of 5-bromo-2(1H)-pyridone 23

1.3.2 ^{1}H NMR spectra of benzyl bromide 25

1.3.3 ^{1}H NMR spectra of the product 29

1.3.4 DOSY experiments 31

2 Surfactant map development 40

2.1 Literature descriptors 41

2.1.1 Critical micelle concentration (CMC) 41

2.1.2 Aggregation number 41

2.1.3 Micelle Sizes 41

2.2 Experimental descriptors 42

2.2.1 Contact angle measurements 42

2.2.2 Zeta (ζ) potential measurements 42

2.3 Computational descriptors ... 43
2.3.1 Volume of hydrophilic and hydrophobic regions of surfactant ... 43
2.3.2 Area of hydrophilic and hydrophobic regions of surfactant ... 44
2.3.3 Rotatable bonds ... 45
2.3.4 Longest chain length ... 45
2.3.5 Number of C=C double bonds (hydrophobic fragment) ... 45
2.3.6 Free OH groups (hydrophilic) ... 45
2.3.7 Electronic structure calculation details ... 46
2.4 List of surfactants ... 47
2.5 Principal component analysis ... 57
2.5.1 Algorithms ... 57
2.5.2 PCA results ... 59
3 Guide to using the *surfactant_map* ... 61
4 Surfactant screening for reaction (3) ... 62
4.1 Standard reaction protocol ... 62
4.2 Surfactant screening results ... 62
4.3 Reaction with allyl bromide ... 63
4.4 Surfactant screening results ... 64
5 Nucleophilic fluorination with fluoride anion ... 65
5.1 Fluorination of episulfoniums ... 65
5.1.1 Standard reaction protocol ... 65
5.1.2 Determination of product ratio ... 66
5.1.3 Synthesis of starting materials and catalysts ... 66
5.1.4 Results of screening with *surfactant_map* ... 80

5.2 Fluorination of sulfonyl chlorides 81
5.2.1 Standard reaction protocol 81
5.2.2 Determination of reaction yields 83
5.2.3 Characterisation data of products 83
5.2.4 Results of screening with *surfactant_map* 90
5.3 Microscope images of reaction mixtures 92
5.3.1 Images of a mixture of 21 and CTAC in water 92
5.3.2 Images of a mixture of 21 and CTAC in water after application of pressure 93
5.4 Labelling of chymotrypsin with sulfonyl fluoride 44 93
5.4.1 Preparation of 4-(Prop-2-ynyloxy)benzenesulfonyl chloride 43 93
5.4.2 Preparation of 4-(Prop-2-ynyloxy)benzenesulfonyl fluoride 44 94
5.4.3 Labelling experiment 94
6 Python and R code 96
6.1 Calculating the volumes of hydrophobic and hydrophilic regions (Python) 96
6.2 Calculating surface areas hydrophilic and hydrophobic surfactant region (PyMOL) 97
6.3 Counting rotatable bonds (Python) 97
6.4 Calculating longest chain length (Python) 97
6.5 Principal component analysis 101
6.5.1 Normalise data (Python) 101
6.5.2 Perform principal component analysis (R) 101
6.5.3 Perform 100 different runs in principal component analysis (R) 104
6.5.4 Averaging the PCs of 100 different runs of PCA (Python) 104
6.6 NIPALS map plotting (Python) 105
6.7 Python code for surfactant selection 106

7 Spectral data 110

8 References 152

1 Chemophysical studies of surfactant-enabled reactions

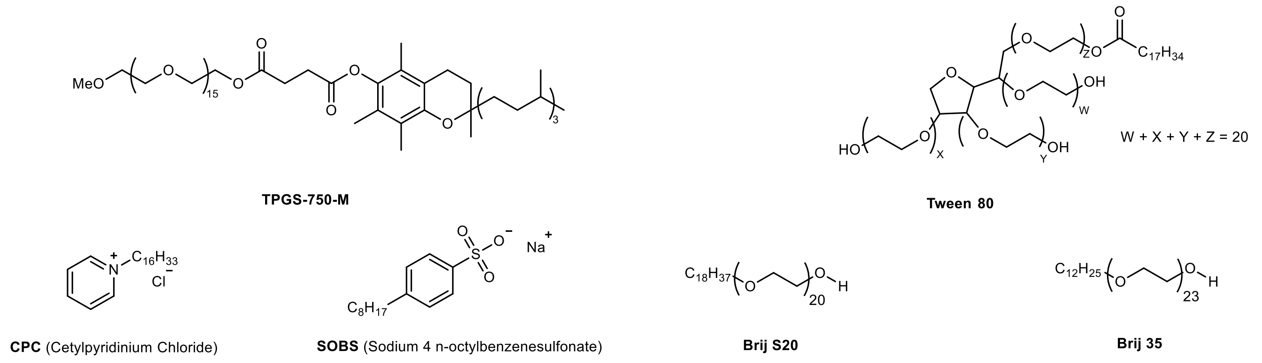

Figure S1. Structures of surfactant used in system study

1.1 Studies of surfactants in water with organic loading

1.1.1 Visual observations

1.1.1.1 Protocols

<u>0.001 M toluene/water</u>

To a 500 mL rbf was added toluene (21 µL) and deionised water (200 mL). This was stirred for 1 hour before a 10 mL aliquot was removed and transferred to a 15 mL vial which contained the desired surfactant (50 mg, 0.5 % w/w). This was left stirring overnight (16 hrs) at room temperature and 1000 RPM, using a slide round PTFE coated magnetic flea 15 x 4.5mm.

<u>0.01 M toluene/water</u>

To a 15 mL vial was added the desired surfactant (50 mg, 0.5 % w/w), Toluene (11 µL) and was made up to a desired volume (10 mL) with deionised water. This was left stirring overnight (16 hrs) at room temperature and 1000 RPM, using a slide round PTFE coated magnetic flea 15 x 4.5mm.

<u>0.05 M toluene/water</u>

To a 15 mL vial was added the desired surfactant (50 mg, 0.5 % w/w), Toluene (53 µL) and was made up to a desired volume (10 mL) with deionised water. This was left stirring overnight (16 hrs) at room temperature and 1000 RPM, using a slide round PTFE coated magnetic flea 15 x 4.5mm.

<u>0.1 M toluene/water</u>

To a 15 mL vial was added the desired surfactant (50 mg, 0.5 % w/w), Toluene (106 µL) and was made up to a desired volume (10 mL) with deionised water. This was left stirring overnight (16 hrs) at room temperature and 1000 RPM, using a slide round PTFE coated magnetic flea 15 x 4.5mm.

<u>0.15 M toluene/water</u>

To a 15 mL vial was added the desired surfactant (50 mg, 0.5 % w/w), Toluene (159 µL) and was made up to a desired volume (10 mL) with deionised water. This was left stirring overnight (16 hrs) at room temperature and 1000 RPM, using a slide round PTFE coated magnetic flea 15 x 4.5mm.

0.2 M toluene/water

To a 15 mL vial was added the desired surfactant (50 mg, 0.5 % w/w), Toluene (212 µL) and was made up to a desired volume (10 mL) with deionised water. This was left stirring overnight (16 hrs) at room temperature and 1000 RPM, using a slide round PTFE coated magnetic flea 15 x 4.5mm.

0.25 M toluene/water

To a 15 mL vial was added the desired surfactant (50 mg, 0.5 % w/w), Toluene (265 µL) and was made up to a desired volume (10 mL) with deionised water. This was left stirring overnight (16 hrs) at room temperature and 1000 RPM, using a slide round PTFE coated magnetic flea 15 x 4.5mm.

0.4 M toluene/water

To a 15 mL vial was added the desired surfactant (50 mg, 0.5 % w/w), Toluene (424 µL) and was made up to a desired volume (10 mL) with deionised water. This was left stirring overnight (16 hrs) at room temperature and 1000 RPM, using a slide round PTFE coated magnetic flea 15 x 4.5mm.

0.6 M toluene/water

To a 15 mL vial was added the desired surfactant (50 mg, 0.5 % w/w), Toluene (636 µL) and was made up to a desired volume (10 mL) with deionised water. This was left stirring overnight (16 hrs) at room temperature and 1000 RPM, using a slide round PTFE coated magnetic flea 15 x 4.5mm.

0.8 M toluene/water

To a 15 mL vial was added the desired surfactant (50 mg, 0.5 % w/w), Toluene (848 µL) and was made up to a desired volume (10 mL) with deionised water. This was left stirring overnight (16 hrs) at room temperature and 1000 RPM, using a slide round PTFE coated magnetic flea 15 x 4.5mm.

1.0 M toluene/water

11 µl To a 15 mL vial was added surfactant (50 mg, 0.5 % w/w), toluene (1060 µL) and deionised water (8940 µl). The vial was fitted with a PTFE septum (16mm) and a PTFE coated magnetic flea (12 mm) and was stirred overnight (16 hr).

1.1.1.2 Images of CPC in water

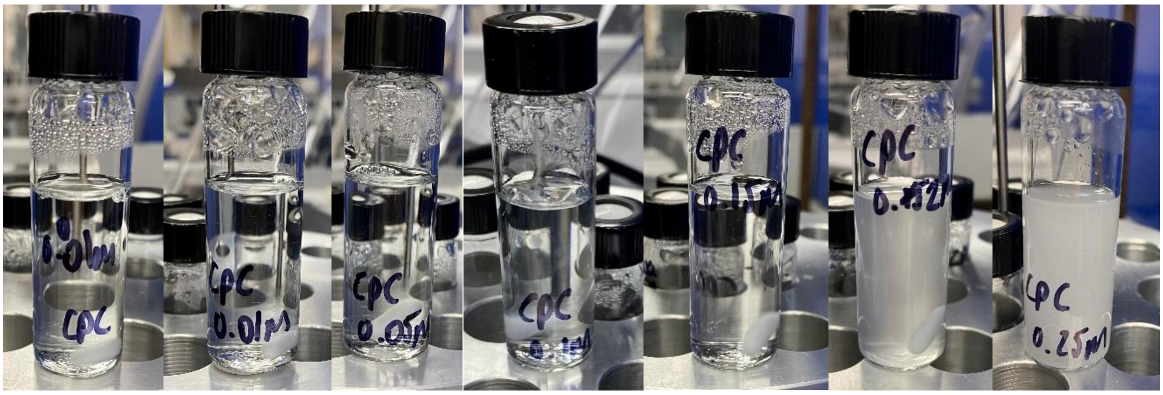

Figure S2. Physical appearance of vial containing CPC (0.5 % w/w) and 0.001 - 0.25 M toluene in deionised water.

1.1.1.3 Images of TPGS-750-M in water

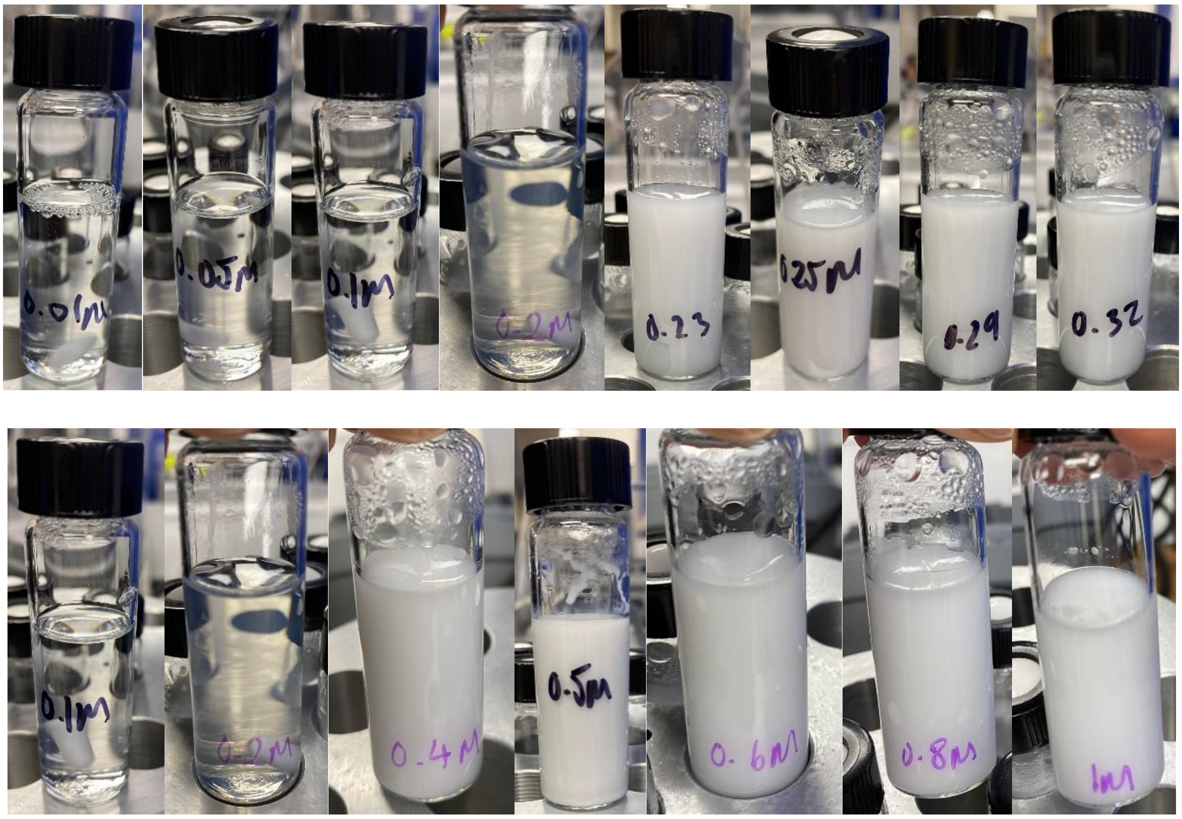

Figure S3. Physical appearance of vial containing TPGS-750-M (0.5 % w/w) and 0.01 – 1.0 M toluene in deionised water.

1.1.1.4 Images of Tween 80 in water

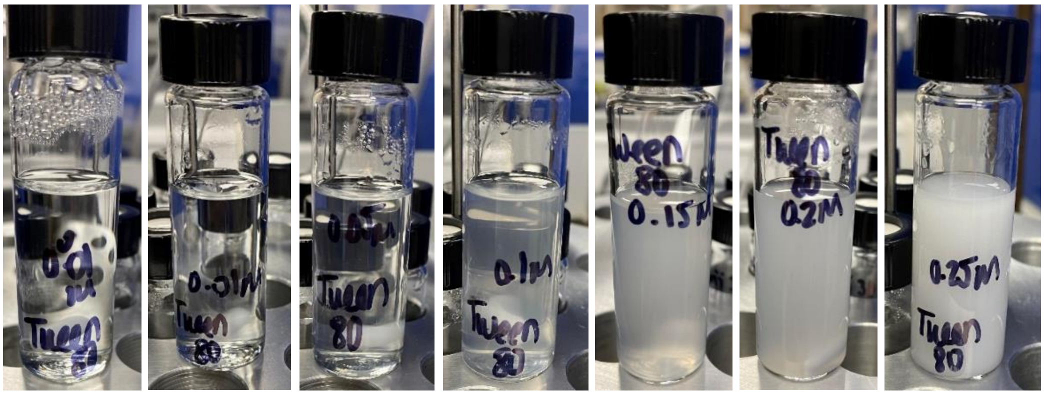

Figure S4. Physical appearance of vial containing Tween 80 (0.5 % w/w) and 0.001 - 0.25 M toluene in deionised water.

1.1.1.5 Images of sodium 4-noctylbenzenesulfonate SOBS in water

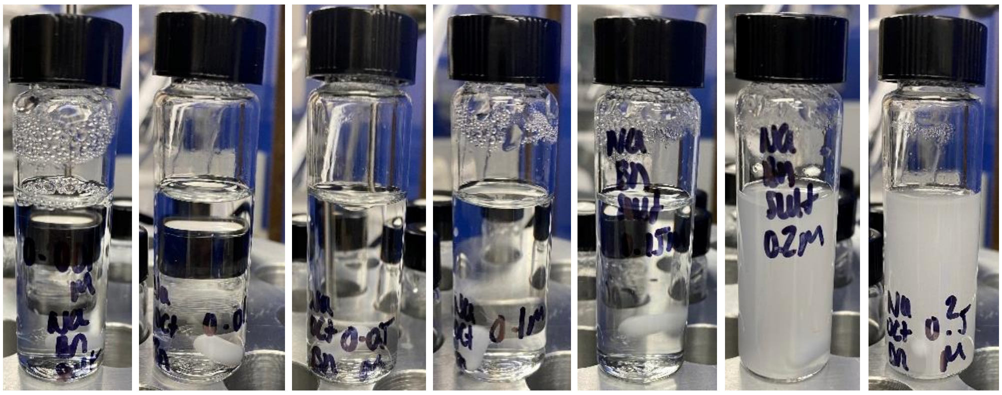

Figure S5. Physical appearance of vial containing SOBS (0.5 % w/w) and 0.001 - 0.25 M toluene in deionised water.

1.1.1.6 Images of Brij 35 in water

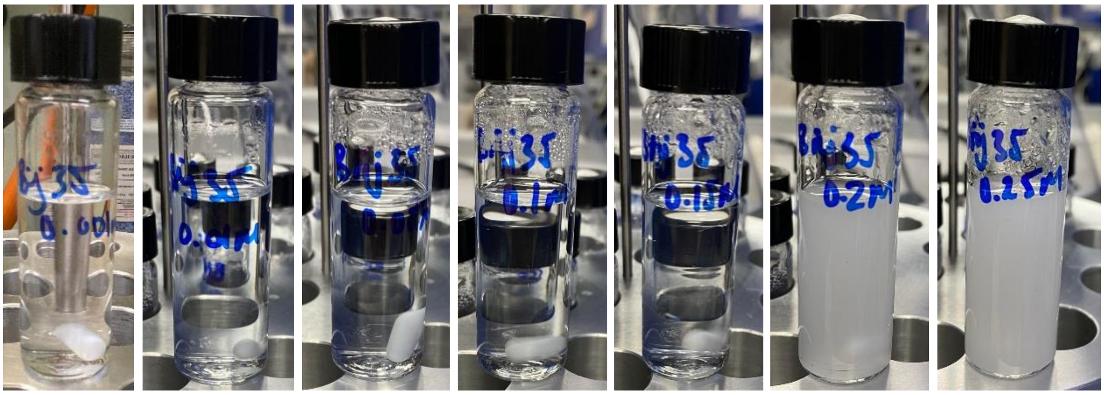

Figure S6. Physical appearance of vial containing Brij 35 (0.5 % w/w) and 0.01 - 0.25 M toluene in deionised water.

1.1.2 Dynamic light scattering measurements

All diameter measurements reported in this paper were measured using Zetasizer® Nano Range Analyzer (Nano-ZSP, Malvern Panalytical Instruments, UK), which uses a 4 mW He-Ne laser operating at a wavelength of 633 nm and a detection angle of 173°. The particle size is determined *via* the Brownian motion of the

dispersed phase by using the dynamic light scattering technique. The speed of the particles in a dispersed liquid are related to the particle size by the Stokes-Einstein equation.

$$D = \frac{k_B T}{6\pi\eta R_H} \tag{1}$$

In equation 1, D [m^2/s] is the translational diffusion coefficient and is effectively the "speed of the particles". K_B [m^2kg/Ks^2] is the Boltzmann constant, T [K] is the temperature, η [Pa.s] is the viscosity and R_H [m] is the hydrodynamic radius.

To a 15 mL vial was added the desired surfactant (50 mg, 0.5 % w/w), Toluene (0.00001 – 1 M) and was made up to a desired volume (10 mL) with deionised water. This was left stirring overnight (16 hrs) at room temperature and 1000 RPM, using a slide round PTFE coated magnetic flea 15 x 4.5mm. 2 mL aliquots were run on the Zetasizer using a 173° backscatter NIBS default setting, an equilibrium period of 120 secs, in quartz glass cuvettes (3.5 mL). 2 consecutive repeats of each aliquot were run and each concentration was measured in triplicate. Vials were continuously stirred until all aliquots had been withdrawn.

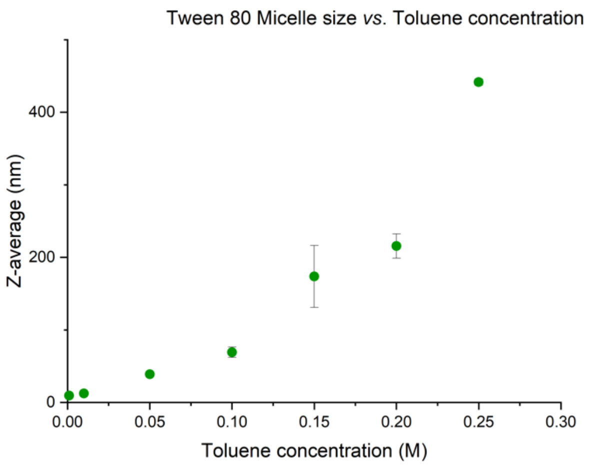

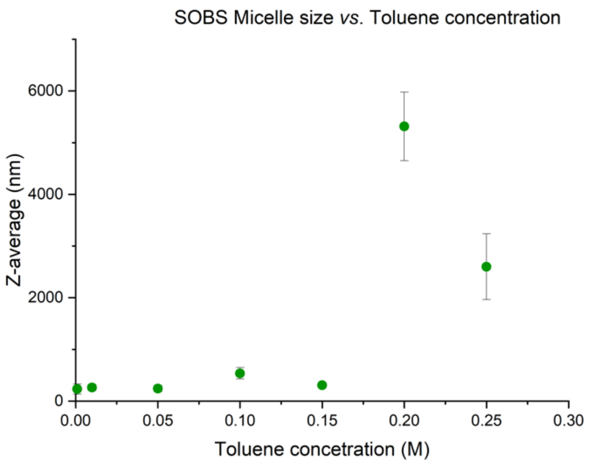

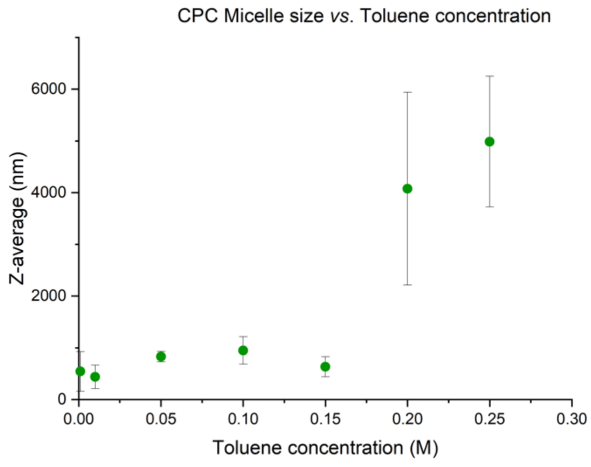

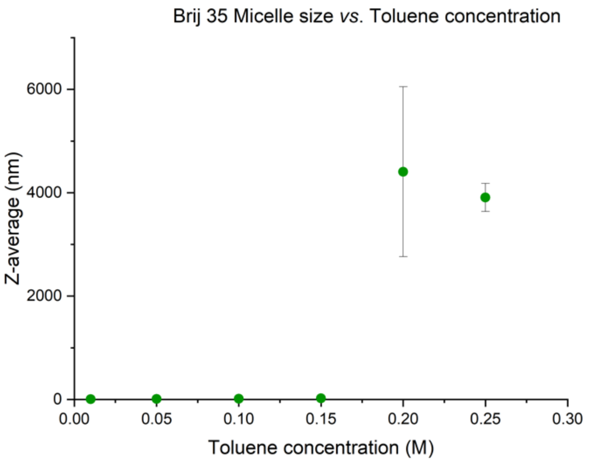

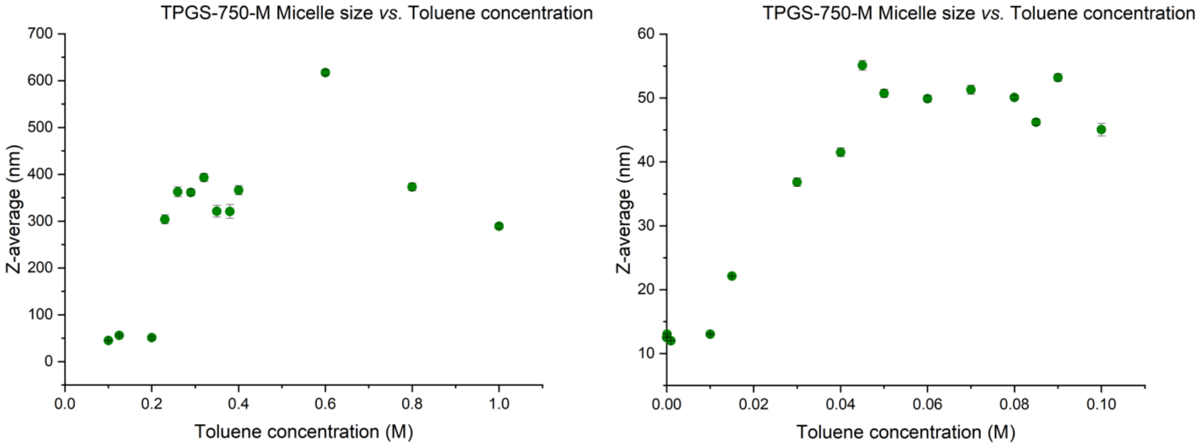

Figure S7. Graphs of several surfactants Z-average *vs*. Toluene concentration graphs.

Table S1. CPC dynamic light scattering results 0.001 – 0.25 M

Concentration toluene (M)	Mean (nm)	SD (nm)	Average PdI
0.001	543.7	383.2	0.652
0.01	438.7	227.0	0.621
0.05	830.8	100.9	0.698
0.1	950.2	265.0	0.776
0.15	633.8	193.5	0.595
0.2	4076	1863	0.707
0.25	4985	1263	0.673

Table S2. Brij 35 dynamic light scattering results 0.001 – 0.25 M

Concentration toluene (M)	Mean (nm)	SD (nm)	Average PdI
0.001	9.2	0.44	0.211
0.01	8.5	0.04	0.132
0.05	9.7	0.08	0.294
0.1	15.2	0.05	0.543
0.15	23.2	1.50	0.727
0.2	4407	1643	0.358
0.25	3909	270	1.00

Table S3. SOBS dynamic light scattering results 0.001 – 0.25 M

Concentration toluene (M)	Mean (nm)	SD (nm)	Average PdI
0.001	234.2	98.7	0.462
0.01	264.6	59.8	0.422
0.05	245.2	61.5	0.469
0.1	538.4	108.5	0.545
0.15	308.0	46.7	0.476
0.2	5315	662	0.341
0.25	2600	637	0.618

Table S4. Tween 80 dynamic light scattering results 0.001 – 0.25 M

Concentration toluene (M)	Mean (nm)	SD (nm)	Average PdI
0.001	9.4	0.04	0.0407
0.01	12.4	0.2	0.372
0.05	38.9	1.1	1.00
0.1	69.2	7.2	1.00
0.15	174	42.8	0.726
0.2	216	16.8	0.944
0.25	442	4.4	0.567

Table S5. TPGS-750-M dynamic light scattering results 0.00001 – 0.1 M

Concentration toluene (M)	Mean (nm)	SD (nm)	Average PdI
1×10^{-5}	12.5	0.03	0.136
1×10^{-4}	13.0	0.1	0.172
0.001	12.0	0.1	0.116
0.01	13.0	0.1	0.233

0.015	22.1	0.02	0.228
0.03	36.8	0.7	0.227
0.04	41.5	0.7	0.229
0.045	55.1	0.8	0.313
0.05	50.7	0.6	0.298
0.06	49.9	0.5	0.254
0.07	51.3	0.7	0.263
0.08	50.1	0.3	0.259
0.085	46.2	0.5	0.244
0.09	53.2	0.6	0.284
0.1	45.1	1.0	0.233

Table S6. TPGS-750-M dynamic light scattering results 0.1 - 1.0 M

**Concentration toluene (M)**	**Mean (nm)**	**SD (nm)**	**Average PdI**
0.1	45.1	1.0	0.233
0.125	56.0	1.8	0.400
0.2	51.1	2.8	0.369
0.23	304	9.5	0.594
0.26	363	10.6	0.473
0.29	362	7.1	0.582
0.32	393	8.1	0.624
0.35	321	12.4	0.520
0.38	321	14.6	0.534
0.4	366	9.8	0.563
0.6	617	5.7	0.233
0.8	373	7.9	0.115

1	289	4.7	0.148

1.1.3 Cryo-SEM

Samples were attached to an EM stub and frozen by plunging into a slushed nitrogen. They were transferred under vacuum into a Quorum PP3010 cryo-preparation system attached to an FEI Helios G4 CX Dualbeam. Samples were sublimed in the station by heating to −90 °C for 3 min and then re-cooled to −140 °C. They were then sputter coated with Ir in an argon environment (5 mA for 45 s) to make them conductive. Samples were transferred from the preparation chamber to the cryostage in the microscope and held at −140 °C. Imaging was performed using a 2 kV landing energy and 0.1nA current.

<u>Sample preparation</u>

To a 15 mL vial was added the desired surfactant (50 mg, 0.5 % wt/wt), Toluene (0.05 – 0.4 M) and was made up to a desired volume (10 mL) with deionised water. This was left stirring overnight (16 hrs) at room temperature and 1000 RPM, using a slide round PTFE coated magnetic flea 15 x 4.5mm.

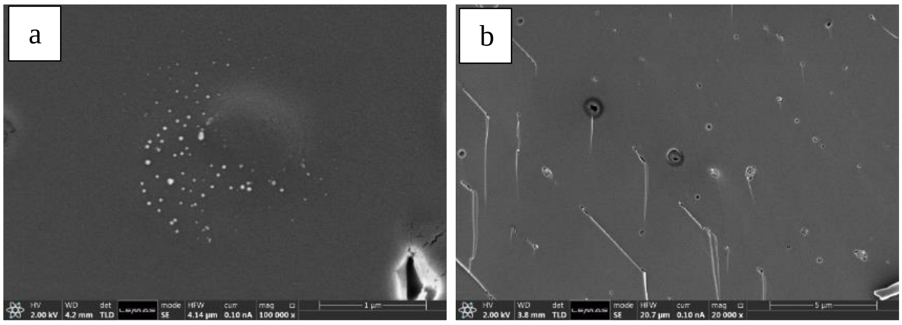

Figure S8. Cryo-SEM images of a) 0.05 M Toluene and b) 0.25 M Toluene in TPGS-750-M/H_2O 0.5% wt/wt; scale bars represent a) 1 µm and b) 5 µm.

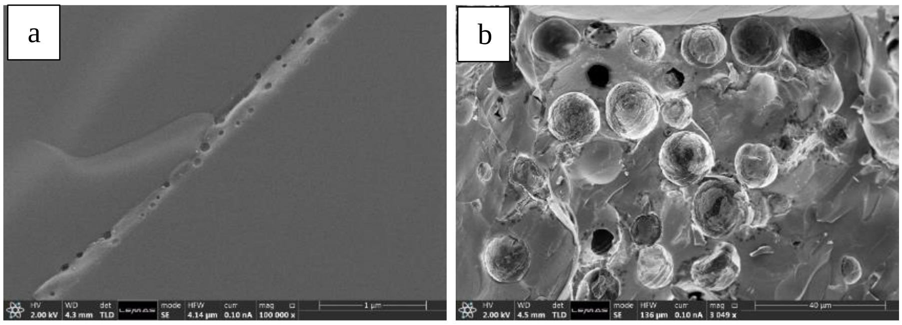

Figure S9. Cryo-SEM images of a) 0.05 M Toluene and b) 0.25 M Toluene in Brij 35/H_2O 0.5% wt/wt; scale bars represent a) 1 µm and b) 40 µm.

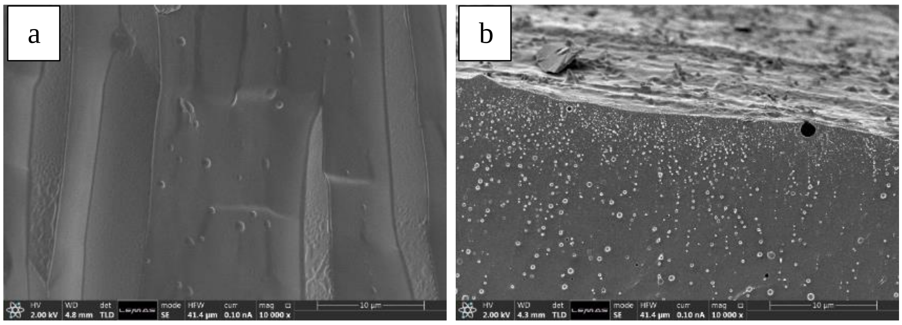

Figure S10. Cryo-SEM images of a) 0.05 M Toluene and b) 0.25 M Toluene in Tween 80/H_2O 0.5% wt/wt; scale bars represent a) 10 µm and b) 10 µm.

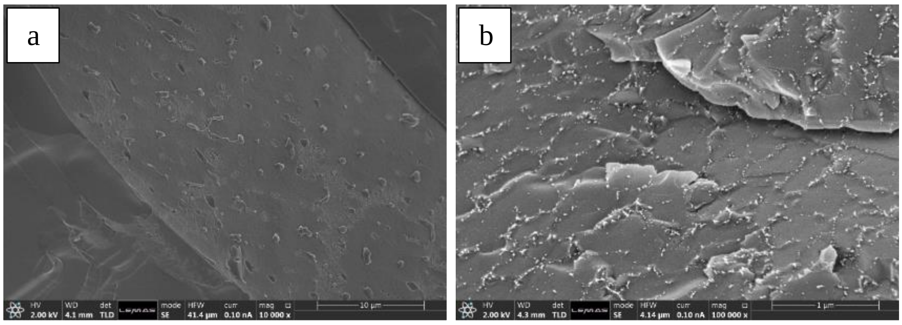

Figure S11. Cryo-SEM images of a) 0.05 M Toluene and b) 0.25 M Toluene in CPC/H_2O 0.5% wt/wt: scale bars represent a) 10 µm and b) 1 µm.

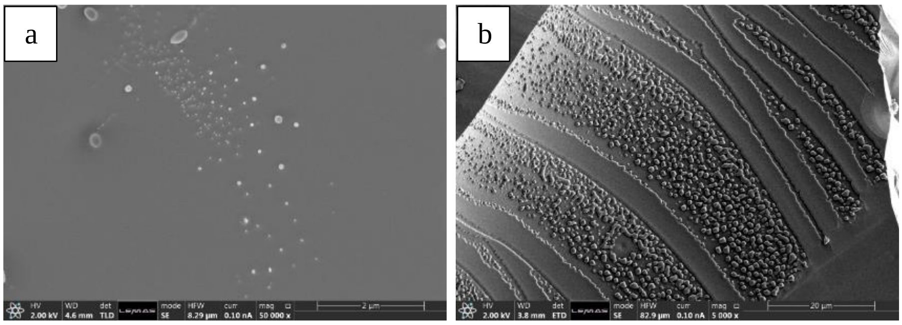

Figure S12. Cryo-SEM images of a) 0.05 M Toluene and b) 0.25 M Toluene in SOBS/H_2O 0.5% wt/wt: scale bars represent a) 2 µm and b) 20 µm.

1.1.4 Microscope imaging

To a 15 mL vial was added the desired surfactant (50 mg, 0.5 % w/w), Toluene (0.05 – 0.4 M) and was made up to a desired volume (10 mL) with deionised water. This was left stirring overnight (16 hrs) at room temperature and 1000 RPM, using a slide round PTFE coated magnetic flea 15 x 4.5mm. Microscope imaging was conducted using an AmScope ×40–1000 LED Lab Binocular Compound w/ 3D Two-Layer Mechanical Stage SKU: B120, with resolution > 0.2 µm. Multiple pictures (5 – 10) were taken of each solution so to capture at least 100 particles for analysis.

Size analysis was conducted using ImageJ. Pictures were converted to an 8 GIB image. A Bandpass filter was applied to the image and the threshold adjusted to aid in particle identification by software. The size distribution of particles was conducted using the outlines of the particles with a circularity limit set from 0.1 – 1.0. The diameters of the particles were calculated in nm from the given areas of the particle size analysis.

1.1.1.7 TPGS-750-M 0.4M Toluene/H_2O

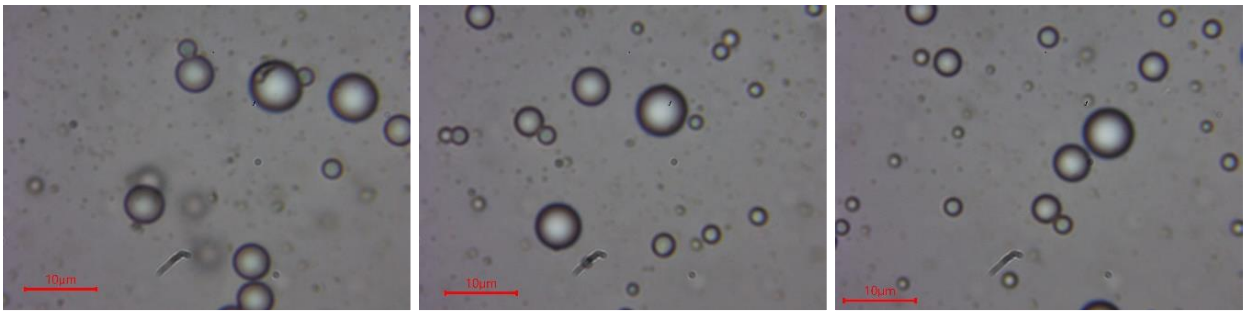

Figure S13. Microscope images of 0.4M Toluene in TPGS-750-M/H_2O; red bars represent 10 microns

1.1.1.8 TPGS-750-M 0.25M Toluene/H_2O

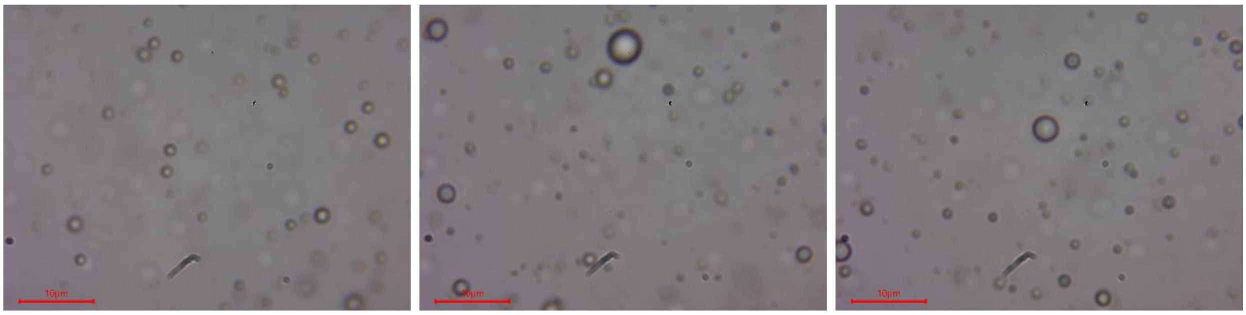

Figure S14. Microscope images of 0.25M Toluene in TPGS-750-M/H_2O; red bars represent 10 microns

1.1.1.9 CPC 0.25M Toluene/H_2O

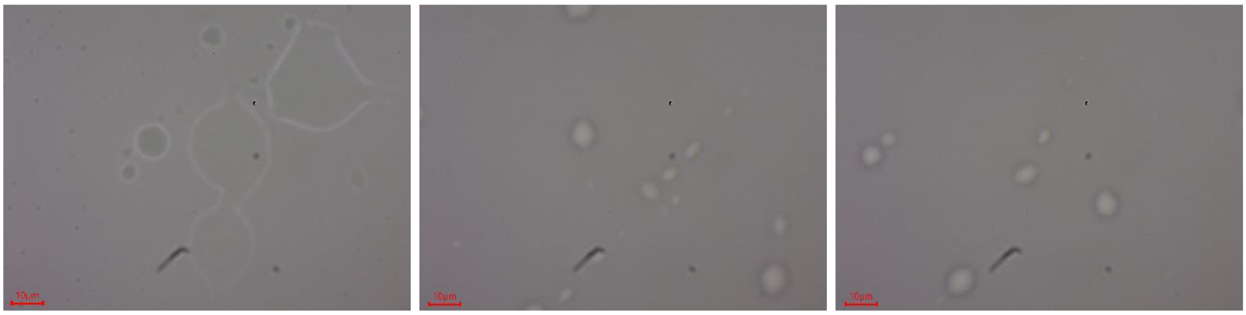

Figure S15. Microscope images of 0.25M Toluene in CPC/H_2O; red bars represent 10 microns

1.1.1.10 SOBS 0.25M Toluene/H_2O

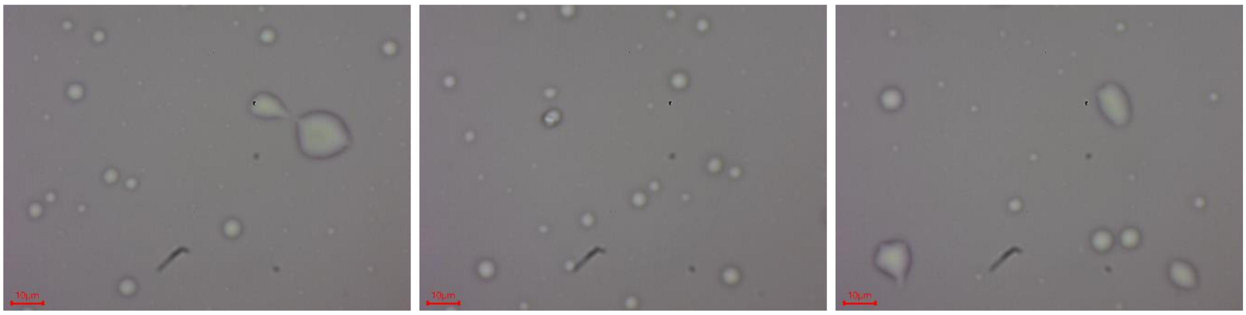

Figure S16. Microscope images of 0.25M Toluene in SOBS/H_2O; red bars represent 10 microns

1.1.1.11 Brij 35 0.25M Toluene/H_2O

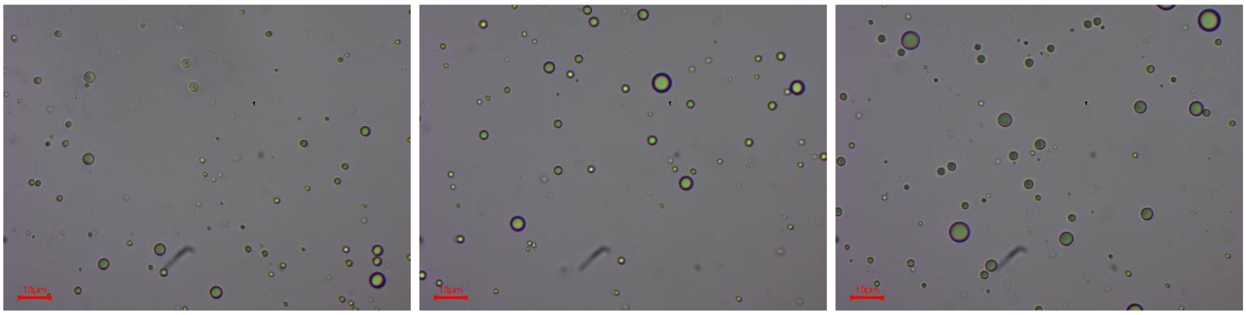

Figure S17. Microscope images of 0.25M Toluene in Brij 35/H_2O; red bars represent 10 microns

1.1.1.12 Brij S20 0.25M Toluene/H_2O

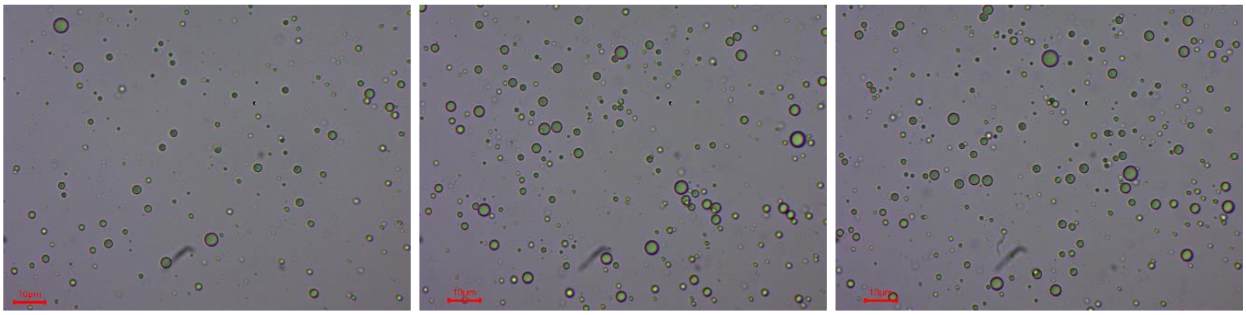

Figure S18. Microscope images of 0.25M Toluene in Brij S20/H_2O; red bars represent 10 microns

1.1.1.13 Tween 80 0.25M Toluene/H_2O

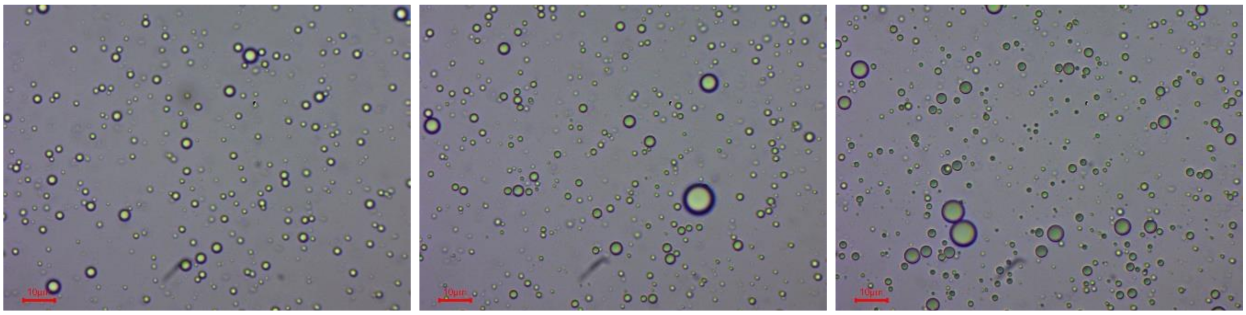

Figure S19. Microscope images of 0.25M Toluene in Tween 80/H_2O; red bars represent 10 microns

1.1.5 Size comparison between different techniques

Table S7. Summary of size observed using DLS, optical imaging and cryo-SEM for 0.05 and 0.25 M toluene in TPGS-750-M/H_2O 0.5% wt/wt.

	Size observed toluene in H_2O 0.05 M (nm)	Size observed toluene in H_2O 0.25 M (nm)
DLS	50.7	363[a]
Optical imaging	–	797 – 4213
Cryo-SEM	19 – 569	35 – 860

[a]toluene concentration of 0.26 M was used.

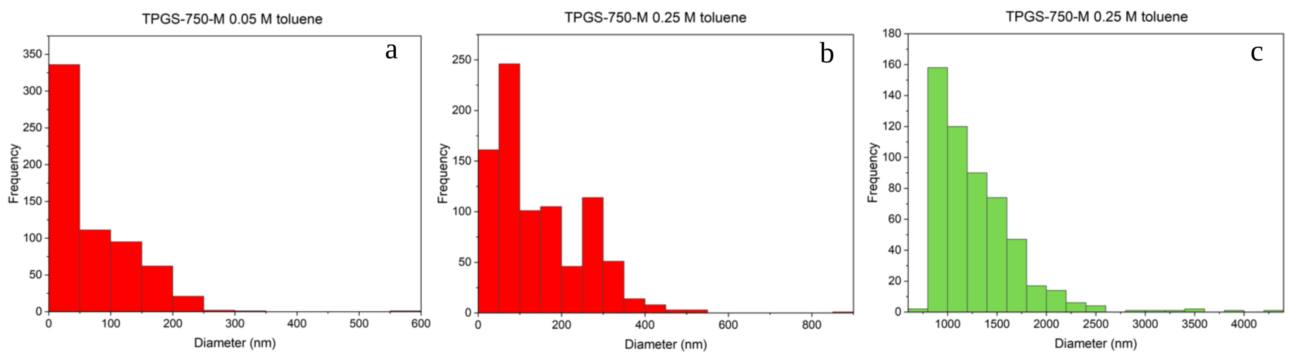

Figure S20. Size histograms of TPGS-750-M/H_2O 0.5 % wt/wt of a) 0.05 M, b) 0.25 M toluene by cryo-SEM and c) 0.25 M toluene by optical imaging.

Table S8. Summary of size observed using DLS, optical imaging and cryo-SEM for 0.05 and 0.25 M toluene in Brij 35/H_2O 0.5% wt/wt

	Size observed toluene in H_2O 0.05 M (nm)	Size observed toluene in H_2O 0.25 M (nm)
DLS	9.7	3909
Optical imaging	–	800 – 5835
Cryo-SEM	3 – 87	192 – 535

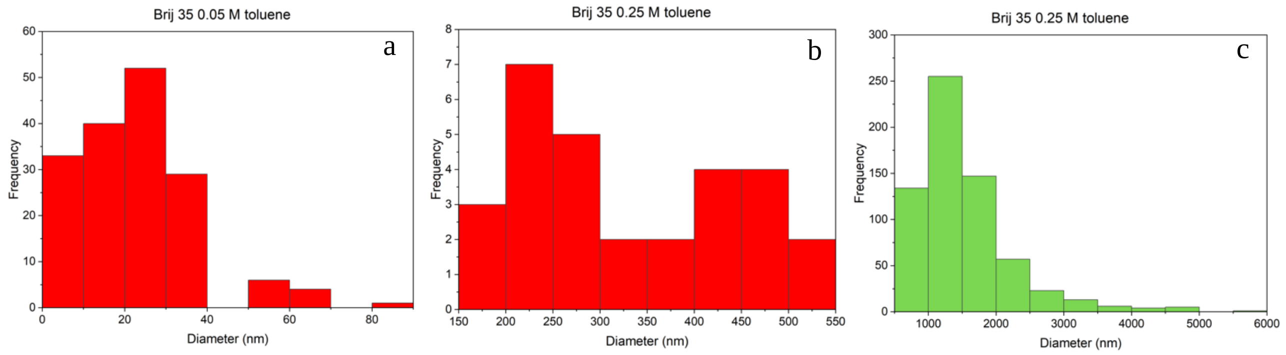

Figure S21. Size histograms of Brij 35/H_2O 0.5 % wt/wt of a) 0.05 M, b) 0.25 M toluene by cryo-SEM and c) 0.25 M toluene by optical imaging.

Table S9. Summary of size observed using DLS, optical imaging and cryo-SEM for 0.05 and 0.25 M toluene in Tween 80/H_2O 0.5% wt/wt

	Size observed toluene in H_2O 0.05 M (nm)	Size observed toluene in H_2O 0.25 M (nm)
DLS	38.9	442
Optical imaging	–	799 – 5548

Cryo-SEM	35 – 932	112 – 673

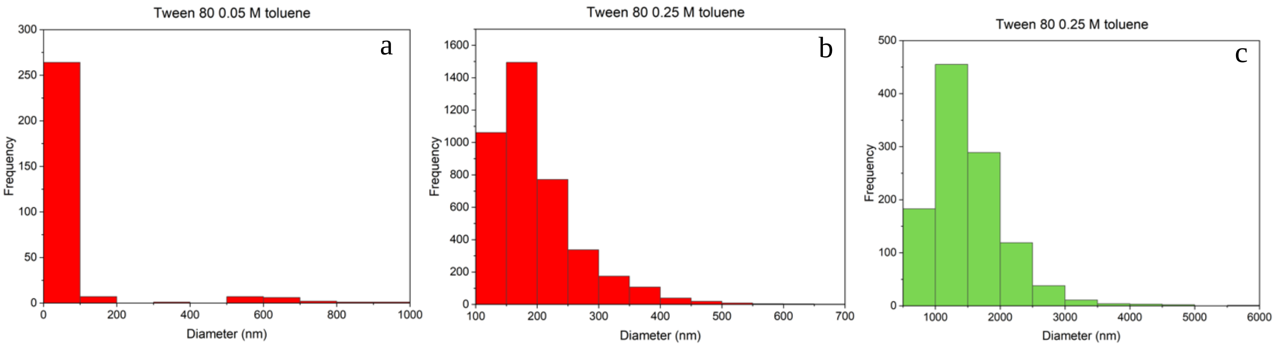

Figure S22. Size histograms of Tween 80/H_2O 0.5 % wt/wt of a) 0.05 M, b) 0.25 M toluene by cryo-SEM and c) 0.25 M toluene by optical imaging.

Table S10. Summary of size observed using DLS, optical imaging and cryo-SEM for 0.05 and 0.25 M toluene in CPC/H_2O 0.5% wt/wt

	Size observed toluene in H_2O 0.05 M (nm)	Size observed toluene in H_2O 0.25 M (nm)
DLS	830.8	4985
Optical imaging	–	803 – 5600
Cryo-SEM	225 – 1272	11 – 50

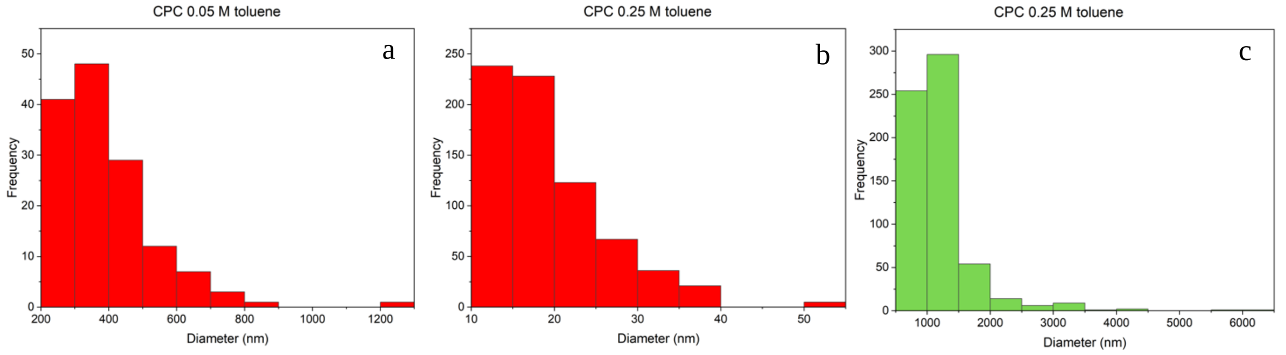

Figure S23. Size histograms of CPC/H_2O 0.5 % wt/wt of a) 0.05 M, b) 0.25 M toluene by cryo-SEM and c) 0.25 M toluene by optical imaging.

Table S11. Summary of size observed using DLS, optical imaging and cryo-SEM for 0.05 and 0.25 M toluene in SOBS/H_2O 0.5% wt/wt.

	Size observed toluene in H_2O 0.05 M (nm)	Size observed toluene in H_2O 0.25 M (nm)
DLS	245.2	2600

Optical imaging	–	805 – 7137
Cryo-SEM	6 – 254	257 – 1323

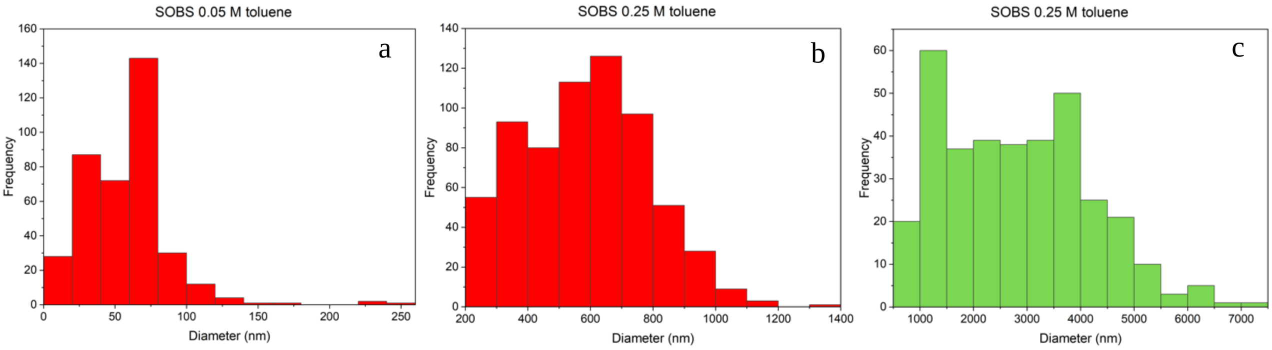

Figure S24. Size histograms of SOBS/H_2O 0.5 % wt/wt of a) 0.05 M, b) 0.25 M toluene by cryo-SEM and c) 0.25 M toluene by optical imaging.

1.2 Studies of reaction (3)

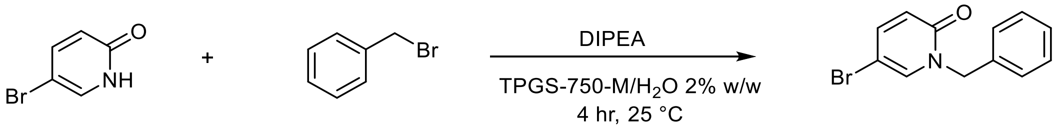

Scheme S1. *N*-alkylation reaction between 5-bromo-2(1H)-pyridone and benzyl bromide.

1.2.1 Microscope imaging of reaction (3) with TPGS-750-M surfactant

The N-alkylation reaction in Scheme S1 was monitored over time through microscope imaging of the reaction mixture. To a 15 mL vial was added, TPGS-750-M (60 mg, 2 % w/w) and deionised water (3 mL). This was stirred for 1 hour using a slide round PTFE coated magnetic flea 15 x 4.5mm at 1000 RPM and 25 °C. To the same vial was added 5-bromo-2(1H)-pyridone (75 mg, 0.431 mmol) and benzyl bromide (105 µL, 0.862 mmol) and the reaction was stirred for a further 15 minutes. DIPEA (150 µL, 0.862mmol) was added to the vial and the reaction was stirred for 4 hours, at 700 RPM and 25 °C. Small aliquots were removed at given time points (60, 120, 180 and 240 minutes) from the reaction using a glass pipette and transferred to a microscope slide.

<u>60 minutes</u>

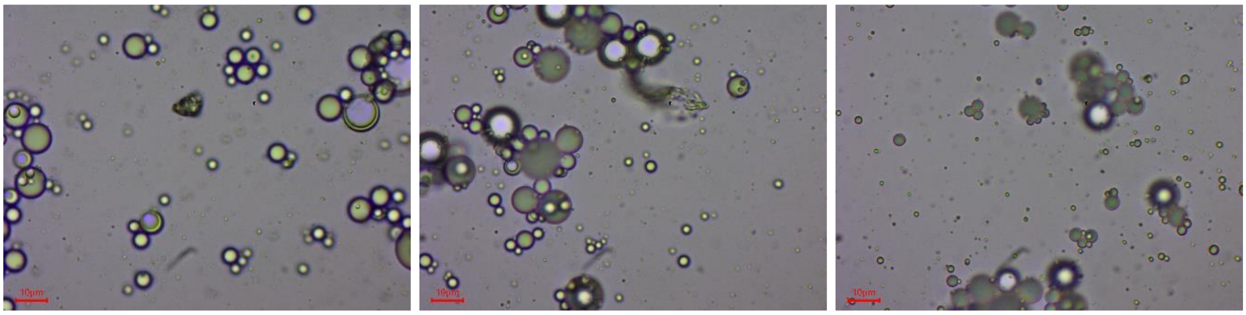

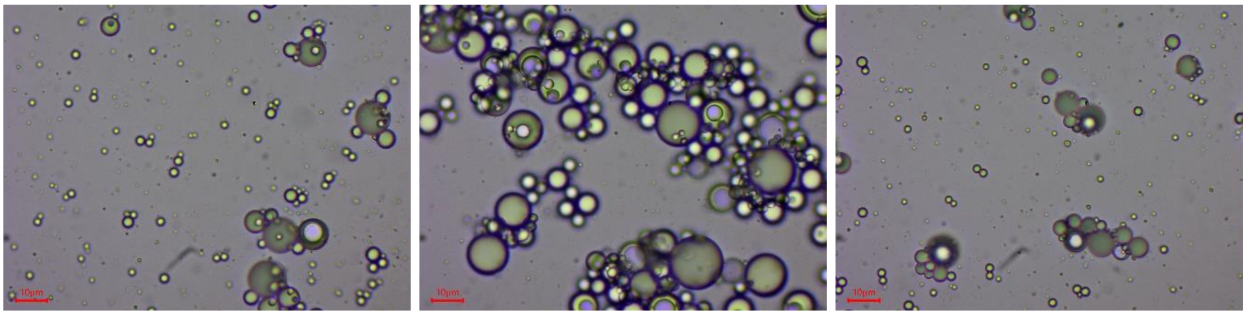

Figure S25. Microscope images of reaction (3) using TPGS-750-M at 60 mins; red bars represent 10 microns

<u>120 minutes</u>

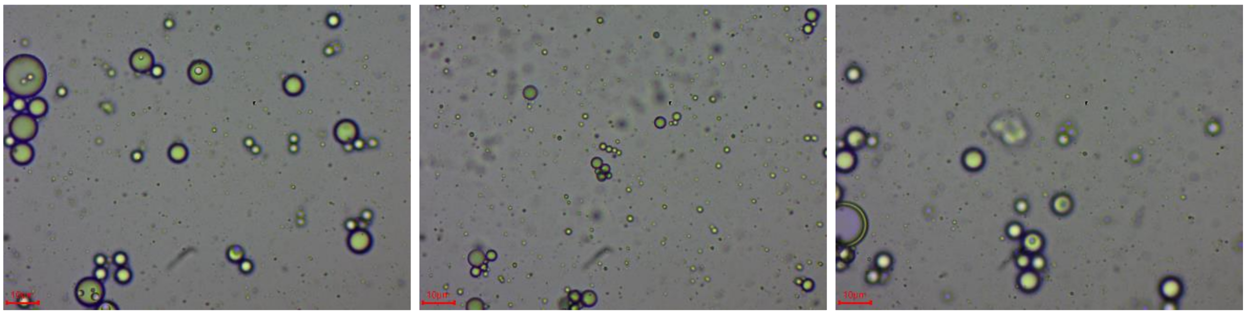

Figure S26. Microscope images of reaction (3) using TPGS-750-M at 120 mins; red bars represent 10 microns

<u>180 minutes</u>

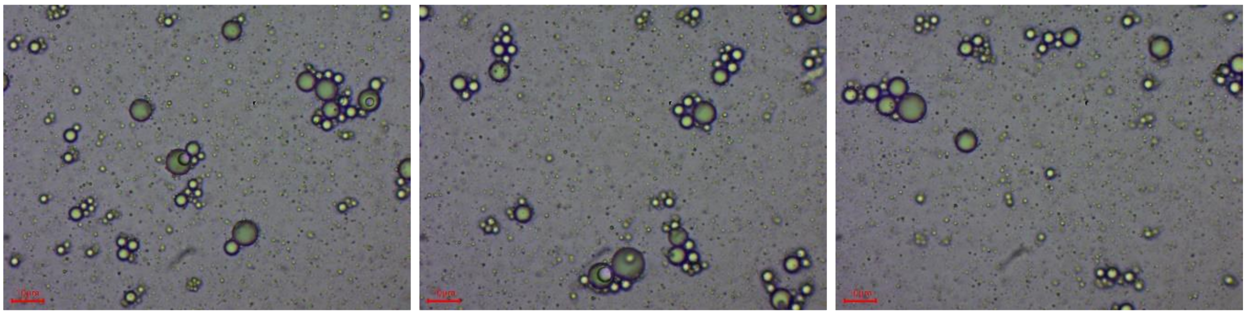

Figure S27. Microscope images of reaction (3) using TPGS-750-M at 180 mins; red bars represent 10 microns

<u>240 minutes</u>

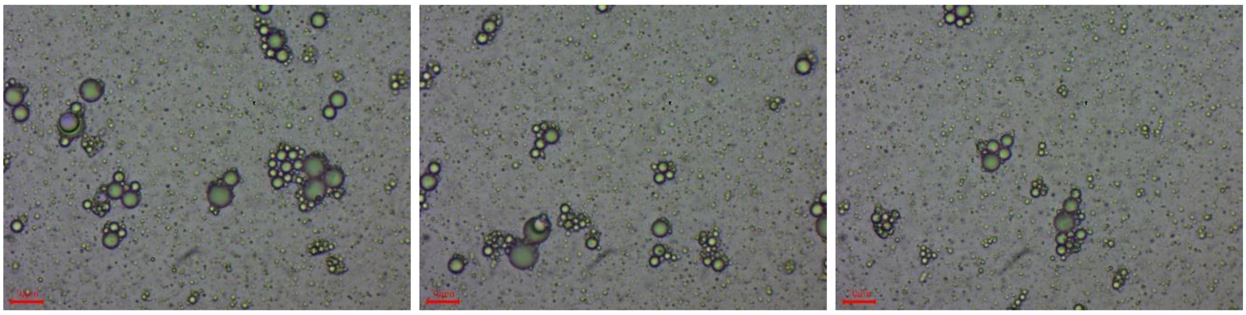

Figure S 28. Microscope images of reaction (3) using TPGS-750-M at 240 mins; red bars represent 10 microns

1.3 NMR studies of reaction (3)

Nuclear magnetic resonance (^1H) analysis was carried out on Bruker DRX 500 spectrometer. Shifts (δ) were given in ppm and locked to the solvent peaks.

Sample preparation in D_2O

To a 5 mL vial was added reaction component (15 mg) and D_2O (3 mL) and the vial was stirred for at least ~ 30 minutes using a slide round PTFE coated magnetic flea 15 x 4.5mm at room temperature and 600 RPM. A sample of the solution (~0.6 mL) was transferred from the vial into an NMR tube and ^1H NMR was conducted on the solution. For some samples solids still remained in the mixture so these solids were removed before transferring to the NMR tube by filtering the sample through a Pasteur pipette equipped with some cotton wool.

Sample preparation in cyclohexane-d_{12}

To a 5 mL vial was added reaction component (15 mg) and cyclohexane-d_{12} (3 mL) and the vial was stirred for at least ~ 30 minutes using a slide round PTFE coated magnetic flea 15 x 4.5mm at room temperature and 700 RPM. A sample of the solution (~0.6 mL) was transferred from the vial into an NMR tube and ^1H NMR was conducted on the mixture. For some samples solids still remained in the mixture so these solids were removed before transferring to the NMR tube by filtering the sample through a Pasteur pipette equipped with some cotton wool.

Sample preparation for surfactant in D_2O

To a 5 mL vial was added surfactant (80 mg, 2.7 % w/w) or (160 mg, 5.3 % w/w) and D_2O (3 mL) and the vial was stirred for at least 3 hours using a slide round PTFE coated magnetic flea 15 x 4.5mm at room temperature and 700 RPM. Depending on which component of the reaction was being studied this was added to the vial (15 mg) and the vial was stirred for a further 30 minutes. A sample of the solution (~0.6 mL) was transferred from the vial into an NMR tube and ^1H NMR was conducted on the mixture. For some samples the reaction component had not fully dissolved so these solids were removed before transferring to the NMR tube by filtering the sample through a Pasteur pipette equipped with some cotton wool.

1.3.1 ¹H NMR spectra of 5-bromo-2(1H)-pyridone

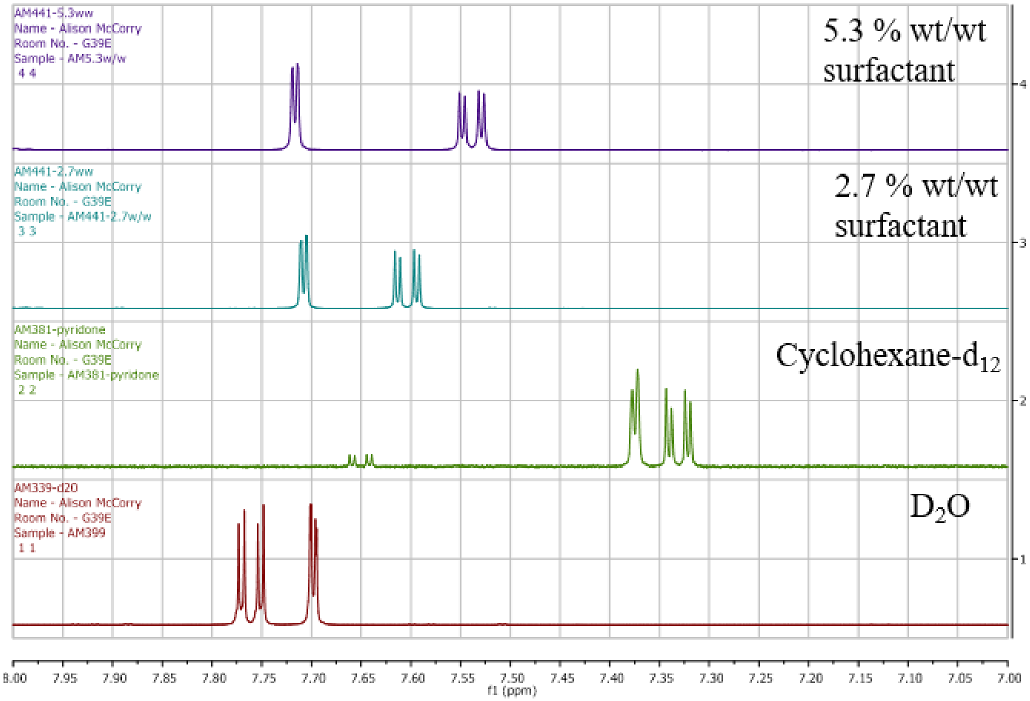

Figure S29. Spectra with CPC surfactant

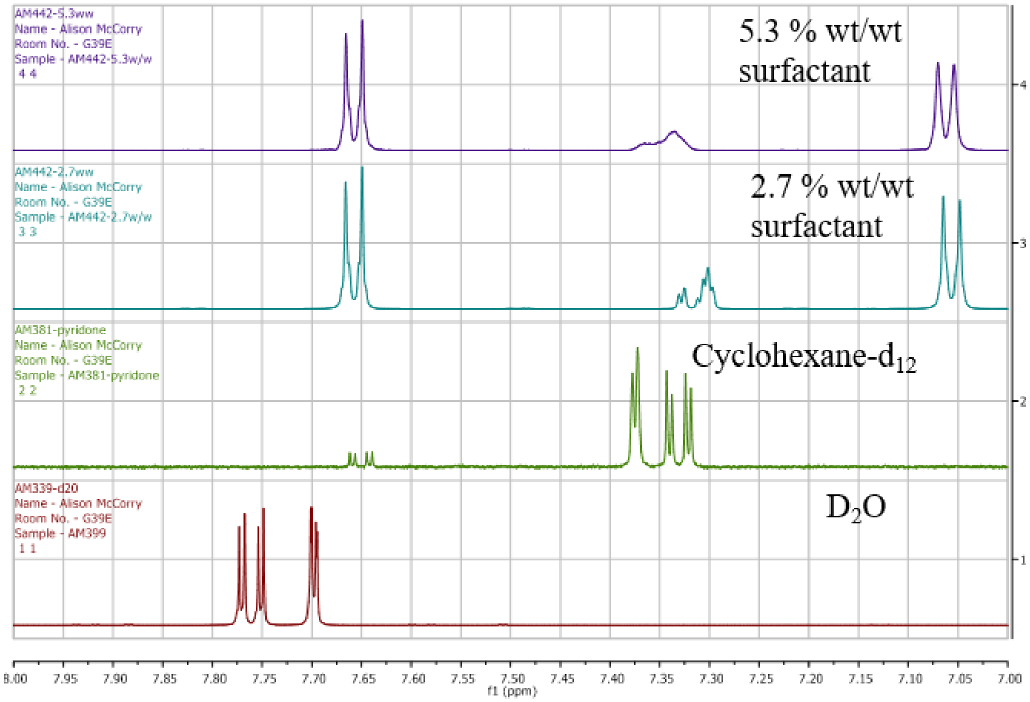

Figure S30. Spectra with SOBS surfactant

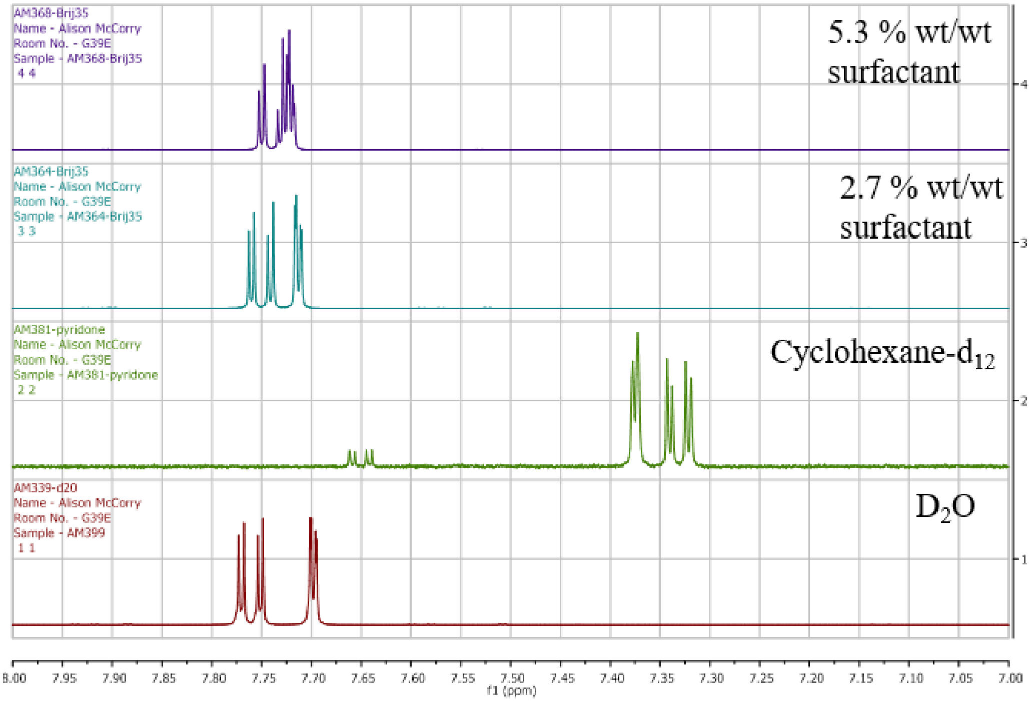

Figure S31. Spectra with Brij 35 surfactant

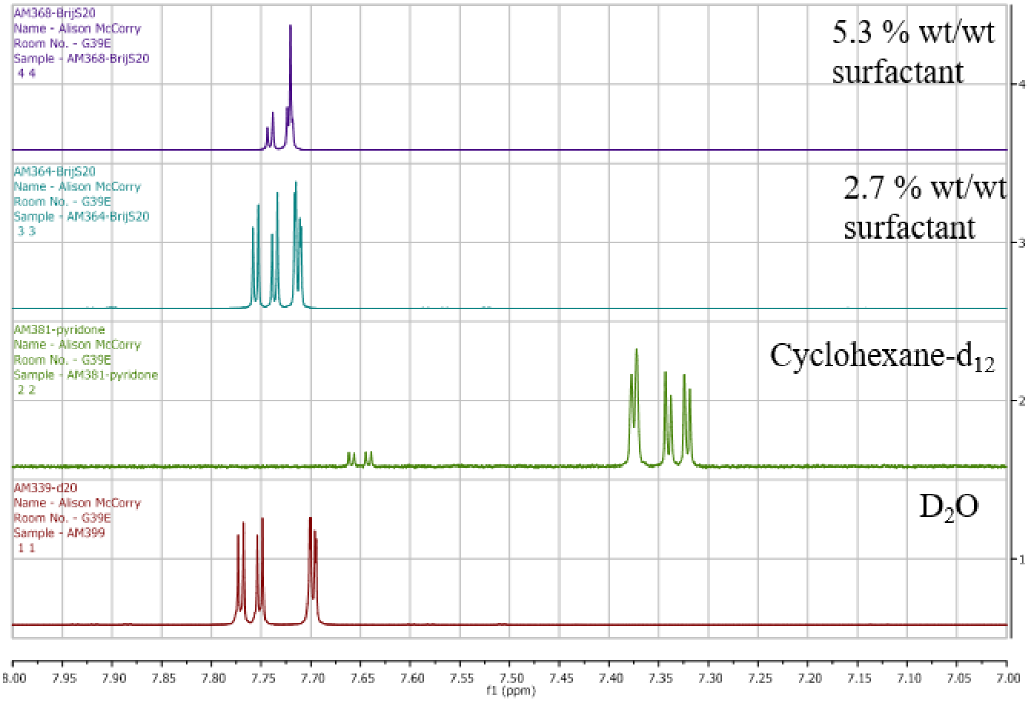

Figure S32. Spectra with Brij S20 surfactant

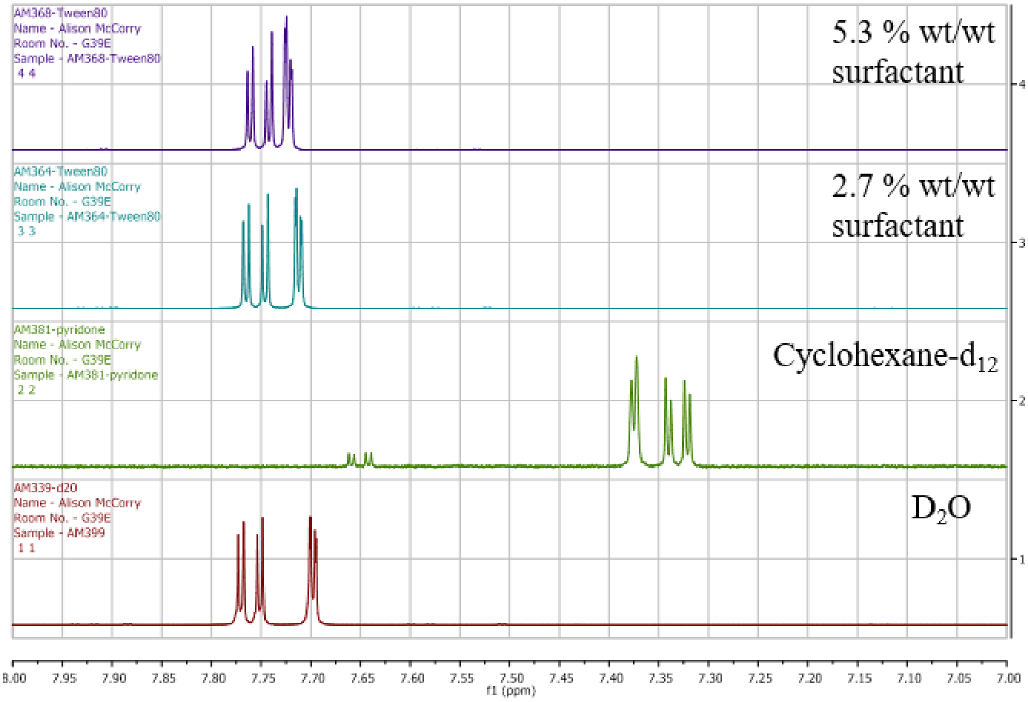

Figure S33. Spectra with Tween 80 surfactant

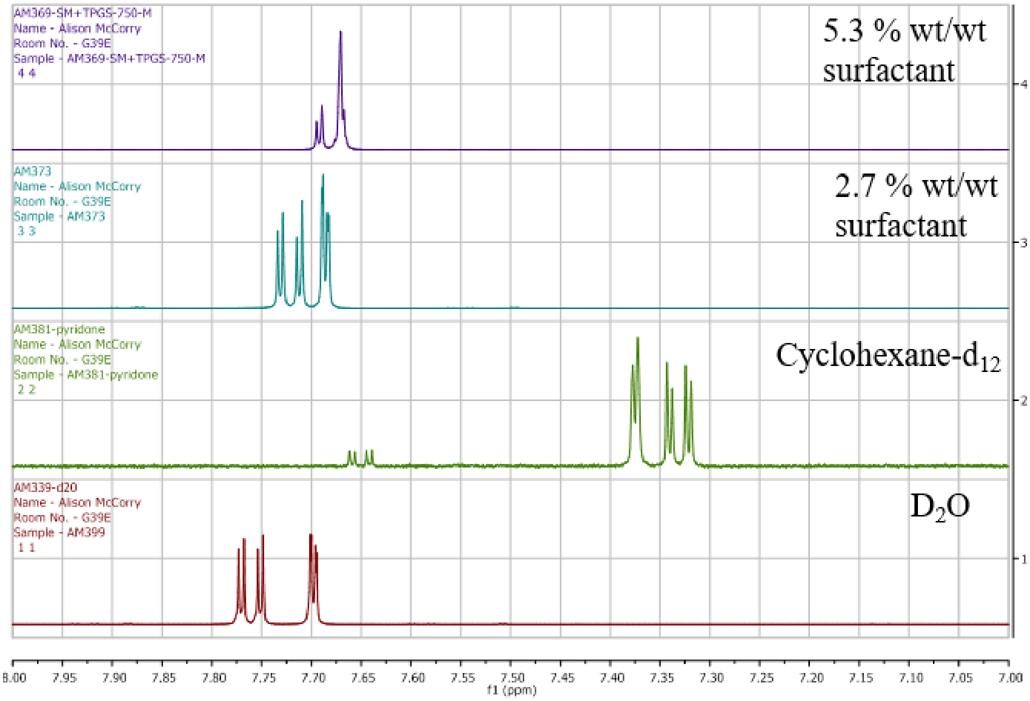

Figure S34. Spectra with TPGS-750-M surfactant

1.3.2 ^{1}H NMR spectra of benzyl bromide

Benzyl bromide was observed to have poor solubility in D_2O alone therefore, DMSO-d_6 was added gradually to obtain a representative spectrum.

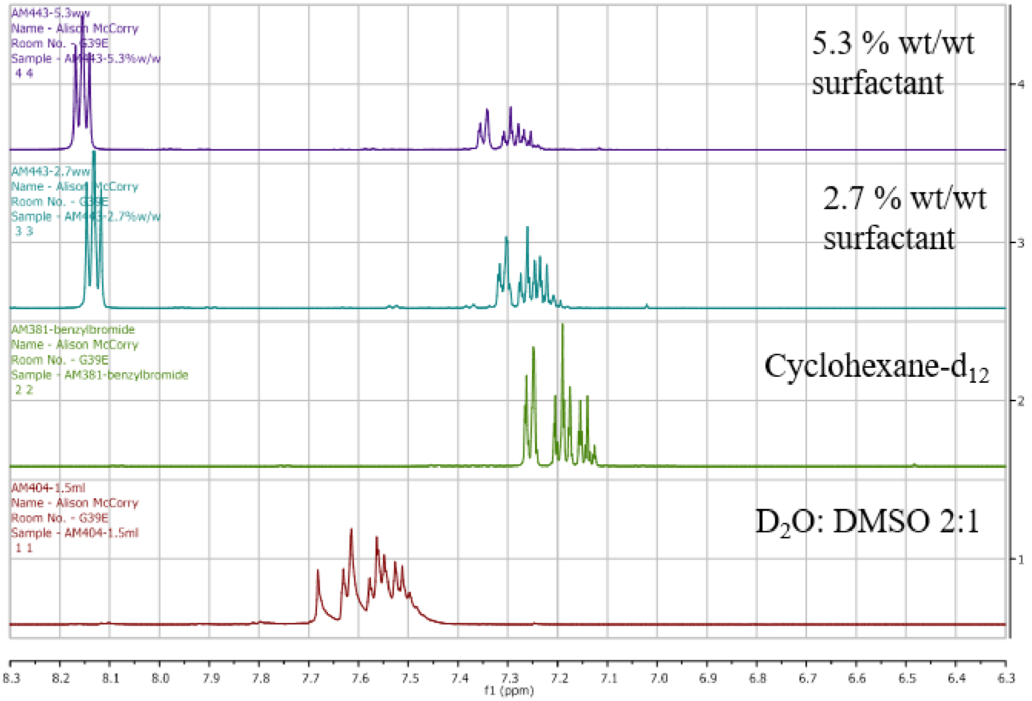

Figure S35. Spectra with CPC surfactant

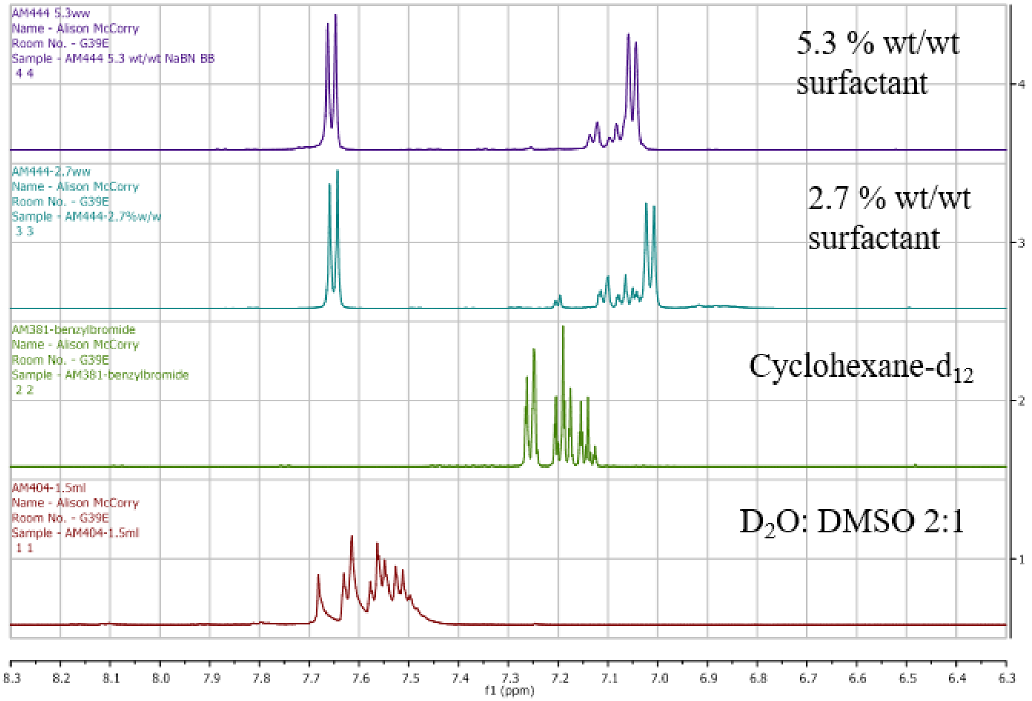

Figure S36. Spectra with SOBS surfactant

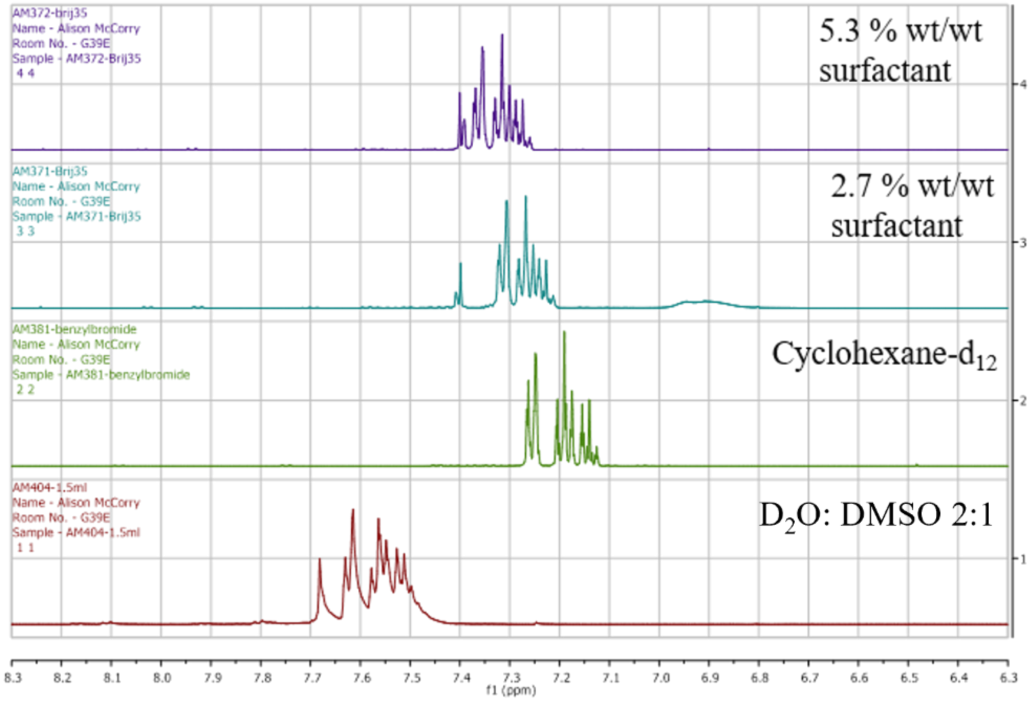

Figure S37. Spectra with Brij 35 surfactant

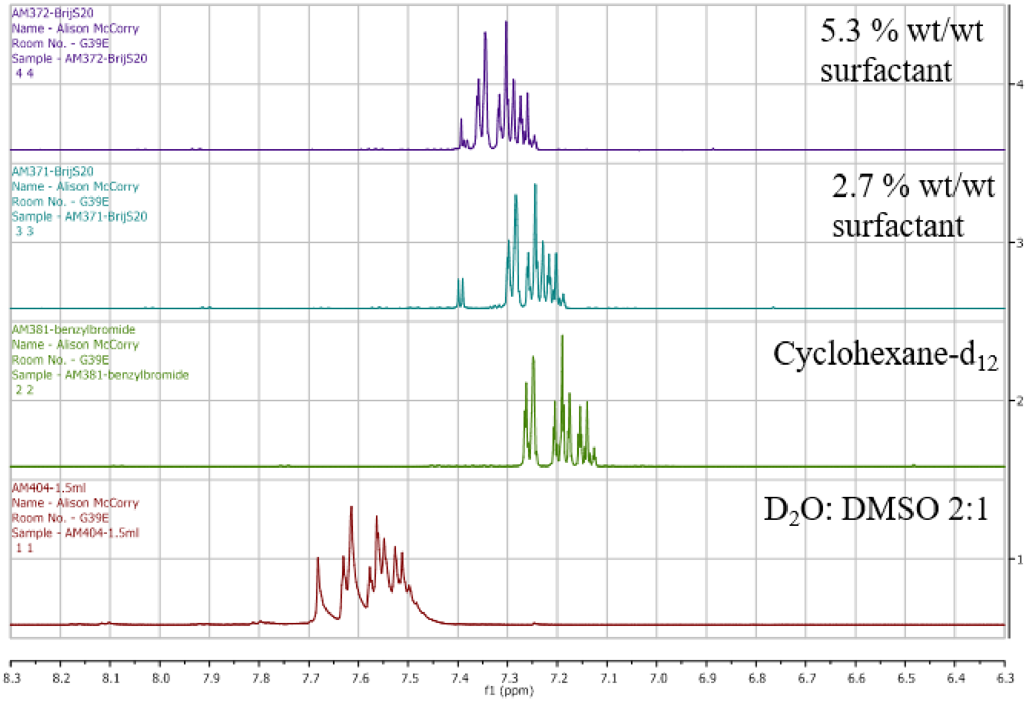

Figure S38. Spectra with Brij S20 surfactant

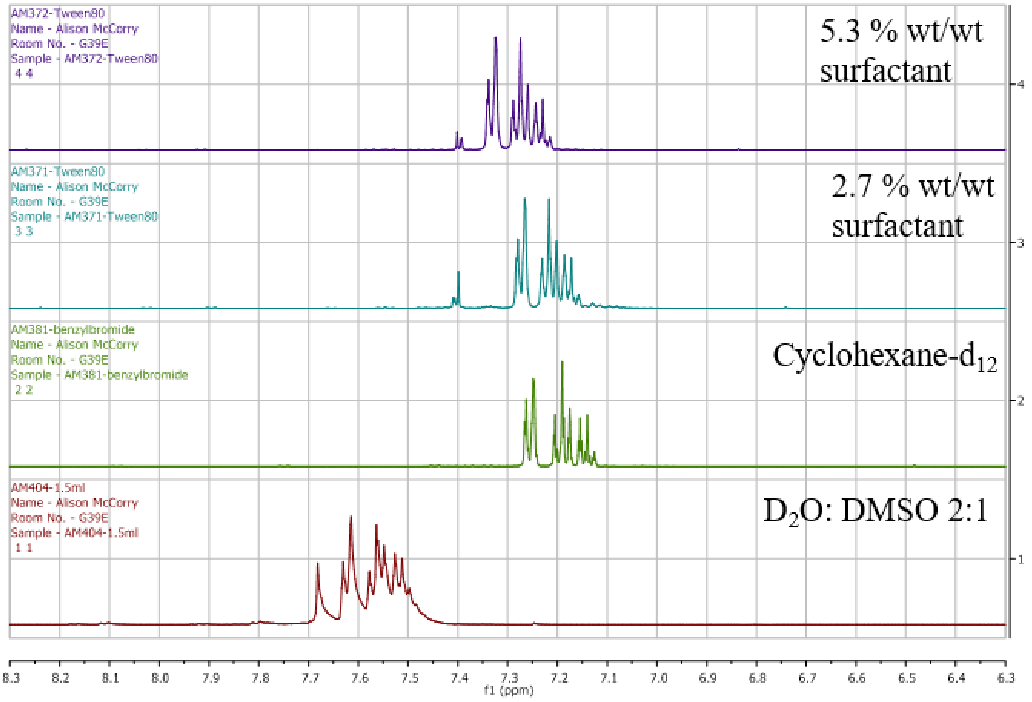

Figure S39. Spectra with Tween 80 surfactant

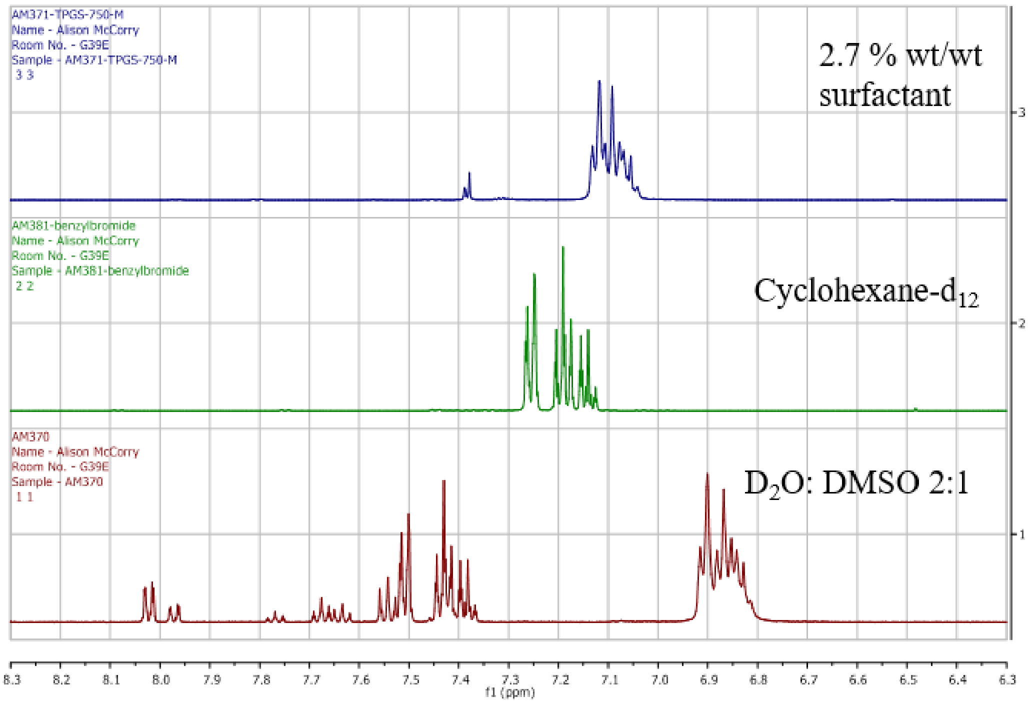

Figure S40. Spectra with TPGS-750-M surfactant

1.3.3 ^{1}H NMR spectra of the product

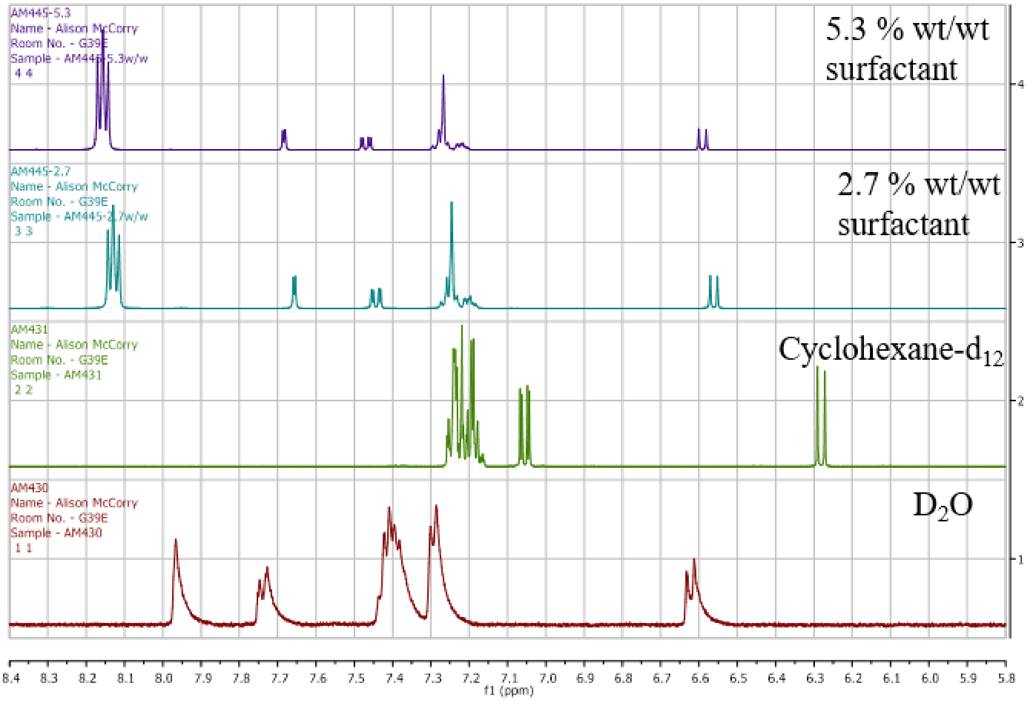

Figure S41. Spectra with CPC surfactant

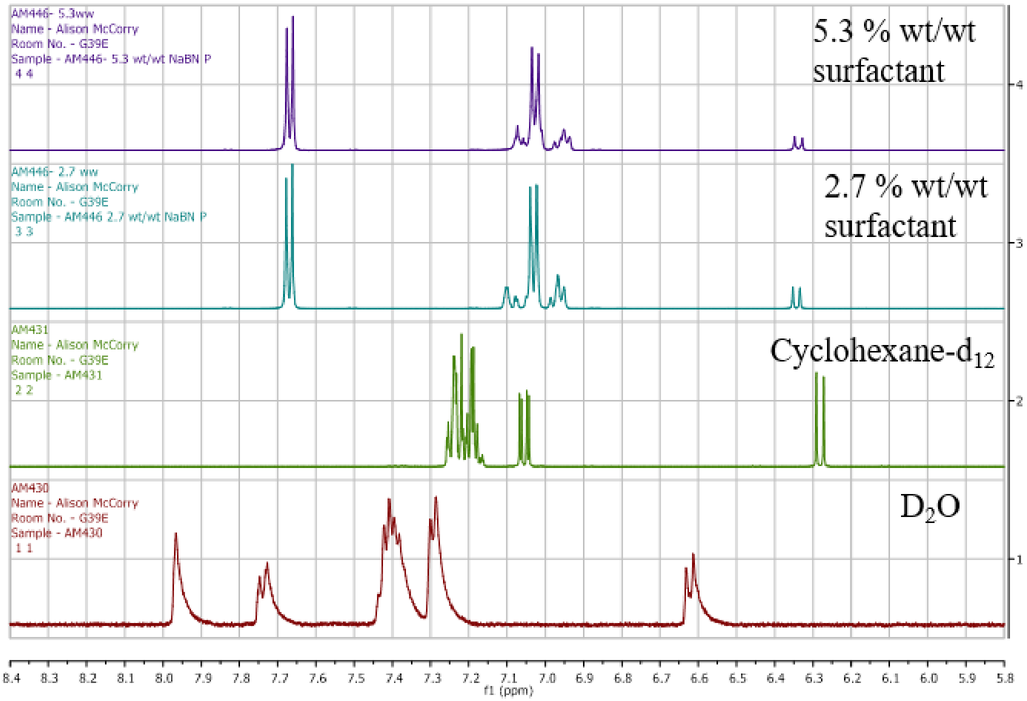

Figure S42. Spectra with SOBS surfactant

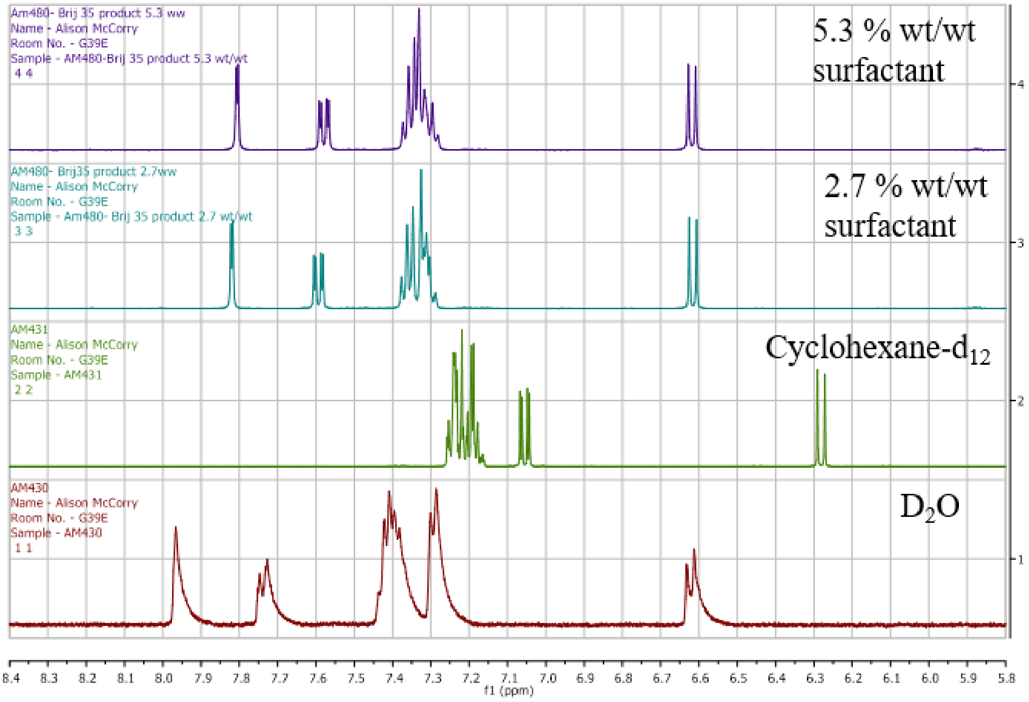

Figure S43. Spectra with Brij 35 surfactant

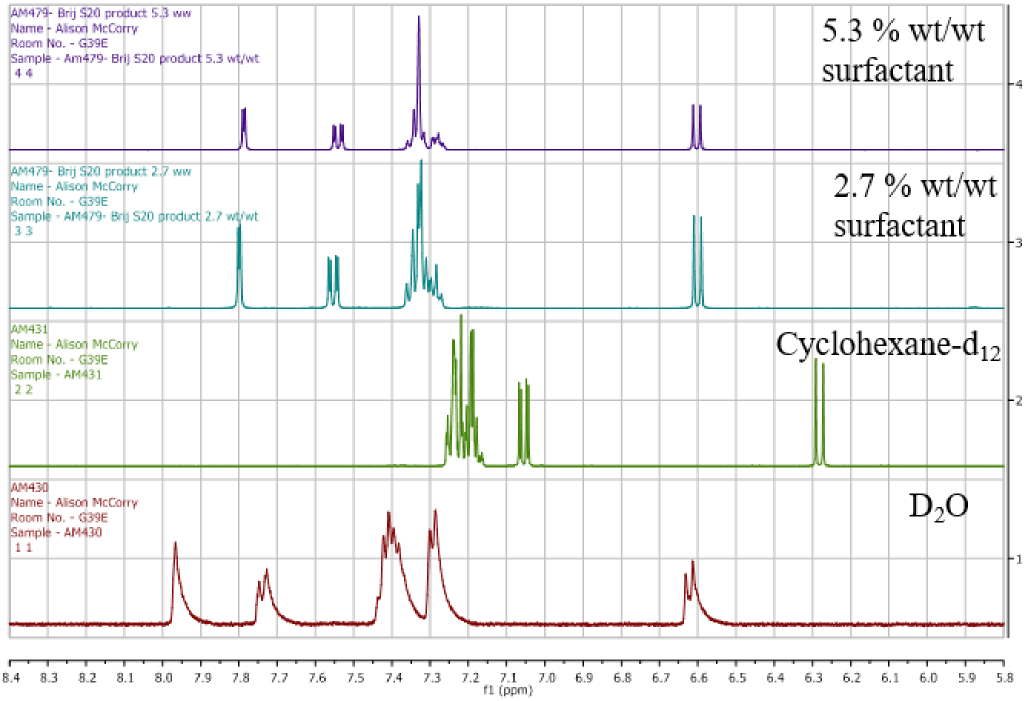

Figure S44. Spectra with Brij S20 surfactant

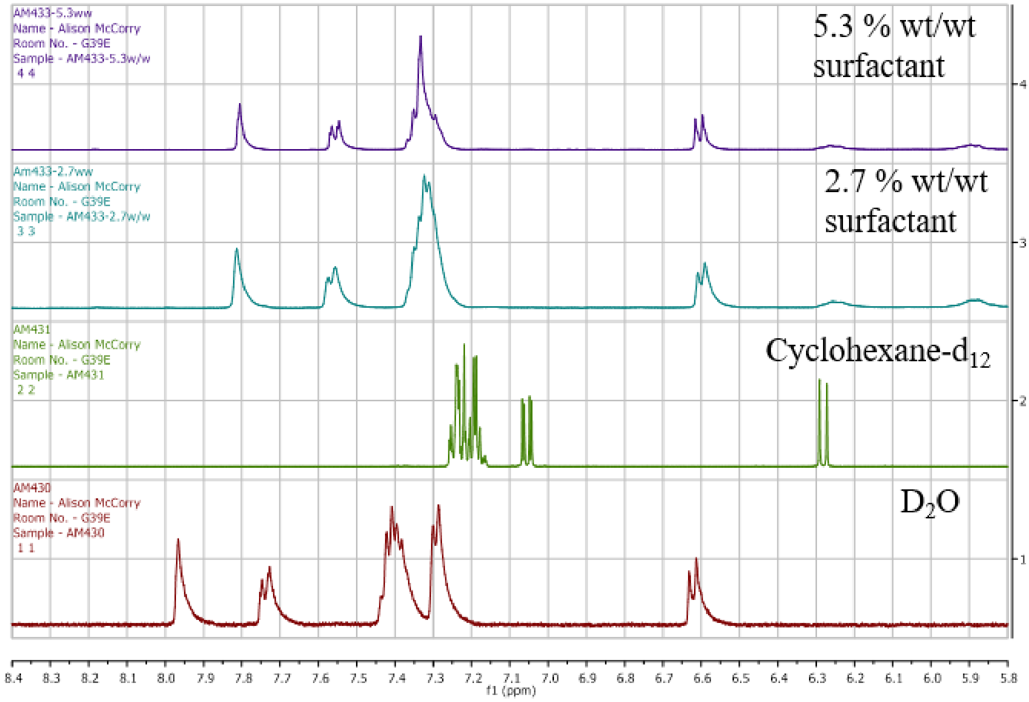

Figure S45. Spectra with Tween 80 surfactant

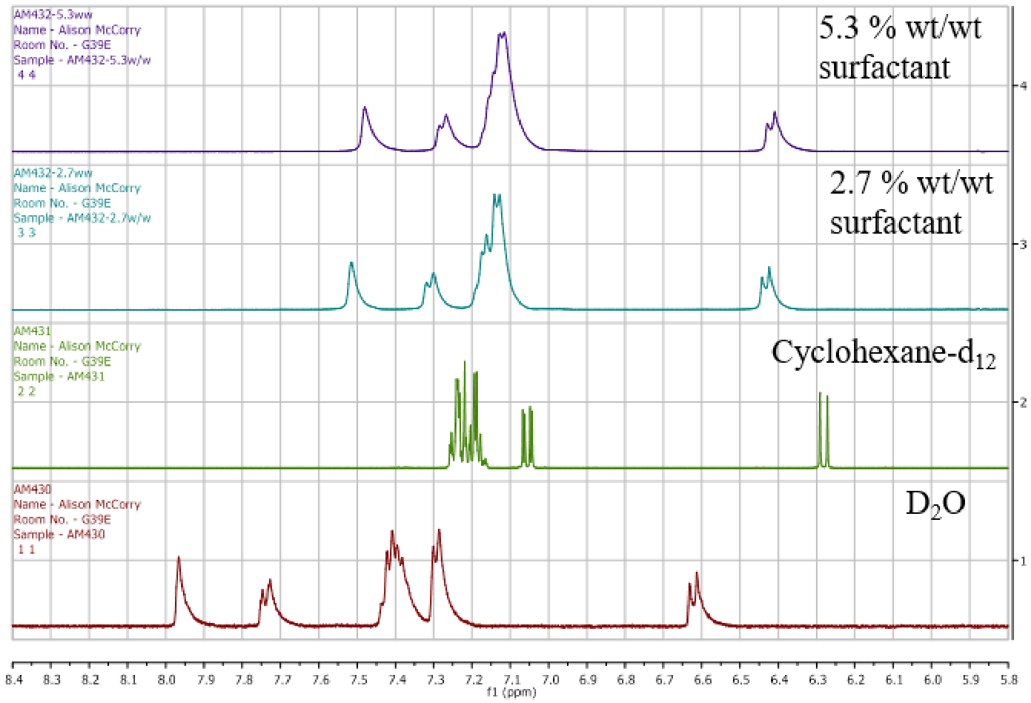

Figure S46. Spectra with TPGS-750-M surfactant

1.3.4 DOSY experiments

DOSY experiments were performed on a Bruker 500 MHz AV4 NEO 11.75 T NMR spectrometer operating at 11.75 T field and equipped with a 5mm-TXI room temperature probe with z field gradient. ^{1}H DOSY experiments were acquired at 298 K (unless otherwise stated). A DOSY sequence with stimulated-echoes with bipolar gradients was used and 8 data slices, each comprised of 32 scans were collected between a 5 – 95%

gradient strength using an exponential ramp. Parameter Δ was set to 250 ms and parameter δ set to 2 ms, resulting in an experiment time of 24 minutes.

The data was processed in Topspin for Dynamics Centre version 2.7.3, with any 1D phasing and manual peak picked performed in Topspin. Dynamics Centre was used to perform the analysis of the DOSY experiment with the 2D spectrum produced along with the sinusoidal fitting of the selected peaks.

The report then produced the tabulated data for the picked peaks from the DOSY spectra giving the tracer diffusion of each along with the error. The processing software extracts the diffusion coefficient out of the signal of decay. A 2D DOSY spectrum with diffusion coefficients along the F1 axis and chemical shifts along the F2 axis. The F1 axis after processing shows the results as a linear scale [$m^2/s \times 10^{-9}$].

Example experimental protocol: To a 15 mL vial was added TPGS-750-M (80 mg, 2.7 % w/w) and D_2O (3 mL). This was stirred for 3 hours. To the vial was added 5-bromo-2(1H)-pyridone (15 mg, 0.0862 mmol) and the mixture was stirred using a slide round PTFE coated magnetic flea 15 x 4.5mm for a further hour at rt and 700 RPM. The solution was clear. A small sample was removed from the vial and transferred to an NMR tube.

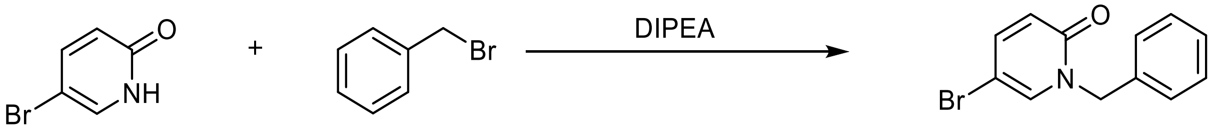

Results for 5-bromo-2(1H)-pyridone

In D_2O

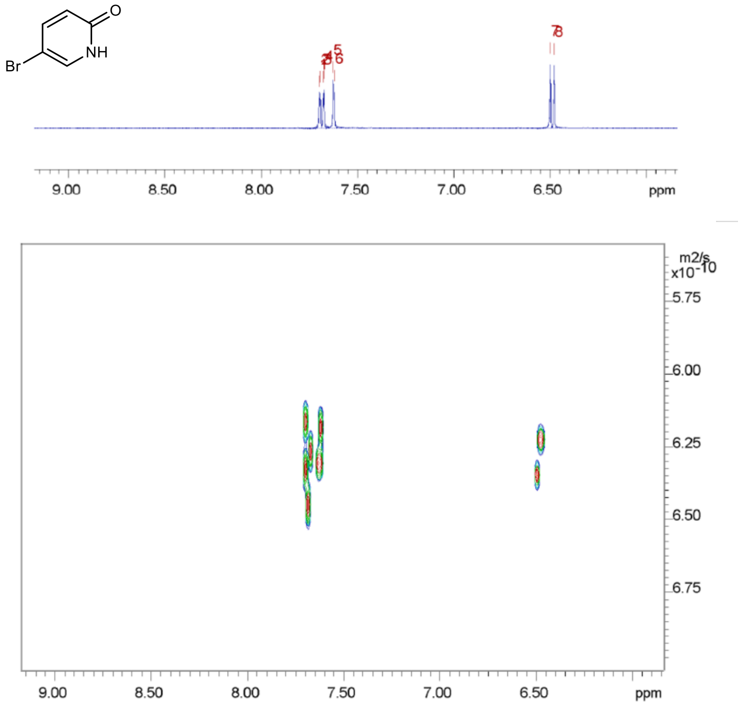

D = 6.28 × 10^{-10} for proton at 7.67 ppm

In D_2O and TPGS-750-M 2.7 % w/w

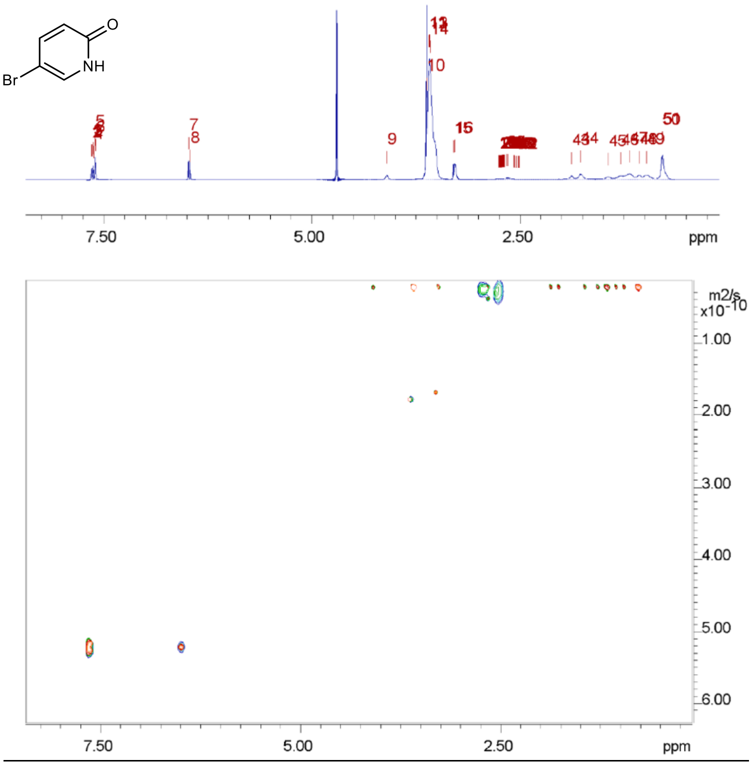

D = 5.19 × 10^{-10} for proton at 7.65 ppm pyridone peak

D= 2.38 × 10^{-11} for proton at 1.89 ppm TPGS-750-M peak

In D_2O and TPGS-750-M 5.3 % w/w

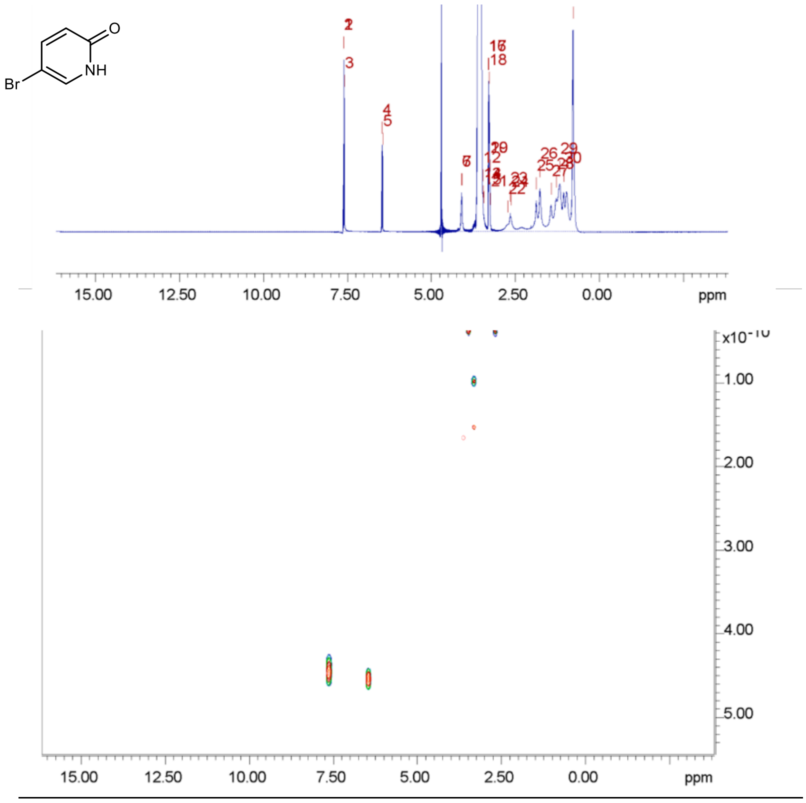

D = 4.41 × 10^{-10} for proton at 7.62 ppm pyridone peak

D= 1.67 × 10^{-11} for proton at 1.88 ppm TPGS-750-M peak

Results for benzyl bromide

<u>In D_2O</u>

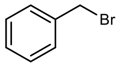

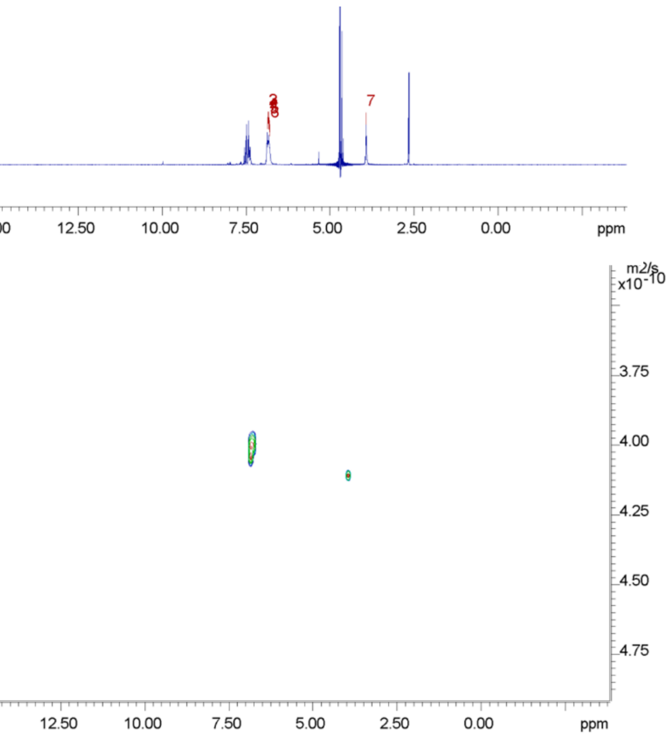

D = 4.00 × 10^{-10} for proton at 6.87 ppm benzyl bromide peak

In D_2O and TPGS-750-M 2.7 % w/w

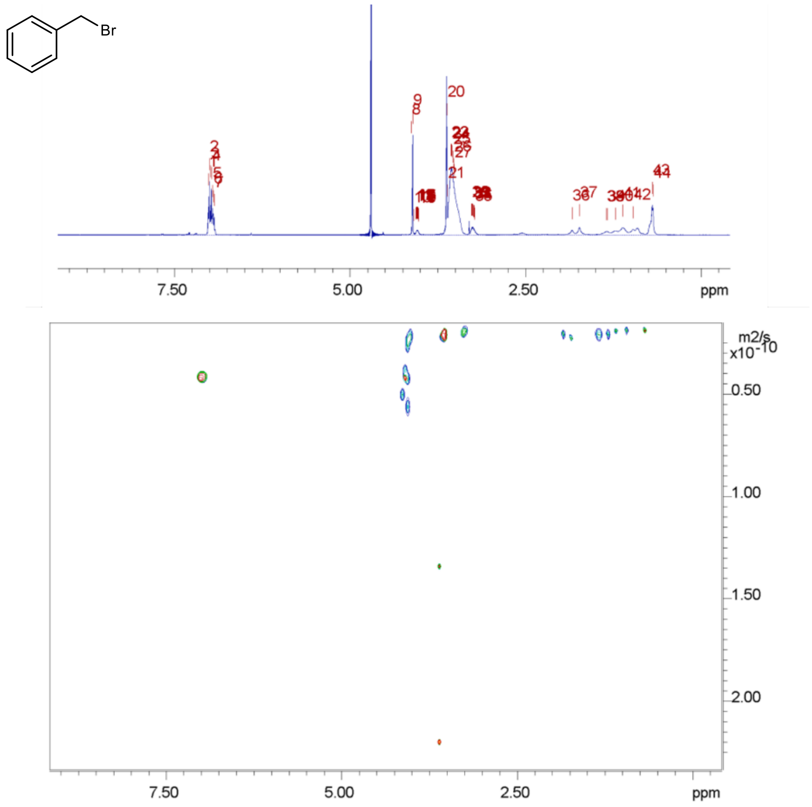

D = 4.24 × 10^{-11} for proton at 6.94 ppm benzyl bromide

D= 2.17 × 10^{-11} for proton at 1.86 ppm TPGS-750-M peak

Results for 1-benzyl-5-bromo-2(1H)-pyridone

In D_2O

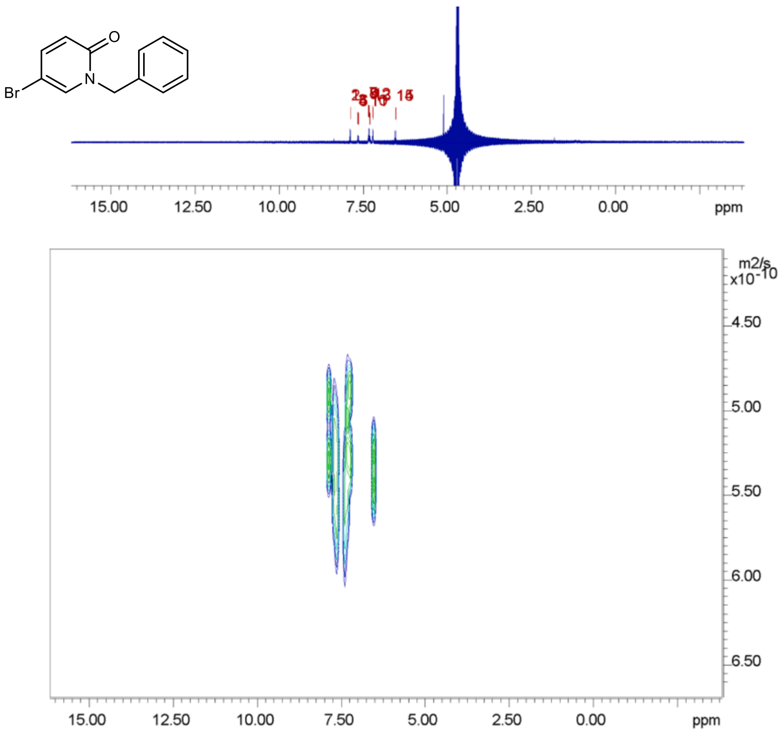

D = 4.93 × 10^{-10} for proton at 7.89 ppm 1-benzyl-5-bromo-2(1H)-pyridone

In D_2O and TPGS-750-M 2.7 % w/w

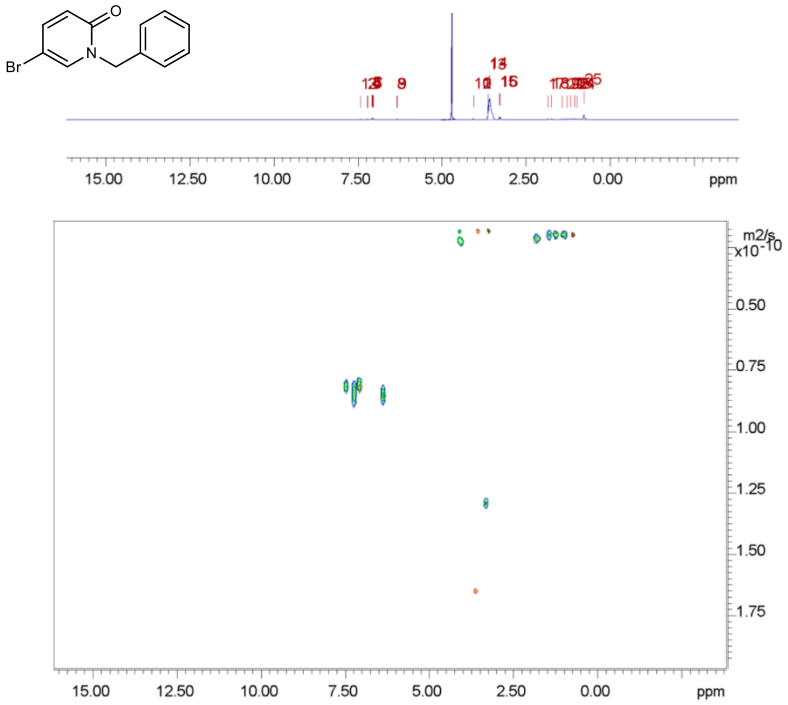

D = 8.19 × 10^{-11} for proton at 7.43 ppm 1-benzyl-5-bromo-2(1H)-pyridone

D= 2.17 × 10^{-11} for proton at 1.86 ppm TPGS-750-M peak

<u>In D_2O and TPGS-750-M 5.3 % w/w</u>

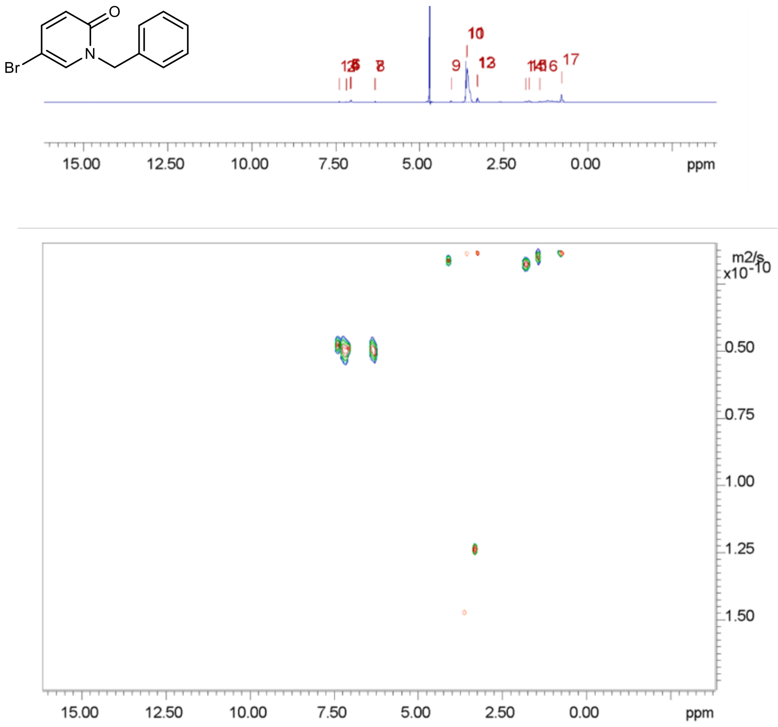

D = 4.82 × 10^{-11} for proton at 7.40 ppm 1-benzyl-5-bromo-2(1H)-pyridone

D= 1.78 × 10^{-11} for proton at 1.86 ppm TPGS-750-M peak

2 Surfactant map development

Table S12. Descriptors for ***surfactant_map***

**Descriptor**	**Classification**	**Representing**	**Source**
Critical micelle concentration (CMC)	Micellar property	Surfactant-surfactant interactions	Literature
Aggregation number range	Micellar property	Surfactant-surfactant interactions	Literature
Micelle size range	Micellar property	Surfactant-surfactant interactions, homogeneity	Literature
Contact angles	Emulsion property	Surface tension and wettability	Experimental
Zeta potential	Emulsion/micellar property	Charge environment around micelles, emulsions	Experimental
Hydrophilic Lipophilic Balance (HLB)	Emulsion property	Emulsion stability	Literature
Hydrophilic fragment rotatable bonds	Molecular property	Flexibility of surfactant molecules and emulsion flexibility/stability	*rdkit*
Hydrophobic fragment rotatable bonds	Molecular property	Flexibility of surfactant molecules and emulsion flexibility/stability	*rdkit*
Hydrophilic fragment longest chain length	Molecular property	Size of the interface layer between organic and aqueous phases	*rdkit*
Hydrophobic fragment longest chain length	Molecular property	Capability for stabilising organic phase inside emulsions	*rdkit*
Hydrophilic fragment volume	Molecular property	Packing of surfactant molecules and stability of emulsion	*rdkit*
Hydrophobic fragment volume	Molecular property	Packing of surfactant molecules and stability of emulsion	*rdkit*
Hydrophilic fragment surface area	Molecular property	Packing of surfactant molecules and stability of emulsion	*rdkit*
Hydrophobic fragment surface area	Molecular property	Packing of surfactant molecules and stability of emulsion	*rdkit*
Hydrophobic fragment number of C=C bonds	Molecular property	Flexibility of surfactant molecules and emulsion flexibility/stability	*rdkit*
Hydrophilic fragment number of OH groups	Molecular property	Capability of H-bonding at the interface of organic and aqueous phases	*rdkit*
Hydrophilic fragment ΔG_{solv}	Molecular property	Stability of emulsion	Gaussian, PM6

Hydrophilic fragment dipole moment	Molecular property	Stability of emulsion	Gaussian, PM6
Hydrophilic fragment HOMO energy	Molecular property	H-bonding capability and interactions with transition states	Gaussian, PM6
Hydrophilic fragment LUMO energy	Molecular property	H-bonding capability and interactions with transition states	Gaussian, PM6
Hydrophobic fragment dipole moment	Molecular property	Stability of emulsion	Gaussian, PM6
Hirshfeld charge for most negative heteroatom	Molecular property	Interactions with transition states	Gaussian, PM6, multiwfn

2.1 Literature descriptors

2.1.1 Critical micelle concentration (CMC)

The CMC is the minimum concentration of surfactant in water required for surfactants to self-assemble and form micelles. When micelles are formed, surfactant monomers no longer gather at the interface but form micelle aggregates. At the CMC surfactants can no longer influence the interfacial properties. Unsurprisingly literature regarding this is the most accessible, with higher CMCs observed for ionic surfactants than non-ionic surfactants. Additionally, from literature values gathered, CMCs are generally observed to decrease with an increase in hydrophobic chain length. CMC values of surfactants were collected from various literature resources where available, and all converted to mM units.

2.1.2 Aggregation number

Aggregation number (N) is the average number of surfactant monomers that form a micelle. Aggregation numbers of surfactants were collected from various literature resources; however this descriptor is dependent upon experimental conditions which poses issues in direct comparison of aggregation numbers. Therefore, aggregation numbers were collected where available under different conditions and a “low” and a “high” aggregation number were included as separate descriptors. Aggregation numbers collected so far cover a broad range from 2 to 16600 surfactant monomers per micelle.

2.1.3 Micelle Sizes

Size of micelles can change in accordance with the concentration used, salt concentration and the characterisation method. Size of the surfactant micelles has been measured using various techniques; light scattering techniques (DLS and SLS),[1–3] SANS and SAXS,[4,5] AFM,[6] Cryogenic-transmission electron microscopy (Cryo-TEM)[7] and TDA.[8] The micelle size is a heavily studied characteristic and it is important to assess how influential it is in regards to micellar catalysis. Similarly, to aggregation number, the size of the micelle is affected by experimental conditions so direct comparison of literature values is difficult. Therefore,

micelle size was collected where available under different conditions and a “low” and a “high” micelle size were included as separate descriptors. The size of micelles in this were expressed by their diameter and values were collected from literature. Diameter sizes in literature showed a distribution of 0.6 - 237 nm.

2.2 Experimental descriptors

2.2.1 Contact angle measurements

Hydrophilic surfactants, which are preferentially wetted by water and will stabilise oil in water emulsions will give a contact angle of θ > 90°. Hydrophobic surfactants, which are preferentially wetted by water and will stabilise oil in water emulsions will give a contact angle of θ < 90°.[9]

Static contact angles were measured using a goniometer KSV CAM 200 optical contact angle meter (KSV instruments, Ltd), using a sessile drop method with a telescope. The CAM software measures the surface and interfacial tension and then performs curve fitting image analysis to determine contact angle.

A vial was charged with surfactant (2 % w/w), deionised water (5 mL) and a slide round magnetic flea (15 x 4.5mm). This was stirred overnight at room temperature at 1000 RPM. For some surfactants, the solution required heating to completely dissolve the surfactant. Contact angles were repeated a further 9 times allowing a mean and standard deviation to be taken. The measurements were performed at ambient temperature and pressure. Contact angles were measured using microscope glass slides 1.0mm thickness, 76 x 26mm (Length x Width).

2.2.2 Zeta (ζ) potential measurements

The ζ–potential has many important applications across engineering, chemistry, and other sciences; therefore this had led to the development of several techniques to measure this. The techniques to measure ζ–potential are based on either electrophoresis, electroosmosis or the streaming potential.[10] The zeta potential has been used to evaluate the stability of nanoparticles in addition with further techniques, however this is not an absolute measurement of nanoparticle stability.[11,12]

All zeta potential measurements reported in this paper were measured using Zetasizer® Nano Range Analyzer (Nano-ZSP, Malvern Panalytical Instruments, UK) fitted with a universal drip cell (ZEN1002). The ζ–potential was obtained by measuring the electrophoretic mobility of particles in a solution and introducing an electric field. The ζ–potential can be derived from the electrophoretic mobility of particles in a solution using the Smoluchowski equation.

$$\upsilon_E = 4\pi\varepsilon_0\varepsilon_r \frac{\zeta}{6\pi\mu} (1 + \kappa r) \quad (2)$$

In this equation, υ_E is the electrophoretic mobility, ε_0 and ε_r are the relative dielectric constant and the electrical permittivity of a vacuum, ζ is the zeta potential, μ is the solution viscosity, κ is the Debye-Hückel parameter and r is the particle radius.[13]

A vial was charged with surfactant (0.1% w/w), deionised water (5 mL) and a slide round magnetic flea (15 x 4.5 mm). This was stirred overnight at room temperature at 1000 RPM. For some surfactants the solution required heating to completely dissolve the surfactant (never above 50 °C). ~ 0.8mL aliquots were removed from the stirring vials and transferred into the dip cell using a 1 mL syringe. An equilibrium period of 120 seconds was used. Measurements were performed in triplicate, with at least 3 different aliquots measured. All measurements were performed at ambient temperature and pressure.

2.3 Computational descriptors

2.3.1 Volume of hydrophilic and hydrophobic regions of surfactant

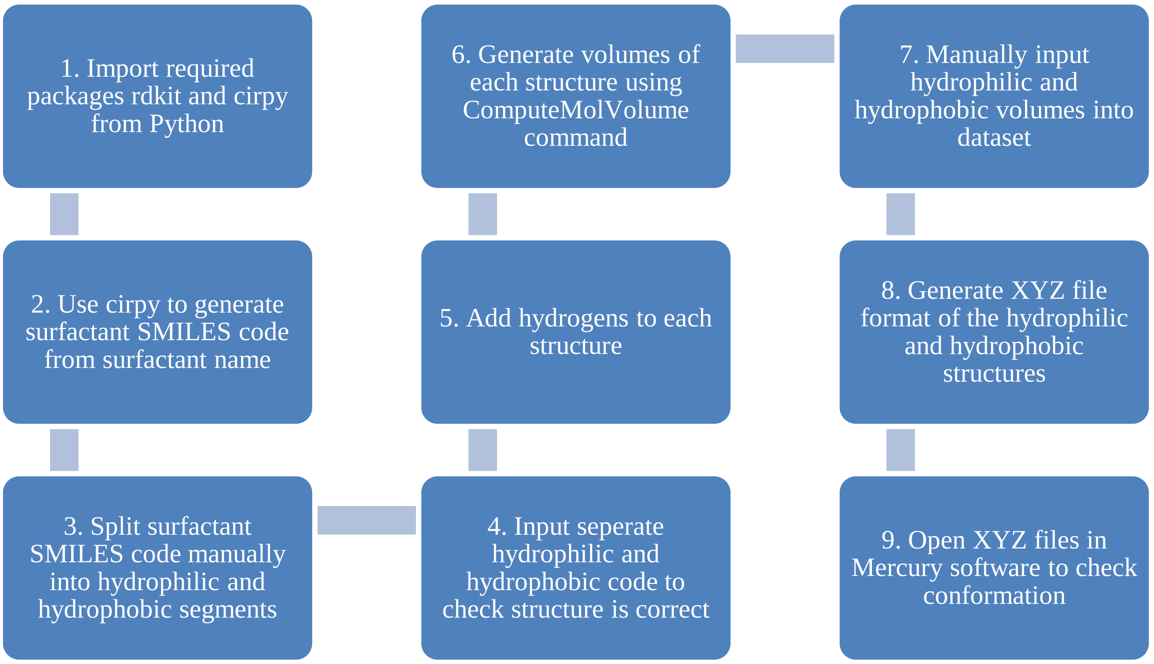

Figure S47. Flow chart to calculate hydrophilic and hydrophobic volume descriptors

The volumes of hydrophobic and hydrophilic fragments, manually split from the surfactant's chemical structure (Figure S48), of each surfactants was calculated computationally using the *rdkit* package in Python.[14] *Cirpy* package was used to convert a chemical identifiers/names to SMILES strings for *rdkit*.[15] When the surfactant name was not recognised by the *cirpy* package, the SMILES string was manually generated from the chemical structure of the surfactant.

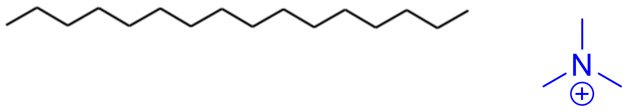

Figure S48. Generation of the surfactant hydrophobic and hydrophilic chemical structures of CTAB from the SMILES code

Hydrogen atoms were added to the generated structure of each fragment and the volume generated with *rdkit*. The hydrophilic head of CTAB generated a volume of 74.5 Å^3 and the hydrophobic tail gave 278.7 Å^3. Comparing this to Brij 52 a volume of 104.9 Å^3 and the hydrophobic tail gave 278.7 Å^3. Brij 52 has a structure represented by the shortened name C16E2, which corresponds to a linear surfactant with a carbon chain length of 16 (C16) in the hydrophobic tail, and 2 PEG groups (E2) as the hydrophilic head section. As expected, the hydrophobic tails generated the same volumes as they both have a 16-carbon chain. No conformation exploration was performed at this stage. The volume command used in *rdkit* then calculates the volume of a particular conformer of the chemical structure based on a grid-encoding of the molecular shape.[16] The 3D coordinates for each surfactant fragments were exported in *.xyz* format for the next step.

2.3.2 Area of hydrophilic and hydrophobic regions of surfactant

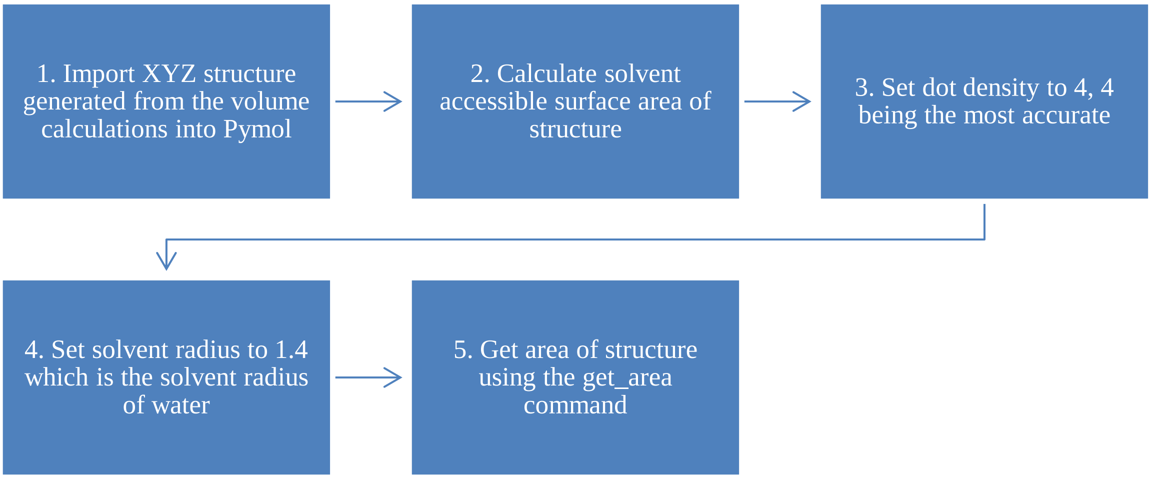

Figure S49. Flow chart of process to calculate the hydrophilic and hydrophobic areas of surfactant molecules.

For surface area measurements, the *.xyz* file of each fragment was imported into PyMOL (Figure S50).[17] The *get_area* measurements were generated using a solvent dot density of 4 and solvent radius of 1.4 Å (for water).

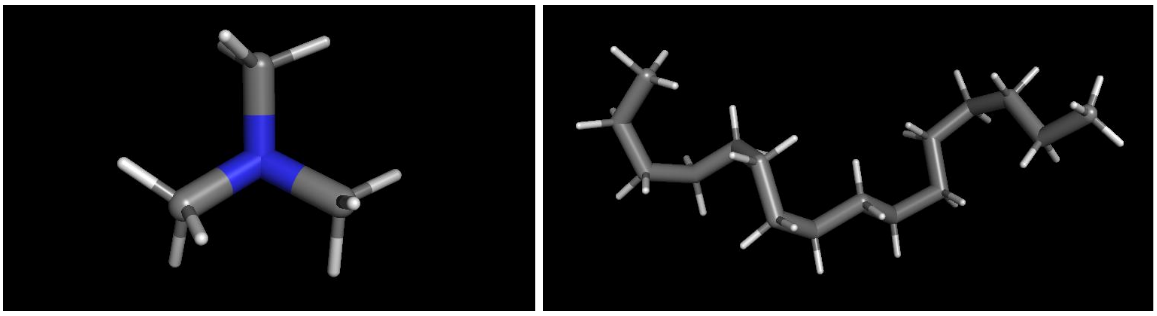

Figure S50. PyMOL imported XYZ. structure of the CTAB hydrophilic and hydrophobic regions

Comparing the same surfactants as in the volume calculations, CTAB generated a hydrophilic area of 232.4 Å^2 and a hydrophobic area of 560.5 Å^2. The non-ionic straight chain surfactant Brij 52 generated a hydrophilic area of 293.7 Å^2 and a hydrophobic tail area of 560.5 Å^2. The hydrophobic areas calculated were the same, since both contain a 16-carbon chain. Coiling of PEG chains (Figure S51) was observed with the majority of PEG-based surfactants.[18–20]

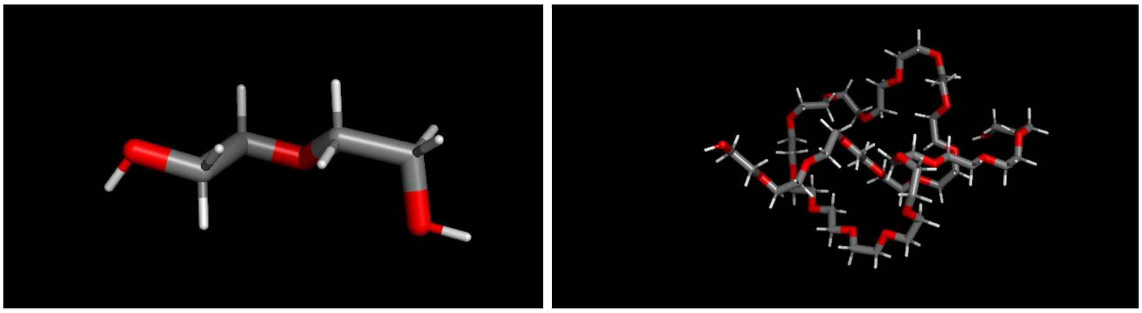

Figure S51. (LHS) Structure of Brij 52 head group having 2 PEG groups. (RHS) Head group of Brij 721 containing 21 PEG groups.

2.3.3 Rotatable bonds

The number of rotatable bonds reflects the molecular flexibility of the molecule.[21] This descriptor was generated using a built-in function of the *rdkit* package.

2.3.4 Longest chain length

The length and shape of surfactants are important physiochemical properties to be aware of. The carbon chain length has been investigated with regards to surfactant based lipsomomes.[22] It was proposed that carbon chain length could have an effect on vesicle rigidity and vesicle size. This descriptor was generated using a built-in function of the *rdkit* package.

2.3.5 Number of C=C double bonds (hydrophobic fragment)

The number of double bonds in the hydrophobic fragment of each surfactant was manually calculated from saved SMILES codes for each surfactant. Surfactants in the dataset were found to possess either 0 or 1 double bonds. All the surfactants in the dataset which contain a double bond are synthesised from oleic acid which is a naturally occurring fatty acid which is found to have predominantly *cis* stereochemistry (18:1 *cis* to *trans*).

2.3.6 Free OH groups (hydrophilic)

The number of hydroxy moieties in the hydrophilic fragment was manually calculated from the SMILES strings of each surfactant. The number of OH bonds ranged from 0 to 3 in the dataset.

2.3.7 Electronic structure calculation details

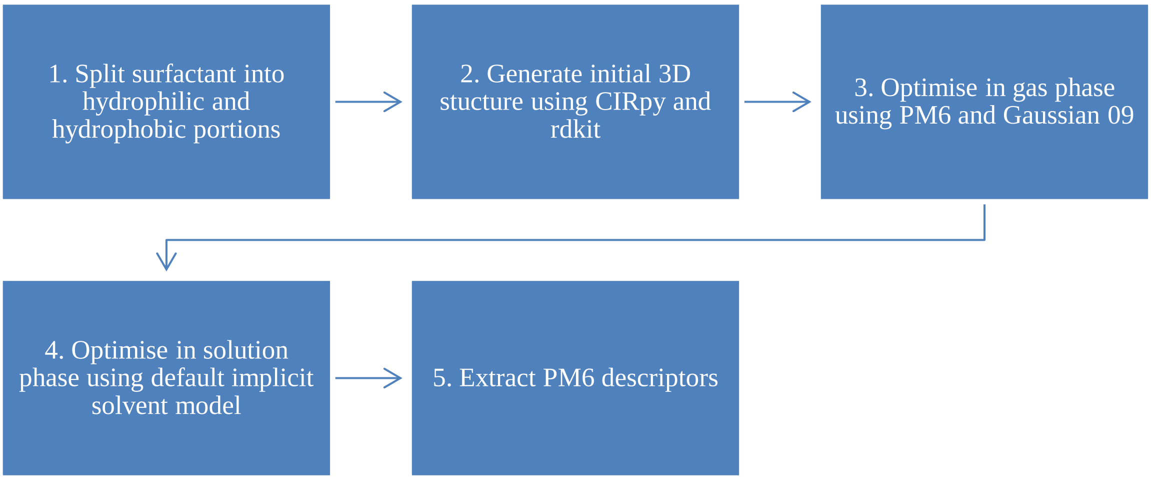

Figure S52. Flow chart of process using to calculate the six PM6 descriptors used in this work

Three-dimensional descriptors based on electronic structure methods are common in chemistry to represent molecules and their physical properties more accurately, with Gibbs energies, point charges, HOMO/LUMO, and dipole as standard values to calculate.[23] To calculate the six PM6 descriptors, surfactants were split into hydrophilic and hydrophobic end as previously described. Initial 3D structures were generated with *cirpy* and *rdkit*. These structures were optimised using PM6,[24] a good trade-off between speed and accuracy for the size of surfactants under study, and Gaussian 09 software.[25] The size of these surfactants meant that exhaustive comformational search was not possible and the xyz structure generated by *rdkit* was used directly as input for PM6 optimisation. Structures were optimised in both gas and solution using the default PCM solvent model.[26] A small number of hydrophilic molecules which contained repeating PEG units could not be optimised due to their size and flexibility, so the descriptors for the longest repeating PEG unit that did optimise was used in these cases. For solution 17 units was used for any molecule > 17 units and for gas 50 units was used for any molecule > 50 units.

From these calculations, the difference between solution and gas Gibbs energies for hydrophilic end (*Philic_DeltaG_sol*, Ha), dipole of solution structure for hydrophilic fragment (*Philic_Solv_dip*, Debye), HOMO energy of gas phase structure for hydrophilic fragment (*Philic_HOMO*, eV), and LUMO energy of gas phase structure for hydrophilic fragment (*Philic_LUMO*, eV) were extracted.

The Hirshfeld charge for most negative heteroatom, relevant in estimating nucleophilic and electrophilic reactivity,[27] for hydrophilic fragment (*Philic_Most_neg*, a.u.) was generated using Multiwfn software,[28] via single point calculations in Gaussian 09. Optimised gas phase structures were rerun with the keyword **out=wfn** in the ground state and the first excited state. Transfer to Multiwfn gave the Hirshfeld charge for each atom

and hence the condensed Fukui function for each atom (difference between the Hirshfeld charge in the ground and first excited state).

The dipole moment of the solution structure of the hydrophobic fragment (*Phobic_Solv_dip*, Debye) was extracted directly from the Gaussian output file.

2.4 List of surfactants

Table S13. Full list of surfactants in this study

No.	Name	Structure
1	Polysorbate 20	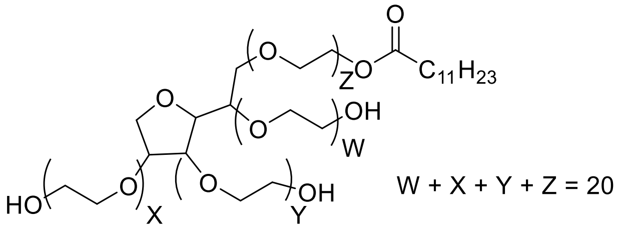
2	Polysorbate 40	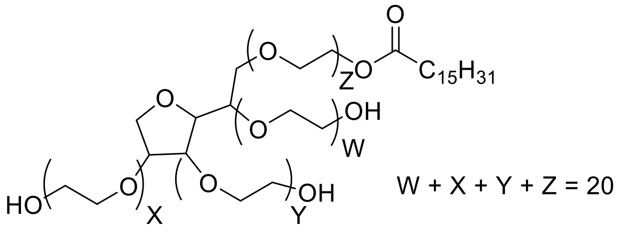
3	Polysorbate 60	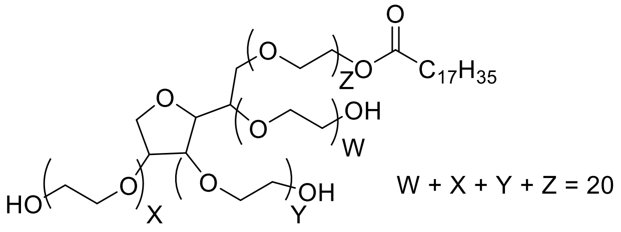
4	Polysorbate 80	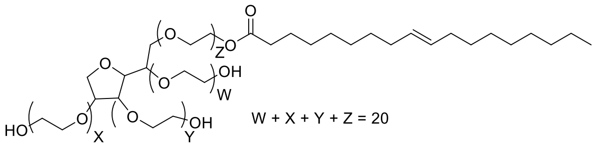
5	Tween 65	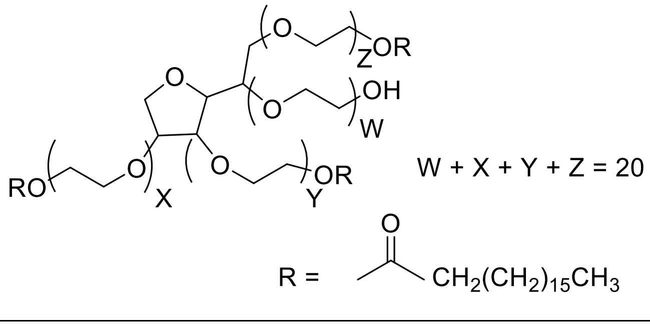

6	Tween 85	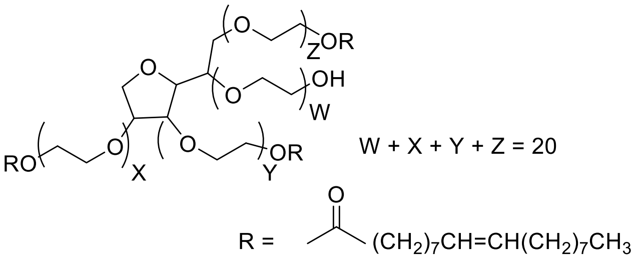
7	Span 20	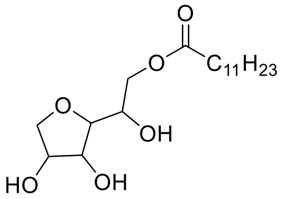
8	Span 40	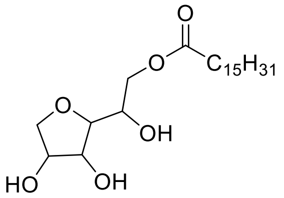
9	Span 60	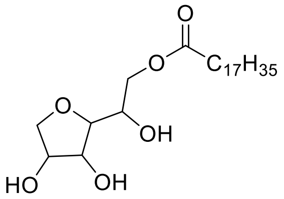
10	Span 80	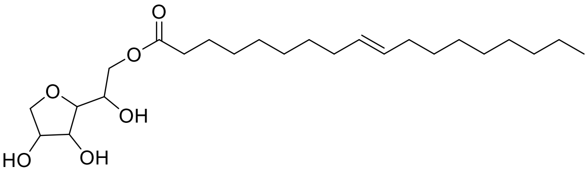
11	Span 65	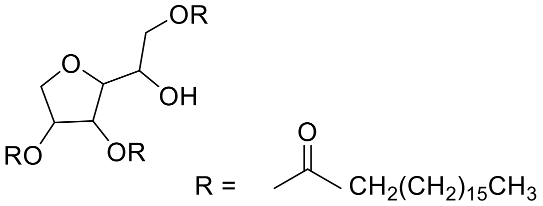
12	Span 85	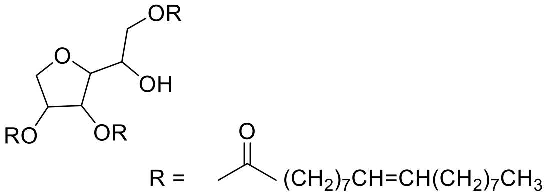
13	C8E6	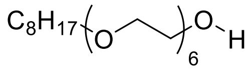
14	Octaethylene glycol monodecyl	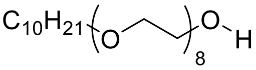
15	Brij 30	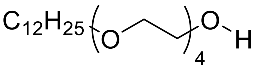

16	C12E5	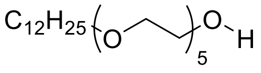
17	C12E6	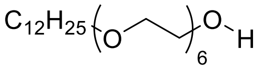
18	Brij 35	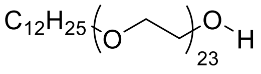
19	Octaethylene glycol monotetradecyl ether	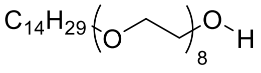
20	SP Brij C2 MBAL/Brij 52	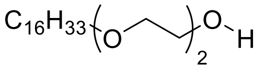
21	C16E8	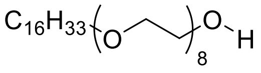
22	Brij 56	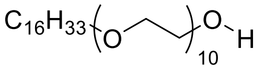
23	Brij 58	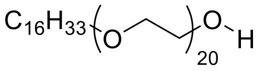
24	Brij 72	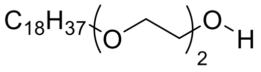
25	C18E8	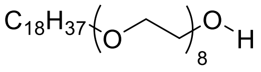
26	Brij S-10	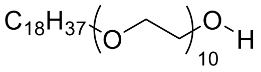
27	Brij 93	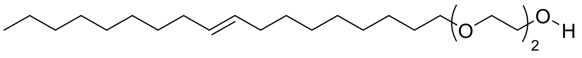
28	Brij O10	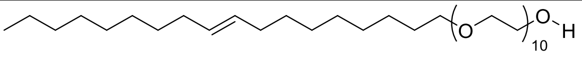
29	Brij 99	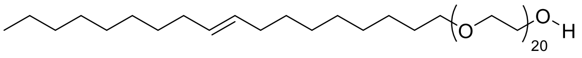
30	Brij S20	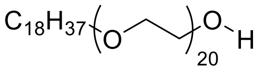
31	Brij 721	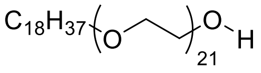
32	Brij 700	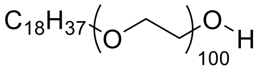
33	Triton-X-15	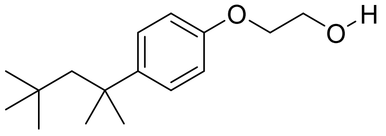
34	Triton-X-35	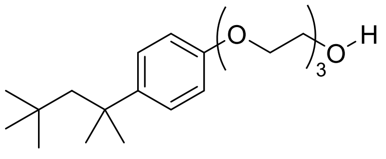

35	Triton-X-45	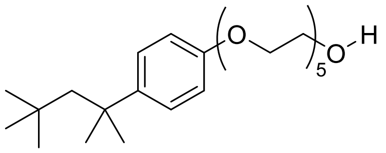
36	Triton-X-100	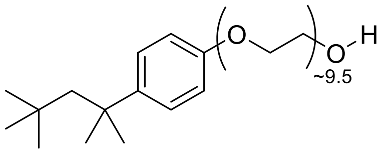
37	Triton-X-102	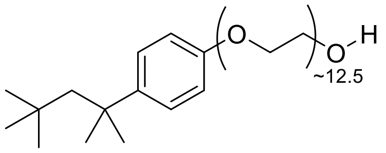
38	Triton-X-114	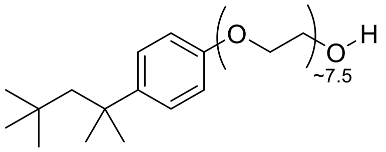
39	Triton-X-165	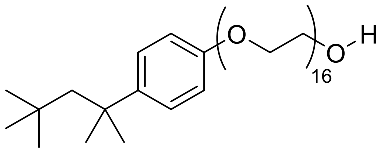
40	Triton-X-305 (	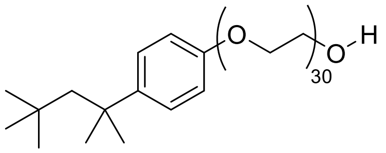
41	Triton-X-405	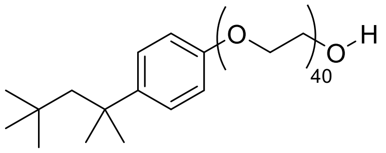
42	Triton-X-705	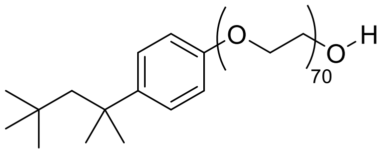
43	IGEPAL CA720	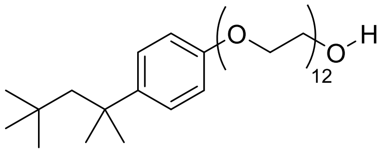
44	IGEPAL CO520	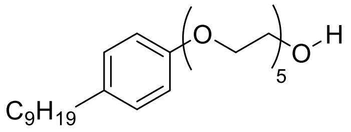
45	IGEPAL CO630	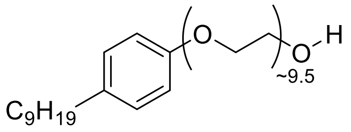

46	IGEPAL CO720	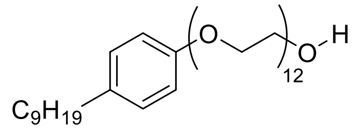
47	Myrj S8	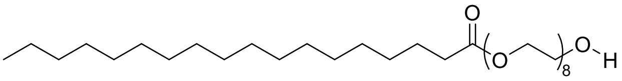
48	Myrj S20	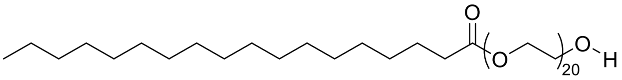
49	Myrj 52	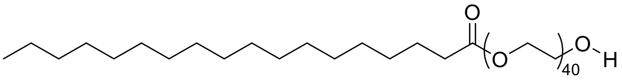
50	Myrj 53	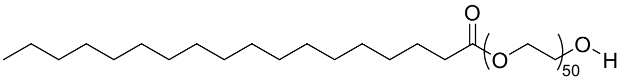
51	Myrj 59	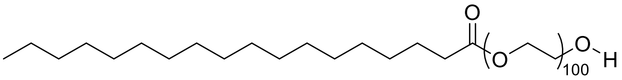
52	DDM (N-Dodecyl β-D-maltoside)	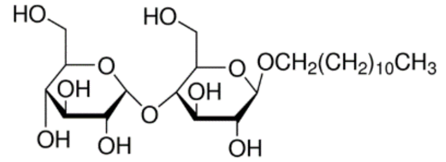
53	Nonyl β-D-glucopyranoside	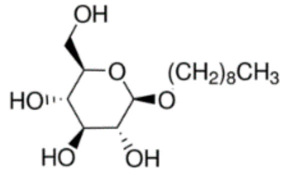
54	Sucrose monolaurate	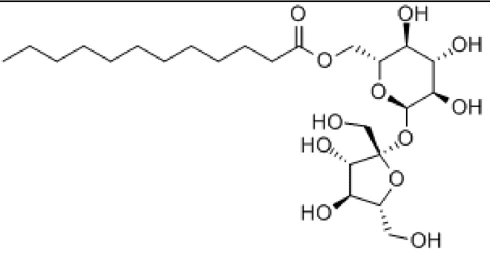
55	Sucrose Distearate	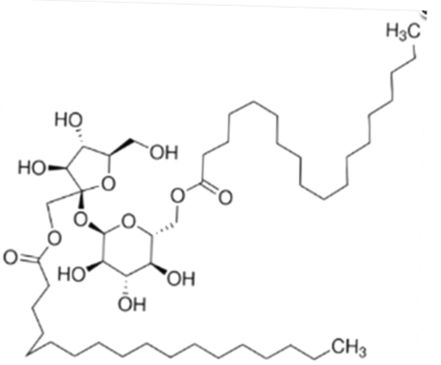
56	CTAB	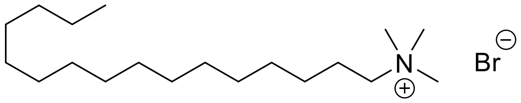

57	CTAC	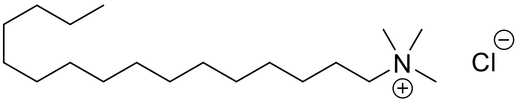
58	DTAB	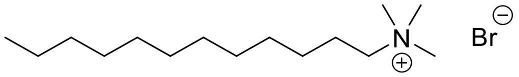
59	DTAC	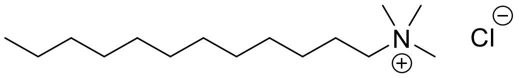
60	TTAC	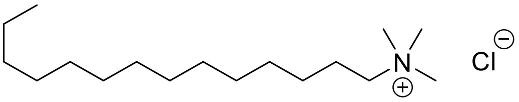
61	TTAB	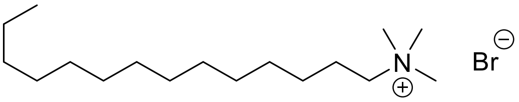
62	CPC ((1-Hexdecyl)pyridinium chloride monohydrate)	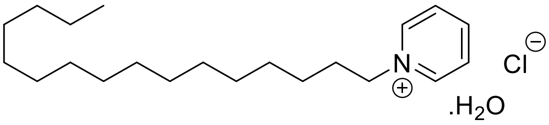
63	DDAB (Di-n-dodecyl)dimethyl ammonium bromide)	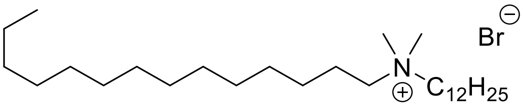
64	CHAPS (3-((3-cholamidopropyl) dimethylammonio)-1-propanesulfonate)	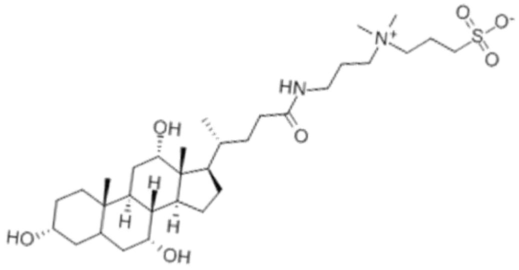
65	DAPS (N-dodecyl-N,N-dimethyl-3-ammonium-1-propane sulfonate)	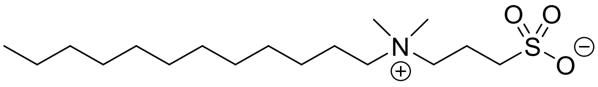
66	3-(N,N-Dimethylmyristylammonio)propanesulfonate	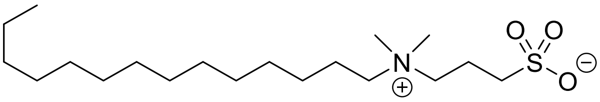
67	Sulfobetaine-16	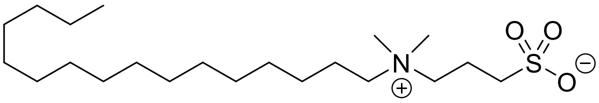
68	Lauryldimethylammonio)acetate	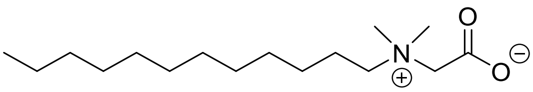
69	TPGS-750-M	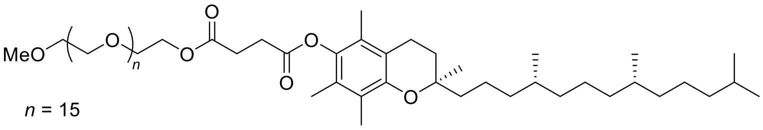
70	TPGS-1000 (DL-alpha-Tocopherolmethoxypolyethyleneglycol 1000 succinate)	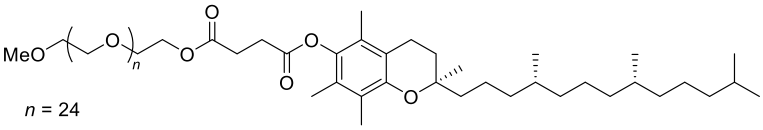

71	SPGS-550-M	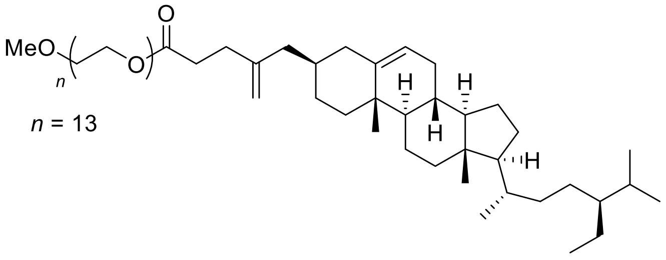
72	PSS	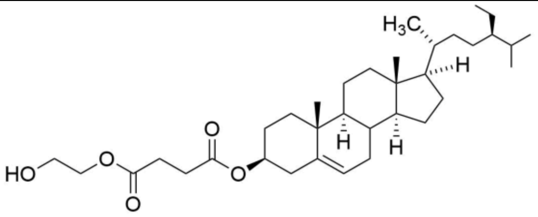
73	PTS	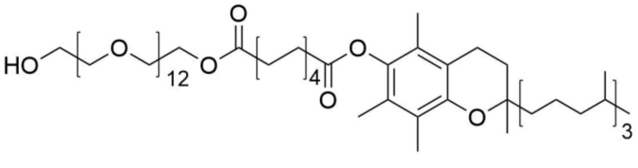
74	Coolade	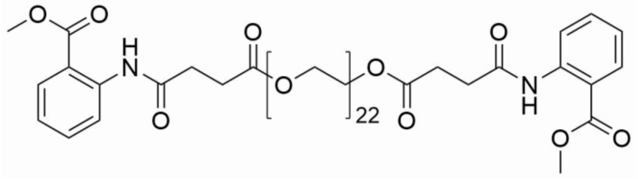
75	PS-750-M	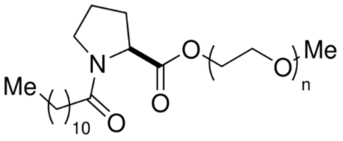
76	Sodium Octanoate/Sodium caprylate	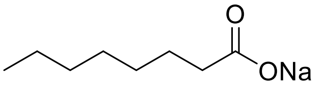
77	Sodium decanoate	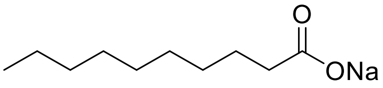
78	Sodium Laurate	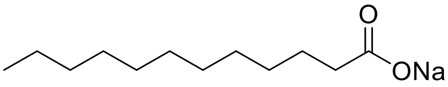
79	Palmitic acid sodium salt	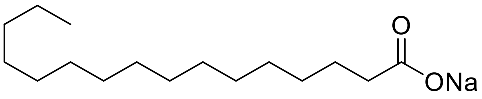
80	Sodium Stearate	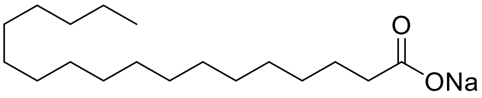
81	Sodium Oleate	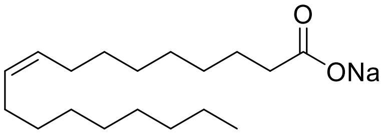
82	1-Decanesulfonic acid, sodium salt	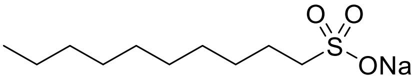

83	1-Dodecanesulfonic acid, sodium salt	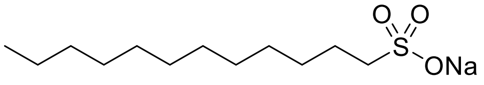
84	Sodium 1-tetradecanesulfonate	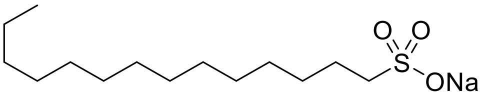
85	Sodium 1-hexadecanesulfonate	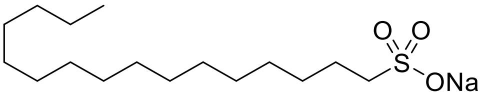
86	n-Decyl sodium sulfate	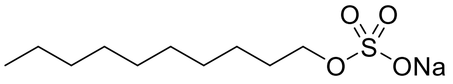
87	SDS	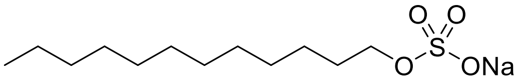
88	Sodium-1-tetradecyl sulfate	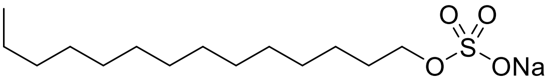
89	Sodium Hexadecyl sulfate	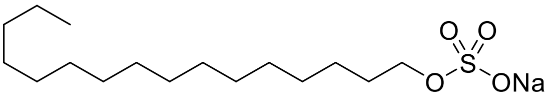
90	Sodium n-octadecy sulfate	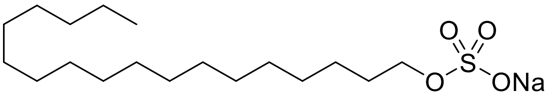
91	Sodium 4-n-octylbenzenesulfonate	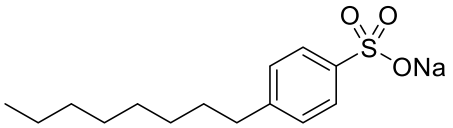
92	SDBS	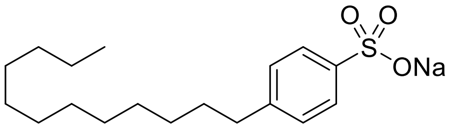
93	4-Dodecylbenzene sulfonic acid	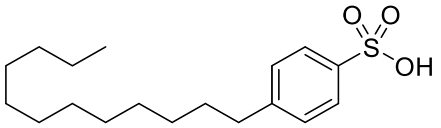
94	Dioctyl sulfosuccinate sodium salt	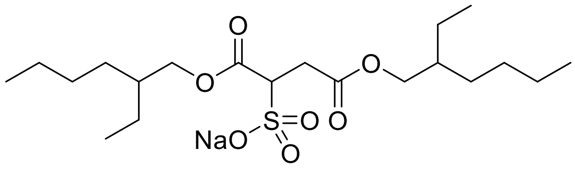
95	Dihexyl sodium sulfosuccinate	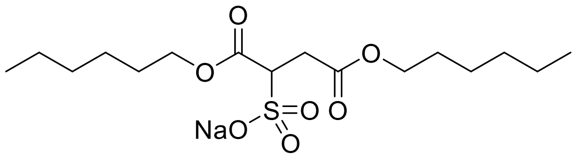
96	Cithrol 10MS	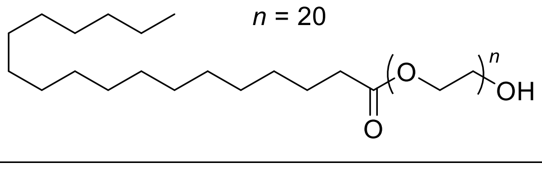

97	Citrhol 4DS	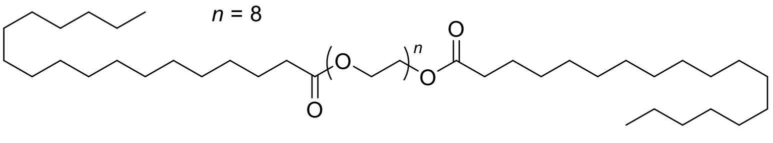
98	Croduret 25-LQ	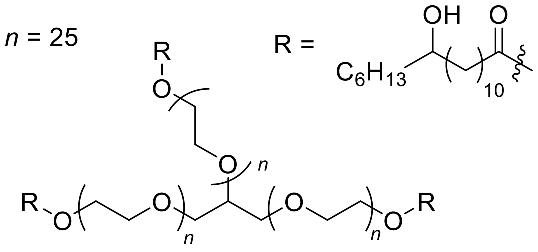
99	Croduret 50-SS	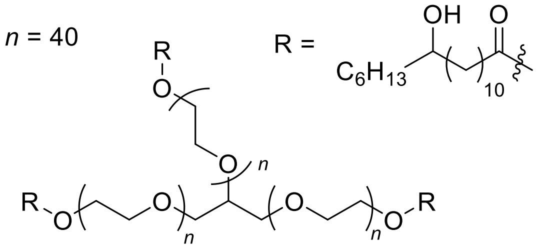
100	Crodasinic LS95	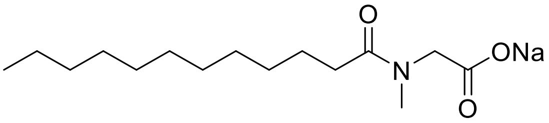

Table S14. Trimmed list of surfactants to reduce uncertainty due to missing data

No.	Name	No.	Name
1	Polysorbate 20	47	Nonyl β-D-glucopyranoside
2	Polysorbate 40	48	Sucrose monolaurate
3	Polysorbate 60	49	CTAB
4	Polysorbate 80	50	CTAC
5	Tween 65	51	DTAB
6	Tween 85	52	DTAC
7	Span 20	53	TTAC
8	Span 40	54	TTAB
9	Span 60	55	CPC ((1-Hexdecyl)pyridinium chloride monohydrate)
10	Span 80	56	DDAB (Di-n-dodecyl)dimethyl ammonium bromide)
11	Span 65	57	CHAPS (3-((3-cholamidopropyl) dimethylammonio)-1-propanesulfonate)
12	Span 85	58	DAPS (N-dodecyl-N,N-dimethyl-3-ammonium-1-propane sulfonate)
13	Octaethylene glycol monodecyl	59	3-(N,N-Dimethylmyristylammonio)propanesulfonate
14	Brij 30	60	Sulfobetaine-16

15	C12E5	61	Lauryldimethylammonio)acetate
16	C12E6	62	TPGS-750-M
17	Brij 35	63	TPGS-1000 (DL-alpha-Tocopherolmethoxypolyethyleneglycol 1000 succinate)
18	Octaethylene glycol monotetradecyl ether	64	SPGS-550-M
19	SP Brij C2 MBAL/Brij 52	65	PTS
20	C16E8	66	PS-750-M
21	Brij 56	67	Sodium Octanoate/Sodium caprylate
22	Brij 58	68	Sodium decanoate
23	Brij S-10	69	Sodium Laurate
24	Brij 93	70	Palmitic acid sodium salt
25	Brij O10	71	Sodium Stearate
26	Brij S20	72	Sodium Oleate
27	Brij 721	73	1-Decanesulfonic acid, sodium salt
28	Brij 700	74	1-Dodecanesulfonic acid, sodium salt
29	Triton-X-15	75	Sodium 1-tetradecanesulfonate
30	Triton-X-35	76	Sodium 1-hexadecanesulfonate
31	Triton-X-45	77	n-Decyl sodium sulfate
32	Triton-X-100	78	SDS
33	Triton-X-102	79	Sodium-1-tetradecyl sulfate
34	Triton-X-114	80	Sodium Hexadecyl sulfate
35	Triton-X-165	81	Sodium n-octadecy sulfate
36	Triton-X-305	82	Sodium 4-n-octylbenzenesulfonate
37	Triton-X-405	83	SDBS
38	Triton-X-705	84	4-Dodecylbenzene sulfonic acid
39	IGEPAL CA720	85	Dioctyl sulfosuccinate sodium salt
40	IGEPAL CO520	86	Dihexyl sodium sulfosuccinate
41	IGEPAL CO630	87	Cithrol 10MS
42	IGEPAL CO720	88	Citrhol 4DS
43	Myrj S8	89	Croduret 25-LQ
44	Myrj S20	90	Croduret 50-SS
45	Myrj 52	91	Crodasinic LS95
46	DDM (N-Dodecyl β-D-maltoside)		

2.5 Principal component analysis

2.5.1 Algorithms

PPCA: Probabilistic Principal Component Analysis is a method to estimate the principal axes when any data vector has one or more missing values.[29,30]

PPCA is based on an isotropic error model. It seeks to relate a *p*-dimensional observation vector *y* to a corresponding *k*-dimensional vector of latent (or unobserved) variable *x*, which is normal with mean zero and covariance *I*(*k*). The relationship is

$$y^T = W * x^T + \mu + \varepsilon,$$

where *y* is the row vector of observed variable, *x* is the row vector of latent variables, and ε is the isotropic error term. ε is Gaussian with mean zero and covariance of $v*I(k)$, where *v* is the residual variance. Here, *k* needs to be smaller than the rank for the residual variance to be greater than 0 ($v>0$). Standard principal component analysis, where the residual variance is zero, is the limiting case of PPCA. The observed variables, *y*, are conditionally independent given the values of the latent variables, *x*. So, the latent variables explain the correlations between the observation variables and the error explains the variability unique to a particular y_i. The *p*-by-*k* matrix *W* relates the latent and observation variables, and the vector μ permits the model to have a nonzero mean. PPCA assumes that the values are missing at random through the data set. This means that whether a data value is missing or not does not depend on the latent variable given the observed data values.

Under this model,

$$y \sim N(\mu, W * W^T + v * I(k)).$$

There is no closed-form analytical solution for *W* and *v*, so their estimates are determined by iterative maximization of the corresponding loglikelihood using an expectation-maximization (EM) algorithm. This EM algorithm handles missing values by treating them as additional latent variables. At convergence, the columns of *W* spans the subspace, but they are not orthonormal. PPCA obtains the orthonormal coefficients, for the components by orthogonalization of *W*.

BPCA: Bayesian Principal Component Analysis combines an EM approach for PCA with a Bayesian model. In standard PCA data far from the training set but close to the principal subspace may have the same reconstruction error. BPCA defines a likelihood function such that the likelihood for data far from the training set is much lower, even if they are close to the principal subspace.[31]

BPCA works iteratively, the complexity is growing with $O(n^3)$ because several matrix inversions are required. The size of the matrices to invert depends on the number of components used for re-estimation.

Finding the optimal number of components for estimation is not a trivial task; the best choice depends on the internal structure of the data. A method called kEstimate is provided to estimate the optimal number of components via cross validation. In general few components are sufficient for reasonable estimation accuracy. See also the package documentation for further discussion about on what data PCA-based missing value estimation makes sense.

Details about the probabilistic model underlying BPCA are found in Oba et. al 2003.[32] The algorithm uses an expectation maximation approach together with a Bayesian model to approximate the principal axes (eigenvectors of the covariance matrix in PCA). The estimation is done iteratively, the algorithm terminates if either the maximum number of iterations was reached or if the estimated increase in precision falls below $1e^-4$.

Complexity: The relatively high complexity of the method is a result of several matrix inversions required in each step. Considering the case that the maximum number of iteration steps is needed, the approximate complexity is given by the term

Where row_miss is the number of rows containing missing values and $O(n^3)$ is the complexity for inverting a matrix of size $components$. Components is the number of components used for re-estimation.

NIPALS: Nonlinear Iterative Partial Least Squares method is a method presented by Wold to allow principal component analysis with missing values.[33] The NIPALS algorithm is applied on the dataset and the obtained PCA model is used to predict the missing values.[34]

The NIPALS algorithm can be modified to accommodate missing values using the method of Martens and Martens (p. 381).[35]

If, for a certain variable *k* [column of *X*], a missing value is encountered in X for a certain object *i* [row of *X*], then the corresponding elements in t_{ih} must also be skipped in the calculation of the loadings, which for *X*-variable *k* is

$$p_{hk} = X_{k,h-1}t'_h/(t'_h t_h)$$

Likewise, if, for a certain sample *i* [row of *X*], a missing value is encountered in *X* for a certain variable *k* [column of *X*], then the corresponding elements in p_{kh} must also be skipped in calculating the scores, which for sample *i* is

$$t_{ih} = X_{i,h-1}p_h/(p'_h p_h)$$

This method may have convergence problems if there are many missing values.

NLPCA: Nonlinear Principal Component Analysis is generally seen as a non-linear generalisation of standard linear principal component analysis. The principal components are generalised from straight lines to curves.[36]

The algorithm, proposed by Kramer,[37] is based on a multi-layer perceptron (deep neural network) with an auto-associative topology, also known as an autoencoder, replicator network, bottleneck or sand glass type network.

The network can be divided into two parts: the first part represents the extraction function $\Phi_{extr}: X \rightarrow Z$, whereas the second part represents the inverse function, the generation or reconstruction function $\Phi_{gen}: Z \rightarrow \hat{X}$. A hidden layer in each part enables the network to perform non-linear mapping functions.

The inverse NLPCA model can be easily extended to be applicable to incomplete datasets. If the *i*th element x_i^n of the *n*th sample vector x^n is missing, the partial error σ_i^n is set to zero before back-propagating; hence this error is ignored, and it has no contribution to the gradients. Thus, the non-linear components are extracted from all the available observations. With these components the original data can be reconstructed, including the missing values. The network output x_i^n gives the estimation of the missing element x_i^n.

2.5.2 PCA results

Due to missing data in the dataset for some experimental values a "traditional" PCA approach could not be used on the dataset. Instead, four PCA approaches which can be employed on incomplete datasets were trialled: Bayesian PCA (BPCA), probabilistic PCA (PPCA), non-linear PCA (NLPCA) and on-linear iterative partial least squares (NIPALS). The relevant code for data normalisation, PCA and plotting are in Section 4.

Table S15. NIPALS loadings for descriptors and PCs

**Descriptor**	**PC1**	**PC2**	**PC3**
Contact_angle_left	0.119849	0.188936	0.146323
Contact_angle_right	0.100006	0.197218	0.134578
Zeta_potential	-0.04506	0.156862	0.317523
Size_low	-0.0092	0.00593	-0.0531
Size_high	-1.61×10^{-5}	0.00063	-0.05109
CMC	-0.07685	-0.03592	-0.02751
Aggregation_number_low	-0.06601	0.102619	0.10661
Aggregation_number_high	-0.04075	0.095504	0.12053
HLB	-0.0652	-0.11232	0.01585
Area_hydrophilic	0.375622	-0.23636	0.082023
Area_hydrophobic	0.2979	0.333079	-0.09485
Volume_hydrophobic	0.30495	0.325878	-0.11382
Volume_hydrophilic	0.36434	-0.25371	0.114468
Rotatable_bonds_hydrophilic	0.364517	-0.25523	0.11818
Rotatable_bonds_hydrophobic	0.285553	0.350459	-0.08946

Longest_chain_length_hydrophilic	0.335862	-0.29298	0.129539
Longest_chain_length_hydrophobic	0.27726	0.28896	-0.0477
Philic_DeltaG_sol	0.189123	-0.26703	0.193799
Philic_Solv_dip	0.00501	0.033201	-0.25541
Philic_HOMO	0.077073	-0.14427	-0.52856
Philic_LUMO	0.074148	-0.17595	-0.49362
Philic_Most_neg	0.053238	0.069546	0.298744
Phobic_Solv_dip	0.202227	0.126911	-0.14908
Double_bonds_hydrophobic	0.069898	0.137099	-0.07263
OH_groups_hydrophilic	0.042828	-0.09282	-0.04409

*Contributors with coefficients above 0.15 are in green

3 Guide to using the *surfactant_map*

The map can be utilised for rational and rapid surfactant screening/optimisation for any given reactions. The step-by-step guide is provided below as guidelines for researchers less familiar with Principal Component Analysis maps. The process is summarised in Figure 5 of the manuscript.

Step 1: Select one surfactant as the initial surfactant. This can be a surfactant which enables the reaction in water from previous experiments, but the results need improvement. If no prior surfactant is known for the reaction, TPGS-750-M is generally a good surfactant to start with, which occupies a central position in the ***surfactant_map***.

Step 2: Select another 7-8 surfactants using the surfactant_map to maximise the space covered by these surfactants. These surfactants will form ***screen1***, and help guide the optimisation in ***screen2*** to the right area of the map. This can be done manually with the ***surfactant_map***, plotted in 3D, or automatically using the Python code in section 5.7. This code randomises the choices of surfactants to maximise the distance between them in the ***surfactant_map*** with only the first selected surfactant being constant as a benchmark.

Step 3: Carry out screen1 using the surfactants selected in Step 2. The best performing surfactants should be compared to identify the area in the surfactant_map where the best results are obtained.

Step 4: Based on the results of screen1, 4-5 surfactants are manually selected around the best performing surfactants for ***screen2***.

Step 5: Perform ***screen2*** and identify the best surfactant for the reaction, enabling further process optimisation through reaction conditions (*e.g.* temperature, concentration, etc.) and stoichiometry.

4 Surfactant screening for reaction (3)

4.1 Standard reaction protocol

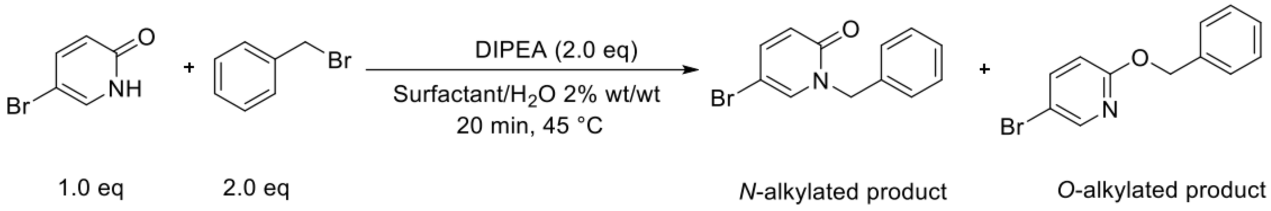

To a 15 mL vial was added surfactant (60 mg, 2 % wt/wt) and deionised water (3 mL). The mixture was stirred for 30 minutes using a PTFE coated magnetic flea 15 x 4.5 mm at 700 RPM and 45 °C. To the same vial was added 5-bromo-2(1H)-pyridone (75 mg, 0.43 mmol, 1.0 eq) and benzyl bromide (105 µL, 0.86 mmol, 2.0 eq) and the reaction was stirred for a further 15 minutes. DIPEA (150 µL, 0.86 mmol, 2.0 eq) were added to the vial and the reaction was stirred for 45 °C for 20 minutes. The reaction was quenched with brine (3 mL) and extracted using DCM (3 x 5 mL). The combined organics were dried using sodium sulfate (Na_2SO_4) and concentrated on the rotary evaporator giving a clear solution / off white solution that sometimes solidified upon standing (suspected to be surfactant dependent).

N-alkylated product

TLC: 4:10 EtOAc : Hexane, R_f = 0.83, UV active

^{1}H NMR (501 MHz, $CDCl_3$) δ 7.39 – 7.29 (m, 7H), 6.54 (d, *J* = 9.7 Hz, 1H), 5.10 (s, 2H).

^{13}C NMR (101 MHz, $CDCl_3$) δ 161.1, 142.5, 137.0, 135.6, 129.1, 128.4, 128.3, 122.4, 98.2, 52.2.

HRMS $[M+H]^+$ calc. 264.0019, 265.9998 ; found 264.0015, 265.9995.

Data found to match those previously reported.[38]

O-alkylated product (not isolated)

^{1}H NMR (501 MHz, $CDCl_3$) δ 8.19 (d, *J* = 2.5 Hz, 1H), 7.64 (dd, *J* = 8.8, 2.5 Hz, 1H), 7.28 – 7.39 (m,7H), 6.71 (d, *J* = 8.8 Hz, 1H), 5.32 (s, 2H).

^{13}C NMR (101 MHz, $CDCl_3$) δ 162.4, 147.4, 141.2, 136.9, 128.5, 128.1, 128.0, 113.0, 111.9, 68.0.

4.2 Surfactant screening results

Table S16 - Yield of N-alkylated and O-alkylated product of the surfactant screen for reaction (3)

Number	Surfactant	*N*-alkylated yield (%)	*O*-alkylated yield (%)
1	None	93	7
2	Span 60	83	7
3	TTAB	95	5
4	Triton-X-305	79	8
5	Sulfobetain-16	95	5
6	TPGS-750-M	64	28
7	Cithrol 4DS	16	7
8	Crodasinic LS95	84	8
9	SOBS	88	7
10	C16E8	48	45
11	Igepal CO520	55	8

4.3 Reaction with allyl bromide

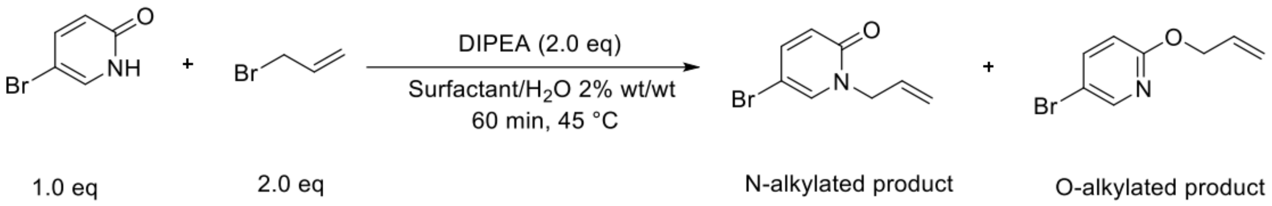

To a 15 mL vial was added surfactant (60 mg, 2 % wt/wt) and deionised water (3 mL). The mixture was stirred for 30 minutes using a PTFE coated magnetic flea 15 x 4.5 mm at 700 RPM and 45 °C. To the same vial was added 5-bromo-2(1H)-pyridone (75 mg, 0.43 mmol, 1.0 eq) and allyl bromide (75 µL, 0.86 mmol, 2.0 eq) and the reaction was stirred for a further 15 minutes. DIPEA (150 µL, 0.86 mmol, 2.0 eq) were added to the vial and the reaction was stirred for 45 °C for 60 minutes. The reaction was quenched with brine (3 mL) and extracted using DCM (3 x 5 mL). The combined organics were dried (Na_2SO_4), and concentrated on the rotary evaporator giving a clear solution / off – white solution that sometimes solidified upon standing.

The crude product was purified using normal phase flash chromatography using 10 – 60% EtOAc:Hexane.

N-alkylated product

TLC: 4.5:10 EtOAc : Hexane, R_f = 0.16, UV active

^{1}H NMR (501 MHz, $CDCl_3$) δ 7.37 – 7.32 (m, 2H), 6.50 (d, *J* = 9.6, 1H), 5.91 (ddt, *J* = 10.2, 17.1, 5.9 Hz, 1H), 5.30 (dd, *J* = 10.2 Hz, 1H), 5.23 (dd, *J* = 17.1, Hz, 1H), 4.52 (dt, J = 5.9, 1.5 Hz, 2H).

^{13}C NMR (126 MHz, $CDCl_3$) δ 160.7, 142.7, 137.1, 131.9, 122.3, 119.7, 98.0, 51.2.

HRMS $[M+H]^+$ calc. 213.9862, 215.9842 ; found 213.9864, 215.9843.

O-alkylated product

TLC: 4.5:10 EtOAc : Hexane, Rf = 0.45, UV active

^{1}H NMR (501 MHz, $CDCl_3$) δ 8.11 (d, *J* = 2.5 Hz, 1H), 7.58 (dd, *J* = 2.5, 8.7 Hz, 1H), 6.62 (d, *J* = 8.7 Hz, 1H), 6.00 (ddt, *J* = 10.5, 17.3, 5.5 Hz, 1H), 5.31 (d, *J* = 17.3 Hz, 1H), 5.19 (d, *J* = 10.3 Hz, 2H).

^{13}C NMR (126 MHz, $CDCl_3$) δ 162.2, 147.4, 141.2, 133.1, 117.7, 112.8, 111.8, 66.9.

4.4 Surfactant screening results

Table S17 - Yield of *N*-alkylated and *O*-alkylated product of the surfactant screen for reaction with allyl bromide

Number	Surfactant	*N*-alkylated yield (%)	*O*-alkylated yield (%)
1	None	70	6
2	Span 60	56	24
3	TTAB	95	5
4	Triton-X-305	71	23
5	Sulfobetain-16	100	0
6	TPGS-750-M	63	28
7	Cithrol 4DS	56	18
8	Crodasinic LS95	78	7
9	SOBS	66	34
10	C16E8	79	14
11	Igepal CO520	57	29

5 Nucleophilic fluorination with fluoride anion

5.1 Fluorination of episulfoniums

5.1.1 Standard reaction protocol

For reaction optimisation

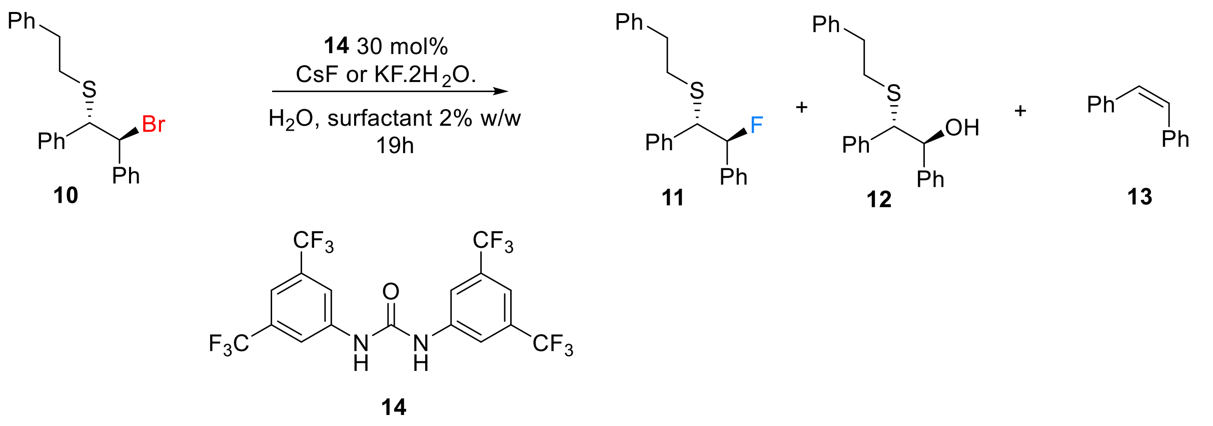

In a 4-dram glass vial (2.1 × 7 cm, 14 mL) equipped with a cross-bar stirrer (1 cm × 1 cm) were sequentially added the substrate **10**, catalyst, fluoride (CsF/KF/NaF), organic co-solvent, and pre-stirred surfactant solution (2% w/w in deionised H_2O). The reaction vial was sealed with a lid (PTFE septum) and was stirred on a custom-made aluminium block with 1 inch off-set stirring at an appropriate stirring speed, temperature, and duration (detailed in the optimisation table). The crude reaction mixture was diluted with DCM and deionised H_2O, the layers were partitioned utilising brine, and the aqueous phase was extracted three times with DCM. The organic layers were combined, dried ($MgSO_4$), filtered and concentrated *in vacuo*. The crude mixture was dissolved in $CDCl_3$ and analysed by ^1H and ^{19}F NMR.

For the substrate scope exploration

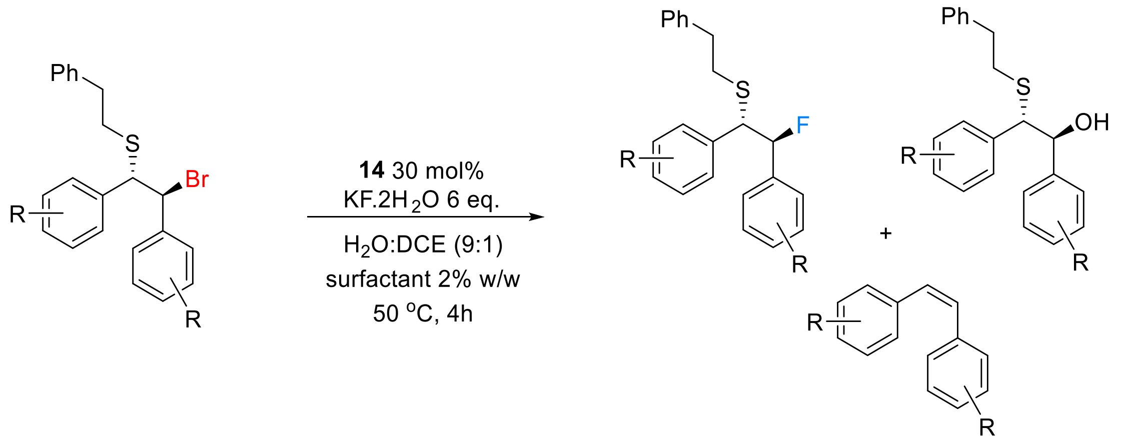

In a 4-dram glass vial (2.1 × 7 cm, 14 mL) equipped with a cross-bar stirrer (1 cm × 1 cm) were sequentially added the appropriate substrate, catalyst **14** (0.3 eq), KF·H_2O (6 eq.), and pre-stirred surfactant solution (2% w/w in deionised H_2O) in water : DCE (9:1). The reaction vial was sealed with a lid (PTFE septum) and was stirred for 4h on a custom-made aluminium block with 1 inch off-set stirring at 500 rpm, 50 °C. The crude

reaction mixture was diluted with DCM and deionised H_2O, the layers were partitioned utilising brine, and the aqueous phase was extracted three times with DCM. The organic layers were combined, dried ($MgSO_4$), filtered and concentrated *in vacuo*. The crude mixture was dissolved in $CDCl_3$ and analysed by ^1H and ^{19}F NMR.

5.1.2 Determination of product ratio

Example .No. 18 from Table 2 in the manuscript (DCE:H_2O 9:1, KF·$2H_2O$, Span 80)

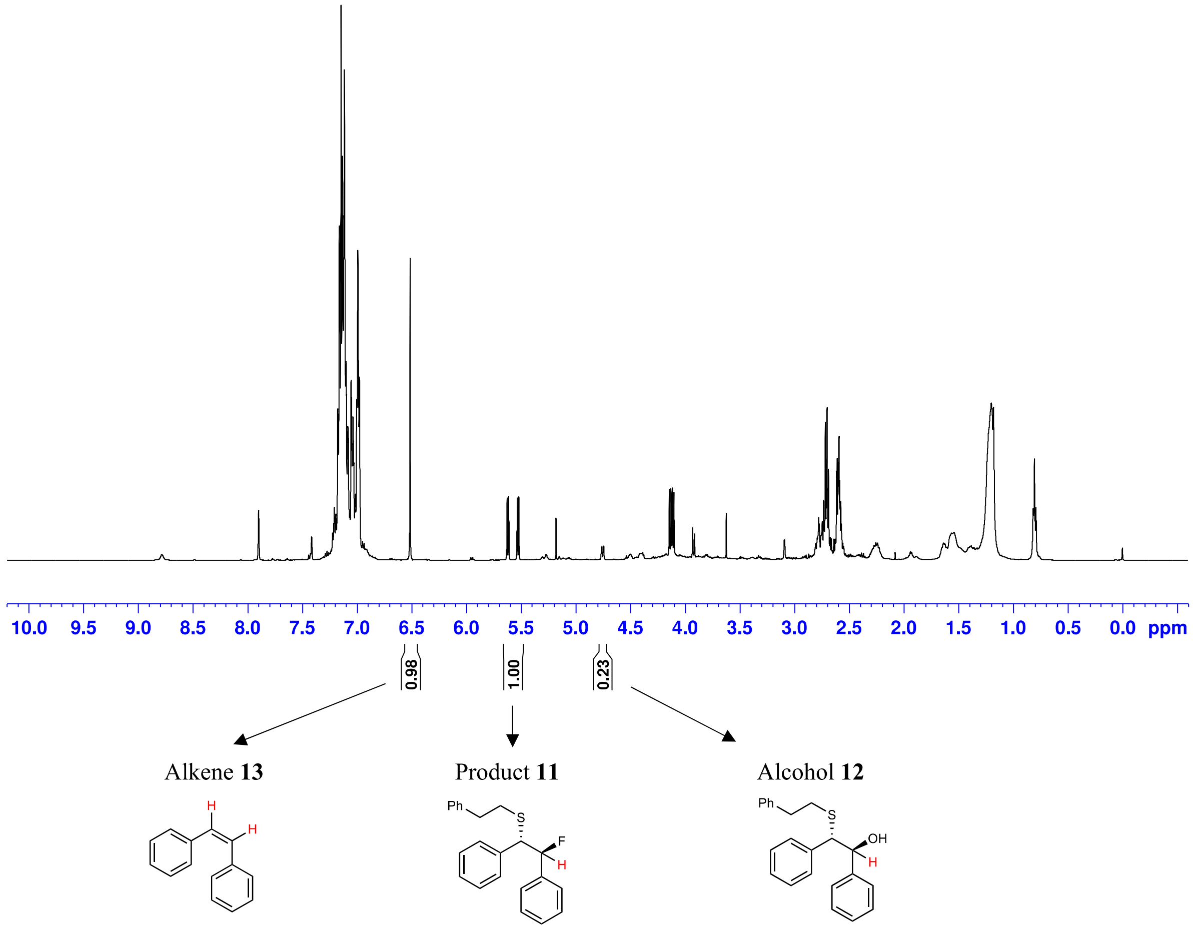

Figure S53. Determination of product ratio using ^1H NMR

Conversion calculation using ^1H NMR integration of CH peaks

$$\% \text{ Conversion product } \mathbf{11} = \frac{\mathbf{11}}{\frac{\mathbf{13}}{2}+\mathbf{11}+\mathbf{12}} * 100 = \frac{1.0}{\frac{0.98}{2}+1.0+0.23} * 100 = 58\%$$

$$\% \text{ Conversion alcohol } \mathbf{12} = \frac{\mathbf{12}}{\frac{\mathbf{13}}{2}+\mathbf{11}+\mathbf{12}} * 100 = \frac{0.23}{\frac{0.98}{2}+1.0+0.23} * 100 = 13\%$$

$$\% \text{ Conversion alkene } \mathbf{13} = \frac{\mathbf{13}}{\frac{\mathbf{13}}{2}+\mathbf{11}+\mathbf{12}} * 100 = \frac{\frac{0.98}{2}}{\frac{0.98}{2}+1.0+0.23} * 100 = 29\%$$

5.1.3 Synthesis of starting materials and catalysts

1,3-Bis(3,5-bis(trifluoromethyl)phenyl)urea 14

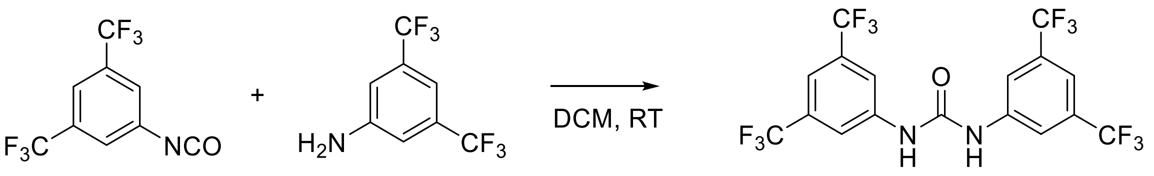

To a solution of 3,5-bis(trifluoromethyl)phenyl isocyanate (0.51 g, 2.0 mmol) in DCM (4.0 mL), was added 3,5-bis(trifluoromethyl)aniline (0.46 g, 2.0 mmol) at rt. After stirring the reaction mixture at rt for 4-5 days the resulting solids were collected by filtration. The solids were then washed using cold DCM (3 × 5 mL) and dried under high vacuum for 24 h to afford the desired product **14** (0.84 g, 87% yield) as a white solid.

δ_H (500 MHz, acetone-d_6): 8.92 (br s, 2H), 8.09 (4H, s), 7.54 (2H, s).

δ_C (126 MHz, acetone-d_6): 152.2 (C=O), 141.4 (Ar C), 131.7 (ArC-CF_3, q, J = 33.0 Hz), 124.6 (ArC), 122.4 (Ar C), 118.7 (Ar CH), 115.5 (Ar CH).

Characterisation data is in agreement with those previously reported:[40] δ_H (acetone-d_6, 500 MHz): 9.05 (br s, 2H), 8.22 (s, 4H), 7.67 (s, 2H); δ_C (acetone-d_6, 125 MHz,): 153.1 (C=O), 142.2, 132.6 (q, ArC-CF_3), 125.5, 123.3, 119.6, 116.3.

cis-Stilbene* 13 *(commercial)

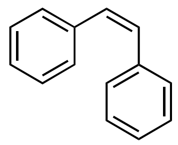

δ_H (500 MHz, $CDCl_3$): 7.48-7.20 (10H, m), 6.73 (2H, s).

δ_C (126 MHz, $CDCl_3$): 137.4, 130.4, 129.0, 128.3, 127.2.

Preparation of substituted cis-stilbenes[41]

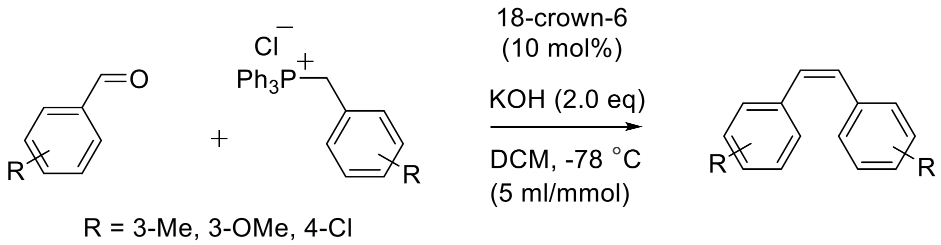

cis-3,3'-Dimethylstilbene

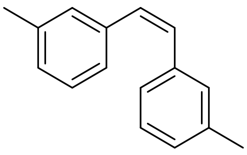

m-Tolualdehyde (561 mg, 4.5 mmol) and 18-crown-6 (119 mg, 0.45 mmol) were added to a solution of (3-methylbenzyl)triphenylphosphonium chloride (0.530 mL, 4.5 mmol) in dichloromethane (22.5 mL). The mixture was cooled to −78 °C, and freshly powdered potassium hydroxide (505 mg, 9 mmol) was added under magnetic stirring. After stirring at −78 °C for 6 h, the mixture was diluted with dichloromethane, filtered off, and washed with water. The organic phase was dried over anhydrous magnesium sulfate. After filtration and concentration of the organic phase, the residue was purified by flash column chromatography using silica gel as the stationary phase and ethyl acetate–hexane (1:49) as mobile phase to afford the desired product as a colourless liquid (200 mg, 21% yield).

δ_H (500 MHz, $CDCl_3$): 7.20-7.02 (8H, m, Ar H), 6.59 (2H, s), 2.31 (6H, s).

δ_C (126 MHz, $CDCl_3$): 137.7, 137.3, 130.2, 129.6, 128.0, 127.8, 125.9, 21.4.

Characterisation data is in agreement with those previously reported:[41] ^{1}H NMR (300 MHz, $CDCl_3$, δ): 7.19–7.02 (m, 8H), 6.54 (s, 2H), 2.27 (s, 6H). ^{13}C NMR (100.6 MHz, $CDCl_3$, δ): 137.6, 137.2, 130.2, 129.6, 128.0, 127.8, 125.9, 21.3.

cis-3,3'-Dimethoxystilbene

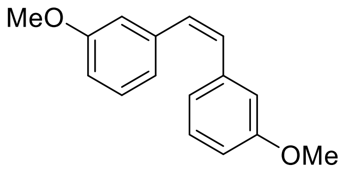

m-Anisaldehyde (0.61 mL, 5 mmol) and 18-crown-6 (132 mg, 0.5 mmol) were added to a solution of (3-methoxybenzyl)triphenylphosphonium chloride (2.09 g, 5 mmol) in dichloromethane (25 mL). The mixture was cooled to −78 °C, and freshly powdered potassium hydroxide (561 mg, 10 mmol) was added under magnetic stirring. After stirring at −78 °C for 6 h, the mixture was diluted with dichloromethane, filtered off, and washed with water. The organic phase was dried over anhydrous magnesium sulfate. After filtration and concentration of the organic phase, the residue was purified by flash column chromatography using silica gel as the stationary phase and ethyl acetate–hexane (1:49) as mobile phase to afford the desired product as a yellow liquid (290 mg, 24% yield).

δ_H (500 MHz, $CDCl_3$): 7.18 (2H, *t*, J = 7.9 Hz, Ar H), 6.90-6.75 (6H, m, Ar H), 6.61 (2H, s), 3.69 (6H, s).

δ_C (126 MHz, $CDCl_3$): 159.4, 138.5, 130.4, 129.2, 121.5, 113.8, 113.3, 55.1.

Characterisation data is in agreement with those previously reported:[41] ^{1}H NMR (300 MHz, $CDCl_3$, δ): 7.18 (t, J = 7.8 Hz, 2H), 6.86–6.73 (m, 6H), 6.58 (s, 2H), 3.66 (s, 6H). ^{13}C NMR (75.5 MHz, $CDCl_3$, δ): 159.4, 138.6, 130.4, 129.2, 121.5, 113.9, 113.3, 55.1.

cis-4,4'-Dichlorostilbene

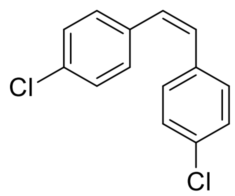

4-Chlorobenzaldehyde (703 mg, 5 mmol) and 18-crown-6 (132 mg, 0.5 mmol) were added to a solution of 4-chlorobenzyltriphenylphosphonium chloride (2.12 g, 5 mmol) in dichloromethane (25 mL). The mixture was cooled to −78 °C, and freshly powdered potassium hydroxide (561 mg, 10 mmol) was added under magnetic stirring. After stirring at −78 °C for 6 h, the mixture was diluted with dichloromethane, filtered off, and washed with water. The organic phase was dried over anhydrous magnesium sulfate. After filtration and concentration of the organic phase, the residue was purified by flash column chromatography using silica gel as the stationary phase and ethyl acetate–hexane (1:49) as mobile phase to afford the desired product as a yellow liquid (670 mg, 54% yield).

δ_H (500 MHz, $CDCl_3$): 7.12 (4H, d, *J* = 8.5 Hz, Ar H), 7.06 (4H, d, *J* = 8.5 Hz, Ar H), 6.47 (2H, s).

δ_C (126 MHz, $CDCl_3$): 135.3, 133.0, 130.2, 129.6, 128.6.

Characterisation data is in agreement with those previously reported:[41] ^{1}H NMR (300 MHz, $CDCl_3$, δ): 7.21 (d, *J* = 6.5 Hz, 4H), 7.15 (d, *J* = 6.5 Hz, 4H), 6.63 (s, 2H). ^{13}C NMR (100.6 MHz, $CDCl_3$, δ): 135.2, 133.0, 130.1, 129.6, 128.5.

Preparation of substituted cis-stilbene oxides[41]

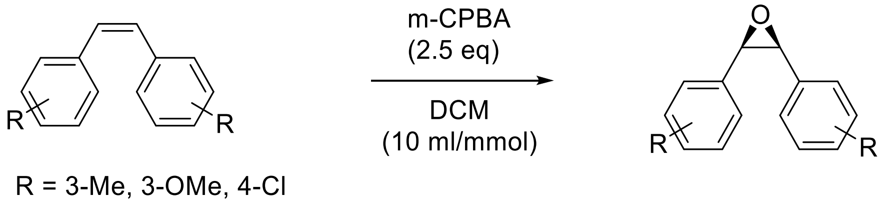

2,3-Di-m-tolyl-oxirane

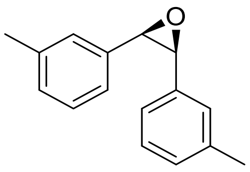

To a solution of cis-3,3′-Dimethylstilbene (200 mg, 0.96 mmol) in dichloromethane (9.6 mL) was added *m*CPBA (414 mg, 2.4 mmol) at 0 °C. After stirring at room temperature for 15 h, the mixture was diluted with diethyl ether, and washed with 5% $NaHCO_3$. The organic phase was dried over anhydrous magnesium sulphate. Following filtration and concentration, the residue was purified by column chromatography using silica gel as the stationary phase and ethyl acetate/hexane (1:19) as mobile phase to deliver the desired product as a colourless liquid (124 mg, 57% yield).

δ_H (500 MHz, $CDCl_3$): 6.93-6.74 (8H, m, Ar H), 4.13 (2H, s), 2.06 (6H, s).

δ_C (126 MHz, $CDCl_3$): 137.3, 134.3, 128.3, 127.65, 127.64, 123.9, 58.9, 21.3.

Characterisation data is in agreement with those previously reported:[41] ^{1}H NMR (300 MHz, $CDCl_3$, δ): 7.09–6.94 (m, 8H), 4.30 (s, 2H), 2.19 (s, 6H). ^{13}C NMR (75.4 MHz, $CDCl_3$, δ): 137.3, 134.5, 129.0, 128.4, 123.9, 59.9, 21.4.

2,3-Bis-(3-methoxy-phenyl)-oxirane

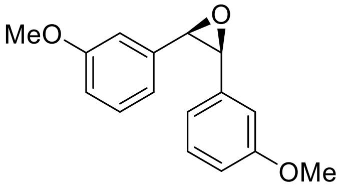

To a solution of cis-3,3′-Dimethoxystilbene (290 mg, 1.21 mmol) in dichloromethane (12.1 mL) was added *m*CPBA (521 mg, 3.0 mmol) at 0 °C. After stirring at room temperature for 15 h, the mixture was diluted with diethyl ether, and washed with 5% $NaHCO_3$. The organic phase was dried over anhydrous magnesium sulphate. Following filtration and concentration, the residue was purified by column chromatography using silica gel as the stationary phase and ethyl acetate/hexane (1:19) as mobile phase to deliver the desired product as a colourless liquid (140 mg, 55% yield).

δ_H (500 MHz, $CDCl_3$): 7.01 (2H, *t*, J = 7.7 Hz, Ar H), 6.73 (2H, d, J = 7.7 Hz, Ar H), 6.67-6.58 (4H, m, ArH), 4.24 (2H, s), 3.57 (6H, s).

δ_C (126 MHz, $CDCl_3$): 159.2, 136.0, 128.9, 119.4, 113.6, 112.0, 59.8, 55.1.

Characterisation data is in agreement with those previously reported:[41] ^{1}H NMR (300 MHz, $CDCl_3$, δ): 7.13–7.07 (t, J = 7.3 Hz, 2H), 6.82 (d, J = 7.5 Hz, 2H), 6.71–6.68 (m, 4H), 4.32 (s, 2H), 3.67 (s, 6H). ^{13}C NMR (100.6 MHz, $CDCl_3$, δ): 159.1, 135.9, 128.8, 119.3, 113.6, 112.0, 59.7, 55.1.

2,3-Bis-(4-chloro-phenyl)-oxirane

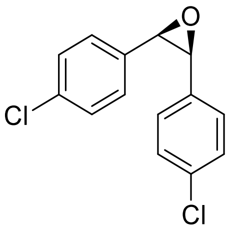

To a solution of cis-4,4′-Dichlorostilbene (667 mg, 2.68 mmol) in dichloromethane (26.7 mL) was added *m*CPBA (1.15 g, 6.69 mmol) at 0 °C. After stirring at room temperature for 15 h, the mixture was diluted with diethyl ether, and washed with 5% $NaHCO_3$. The organic phase was dried over anhydrous magnesium sulfate. Following filtration and concentration, the residue was purified by column chromatography using silica gel as

the stationary phase and ethyl acetate/hexane (1:19) as mobile phase to deliver the desired product as a white solid (590 mg, 83% yield).

δ_H (500 MHz, $CDCl_3$): 7.19 (4H, d, *J* = 8.5 Hz, Ar H), 7.11 (4H, d, *J* = 8.5 Hz, Ar H), 4.34 (2H, s).

δ_C (126 MHz, $CDCl_3$): 133.6, 132.6, 128.2, 128.1, 59.1.

Characterisation data is in agreement with those previously reported:[41] ^1H NMR (300 MHz, $CDCl_3$, δ): 7.18 (d, *J* = 8.4 Hz, 4H), 7.09 (d, *J* = 9.0 Hz, 4H), 4.31 (s, 2H). ^{13}C NMR (75.4 MHz, $CDCl_3$, δ): 133.6, 132.5, 128.3, 59.1.

Preparation of substituted hydroxy sulfides[42]

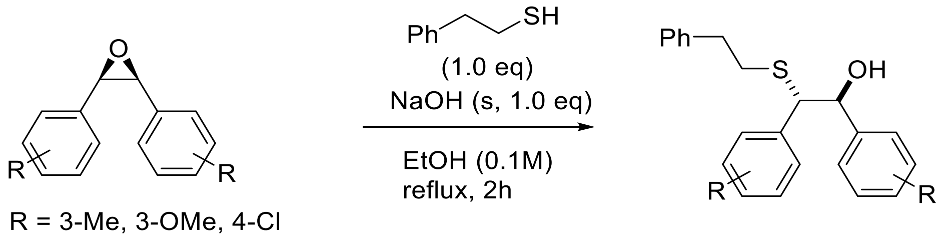

rac-2-(phenethylthio)-1,2-diphenylethan-1-ol 12

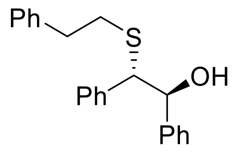

12

To a solution of *cis*-stilbene oxide (1.00 g, 5.1 mmol) in EtOH (51 mL), were added NaOH (s, 0.20 g, 5.1 mmol) and 2-phenylethanethiol (0.68 mL, 5.1 mmol) at rt. After stirring the reaction mixture at reflux for 2 h (monitored by TLC using hexane : DCM = 8 : 2 as eluent) the solvent was removed *in vacuo*. The crude mixture was diluted with Et_2O and H_2O, the layers were partitioned, and the aqueous phase was extracted three times with Et_2O. The organic layers were combined, dried ($MgSO_4$), filtered and concentrated *in vacuo*. Purification via flash column chromatography on silica gel (pentane/Et_2O = 100:0 to 80:20 gradient) gave **12** (1.52 g, 89% yield) as a yellow solid.

δ_H (500 MHz, $CDCl_3$): 7.22-7.16 (2H, m), 7.15-7.06 (7H, m), 7.06-7.01 (4H, m), 7.01-6.97 (2H, m), 4.76 (1H, d, *J* 8.6), 3.92 (1H, d, *J* 8.5), 2.78-2.68 (2H, m), 2.65-2.53 (2H, m).

δ_C (126 MHz, $CDCl_3$): 140.9 (Ar C), 140.2 (Ar C), 139.5 (Ar C), 128.6 (Ar CH), 128.5 (Ar CH), 128.5 (Ar CH), 128.3 (Ar CH), 128.0 (Ar CH), 127.7 (Ar CH), 127.3 (Ar CH), 126.7 (Ar CH), 126.4 (Ar CH), 77.3 (CH), 60.3 (CH), 36.2 (CH_2), 33.3 (CH_2).

Characterisation data is in agreement with those previously reported:[42] ^1H NMR (500 MHz, $CDCl_3$) δ = 7.32–7.26 (m, 2H), 7.25–7.17 (m, 7H), 7.16–7.12 (m, 4H), 7.12–7.08 (m, 2H), 4.86 (dd, *J* = 8.5, 2.7 Hz, 1H), 4.03 (d, *J* = 8.5 Hz, 1H), 3.17 (d, *J* = 2.7 Hz, 1H), 2.87–2.78 (m, 2H), 2.76–2.62 (m, 2H), ^{13}C NMR (126 MHz,

$CDCl_3$) δ = 141.1, 140.3, 139.6, 128.7, 128.6, 128.6, 128.4, 128.1, 127.8, 127.4, 126.8, 126.5, 77.4, 60.4, 36.3, 33.4.

rac-2-(phenethylthio)-1,2-di-m-tolylethan-1-ol

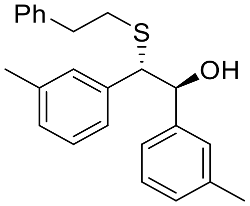

To a solution of 2,3-Di-m-tolyl-oxirane (124 mg, 0.55 mmol) in EtOH (5.5 mL), were added NaOH (s, 22 mg, 0.55 mmol) and 2-phenylethanethiol (0.074 mL, 0.55 mmol) at rt. After stirring the reaction mixture at reflux for 2 h (monitored by TLC using hexane : DCM = 8 : 2 as eluent) the solvent was removed *in vacuo*. The crude mixture was diluted with Et_2O and H_2O, the layers were partitioned, and the aqueous phase was extracted three times with Et_2O. The organic layers were combined, dried ($MgSO_4$), filtered and concentrated *in vacuo*. Purification via flash column chromatography on silica gel (pentane/Et_2O = 100:0 to 80:20 gradient) gave the desired product as a yellow oil (158 mg, 78% yield).

δ_H (500 MHz, $CDCl_3$): 7.18-6.78 (13H, m, ArH), 4.74 (1H, d, *J* = 8.1 Hz), 3.90 (1H, d, *J* = 8.1 Hz), 2.75-2.67 (2H, m), 2.64-2.51 (2H, m), 2.19 (3H, s), 2.18 (3H, s).

δ_C (126 MHz, $CDCl_3$): 141.0 (Ar C), 140.3 (Ar C), 139.5 (Ar C), 137.8 (Ar C), 137.6 (Ar C), 129.3 (Ar CH), 128.5 (Ar CH), 128.4 (Ar CH), 128.4 (Ar CH), 128.1 (Ar CH), 128.1 (Ar CH), 127.8 (Ar CH), 127.2 (Ar CH), 126.4 (Ar CH), 125.7 (Ar CH), 123.8 (Ar CH), 60.1, 36.2, 33.3, 31.6, 21.4, 21.4.

Characterisation data is in agreement with those previously reported:[42] ^{1}H NMR (400 MHz, $CDCl_3$) δ = 7.24–7.12 (m, 3H), 7.08–7.00 (m, 4H), 6.98–6.83 (m, 6H), 4.77 (d, *J* = 8.1 Hz, 1H), 3.94 (d, *J* = 8.1 Hz, 1H), 3.02 (s br, 1H), 2.78–2.70 (m, 2H), 2.68–2.55 (m, 2H), 2.23 (s, 3H), 2.22 (s, 3H), ^{13}C NMR (101 MHz, $CDCl_3$) δ = 141.1, 140.4, 139.6, 138.0, 137.7, 129.4, 128.6, 128.6, 128.5, 128.2, 128.2, 127.9, 127.4, 126.5, 125.8, 124.0, 77.2, 60.1, 36.3, 33.4, 21.5, 21.5.

rac-1,2-bis(3-methoxyphenyl)-2-(phenethylthio)ethan-1-ol

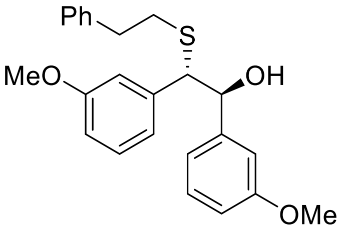

To a solution of 2,3-Bis-(3-methoxy-phenyl)-oxirane (140 mg, 0.55 mmol) in EtOH (5.5 mL), were added NaOH (s, 22 mg, 0.55 mmol) and 2-phenylethanethiol (0.074 mL, 0.55 mmol) at rt. After stirring the reaction mixture at reflux for 2 h (monitored by TLC using hexane : DCM = 8 : 2 as eluent) the solvent was removed *in vacuo*. The crude mixture was diluted with Et_2O and H_2O, the layers were partitioned, and the aqueous phase

was extracted three times with Et_2O. The organic layers were combined, dried ($MgSO_4$), filtered and concentrated *in vacuo*. Purification via flash column chromatography on silica gel (pentane/Et_2O = 100:0 to 80:20 gradient) gave the desired product as a yellow oil (129 mg, 60% yield).

δ_H (500 MHz, $CDCl_3$): 7.33-7.18 (3H, m, ArH), 7.17-7.08 (4H, m, ArH), 6.80-6.67 (6H, m, ArH), 4.82 (1H, d, *J* = 8.3 Hz), 3.98 (1H, d, *J* = 8.3 Hz), 3.74 (3H, s), 3.72 (3H, s), 2.86-2.78 (2H, m), 2.77-2.59 (2H, m).

δ_C (126 MHz, $CDCl_3$): 159.5 (Ar C), 159.3 (Ar C), 142.6 (Ar C), 141.1 (Ar C), 140.2 (Ar C), 129.2 (Ar CH), 129.0 (Ar CH), 128.5 (Ar CH), 128.5 (Ar CH), 126.4 (Ar CH), 121.1 (Ar CH), 119.1 (Ar CH), 114.2 (Ar CH), 113.6 (Ar CH), 112.9 (Ar CH), 111.9 (Ar CH), 77.1, 60.2, 55.2, 55.2, 36.2, 33.4.

Characterisation data is in agreement with those previously reported:[42] ^1H NMR (400 MHz, $CDCl_3$) δ = 7.25–7.18 (m, 2H), 7.17–7.11 (m, 1H), 7.10–7.00 (m, 4H), 6.71–6.60 (m, 6H), 4.75 (dd, *J* = 8.3, 2.8 Hz, 1H), 3.91 (d, *J* = 8.3 Hz, 1H), 3.66 (s, 3H), 3.64 (s, 3H), 3.05 (d, *J* = 2.8 Hz, 1H), 2.78–2.72 (m, 2H), 2.68–2.58 (m, 2H), ^{13}C NMR (101 MHz, $CDCl_3$) δ = 159.6, 159.4, 142.7, 141.2, 140.3, 129.3, 129.1, 128.7, 128.6, 126.5, 121.2, 119.2, 114.3, 113.7, 113.0, 112.1, 77.2, 60.2, 55.3, 55.3, 36.3, 33.5.

rac-1,2-bis(4-chlorophenyl)-2-(phenethylthio)ethan-1-ol

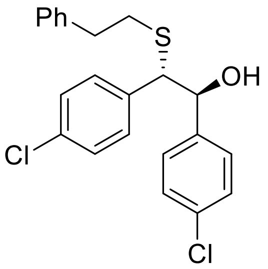

To a solution of 2,3-Bis-(4-chloro-phenyl)-oxirane (0.59 g, 2.2 mmol) in EtOH (22 mL), were added NaOH (s, 89 mg, 2.2 mmol) and 2-phenylethanethiol (0.297 mL, 2.2 mmol) at rt. After stirring the reaction mixture at reflux for 2 h (monitored by TLC using hexane : DCM = 8 : 2 as eluent) the solvent was removed *in vacuo*. The crude mixture was diluted with Et_2O and H_2O, the layers were partitioned, and the aqueous phase was extracted three times with Et_2O. The organic layers were combined, dried ($MgSO_4$), filtered and concentrated *in vacuo*. Purification via flash column chromatography on silica gel (pentane/Et_2O = 100:0 to 80:20 gradient) gave the desired product as a yellow oil (0.65 g, 73% yield).

δ_H (500 MHz, $CDCl_3$): 7.34-6.97 (13H, m, Ar H), 4.75 (1H, d, *J* = 8.7 Hz), 3.87 (1H, d, *J* = 8.7 Hz), 2.87-2.78 (2H, m), 2.77-2.62 (2H, m).

δ_C (126 MHz, $CDCl_3$): 139.9 (Ar C), 139.0 (Ar C), 137.7 (Ar C), 133.6 (Ar C), 133.2 (Ar C), 129.9 (Ar CH), 128.6 (Ar CH), 128.3 (Ar CH), 128.0 (Ar CH), 126.5 (Ar CH), 76.6, 59.6, 36.1, 33.4.

Characterisation data is in agreement with those previously reported:[42] ^1H NMR (400 MHz, $CDCl_3$) δ = 7.23–7.17 (m, 7H), 7.15–7.09 (m, 2H), 7.06–6.99 (m, 4H), 4.65 (dd, *J* = 8.6, 2.5 Hz, 1H), 3.79 (d, *J* = 8.6 Hz, 1H), 3.10 (d, *J* = 2.5 Hz, 1H), 2.74 (t, *J* = 7.3 Hz, 2H), 2.64–2.55 (m, 2H), ^{13}C NMR (101 MHz, $CDCl_3$) [overlapping signals] δ = 140.0, 139.2, 137.8, 133.6, 133.3, 130.0, 128.7,128.7, 128.4, 128.2, 126.7, 76.7, 59.7, 36.2, 33.4.

Preparation of substituted bromo sulfides[42]

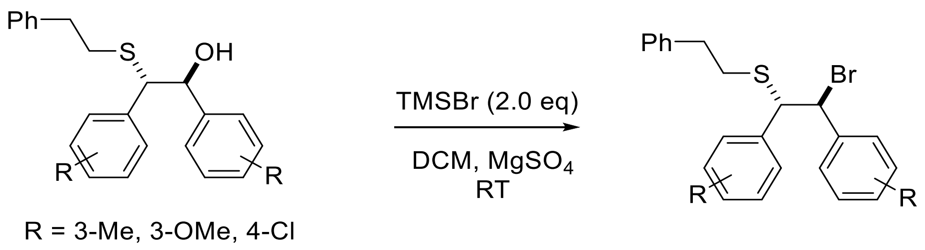

rac-2-bromo-1,2-diphenylethyl(phenethyl)sulfane 10

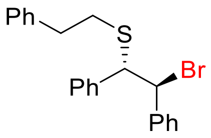

To a solution of hydroxy sulfide **12** (1.64 g, 4.9 mmol) in DCM (4.9 mL) taken in a round bottom flask equipped with a stirring bar, was added anhydrous $MgSO_4$ (2.45 g). This was followed by dropwise addition of TMSBr (1.27 mL, 9.8 mmol, 2 eq). After stirring the reaction mixture at rt for 1 h, the crude mixture was filtered over celite and solvent was removed *in vacuo* to afford the desired product. Recrystallisation using Hexane:Et_2O (20:1) gave **10** (1.30 g, 67% yield) as a white solid.

δ_H (500 MHz, $CDCl_3$): 7.19-6.93 (15H, m, ArH), 5.16 (1H, d, *J* = 9.1 Hz), 4.39 (1H, d, *J* = 9.1 Hz), 2.79-2.63 (2H, m, CH_2), 2.50-2.42 (2H, m, CH_2).

δ_C (126 MHz, $CDCl_3$): 140.3, 139.6, 138.5, 128.9, 128.5, 128.4, 128.3, 128.2, 128.2, 128.1, 127.7, 126.3, 58.2, 58.1, 36.0, 33.6.

Characterisation data is in agreement with those previously reported:[42] ^{1}H NMR (400 MHz, $CDCl_3$) δ = 7.27–7.04 (m, 15H), 5.24 (d, *J* = 9.0 Hz, 1H), 4.48 (d, *J* = 9.0 Hz, 1H), 2.89–2.73 (m, 2H), 2.60–2.53 (m, 2H), ^{13}C NMR (100 MHz, $CDCl_3$) δ = 140.3, 139.6, 138.5, 128.9, 128.5, 128.5, 128.4, 128.3, 128.3 128.2, 127.8, 126.3, 58.1, 58.0, 36.0, 33.6

rac-2-bromo-1,2-di-m-tolylethyl(phenethyl)sulfane 17

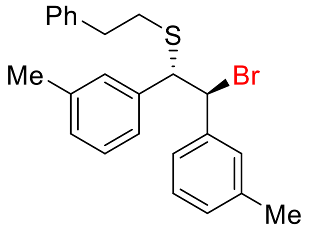

To a solution of rac-2-(phenethylthio)-1,2-di-m-tolylethan-1-ol (158 mg, 0.43 mmol) in DCM (0.43 mL) taken in a round bottom flask equipped with a stirring bar, was added anhydrous $MgSO_4$ (0.22 g). This was followed by dropwise addition of TMSBr (0.11 mL, 0.86 mmol, 2 eq). After stirring the reaction mixture at rt for 1 h, the crude mixture was filtered over Celite and solvent was removed *in vacuo* to afford the desired product. Recrystallisation using Hexane:Et_2O (20:1) gave the desired product **17** as a brown solid (180 mg, 98% yield).

δ_H (500 MHz, $CDCl_3$): 7.19-7.09 (3H, m, ArH), 7.01-6.95 (4H, m, ArH), 6.94-6.81 (6H, m, ArH), 5.14 (1H, d, *J* = 8.7 Hz), 4.35 (1H, d, *J* = 8.7 Hz), 2.80-2.63 (2H, m, CH_2), 2.48 (2H, t, *J* = 7.9 Hz, CH_2), 2.17 (6H, s, Me).

δ_C (126 MHz, $CDCl_3$): 138.5, 137.5, 136.5, 135.8, 135.7, 127.6, 127.0, 127.0, 126.6, 126.5, 126.4, 126.0, 125.9, 124.3, 124.1, 123.4, 56.5, 56.0, 34.2, 31.7, 19.4, 19.3.

Characterisation data is in agreement with those previously reported:[42] ^{1}H NMR (500 MHz, $CDCl_3$) δ = 7.29–7.20 (m, 3H), 7.13–7.05 (m, 4H), 7.09–6.90 (m, 6H), 5.24 (d, *J* = 8.7 Hz, 1H), 4.45 (d, *J* = 8.7 Hz, 1H), 2.85-2.75 (m, 2H), 2.58 (t, *J* = 7.9 Hz, 2H), 2.26 (s, 6H), ^{13}C NMR (126 MHz, $CDCl_3$) δ = 140.4, 139.5, 138.5, 137.7, 137.6, 129.5, 129.0, 129.0, 128.5, 128.4, 128.4, 128.0, 127.9, 126.3, 126.0, 125.3, 58.4, 58.0, 36.1, 33.6, 21.3, 21.3.

rac-2-bromo-1,2-bis(3-methoxyphenyl)ethyl(phenethyl)sulfane 15

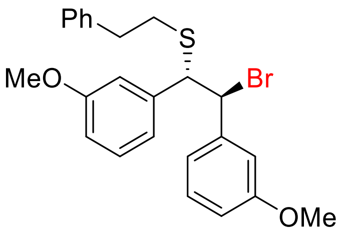

To a solution of *rac*-1,2-bis(3-methoxyphenyl)-2-(phenethylthio)ethan-1-ol (129 mg, 0.33 mmol) in DCM (0.33 mL) taken in a round bottom flask equipped with a stirring bar, was added anhydrous $MgSO_4$ (0.17 g). This was followed by dropwise addition of TMSBr (0.09 mL, 0.66 mmol, 2 eq). After stirring the reaction mixture at rt for 1 h, the crude mixture was filtered over celite and solvent was removed *in vacuo* to afford the desired product. Recrystallisation using Hexane:Et_2O (20:1) gave the desired product **15** as a yellow oil (98 mg, 65% yield).

δ_H (500 MHz, $CDCl_3$): 7.19-7.06 (3H, m, ArH), 7.05-6.94 (4H, m, ArH), 6.76-6.57 (6H, m, ArH), 5.11 (1H, d, *J* = 8.9 Hz), 4.33 (1H, d, *J* = 8.9 Hz), 3.64 (3H, s, Me), 3.63 (3H, s, Me), 2.80-2.65 (2H, m, CH_2), 2.56-2.44 (2H, m, CH_2).

δ_C (126 MHz, $CDCl_3$): 157.4, 157.1, 139.0, 138.4, 138.1, 127.2, 127.1, 126.6, 126.5, 124.4, 119.4, 118.7, 112.3, 112.1, 111.9, 111.4, 56.1, 55.9, 53.3, 53.2, 34.1, 31.7.

Characterisation data is in agreement with those previously reported:[42] ^{1}H NMR (400 MHz, $CDCl_3$) δ = 7.30–7.16 (m, 4H), 7.13–7.06 (m, 3H), 6.78 (d, *J* = 7.8 Hz, 1H), 6.75–6.67 (m, 5H), 5.19 (d, *J* = 8.8 Hz, 1H), 4.40 (d, *J* = 8.8 Hz, 1H), 3.70 (s, 3H), 3.69 (s, 3H), 2.89–2.69 (m, 2H), 2.64–2.49 (m, 2H), ^{13}C NMR (101 MHz, $CDCl_3$) δ = 159.3, 159.1, 140.9, 140.3, 140.1, 129.1, 129.1, 128.6, 128.4, 126.3, 121.4, 120.6, 114.3, 114.0, 113.9, 113.3, 58.1, 57.9, 55.2, 55.2, 36.0, 33.6.

rac-2-bromo-1,2-bis(4-chlorophenyl)ethyl(phenethyl)sulfane 19

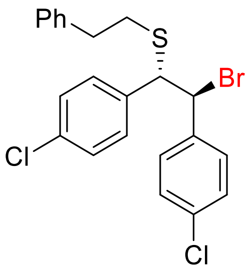

To a solution of rac-1,2-bis(4-chlorophenyl)-2-(phenethylthio)ethan-1-ol (655 mg, 1.62 mmol) in DCM (2.0 mL) taken in a round bottom flask equipped with a stirring bar, was added anhydrous $MgSO_4$ (0.81 g). This was followed by dropwise addition of TMSBr (0.42 mL, 3.25 mmol, 2 eq). After stirring the reaction mixture at rt for 1 h, the crude mixture was filtered over celite and solvent was removed *in vacuo* to afford the desired product. Recrystallisation using Hexane:Et_2O (20:1) gave the desired product **19** as a yellow oil (588 mg, 78% yield).

δ_H (500 MHz, $CDCl_3$): 7.24-6.90 (13H, m, ArH), 5.06 (1H, d, *J* = 8.3 Hz), 4.24 (1H, d, *J* = 8.3 Hz), 2.79-2.67 (2H, m, CH_2), 2.56-2.42 (2H, m, CH_2).

δ_C (126 MHz, $CDCl_3$): 138.1, 135.5, 134.7, 132.2, 131.6, 128.3, 127.8, 126.6, 126.6, 126.5, 126.4, 124.5, 55.4, 54.3, 34.0, 31.7.

Characterisation data is in agreement with those previously reported:[42] ^{1}H NMR (400 MHz, $CDCl_3$) δ = 7.30–6.99 (m, 13H), 5.13 (d, *J* = 8.3 Hz, 1H), 4.31 (d, *J* = 8.2 Hz, 1H), 2.80 (dd, *J* = 8.4, 5.6 Hz, 2H), 2.56 (t, *J* = 7.7 Hz, 2H), ^{13}C NMR (101 MHz, $CDCl_3$) δ = 140.0, 137.4, 136.6, 134.2, 133.6, 130.3, 129.7, 128.5, 128.5, 128.4, 128.3, 126.5, 57.3, 56.3, 36.0, 33.6.

rac-2-fluoro-1,2-diphenylethyl)(phenethyl)sulfane 11

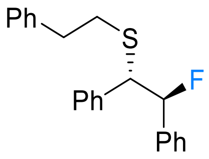

11

In a 4-dram glass vial (2.1 × 7 cm, 14 mL) equipped with a cross-bar stirrer (1 cm × 1 cm) were sequentially added substrate **10** (60 mg, 0.15 mmol), catalyst **14** (22 mg, 0.05 mmol, 0.3 eq), DCE (0.1 mL), pre-stirred Span-80 surfactant solution (18 mg in 0.9 mL deionised H_2O, 2% w/w) and $KF{\cdot}2H_2O$ (86 mg, 0.91 mmol, 6 eq.). The reaction vial was sealed with a lid (PTFE septum) and was stirred for 4h on a custom-made aluminium block with 1 inch off-set stirring at 500 rpm, 50 °C. The crude reaction mixture was diluted with 9 mL deionised H_2O, and the aqueous phase was extracted three times with 10 mL DCM. The organic layers were combined, dried ($MgSO_4$), filtered and concentrated *in vacuo*. The crude mixture was dissolved in $CDCl_3$ and analysed by ^{1}H and ^{19}F NMR.

Purification via flash column chromatography on silica gel (Hexane/EtOAc = 99:1 to 95:5 gradient), gave **11** (24 mg, 47% yield) as a crude colourless oil consisting of an inseparable mixture of the desired fluorinated product **11** (major) and alkene by-product **13** in ratio **11**:**13** = 17:10.

δ_H (400 MHz, $CDCl_3$): 7.30-6.93 (15H, m, ArH), 5.58 (1H, dd, *J* = 46.3, 7.5 Hz), 4.13 (1H, dd, *J* = 13.6, 7.5 Hz), 2.82-2.55 (4H, m, CH_2).

δ_C (126 MHz, $CDCl_3$): 140.4, 137.7 (d, $J_{C\text{-}F}$ = 4.7 Hz), 137.6 (d, $J_{C\text{-}F}$ = 20.9 Hz), 129.0, 128.6, 128.4, 128.4, 128.0, 127.7, 126.3 (d, $J_{C\text{-}F}$ = 1.3 Hz), 126.3, 97.0 (d, $J_{C\text{-}F}$ = 180.3 Hz), 56.3 (d, $J_{C\text{-}F}$ = 24.0 Hz), 36.2, 33.3.

δ_F (376 MHz, $CDCl_3$): -172.5 (1F).

Characterisation data is in agreement with those previously reported:[42] ^{1}H NMR (400 MHz, $CDCl_3$) δ = 7.30–7.19 (m, 9H), 7.17–7.14 (m, 2H), 7.11–7.09 (m, 4H), 5.67 (dd, *J* = 46.3, 7.5 Hz, 1H), 4.22 (dd, *J* = 13.6, 7.5 Hz, 1H), 2.88–2.62 (m, 4H), ^{19}F NMR (376 MHz, $CDCl_3$) δ = -172.6 (dd, *J* = 46.3, 13.6 Hz, 1F), ^{13}C NMR (101 MHz, $CDCl_3$) δ = 140.5, 137.8 (d, $J_{C–F}$ = 4.8 Hz), 137.6 (d, $J_{C–F}$ = 21.1 Hz), 129.1, 128.7, 128.6 (d, $J_{C–F}$ = 2.4 Hz), 128.5, 128.5, 128.1, 127.8, 126.4 (d, $J_{C–F}$ = 7.0 Hz), 126.4, 97.1 (d, $J_{C–F}$ = 180.3 Hz), 56.4 (d, $J_{C–F}$ = 23.9 Hz), 36.5, 33.4.

Compound 18

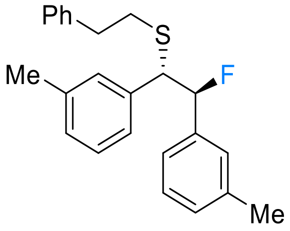

In a 4-dram glass vial (2.1 × 7 cm, 14 mL) equipped with a cross-bar stirrer (1 cm × 1 cm) were sequentially added substrate **17** (65 mg, 0.15 mmol), catalyst **14** (22 mg, 0.05 mmol, 0.3 eq), DCE (0.1 mL), pre-stirred Span-80 surfactant solution (18 mg in 0.9 mL deionised H_2O, 2% w/w) and $KF{\cdot}2H_2O$ (86 mg, 0.91 mmol, 6 eq.). The reaction vial was sealed with a lid (PTFE septum) and was stirred for 4h on a custom-made aluminium block with 1 inch off-set stirring at 500 rpm, 50 °C. The crude reaction mixture was diluted with 9 mL deionised H_2O, and the aqueous phase was extracted three times with 10 mL DCM. The organic layers were combined, dried ($MgSO_4$), filtered and concentrated *in vacuo*. The crude mixture was dissolved in $CDCl_3$ and analysed by ^{1}H and ^{19}F NMR.

Purification via flash column chromatography on silica gel (Hexane/EtOAc = 99:1 to 95:5 gradient), gave **18** (25 mg, 46% yield) as a crude colourless oil consisting of an inseparable mixture of the desired fluorinated product **18** (major) and corresponding alkene by-product in ratio **18**:alkene = 24:10.

Δ_H (400 MHz, $CDCl_3$): 7.28-6.76 (13H, m, ArH), 5.55 (1H, dd, *J* = 46.4, 7.2 Hz), 4.07 (1H, dd, *J* = 14.5, 7.2 Hz), 2.80-2.66 (2H, m, CH_2), 2.65-2.53 (2H, m, CH_2), 2.19 (6H, s, CH_3).

Δ_C (126 MHz, $CDCl_3$): 140.5, 138.0, 137.8 (d, J_{C-F} = 4.4 Hz), 137.6, 137.5, 137.4, 129.6, 129.2, 128.6, 128.4, 128.2, 126.9 (d, J_{C-F} = 6.9 Hz), 126.3, 126.0, 123.4 (d, J_{C-F} = 6.9 Hz), 97.0 (d, J_{C-F} = 180.0 Hz), 56.2 (d, J_{C-F} = 23.7 Hz), 36.2, 33.3 (d, J_{C-F} = 2.1 Hz).

Δ_F (376 MHz, $CDCl_3$): -173.3 (1F).

Characterisation data is in agreement with those previously reported:[42] ^{1}H NMR (500 MHz, $CDCl_3$) δ = 7.34–7.20 (m, 3H), 7.19–6.88 (m, 10H), 5.66 (dd, J = 46.5, 7.2 Hz, 1H), 4.19 (dd, J = 14.5, 7.2 Hz, 1H), 2.92–2.76 (m, 2H), 2.71 (m, 2H), 2.31 (s, 3H), 2.31 (s, 3H), ^{19}F NMR (470 MHz, $CDCl_3$) δ = -173.4 (dd, J = 46.4, 14.5 Hz, 1F), ^{13}C NMR (126 MHz, $CDCl_3$) [overlapping signals] δ = 140.5, 138.0, 137.8 ($J_{C–F}$ = 4.3 Hz), 137.6, 137.6 ($J_{C–F}$ = 20.7 Hz), 129.5, 129.2, 129.1, 128.5, 128.4, 128.2, 127.8, 126.9, ($J_{C–F}$ = 6.8 Hz), 126.3, 126.0, 123.4 ($J_{C–F}$ = 6.9 Hz), 97.0 ($J_{C–F}$ = 180.0 Hz), 56.2 ($J_{C–F}$ = 23.7 Hz), 36.2, 33.3 ($J_{C–F}$ = 2.1 Hz), 21.4.

Compound 16

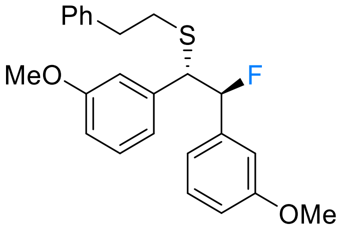

In a 4-dram glass vial (2.1 × 7 cm, 14 mL) equipped with a cross-bar stirrer (1 cm × 1 cm) were sequentially added substrate **15** (28.6 mg, 0.06 mmol), catalyst **14** (9.1 mg, 0.02 mmol, 0.3 eq), DCE (0.04 mL), pre-stirred Span-80 surfactant solution (7.4 mg in 0.37 mL deionised H_2O, 2% w/w) and $KF·2H_2O$ (35 mg, 0.375 mmol, 6 eq.). The reaction vial was sealed with a lid (PTFE septum) and was stirred for 4h on a custom-made aluminium block with 1 inch off-set stirring at 500 rpm, 50 °C. The crude reaction mixture was diluted with 9 mL deionised H_2O, and the aqueous phase was extracted three times with 10 mL DCM. The organic layers were combined, dried ($MgSO_4$), filtered and concentrated *in vacuo*. The crude mixture was dissolved in $CDCl_3$ and analysed by ^{1}H and ^{19}F NMR.

Purification via flash column chromatography on silica gel (Hexane/EtOAc = 99:1 to 95:5 gradient), gave **16** (20 mg, 33% yield) as a pure colourless oil.

δ_H (400 MHz, $CDCl_3$): 7.21-6.99 (7H, m), 6.74-6.54 (6H, m), 5.55 (1H, dd, *J* 46.35, 7.27), 4.08 (1H, dd, *J* 14.20, 7.12), 3.64 (3H, s), 3.63 (3H, s), 2.80-2.67 (2H, m, CH_2), 2.64-2.58 (2H, m, CH_2).

δ_C (126 MHz, $CDCl_3$): 159.5, 159.2, 140.4, 139.3 (d, J_{C-F} 4.45), 139.1, 139.0, 129.3, 129.0, 128.5, 128.4, 126.3, 121.4, 118.6 (d, J_{C-F} 7.03), 114.3, 113.4, 111.6 (d, J_{C-F} 7.45), 96.7 (d, J_{C-F} 181.0), 56.2 (d, J_{C-F} 14.39), 55.2, 36.2, 33.3 (d, J_{C-F} 2.0).

δ_F (376 MHz, $CDCl_3$): -173.1 (1F).

Characterisation data is in agreement with those previously reported:[42] ^{1}H NMR (500 MHz, $CDCl_3$) δ = 7.32–7.09 (m, 7H), 6.84– 6.64 (m, 6H), 5.66 (dd, J = 46.4, 7.2 Hz, 1H), 4.18 (dd, J = 14.1, 7.2 Hz, 1H), 3.75 (s, 3H),

3.73 (s, 3H), 2.89–2.77 (m, 2H), 2.76–2.68 (m, 2H), ^{19}F NMR (470 MHz, $CDCl_3$) δ = -173.1 (dd, J = 46.4, 14.1 Hz, 1F), ^{13}C NMR (126 MHz, $CDCl_3$) [overlapping signals] δ = 159.5, 159.2, 140.4, 139.3 (d, $J_{C–F}$ = 4.3 Hz), 139.0 (d, $J_{C–F}$ = 21.0 Hz), 129.3, 129.0, 128.5, 128.4, 126.3, 121.4, 118.6 (d, $J_{C–F}$ = 7.0 Hz), 114.3 (d, $J_{C–F}$ = 2.9 Hz), 114.3, 113.4, 111.6 (d, $J_{C–F}$ = 7.5 Hz), 96.7 (d, $J_{C–F}$ = 181.0 Hz), 56.2 (d, $J_{C–F}$ = 23.8 Hz), 55.2, 36.2, 33.3 (d, $J_{C–F}$ = 2.0 Hz).

Compound 20

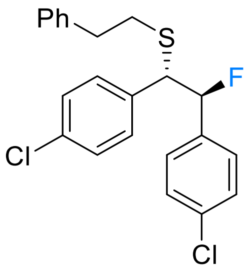

In a 4-dram glass vial (2.1 × 7 cm, 14 mL) equipped with a cross-bar stirrer (1 cm × 1 cm) were sequentially added substrate **19** (71 mg, 0.15 mmol), catalyst **14** (22 mg, 0.05 mmol, 0.3 eq), DCE (0.1 mL), pre-stirred Span-80 surfactant solution (18 mg in 0.9 mL deionised H_2O, 2% w/w) and $KF{\cdot}2H_2O$ (86 mg, 0.91 mmol, 6 eq.). The reaction vial was sealed with a lid (PTFE septum) and was stirred for 4h on a custom-made aluminium block with 1 inch off-set stirring at 500 rpm, 50 °C. The crude reaction mixture was diluted with 9 mL deionised H_2O, and the aqueous phase was extracted three times with 10 mL DCM. The organic layers were combined, dried ($MgSO_4$), filtered and concentrated *in vacuo*. The crude mixture was dissolved in $CDCl_3$ and analysed by ^{1}H and ^{19}F NMR.

Purification via flash column chromatography on silica gel (Hexane/EtOAc = 99:1 to 95:5 gradient), gave **20** (20 mg, 33% yield) as a crude colourless oil consisting of an inseparable mixture of the desired fluorinated product **20** and corresponding alkene by-product in ratio **20**:alkene = 10:13.

δ_H (400 MHz, $CDCl_3$): 7.23-7.04 (7H, m, ArH), 7.01 (2H, d, *J* = 7.2 Hz, ArH), 6.95 (2H, d, *J* = 8.4 Hz, ArH), 6.90 (2H, d, *J* = 8.4 Hz, ArH), 5.51 (1H, dd, *J* = 45.8, 7.0 Hz), 4.07 (1H, dd, *J* = 13.8, 7.0 Hz), 2.77-2.70 (2H, m, CH_2), 2.66-2.53 (2H, m, CH_2).

δ_C (126 MHz, $CDCl_3$): 140.1, 135.8 (d, $J_{C\text{-}F}$ = 4.1 Hz), 135.5 (d, $J_{C\text{-}F}$ = 21.4 Hz), 134.5 (d, $J_{C\text{-}F}$ = 1.9 Hz), 133.7, 130.3, 128.6, 128.6, 128.5, 128.3, 127.7 (d, $J_{C\text{-}F}$ = 6.9 Hz), 126.5, 95.8 (d, $J_{C\text{-}F}$ = 181.7 Hz), 55.3 (d, $J_{C\text{-}F}$ = 24.5 Hz), 36.1, 33.3 (d, $J_{C\text{-}F}$ = 1.7 Hz).

δ_F (376 MHz, $CDCl_3$): -172.4.

Characterisation data is in agreement with those previously reported:[42] ^{1}H NMR (400 MHz, $CDCl_3$) δ = 7.32–7.15 (m, 7H), 7.10–7.05 (m, 2H), 7.02 (d, J = 8.4 Hz, 2H), 6.97 (d, J = 8.4 Hz, 2H), 5.58 (dd, J = 45.8, 7.0 Hz, 1H), 4.09 (dd, J = 13.8, 7.0 Hz, 1H), 2.90–2.76 (m, 2H), 2.73–2.56 (m, 2H), ^{19}F NMR (376 MHz, $CDCl_3$) δ = -172.4 (dd, J = 45.9, 13.9 Hz, 1F), ^{13}C NMR (101 MHz, $CDCl_3$) δ = 140.2, 135.9 (d, $J_{C–F}$ = 4.0 Hz), 135.6 (d,

J_{C-F} = 21.4 Hz), 134.7 (d, J_{C-F} = 1.9 Hz), 133.8, 130.4, 128.7, 128.7, 128.6, 128.4, 127.8 (d, J_{C-F} = 6.9 Hz), 126.6, 95.9 (d, J_{C-F} = 181.7 Hz), 55.4 (d, J_{C-F} = 24.4 Hz), 36.2, 33.4 (d, J_{C-F} = 1.8 Hz).

5.1.4 Results of screening with *surfactant_map*

The results, represented as the 11:12 ratio, is summarised below:

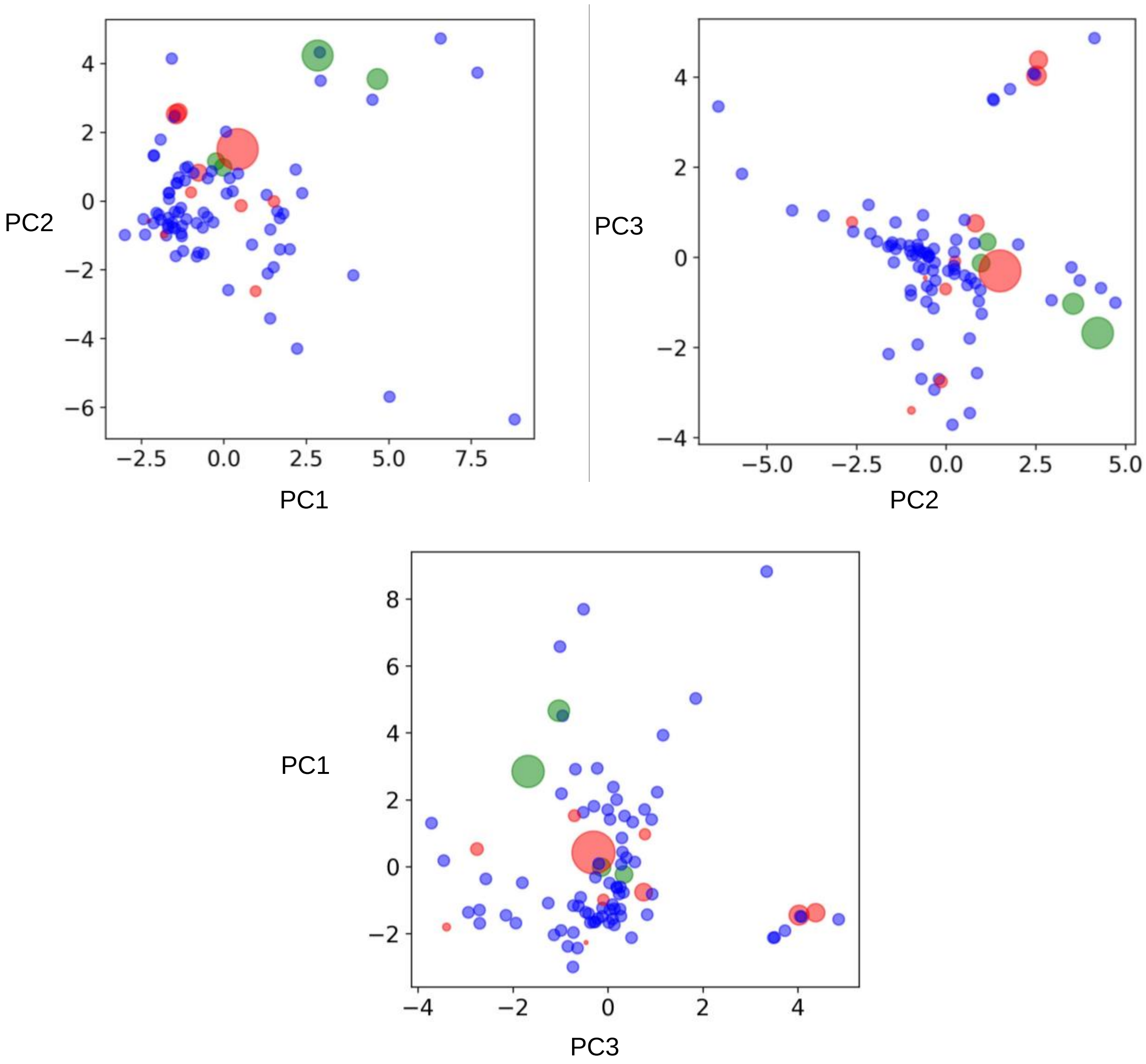

Figure S54. Projected ratios of 11:12 (dot size) for each screened surfactant (red for ***screen1*** and green for ***screen2***) in PC1-PC2, PC2-PC3 and PC3-PC1 combinations

Table S18. Surfactant screen for fluorination of β-bromosulfide **10**

No.	Solvent	Fluoride	Surfactant	Yield (% **10**:**11**:**12**:**13**)[a]	Combined yield **11** + **13** (%)
1[b,c]	Toluene:H_2O (10:90)	CsF	None	81:**6**:7:6	12
2[b,c]	DCE:H_2O (10:90)	CsF	None	69:**10**:9:12	22
3	DCE:H_2O (10:90)	CsF	Span 20	0:**39**:33:28	67
4	DCE:H_2O (10:90)	CsF	SPGS-550-M	0:**27**:45:28	55
5	DCE:H_2O (10:90)	CsF	Sodium 1-decanesulfonate	0:**0**:90:10	10

6	DCE:H_2O (10:90)	CsF	CPC	0:**27**:21:51	78
7	DCE:H_2O (10:90)	CsF	Lauryl Betaine	0:**12**:53:35	47
8	DCE:H_2O (10:90)	CsF	CTAC	0:**32**:21:47	79
9	DCE:H_2O (10:90)	CsF	Span 80	0:**57**:8:35	92
10	DCE:H_2O (10:90)	CsF	Brij 52	0:**19**:39:42	61
11	DCE:H_2O (10:90)	CsF	PS-750-M	0:**25**:47:28	53
12[d]	DCE:H_2O (10:90)	CsF	Span 80	0:**56**:8:36	92
13	DCE:H_2O (10:90)	CsF	Span 85	0:**52**:13:35	87
14	DCE:H_2O (10:90)	CsF	Span 40	0:**38**:32:30	68
15	DCE:H_2O (10:90)	CsF	Span 60	0:**39**:31:30	69
16	DCE:H_2O (10:90)	CsF	Tween 85	0:**44**:25:31	75
17	DCE:H_2O (10:90)	CsF	Brij 35	0:**20**:49:31	51
18	DCE:H_2O (10:90)	KF.2H_2O	Span 80	0:**49**:10:41	90
19[e]	DCE:H_2O (10:90)	KF.2H_2O	Span 80	0:**58**:13:29	87
20	Toluene:H_2O (10:90)	KF.2H_2O	Span 80	0:**40**:28:32	72
21[c]	DCE:H_2O (10:90)	TBAF	None	75:**0**:6:18	18
22[e]	DCE:H_2O (10:90)	KF.2H_2O	Dibenzo-18-crown-6	18:**16**:50:16	32

Standard conditions [SM] = 0.15M, 3.0 eq. of fluoride, 30 mol% of catalyst **14**, 2% w/w surfactant in water, 19h at 50 °C; [a]Determined by ^1H NMR; [b][SM] = 0.08M; [c]at room temperature; [d][SM] = 0.23M; [e]6.0 eq. of KF.2H_2O.

5.2 Fluorination of sulfonyl chlorides

5.2.1 Standard reaction protocol

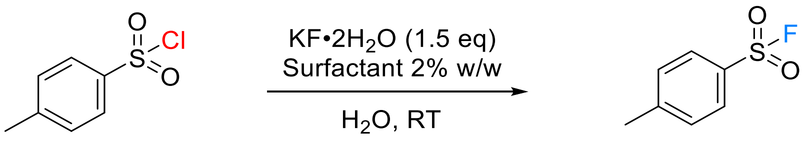

21 **22**

For reaction optimisation

In a 4-dram glass vial (2.1 × 7 cm, 14 mL) equipped with a cross-bar stirrer (1 cm × 1 cm) were sequentially added the appropriate sulfonyl chloride substrate (1.06 mmol), KF•2H_2O (1.5 eq.), and pre-stirred CTAC surfactant solution (2% w/w) in deionised H_2O (7 mL, 0.152 M in sulfonyl chloride). The reaction vial was sealed with a lid (PTFE septum) and was stirred on a custom-made aluminium block with 1 inch off-set stirring at an appropriate stirring speed, temperature, and duration (detailed in the optimisation table). The crude reaction mixture was diluted with DCM and deionised H_2O, the layers were partitioned utilising brine, and the aqueous phase was extracted three times with DCM. The organic layers were combined, dried ($MgSO_4$), filtered and concentrated *in vacuo*. The crude mixture was dissolved in $CDCl_3$ and analysed by ^1H and ^{19}F NMR.

For substrate scope experiments

In a 4-dram glass vial (2.1 × 7 cm, 14 mL) equipped with a cross-bar stirrer (1 cm × 1 cm) were sequentially added the appropriate sulfonyl chloride substrate (1.06 mmol), $KF{\bullet}2H_2O$ (3.19 mmol, 3 eq.), and pre-stirred CTAC surfactant solution (2% w/w) in deionised H_2O (7 mL, 0.152 M in sulfonyl chloride). The reaction vial was sealed with a lid (PTFE septum) and was stirred for 3 hours at room temperature on a custom-made aluminium block with 1 inch off-set stirring at 500 rpm. In case where the product was solid simply a filtration of the reaction mixture was carried out. Product was washed with water (10 mL) 4-5 times to remove residual surfactant CTAC, dried overnight in a desiccator and then analysed by ^{1}H, ^{13}C and ^{19}F NMR. In case where the product was liquid- the crude reaction mixture was diluted with DCM and deionised H_2O, the layers were partitioned utilising brine, and the aqueous phase was extracted three times with DCM. The organic layers were combined, dried ($MgSO_4$), filtered, and concentrated *in vacuo* after passing the solution through a plug of silica. The isolated product was dissolved in $CDCl_3$ and analysed by ^{1}H, ^{13}C and ^{19}F NMR.

5.2.2 Determination of reaction yields

Example .No. 2 from Table 3 in the manuscript (Span 60)

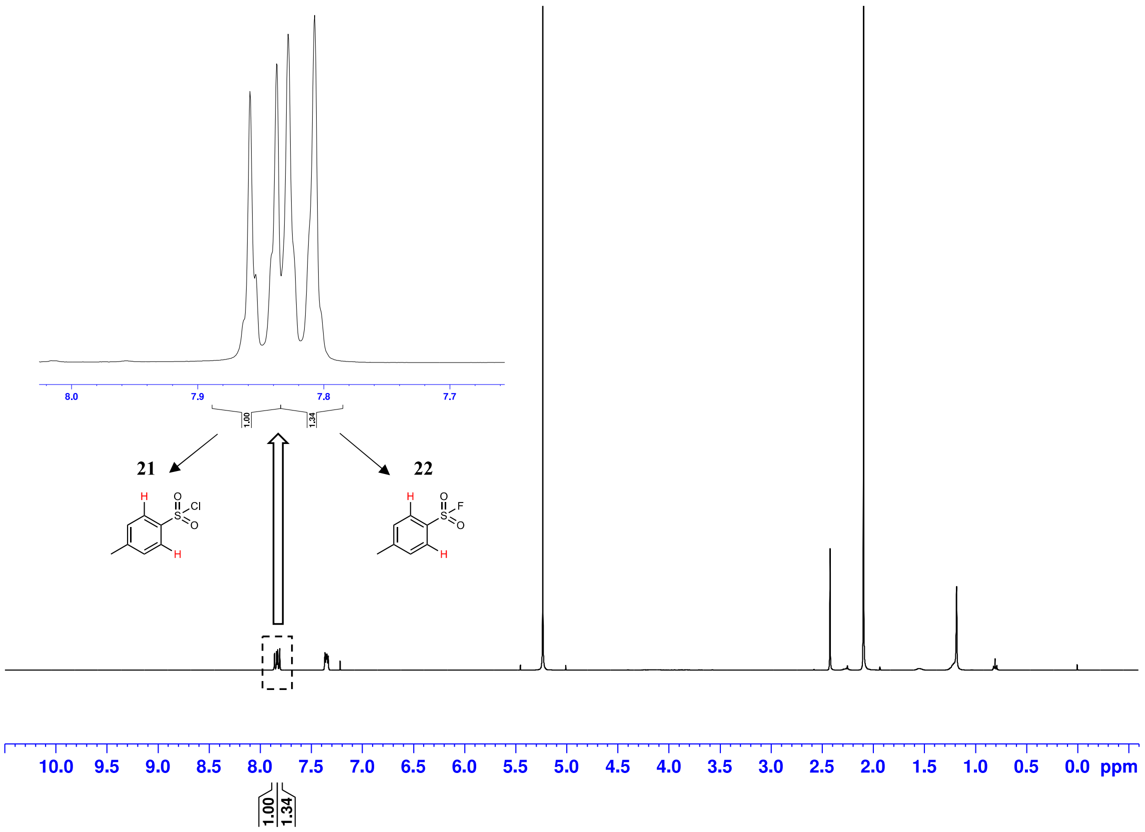

Figure S55. Determination of reaction yield with ^{1}H NMR

Conversion calculation using ^{1}H NMR integration of CH peaks

% Conversion product **22** = $\frac{\mathbf{22}}{\mathbf{21}+\mathbf{22}} * 100 = \frac{1.34}{1.0+1.34} * 100$ = 57%

5.2.3 Characterisation data of products

p-Tolylsulfonyl fluoride 22

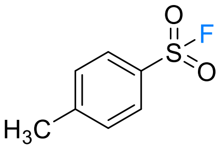

In a 4-dram glass vial (2.1 × 7 cm, 14 mL) equipped with a cross-bar stirrer (1 cm × 1 cm) were sequentially added *p*-toluenesulfonyl chloride (203 mg, 1.06 mmol), pre-stirred CTAC surfactant solution (126 mg, 2% w/w) in deionised H_2O (7 mL, 0.152 M), and KF•2H_2O (300 mg, 3.19 mmol, 3 eq). The reaction vial was sealed with a lid (PTFE septum) and was stirred for 3 hours at room temperature on a custom-made aluminium

block with 1 inch off-set stirring at 500 rpm. Filtration of the reaction mixture was carried out and the filtered precipitate was washed with water (10 mL) 4-5 times to remove residual surfactant CTAC, dried overnight in a desiccator to afford the desired product **22** as white solid (172 mg, 93% yield). Product was analysed by ^{1}H, ^{13}C and ^{19}F NMR.

δ_H (500 MHz, $CDCl_3$): 7.79 (2H, d, *J* = 8.4 Hz, ArH), 7.33 (2H, d, *J* = 4.3 Hz, ArH), 2.40 (3H, s, Me).

δ_C (126 MHz, $CDCl_3$): 147.2, 130.3, 130.0 (d, *J* = 24.3 Hz), 128.4, 21.8 (CH_3).

δ_F (376 MHz, $CDCl_3$): 66.2.

Characterisation data is in agreement with those previously reported:[43] ^{1}H NMR (400 MHz, $CDCl_3$): δ [ppm] = 7.89 (d, 8.0 Hz, 2H), 7.42 (d, 8.0 Hz, 2H), 2.49 (s, 3H); ^{13}C NMR (100 MHz, $CDCl_3$): δ [ppm] = 147.2, 130.4, 130.3, 130.1, 128.6, 22.0; ^{19}F NMR (376 MHz, $CDCl_3$): δ [ppm] = 66.2.

o-Tolylsufonyl fluoride 32

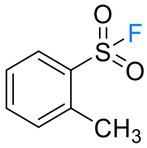

In a 4-dram glass vial (2.1 × 7 cm, 14 mL) equipped with a cross-bar stirrer (1 cm × 1 cm) were sequentially added *o*-toluenesulfonyl chloride (203 mg, 1.06 mmol), pre-stirred CTAC surfactant solution (126 mg, 2% w/w) in deionised H_2O (7 mL, 0.152 M), and $KF•2H_2O$ (300 mg, 3.19 mmol, 3 eq). The reaction vial was sealed with a lid (PTFE septum) and was stirred for 3 hours at room temperature on a custom-made aluminium block with 1 inch off-set stirring at 500 rpm. The crude reaction mixture was diluted with DCM and deionised H_2O, the layers were partitioned utilising brine, and the aqueous phase was extracted three times with DCM. The organic layers were combined, dried ($MgSO_4$), filtered, and concentrated *in vacuo* after passing the solution through a plug of silica to afford the desired product **32** as colourless oil (165 mg, 89% yield). Product was analysed by ^{1}H, ^{13}C and ^{19}F NMR.

δ_H (500 MHz, $CDCl_3$): 7.90 (1H, d, *J* = 8.1 Hz, ArH), 7.52 (1H, t, *J* = 4.3 Hz, ArH), 7.35-7.25 (2H, m, ArH), 2.57 (3H, s, Me).

δ_C (126 MHz, $CDCl_3$): 139.0, 135.4, 132.9, 132.2 (d, *J* = 22.2 Hz), 130.3, 126.7, 20.2 (CH_3).

δ_F (376 MHz, $CDCl_3$): 60.3.

Characterisation data is in agreement with those previously reported:[44] ^{1}H NMR (399 MHz, $CDCl_3$) δ 8.02 (d, *J* = 7.9 Hz, 1H), 7.63 (t, *J* = 7.6 Hz, 1H), 7.48 –7.37 (m, 2H), 2.69 (s, 3H). $^{13}C\{1H\}$ NMR (100 MHz, $CDCl_3$) δ 139.1, 135.4, 133.0, 132.4 (d, *J* = 22.1 Hz), 130.1 (d, *J* = 1.8 Hz), 126.8, 20.3 (d, *J* = 1.3 Hz). ^{19}F NMR (376 MHz, $CDCl_3$) δ 60.31 (s).

p-Bromophenylsulfonyl fluoride 24

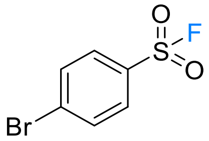

In a 4-dram glass vial (2.1 × 7 cm, 14 mL) equipped with a cross-bar stirrer (1 cm × 1 cm) were sequentially added *p*-bromobenzene sulfonyl chloride (272 mg, 1.06 mmol), pre-stirred CTAC surfactant solution (126 mg, 2% w/w) in deionised H_2O (7 mL, 0.152 M), and KF•2H_2O (300 mg, 3.19 mmol, 3 eq). The reaction vial was sealed with a lid (PTFE septum) and was stirred for 3 hours at room temperature on a custom-made aluminium block with 1 inch off-set stirring at 500 rpm. Filtration of the reaction mixture was carried out and the filtered precipitate was washed with water (10 mL) 4-5 times to remove residual surfactant CTAC, dried overnight in a desiccator to afford the desired product **24** as white solid (223 mg, 88% yield). Product was analysed by ^{1}H, ^{13}C and ^{19}F NMR.

δ_H (500 MHz, $CDCl_3$): 7.79 (2H, d, *J* = 8.8 Hz, ArH), 7.70 (2H, t, *J* = 8.8 Hz, ArH).

δ_C (126 MHz, $CDCl_3$): 133.1, 131.9 (d, *J* = 25.7 Hz), 131.3, 129.8.

δ_F (376 MHz, $CDCl_3$): 66.4.

Characterisation data is in agreement with those previously reported:[44] ^{1}H NMR (399 MHz, $CDCl_3$) δ 7.87 (d, *J* = 8.7 Hz, 2H), 7.78 (d, *J* = 8.4 Hz, 2H). ^{13}C NMR (100 MHz, $CDCl_3$) δ 133.2, 132.0 (d, *J* = 25.7 Hz), 131.4, 129.9. ^{19}F NMR (376 MHz, $CDCl_3$) δ 66.36 (s).

o-Bromobenzenesulfonyl fluoride 34

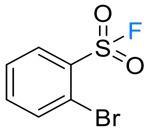

In a 4-dram glass vial (2.1 × 7 cm, 14 mL) equipped with a cross-bar stirrer (1 cm × 1 cm) were sequentially added *o*-bromobenzenesulfonyl chloride (272 mg, 1.06 mmol), pre-stirred CTAC surfactant solution (126 mg, 2% w/w) in deionised H_2O (7 mL, 0.152 M), and KF•2H_2O (300 mg, 3.19 mmol, 3 eq). The reaction vial was sealed with a lid (PTFE septum) and was stirred for 3 hours at room temperature on a custom-made aluminium block with 1 inch off-set stirring at 500 rpm. Filtration of the reaction mixture was carried out and the filtered precipitate was washed with water (10 mL) 4-5 times to remove residual surfactant CTAC, dried overnight in a desiccator to afford the desired product **34** as white solid (234 mg, 92% yield). Product was analysed by ^{1}H, ^{13}C and ^{19}F NMR.

δ_H (500 MHz, $CDCl_3$): 8.15 (1H, dd, *J* = 7.6, 2.0 Hz, ArH), 7.87 (1H, dd, *J* = 7.6, 1.6 Hz, ArH), 7.65-7.54 (2H, m, ArH).

δ_C (126 MHz, $CDCl_3$): 136.2, 135.9, 133.9 (d, *J* = 24.2 Hz), 132.1 (d, *J* = 1.5 Hz), 128.0, 121.1 (d, *J* = 1.1 Hz).

δ_F (376 MHz, $CDCl_3$): 57.9.

Characterisation data is in agreement with those previously reported:[45] ^{1}H NMR (400 MHz, $CDCl_3$): δ 8.17 – 8.12 (m, 1H), 7.87 – 7.84 (m, 1H), 7.61 – 7.53 (m, 2H). ^{13}C NMR (100 MHz, $CDCl_3$): δ 136.2, 136.0, 134.1 (d, *J* = 24.3 Hz), 132.2 (d, *J* = 1.7 Hz), 128.1, 121.2. ^{19}F NMR (376 MHz, $CDCl_3$): δ 57.94.

***p*-Nitrophenylsulfonyl fluoride 36**

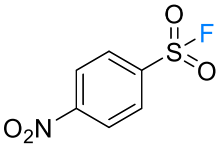

In a 4-dram glass vial (2.1 × 7 cm, 14 mL) equipped with a cross-bar stirrer (1 cm × 1 cm) were sequentially added *p*-nitrobenzenesulfonyl chloride (236 mg, 1.06 mmol), pre-stirred CTAC surfactant solution (126 mg, 2% w/w) in deionised H_2O (7 mL, 0.152 M), and KF•$2H_2O$ (300 mg, 3.19 mmol, 3 eq.). The reaction vial was sealed with a lid (PTFE septum) and was stirred for 3 hours at room temperature on a custom-made aluminium block with 1 inch off-set stirring at 500 rpm. Filtration of the reaction mixture was carried out and the filtered precipitate was washed with water (10 mL) 4-5 times to remove residual surfactant CTAC, dried overnight in a desiccator to afford the desired product **36** as white solid (180 mg, 82% yield). Product was analysed by ^{1}H, ^{13}C and ^{19}F NMR.

δ_H (500 MHz, $CDCl_3$): 8.51 (2H, d, *J* = 8.0 Hz, ArH), 8.26 (2H, d, *J* = 7.8 Hz, ArH).

δ_C (126 MHz, $CDCl_3$): 151.8, 138.2 (d, *J* = 26.7 Hz), 130.0, 124.9.

δ_F (376 MHz, $CDCl_3$): 66.1.

Characterisation data is in agreement with those previously reported:[46] ^{1}H NMR (400 MHz, $CDCl_3$): δ 8.49 (d, *J* = 8.9 Hz, 2H), 8.25(d, *J* = 8.9 Hz, 2H); ^{19}F NMR (376 MHz, $CDCl_3$): δ 66.1 (s, 1F); ^{13}C NMR(101 MHz, $CDCl_3$): δ 152.0, 138.5 (d, *J* = 26.9 Hz), 130.1, 125.0 ppm.

^{1}H NMR (400 MHz, $CDCl_3$): δ [ppm] = 8.49 (d, 8.4 Hz, 2H), 8.25 (d, 8.4 Hz, 2H); ^{13}C NMR (100 MHz, $CDCl_3$): δ [ppm] = 142.8, 131.7, 131.4, 130.3, 130.0; ^{19}F NMR (376 MHz, $CDCl_3$): δ [ppm] = 66.3.

***o*-Nitrophenylsulfonyl fluoride 26**

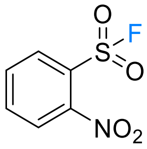

In a 4-dram glass vial (2.1 × 7 cm, 14 mL) equipped with a cross-bar stirrer (1 cm × 1 cm) were sequentially added *o*-nitrobenzenesulfonyl chloride (236 mg, 1.06 mmol), pre-stirred CTAC surfactant solution (126 mg, 2% w/w) in deionised H_2O (7 mL, 0.152 M), and KF•$2H_2O$ (300 mg, 3.19 mmol, 3 eq). The reaction vial was sealed with a lid (PTFE septum) and was stirred for 3 hours at room temperature on a custom-made aluminium block with 1 inch off-set stirring at 500 rpm. Filtration of the reaction mixture was carried out and the filtered precipitate was washed with water (10 mL) 4-5 times to remove residual surfactant CTAC, dried overnight in

a desiccator to afford the desired product **26** as white solid (183 mg, 84% yield). Product was analysed by ^{1}H, ^{13}C and ^{19}F NMR.

δ_H (500 MHz, $CDCl_3$): 8.32-8.22 (1H, m, ArH), 8.07 (1H, d, *J* = 8.1 Hz, ArH), 8.04-7.96 (1H, m, ArH), 7.96-7.87 (1H, m, ArH).

δ_C (126 MHz, $CDCl_3$):148.1, 136.8, 133.5, 131.8, 130.4, 125.9.

δ_F (376 MHz, $CDCl_3$): 65.0.

Characterisation data is in agreement with those previously reported:[47] ^{1}H NMR (500 MHz, $CDCl_3$) δ 8.26 (dd, *J* = 7.9, 1.3 Hz, 1H), 8.06 (d, *J* = 7.9 Hz, 1H), 7.97 (td, *J* = 7.8, 1.4 Hz, 1H), 7.93–7.85 (m, 1H). ^{13}C NMR (101 MHz, $CDCl_3$) δ 148.3, 136.7, 133.4, 132.0 (d, *J* = 1.6 Hz), 127.2 (d, *J* = 29.1 Hz), 126.0. ^{19}F NMR (470 MHz, $CDCl_3$) δ 65.1.

p-Iodobenzenesulfonyl fluoride 28

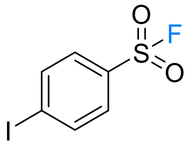

In a 4-dram glass vial (2.1 × 7 cm, 14 mL) equipped with a cross-bar stirrer (1 cm × 1 cm) were sequentially added *p*-iodobenzenesulfonyl chloride (322 mg, 1.06 mmol), pre-stirred CTAC surfactant solution (126 mg, 2% w/w) in deionised H_2O (7 mL, 0.152 M), and KF•2H_2O (300 mg, 3.19 mmol, 3 eq). The reaction vial was sealed with a lid (PTFE septum) and was stirred for 3 hours at room temperature on a custom-made aluminium block with 1 inch off-set stirring at 500 rpm. Filtration of the reaction mixture was carried out and the filtered precipitate was washed with water (10 mL) 4-5 times to remove residual surfactant CTAC, dried overnight in a desiccator to afford the desired product **28** as white solid (277 mg, 91% yield). Product was analysed by ^{1}H, ^{13}C and ^{19}F NMR.

δ_H (500 MHz, $CDCl_3$): 8.03 (2H, d, *J* = 8.1 Hz, ArH), 7.73 (2H, d, *J* = 8.1 Hz, ArH).

δ_C (126 MHz, $CDCl_3$): 139.1, 132.6 (d, *J* = 25.6 Hz), 129.5, 104.1.

δ_F (376 MHz, $CDCl_3$): 66.1.

Characterisation data is in agreement with those previously reported:[43] ^{1}H NMR (400 MHz, $CDCl_3$): δ [ppm] = 8.01 (d, 8.0 Hz, 2H), 7.71 (d, 8.0 Hz, 2H); ^{13}C NMR (100 MHz, $CDCl_3$): δ [ppm] = 139.2, 132.9, 129.6, 104.2; ^{19}F NMR (376 MHz, $CDCl_3$): δ [ppm] = 66.2.

o-Nitrilephenylsulfonyl fluoride 38

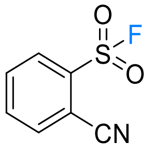

In a 4-dram glass vial (2.1 × 7 cm, 14 mL) equipped with a cross-bar stirrer (1 cm × 1 cm) were sequentially added *2*-cyanobenzenesulfonyl chloride (214 mg, 1.06 mmol), pre-stirred CTAC surfactant solution (126 mg, 2% w/w) in deionised H_2O (7 mL, 0.152 M), and KF•$2H_2O$ (300 mg, 3.19 mmol, 3 eq.). The reaction vial was sealed with a lid (PTFE septum) and was stirred for 3 hours at room temperature on a custom-made aluminium block with 1 inch off-set stirring at 500 rpm. Filtration of the reaction mixture was carried out and the filtered precipitate was washed with water (10 mL) 4-5 times to remove residual surfactant CTAC, dried overnight in a desiccator to afford the desired product **38** as white solid (159 mg, 81% yield). Product was analysed by ^{1}H, ^{13}C and ^{19}F NMR.

δ_H (500 MHz, $CDCl_3$): 8.30-8.22 (1H, m, ArH), 8.07-8.00 (1H, m, ArH), 7.99-7.89 (2H, m, ArH)

δ_C (126 MHz, $CDCl_3$): 135.9, 135.6, 135.2, 135.0, 133.5, 130.9, 114.1, 111.9.

δ_F (376 MHz, $CDCl_3$): 64.

Characterisation data is in agreement with those previously reported:[48] ^{1}H NMR (400 MHz, $CDCl_3$) δ 8.23 (dd, *J* = 6.8, 2.2 Hz, 1H), 8.01 (dd, *J* = 6.8, 2.2 Hz, 1H), 7.95–7.87 (m, 2H); ^{13}C NMR (101 MHz, $CDCl_3$) δ 136.0, 135.7, 135.2 (d, *J* = 27.8 Hz), 133.6, 131.0, 114.2, 112.0; ^{19}F NMR (377 MHz, $CDCl_3$) δ 64.6.

p-(Trifluoromethyl)benzenesulfonyl fluoride 30

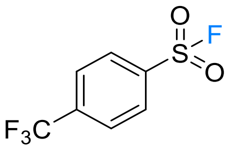

In a 4-dram glass vial (2.1 × 7 cm, 14 mL) equipped with a cross-bar stirrer (1 cm × 1 cm) were sequentially added *p*-(trifluoromethyl)benzenesulfonyl chloride (260 mg, 1.06 mmol), pre-stirred CTAC surfactant solution (126 mg, 2% w/w) in deionised H_2O (7 mL, 0.152 M), and KF•$2H_2O$ (300 mg, 3.19 mmol, 3 eq). The reaction vial was sealed with a lid (PTFE septum) and was stirred for 3 hours at room temperature on a custom-made aluminium block with 1 inch off-set stirring at 500 rpm. Filtration of the reaction mixture was carried out and the filtered precipitate was washed with water (10 mL) 4-5 times to remove residual surfactant CTAC, dried overnight in a desiccator to afford the desired product **30** as white solid (70 mg, 29% yield). Product was analysed by ^{1}H, ^{13}C and ^{19}F NMR.

δ_H (500 MHz, $CDCl_3$): 8.17-8.06 (2H, m, ArH), 7.89-7.80 (2H, m, ArH).

δC (126 MHz, CDCl3): 137.2 (q, *J* = 33.6 Hz) 136.7 (d, *J* = 33.5 Hz), 129.1, 126.9 (q, *J* = 5.5 Hz), 122.7 (q, *J* = 273.4 Hz)

δ_F (376 MHz, $CDCl_3$): 65.8 ($SO_2\underline{F}$), -63.5 ($C\underline{F}_3$).

Characterisation data is in agreement with those previously reported:[43] ^{1}H NMR (400 MHz, $CDCl_3$): δ [ppm] = 8.17 (d, 8.0 Hz, 2H), 7.92 (d, 8.0 Hz, 2H); ^{13}C NMR (100 MHz, $CDCl_3$): δ [ppm] = 137.5, 137.2, 136.8, 129.3, 127.1, 127.1, 127.0, 127.0, 124.2, 121.5; ^{19}F NMR (376 MHz, $CDCl_3$): δ [ppm] = 65.9, -63.5.

2,4-Demethoxyphenylsulfonyl fluoride 42

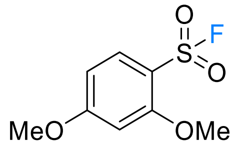

In a 4-dram glass vial (2.1 × 7 cm, 14 mL) equipped with a cross-bar stirrer (1 cm × 1 cm) were sequentially added 2,4-Dimethoxybenzenesulfonyl chloride (252 mg, 1.06 mmol), pre-stirred CTAC surfactant solution (126 mg, 2% w/w) in deionised H_2O (7 mL, 0.152 M), and KF•2H_2O (300 mg, 3.19 mmol, 3 eq). The reaction vial was sealed with a lid (PTFE septum) and was stirred for 3 hours at room temperature on a custom-made aluminium block with 1 inch off-set stirring at 500 rpm. Filtration of the reaction mixture was carried out and the filtered precipitate was washed with water (10 mL) 4-5 times to remove residual surfactant CTAC, dried overnight in a desiccator to afford the desired product **42** as white solid (218 mg, 93% yield). Product was analysed by ^{1}H, ^{13}C and ^{19}F NMR.

δ_H (500 MHz, $CDCl_3$): 7.71 (1H, d, *J* = 8.8 Hz, ArH), 6.52-6.42 (2H, m, ArH), 3.86 (3H, s, OMe), 3.80 (3H, s, OMe).

δ_C (126 MHz, $CDCl_3$): 167.2, 159.9, 133.1, 113.0, 105.3, 99.4, 56.5, 56.0.

δ_F (376 MHz, $CDCl_3$): 60.3.

Characterisation data is in agreement with those previously reported:[49] ^{1}H NMR ($CDCl_3$, 500 MHz) δ 7.84 (d, *J* = 8.9 Hz, 1H), 6.57 (d, *J* = 8.8 Hz, 1H), 6.54 (d, *J* = 2.3 Hz, 1H), 3.96 (s, 3H), 3.89 (s, 3H). ^{19}F NMR ($CDCl_3$, 471 MHz) δ 59.9 (s, 1F). ^{13}C NMR ($CDCl_3$, 126 MHz) δ 167.2., 160.0 (d, *J* = 1.9 Hz), 133.3, 113.4 (d, *J* =23.6 Hz), 105.2, 99.6, 56.6, 56.1.

Octane-1-sulfonyl fluoride 40

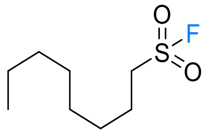

In a 4-dram glass vial (2.1 × 7 cm, 14 mL) equipped with a cross-bar stirrer (1 cm × 1 cm) were sequentially added octane-1-sulfonyl chloride (225 mg, 1.06 mmol), pre-stirred CTAC surfactant solution (126 mg, 2% w/w) in deionised H_2O (7 mL, 0.152 M), and KF•2H_2O (300 mg, 3.19 mmol, 3 eq). The reaction vial was sealed with a lid (PTFE septum) and was stirred for 3 hours at room temperature on a custom-made aluminium block with 1 inch off-set stirring at 500 rpm. The crude reaction mixture was diluted with DCM and deionised H_2O, the layers were partitioned utilising brine, and the aqueous phase was extracted three times with DCM.

The organic layers were combined, dried ($MgSO_4$), filtered, and concentrated *in vacuo* after passing the solution through a plug of silica to afford the desired product **40** as colourless oil (170 mg, 87% yield). Product was analysed by ^{1}H, ^{13}C and ^{19}F NMR.

δ_H (500 MHz, $CDCl_3$): 3.42-3.33 (2H, m, CH_2), 2.06-1.89 (2H, m, CH_2), 1.57-1.44 (2H, m, CH_2), 1.42-1.22 (8H, m, CH_2), 1.00-0.83 (3H, m, Me).

δC (126 MHz, CDCl3): 50.9 (d, J = 15.98 Hz), 31.6, 28.8, 28.7, 27.8, 23.4, 22.6, 14.0.

δ_F (376 MHz, $CDCl_3$): 53.4.

Characterisation data is in agreement with those previously reported:[43] ^{1}H NMR (400 MHz, $CDCl_3$): δ [ppm] = 3.38-3.33 (m, 2H), 1.98-1.91 (m, 2H), 1.51-1.44 (m, 2H), 1.33-1.28 (m, 8H), 0.89 (t, 7.2 Hz, 3H); ^{13}C NMR (100 MHz, $CDCl_3$): δ [ppm] = 51.1, 50.9, 31.8, 29.0, 29.0, 28.0, 23.5, 22.7, 14.2; ^{19}F NMR (376 MHz, $CDCl_3$): δ [ppm] = 53.2.

5.2.4 Results of screening with *surfactant_map*

The results, yield of **22**, is summarised below:

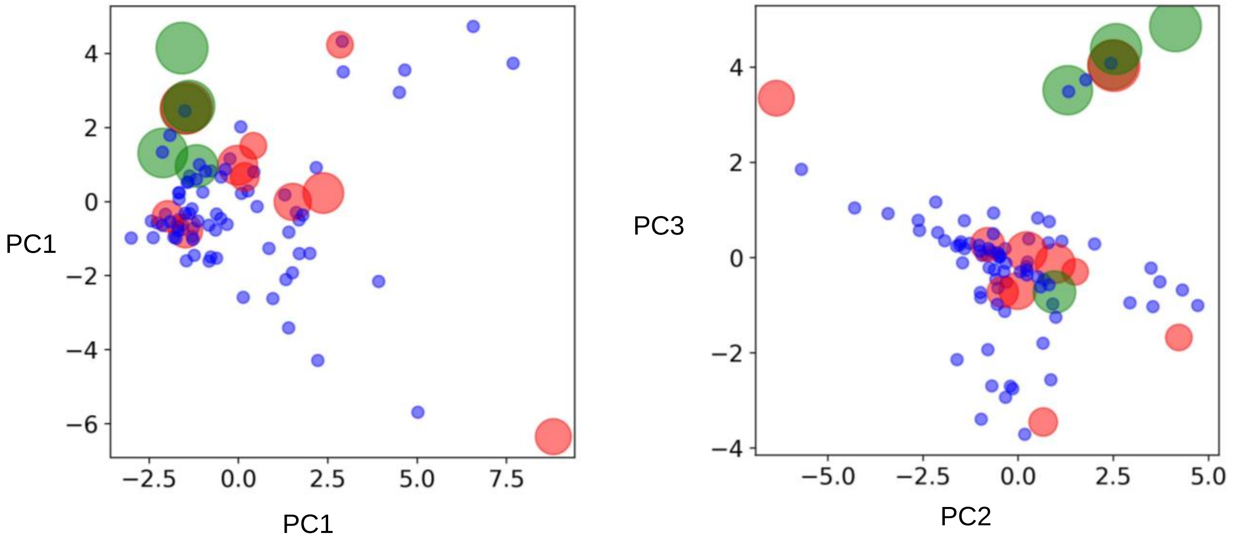

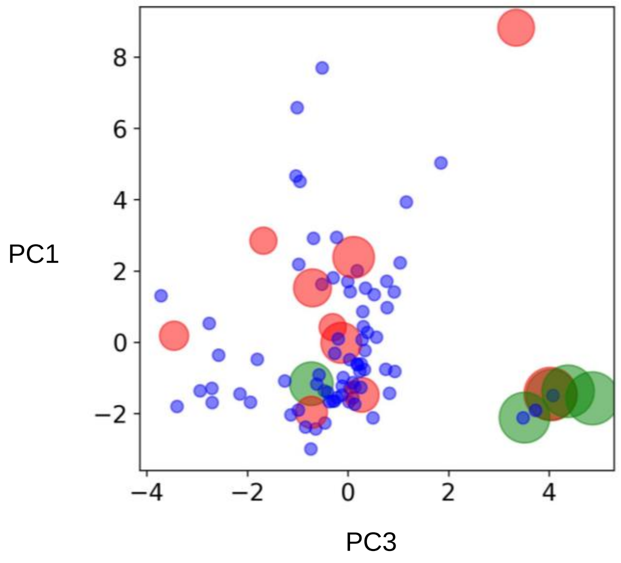

Figure S56. Projected reaction yields (dot size) with screened surfactants (red for ***screen1*** and green for ***screen2***) onto PC1-PC2, PC2-PC3 and PC3-PC1 combinations

Table S19. Surfactant screen for fluorination of **21** to **22**[a]

No.	Surfactant	^{1}H NMR yield of **22** (%)
1	None	13
2	Span 60	57
3	Span 80	25
4	Span 85	25
5	Triton-X-45	41
6	Brij-700	46
7	TPGS-1000-M	59
8	1-Dodecanesulfonic acid sodium salt	35
9	PS-750-M	49
10	CHAPS	29
11	CTAC	96
12	CPC	94
13	CTAB	90

14	DDAB	96
15	DTAB	88
16	Sodium stearate	65
17	18-crown-6	77

[a]0.15 M substrate, 1.5 eq. of $KF.2H_2O$, 2% w/w surfactant at room temperature, 3h reaction time.

5.3 Microscope images of reaction mixtures

5.3.1 Images of a mixture of 21 and CTAC in water

In a 4-dram glass vial (2.1 × 7 cm, 14 mL) equipped with a cross-bar stirrer (1 cm × 1 cm) were sequentially added *p*-toluenesulfonyl chloride (29 mg, 0.15 mmol) and pre-stirred CTAC surfactant solution (18 mg, 2% w/w) in deionised H_2O (1 mL, 0.152 M). After stirring the reaction mixture for 5 minutes an aliquot from the mixture was taken out on a slide for microscope analysis.

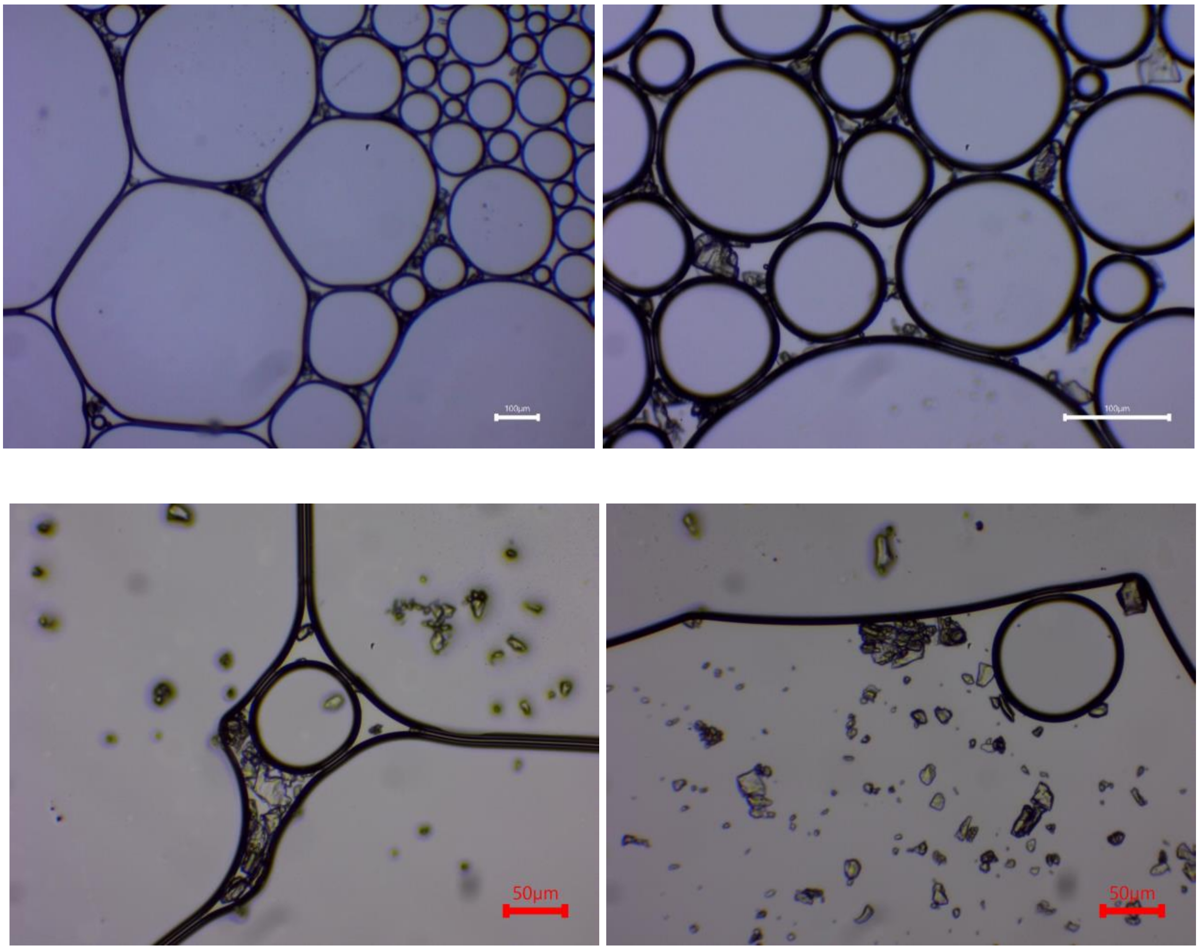

Figure S57. Microscope images of a mixture of CTAC (2% w/w) and substrate **21** from two different experiments

5.3.2 Images of a mixture of 21 and CTAC in water after application of pressure

Pressure was applied to the glass slides housing the samples above before the images were collected again.

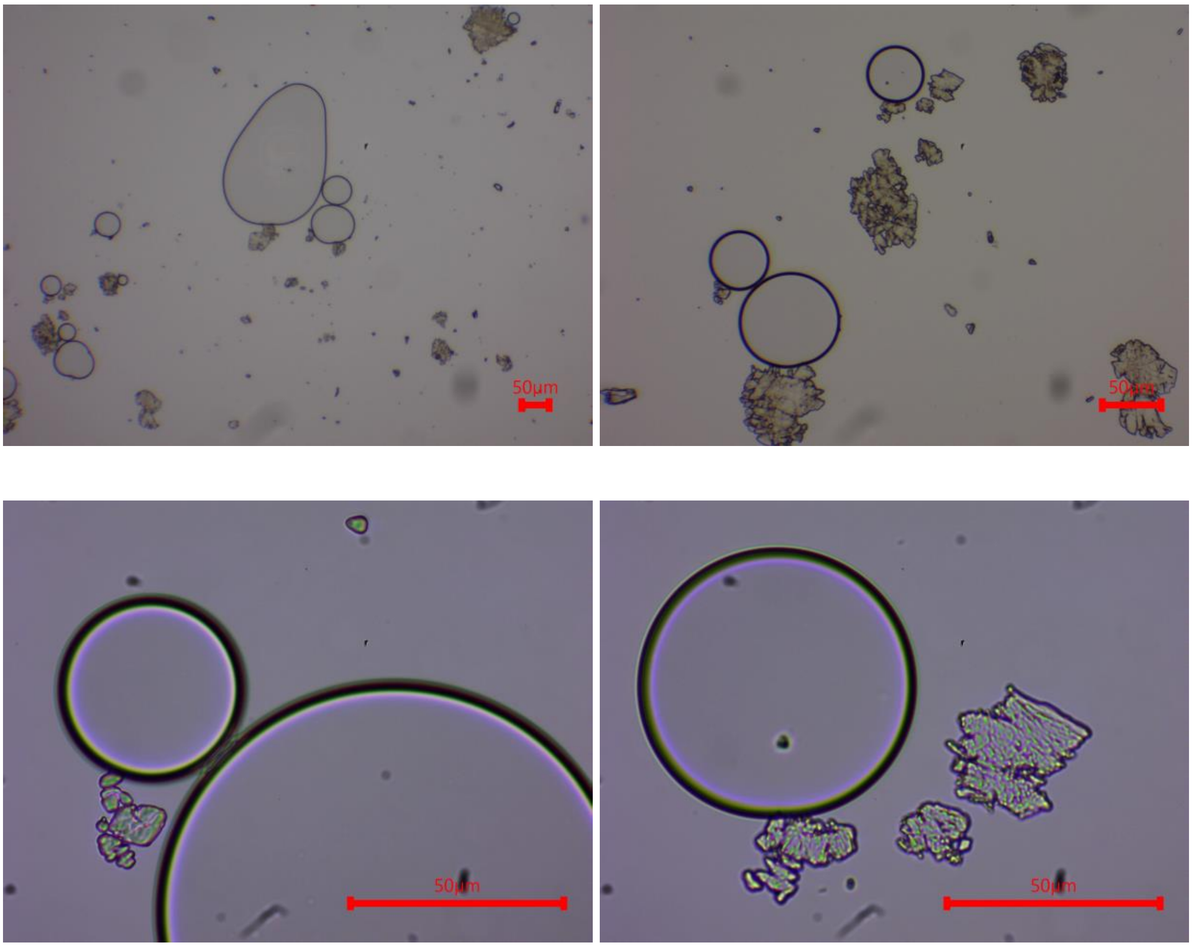

Figure S58. Microscope images of a mixture of CTAC (2% w/w) and substrate **21** after pressure was applied to the glass slides

5.4 Labelling of chymotrypsin with sulfonyl fluoride 44

5.4.1 Preparation of 4-(Prop-2-ynyloxy)benzenesulfonyl chloride 43

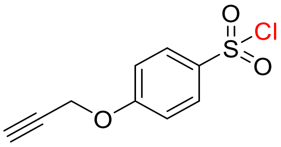

Chlorosulfonic acid (1.156 mL, 17.4 mmol, 2.3 eq) was added dropwise to substrate phenyl propargyl ether (0.97 ml, 7.57 mmol) taken in DCM (5.82 mL, 1.3 M) at 0 °C. The reaction was stirred at 0 °C for 1 hour and then poured into ice-water and extracted with DCM. The extract was washed with water, dried with $MgSO_4$, and evaporated. The residue was purified by flash column chromatography (30% EtOAc/Hexane) to afford product **43** as an oil (695 mg, 40% yield).

δ_H (500 MHz, $CDCl_3$): δ7.93 (2H, d, J = 9.2 Hz, ArH), 7.07 (d, 2H, J = 9.1 Hz, Ar H), 4.74 (2H, d, J = 2.4 Hz, CH_2), 2.54 (1H, t, J = 2.4 Hz, ≡CH).

δ_C (126 MHz, $CDCl_3$): 162.6, 137.0, 129.5, 115.6, 77.0, 76.8, 56.3.

5.4.2 Preparation of 4-(Prop-2-ynyloxy)benzenesulfonyl fluoride 44

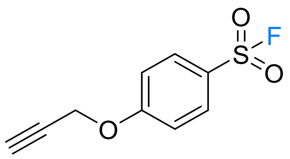

44

In a 4-dram glass vial (2.1 × 7 cm, 14 mL) equipped with a cross-bar stirrer (1 cm × 1 cm) were sequentially added the appropriate sulfonyl chloride substrate **43** (151 mg, 0.65 mmol), KF•2H2O (185 mg, 1.96 mmol, 3 eq), and pre-stirred CTAC surfactant solution (2% w/w) in deionised H_2O (4.3 mL, 0.152 M). The reaction vial was sealed with a lid (PTFE septum) and was stirred for 3 hours at room temperature on a custom-made aluminium block with 1 inch off-set stirring at 500 rpm. The crude reaction mixture was diluted with DCM and deionised H_2O, the layers were partitioned utilising Brine, and the aqueous phase was extracted three times with DCM. The organic layers were combined, dried ($MgSO_4$), filtered, and concentrated *in vacuo* after passing the solution through a plug of silica, to afford the desired product **44** (138 mg, 98% yield) as an oil. The isolated product was dissolved in $CDCl_3$ and analysed by ^{1}H and ^{19}F NMR.

δ_H (500 MHz, $CDCl_3$): δ 7.89 (2H, d, J = 8.9 Hz, ArH), 7.08 (d, 2H, J = 8.9 Hz, ArH), 4.73 (2H, d, J = 2.4 Hz, CH_2), 2.53 (1H, t, J = 2.4 Hz, ≡CH).

δ_C (126 MHz, $CDCl_3$): 163.0, 130.8, 125.0 (d, J = 24.9 Hz), 115.8, 77.0, 76.9, 56.2.

δ_F (376 MHz, $CDCl_3$): 67.2.

Characterisation data is in agreement with literature data:[50] ^{1}H NMR (400 MHz, CD_2Cl_2): δ 2.66 (t, J = 2.4 Hz, 1H); 4.83 (d, J = 2.4 Hz, 2H); 7.18 (d, J = 8.7 Hz, 2H); 7.97 (d, J = 8.97 Hz, 2H). ^{19}F NMR (300 MHz, CD_2Cl_2): δ 66.16. ^{13}C NMR (400 MHz, CD_2Cl_2): δ 56.75; 77.01; 77.29; 116.18; 125.18 [C, JC-F = 24.9 Hz]; 131.17; 163.49.

5.4.3 Labelling experiment

Protein stock solution: A 100 µM stock solution of bovine chymotrypsin was prepared in PBS by dissolving 20 mg bovine chymotrypsin in 8 mL PBS. Bovine chymotrypsin (Product Number: C4129) and PBS (Product Number: P2272) were purchased from Sigma Aldrich.

Stock solution of **44**: A 1 mM stock solution of sulfonyl fluoride **44** was prepared in DMSO. This was diluted with PBS to a 100 µM solution for a final DMSO concentration of 10%.

Protein labelling protocol- 5 μL of 100 μM chymotrypsin solution and 5 μL of 100 μM sulfonyl fluoride **44** solution were added to 40 μL PBS taken in an LC-MS vial (final concentrations: 10 μM protein, 10 μM sulfonyl fluoride, 1% DMSO in 50 μL total volume). Another control sample with no sulfonyl fluoride was also prepared by taking 5 μL of 100 μM chymotrypsin solution and 5 μL of 1% DMSO in 40 μL PBS in another LC-MS vial. These samples were analysed by LC-MS at time intervals of 1h and 20h.

LC-MS was performed using ProteinQuick protocol for the determination of protein masses. Method also uses maximum entropy deconvolution methods as part of the processing. Column: Waters Acquity Vanguard Protein BEH C4 300A 1.7 μm, 2.1 mm × 100 mm (p/n 186004496). Guard column: Waters Acquity Vanguard Protein BEH C4 300A 1.7 μm, 2.1 mm × 5 mm (p/n 186004623). Eluent: 0.7 mL/min; A: Water (0.1% formic acid); B: Acetonitrile (0.1% formic acid). Gradient (linear interpolation): 99% A. Ionisation method: +ev electrospray.

6 Python and R code

6.1 Calculating the volumes of hydrophobic and hydrophilic regions (Python)

```
# Import Python packages rdkit and cirpy

import cirpy

import rdkit

from rdkit.Chem import AllChem

from rdkit import Chem

# Convert the name "CTAB" to the surfactant SMILES code

cirpy.resolve('CTAB', 'smiles')

# The hydrophobic region SMILES code is 'X'. Convert the SMILES code 'X' to a chemical
structure

smileshydrophobic = 'CCCCCCCCCCCCCCCC'

hydrophobic = AllChem.MolFromSmiles(smileshydrophobic)

hydrophobic

# The hydrophilic region SMILES code is 'X'. Convert the SMILES code 'X' to a chemical
structure

smileshydrophilic = '[N+](C)(C)C'

hydrophilic = AllChem.MolFromSmiles(smileshydrophilic)

hydrophilic

# Add hydrogens to the hydrophobic structure

molhydrophobic = Chem.AddHs(hydrophobic)

molhydrophobic

# Add hydrogens to the hydrophilic structure

molhydrophilic = Chem.AddHs(hydrophilic)

molhydrophilic

# Calculate volume from hydrophobic structure

AllChem.EmbedMolecule(molhydrophobic)

AllChem.ComputeMolVolume(molhydrophobic)

# Calculate volume from hydrophilic structure

AllChem.EmbedMolecule(molhydrophilic, useRandomCoords=True)

AllChem.ComputeMolVolume(molhydrophilic)

# Convert hydrophobic structure to an xyz. File named 'molhydrophobic'

Chem.MolToXYZFile(molhydrophobic, 'molhydrophobic.xyz')

# Convert hydrophilic structure to an xyz. File named 'molhydrophilic'

Chem.MolToXYZFile(molhydrophilic, 'molhydrophilic.xyz')
```

6.2 Calculating surface areas hydrophilic and hydrophobic surfactant region (PyMOL)

```
Set dot_solvent, on/1

# Calculate SASA (Solvent Accessible Surface Area)

Set dot_density,4

# Use a 4 dot density (1-4) where 4 is the most accurate

Set solvent_radius,1.4

# Set solvent radius to 1.4 A (Radius for water)

Get_area

# Calculate area of molecule
```

6.3 Counting rotatable bonds (Python)

```
# Import Python packages rdkit

from rdkit import Chem

# Import Python packages calculate number of rotatable bonds package

from rdkit.Chem.rdMolDescriptors import CalcNumRotatableBonds

# Denoting "smi" as surfactant SMILES code used from previous volume calculation

smi="CCCCCCCCCCCCCCCCCC=O"

# Generating molecule from SMILES code and denoting this as "mol"

mol=Chem.MolFromSmiles(smi)

# Add hydrogens to structure

mol=Chem.AddHs(mol)

# Calculate number of rotatable bonds and print number

print(CalcNumRotatableBonds(mol))
```

6.4 Calculating longest chain length (Python)

```
import cirpy

import rdkit

import numpy as np

from rdkit.Chem import AllChem

from rdkit import Chem

from collections import deque


# Import Python packages rdkit and cirpy
# This class allow us to build a graph of connectivity to represent the molecules.
# And it will also quickly find the longest path, which is what we want.
# The code was adapted from https://www.geeksforgeeks.org/longest-path-undirected-tree/
[illegible]
```

```
# Intialisation of graph
    def __init__(self, vertices):
        # No. of vertices
        self.vertices = vertices
        # adjacency list
        self.adj = {i: [] for i in range(self.vertices)}
     def addEdge(self, u, v):
        # add u to v's list
        self.adj[u].append(v)
        # since the graph is undirected
        self.adj[v].append(u)
    # method return farthest node and its distance from node u
    def BFS(self, u):
        # marking all nodes as unvisited
        visited = [False for i in range(self.vertices + 1)]
        # mark all distance with -1
        distance = [-1 for i in range(self.vertices + 1)]
        # distance of u from u will be 0
        distance[u] = 0
        # in-built library for queue which performs fast operations on both the ends
        queue = deque()
        queue.append(u)
        # mark node u as visited
        visited[u] = True
         while queue:
            # pop the front of the queue(0th element)
            front = queue.popleft()
            # loop for all adjacent nodes of node front
            for i in self.adj[front]:
                if not visited[i]:
                    # mark the ith node as visited
                    visited[i] = True
                    # make distance of i , one more than distance of front
                    distance[i] = distance[front]+1
                    # Push node into the stack only if it is not visited already
```

```
                    queue.append(i)


        maxDis = 0
        # get farthest node distance and its index
        for i in range(self.vertices):
            if distance[i] > maxDis:
                 maxDis = distance[i]
                nodeIdx = i
        return nodeIdx, maxDis
    # method prints longest path of given tree
    def LongestPathLength(self):
        # first DFS to find one end point of longest path
        node, Dis = self.BFS(0)
        # second DFS to find the actual longest path
        node_2, LongDis = self.BFS(node)
        print('Longest path is from', node, 'to', node_2, 'of length', LongDis)
# ask cirpy to get the SMILES code for the surfactant
cirpy.resolve('toluene', 'smiles')
smileshydrophobic = 'CNC1CCC(C2=CC=CC=C12)C3=CC(=C(C=C3)Cl)Cl'
mol = AllChem.MolFromSmiles(smileshydrophobic)
# Label all atoms with index number
atoms = mol.GetNumAtoms()
for idx in range( atoms ):
        mol.GetAtomWithIdx( idx ).SetProp( 'molAtomMapNumber', str(
mol.GetAtomWithIdx( idx ).GetIdx() ) )
        #print(mol.GetBonds()[0].GetBondType())
# Set up the bond matrix of the right size
print(atoms, 'x', atoms)
size = atoms * atoms
bonds_matrix = np.arange(size).reshape(atoms,atoms)
# We go through all the bonds and assign values to the bond matrix
# We assign 1 for any bond, and 0 for no bond
for idx1 in range(atoms):
    for idx2 in range(atoms):
        bond = mol.GetBondBetweenAtoms(idx1,idx2)
```

```
        if bond == None:
            bond_number = 0
        else:
            bond_number = 1
        bonds_matrix[idx1,idx2] = bond_number
# Now we use the bond matrix to find all the bonds between 2 atoms.
# We also create a graph to store the graph presentation of the bonds.
bonded_atoms = []
G = Graph(atoms)
for idx1 in range(atoms):
    row = bonds_matrix[idx1]
    for idx2 in range(atoms):
        if row[idx2] == 1:
            bonded_atoms.append([idx1, idx2])
            G.addEdge(idx1, idx2)
print(bonded_atoms)
G.LongestPathLength()
```

6.5 Principal component analysis

6.5.1 Normalise data (Python)

```
df = pd.read_csv('pca_data_date.csv')

# Read in PCA dataset csv file

df.columns

# Data frame columns

del df['Name']

df.columns

# Remove column names before normalising

def normalize(df):

result = df.copy()

for feature_name in df.columns:

max_value = df[feature_name].max()

min_value = df[feature_name].min()

result[feature_name] = (df[feature_name] - min_value) / (max_value - min_value)

return result

# Normalise dataset

norm_df = normalize(df)

norm_df.to_csv('norm_data_29062022.csv')

# Convert dataset back to csv file
```

6.5.2 Perform principal component analysis (R)

```
library(readxl)

# Loading read excel package

pca_dat <- read_excel("norm_data_29062022.xlsx")

#Read in excel file

library(pcaMethods)

# Load library and pca methods

pca_matrix <- data.matrix(pca_dat)
```

```
#Converting the dataframe into a matrix
summary(pca_matrix)
# Generate summary of PCA
ppca_fit <- ppca(pca_matrix, nPcs=5, seed=NA, threshold=1e-05, maxIterations=10000,
center=TRUE, scale=TRUE)
# PPCA, npcs=5 - 5 principal components, seed=NA - PPCA creates fills the initial
loading matrix with random numbers chosen from a normal distribution. Thus results may
vary slightly. Set the seed for exact reproduction of your results.
# Threshold - convergence threshold
summary(ppca_fit)
# Summary of this data giving R2 scores
print(ppca_fit)
# Show summary of this analysis
ppca_scores <- ppca_fit@scores
ppca_loadings <- loadings(ppca_fit)
# Putting scores and loadings as standardised names
write.csv(ppca_scores, "ppca_scores.csv", row.names=TRUE)
write.csv(ppca_loadings, "ppca_loadings.csv", row.names=TRUE)
# Saving R dataframe as a csv file
bpca_fit <- bpca(pca_matrix, nPcs=5, seed=NA, threshold=1e-05, maxIterations=10000,
maxSteps=100, center=TRUE, scale=TRUE)
# Bpca analysis
summary(bpca_fit)
# Summary of this data giving R2 scores
bpca_scores <- bpca_fit@scores
bpca_loadings <- loadings(bpca_fit)
# Putting scores and loadings as standardised names
write.csv(bpca_scores, "bpca_scores.csv", row.names = TRUE)
write.csv(bpca_loadings, "bpca_loadings.csv", row.names = TRUE)
# Saving R dataframe as a csv file
nlpca_fit <- pca(pca_matrix, nPcs=5, method="nlpca", maxSteps=1000, center=TRUE,
scale=NULL)
# Nlpca analysis
summary(nlpca_fit)
# Summary of this data giving R2 scores
```

```
nlpca_scores <- nlpca_fit@scores

nlpca_loadings <- loadings(nlpca_fit)

# Putting scores and loadings as standardised names

write.csv(nlpca_scores, "nlpca_scores.csv", row.names = TRUE)

write.csv(nlpca_loadings, "nlpca_loadings.csv", row.names = TRUE)

# Saving R dataframe as a csv file

library(nipals)

# Nipals isn't a pca methods so load it separately

nipals_fit <- nipals(pca_matrix, ncomp=min(nrow(pca_matrix),ncol(pca_matrix)),
center=TRUE, scale=TRUE, maxiter=10000, tol=1e-06, startcol=0, fitted=FALSE,
gramschmidt=TRUE, verbose=FALSE)

# Nipals PCA

nipals_scores <- nipals_fit$scores

nipals_loadings <- loadings(nipals_fit)

# Putting scores and loadings as standardised names

print(nipals_fit$R2)

# Summary doesn't automatically give R2 scores so load them up this way

write.csv(nipals_scores, "nipals_scores.csv", row.names = TRUE)

write.csv(nipals_loadings, "nipals_loadings.csv", row.names = TRUE)

# Saving R dataframe as a csv file
```

6.5.3 Perform 100 different runs in principal component analysis (R)

```r
# Read in the data

library(readr)

pca_data_raw <- read_csv("pca_data_updated_14022023_trimmed_noC16E6.csv")

View(pca_data_raw)


# Scale the data

df = subset(pca_data_raw, select = -c(Name))

pca_data <- scale(df)


# Run PCA 100 times

library(pcaMethods)

for (x in 1:100) {

	print(x)

	nipals_fit <- pca(pca_data, nPcs=3, method="nipals", maxSteps=100)

	nipals_scores <- nipals_fit@scores

	filename_scores <- paste("nipals_run",x,"trimmed_noC16E6.csv",sep = '_')

	write.csv(nipals_scores, filename_scores, row.names = TRUE)

	nipals_loadings <- nipals_fit@loadings

	filename_loadings <- paste("nipals_run",x,"trimmed_noC16E6_loadings.csv",sep =
'_')

	write.csv(nipals_loadings, filename_loadings, row.names = TRUE)

}
```

6.5.4 Averaging the PCs of 100 different runs of PCA (Python)

```python
import pandas as pd

import glob

# create a list of file paths using glob

file_paths = glob.glob("*.csv")

# create an empty list to store the dataframes

df_list = []

# loop through each file path and read the csv file into a dataframe

for file_path in file_paths:

    df = pd.read_csv(file_path)
```

```
    df_list.append(df)
# create a pandas series of dataframes
df_series = pd.Series(df_list)
# compute the median value for each entry in the dataframes
median_df = pd.concat(df_list).groupby(level=0).median()
mean_df = pd.concat(df_list).groupby(level=0).mean()
std_df = pd.concat(df_list).groupby(level=0).std()
# saving the data into csv files
median_df.to_csv("nipals_trimmed_scaled_scores_median_noC16E6.csv")
mean_df.to_csv("nipals_trimmed_scaled_scores_mean_noC16E6.csv")
std_df.to_csv("nipals_trimmed_scaled_scores_stdev_noC16E6.csv")
```

6.6 NIPALS map plotting (Python)

```
import numpy as np
import matplotlib.pyplot as plt
import pandas as pd
# read in data file
df = pd.read_csv('Fig3c.csv')
# Creating figure
fig = plt.figure(figsize=(15,15), dpi=300)
ax = plt.axes(projection='3d')
# Creating plot
df['T_C'].map(colors), s=64, alpha=1)
ax.scatter3D(df['PC1'], df['PC2'], df['PC3'], color = df['Colour'], s =
df['Ratio']*100, alpha=0.5)
# remove colours from the planes
ax.xaxis.pane.fill = False
ax.yaxis.pane.fill = False
ax.zaxis.pane.fill = False
# set up axis labels
ax.set_xlabel('PC1', labelpad=14, fontsize= 18)
ax.set_ylabel('PC2', labelpad=14, fontsize= 18)
ax.set_zlabel('PC3', labelpad=14, fontsize= 18)
```

```
# set up axis labels
ax.set_xlabel('PC1', labelpad=14, fontsize= 18)
ax.set_ylabel('PC2', labelpad=14, fontsize= 18)
ax.set_zlabel('PC3', labelpad=14, fontsize= 18)
# set up axis ticks size
ax.tick_params(axis='x', labelsize=14)
ax.tick_params(axis='y', labelsize=14)
ax.tick_params(axis='z', labelsize=14)
# set grid lines
ax.grid(True)
# rotate the plot for a better viewing angle
ax.view_init(20, 38)
# show plot
plt.savefig('Fig3c.jpg')
plt.show()
```

6.7 Python code for surfactant selection

```
import pandas as pd
import math
import random

df = pd.read_csv("surfactant_map.csv")

# the numbering of the first surfactant hit for the reaction
hit_index = 26
# the total number of cycles for optimisation of covering surfactant space
max_cycles = 1000
# the number of surfactants to be selected in the screen
number_of_surfactants = 8

# function to calculate the distance in 3D space between two surfactants in PC1-3
def distance(index1,index2):
    pc1_1 = float(df['PC1'][index1-1])
    pc2_1 = float(df['PC2'][index1-1])
```

```
    pc3_1 = float(df['PC3'][index1-1])

    pc1_2 = float(df['PC1'][index2-1])

    pc2_2 = float(df['PC2'][index2-1])

    pc3_2 = float(df['PC3'][index2-1])

    calc_distance = math.sqrt((pc1_1 - pc1_2)**2 + (pc2_1 - pc2_2)**2 + (pc3_1 -
pc3_2)**2)

    return calc_distance


# Calculate sum of distances to index 1

def distance_sum(hit_indexes):

    sum_of_distances = 0

    for ind in df['Number']:

        distance_from_hit = 0

        if ind not in hit_indexes :

            distance_from_hit = 100

            for hit_ind in hit_indexes:

                individual_distance = distance(hit_ind, ind)

                # print (individual_distance)

                if individual_distance < distance_from_hit:

                    distance_from_hit = individual_distance

        sum_of_distances = sum_of_distances + distance_from_hit

    return sum_of_distances


def all_hit_indexes():

    gen_hit_indexes = list(combinations(df['Number'], number_of_surfactants-1))

    all_hit_indexes = [list(elem) for elem in gen_hit_indexes] # convert into list of
lists

    return all_hit_indexes


# need a faster way of generating the first index_combinations = []

index_list = list(df['Number'])

index_list.remove(hit_index)

hit_indexes = []
```

```
for i in range (1, number_of_surfactants):
    random_item = random.choice(index_list)
    index_list.remove(random_item)
    hit_indexes.append(random_item)

# create a copy and add the first hit index
full_hit_indexes = hit_indexes
full_hit_indexes.append(hit_index)
print(full_hit_indexes)

# store data of the first position
current_hit_indexes = full_hit_indexes
current_distance = distance_sum(current_hit_indexes)
print (current_hit_indexes, current_distance)

# optimisation code to minimise distance by swapping one element in the list at a time
temp_hit_indexes = current_hit_indexes.copy()
for i in range(1, max_cycles):
    remove_index = random.randint(0, len(temp_hit_indexes)-1)
    new_index = random.randint(1, len(df['Number']))
    if temp_hit_indexes[remove_index] != hit_index:
        if new_index not in current_hit_indexes:
            temp_hit_indexes.remove(temp_hit_indexes[remove_index])
            temp_hit_indexes.append(new_index)
            temp_distance = distance_sum(temp_hit_indexes)
            if temp_distance < current_distance:
                current_hit_indexes = temp_hit_indexes.copy()
                current_distance = distance_sum(current_hit_indexes)
                print (current_hit_indexes, current_distance)
        temp_hit_indexes = current_hit_indexes.copy()
print ('Final results: ',current_hit_indexes, current_distance)

final_data = []
final_datum = [current_hit_indexes, current_distance]
```

```
final_data.append(final_datum)

final_df = pd.DataFrame(final_data, columns = ['Indexes', 'Distance_sum'])


final_df.to_csv('nozp__8_surfactants_from_26.csv', index=False)
```

7 Spectral data

1,3-Bis(3,5-bis(trifluoromethyl)phenyl)urea 14

^{1}H NMR

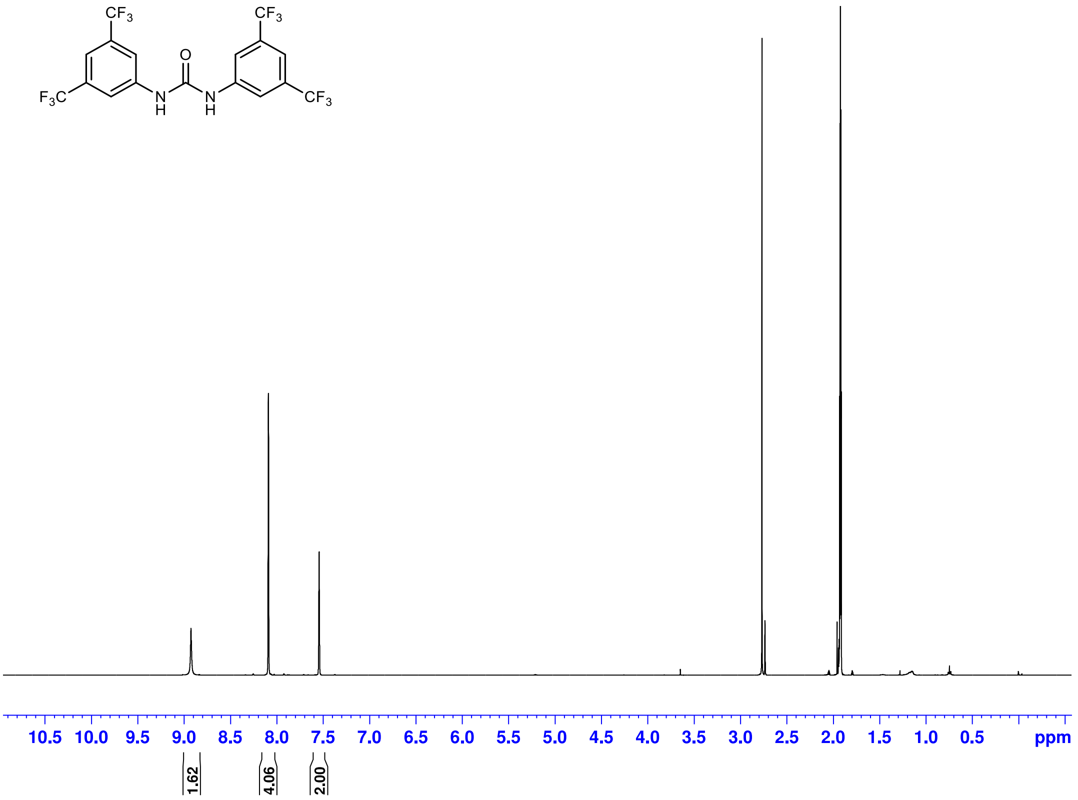

^{13}C NMR

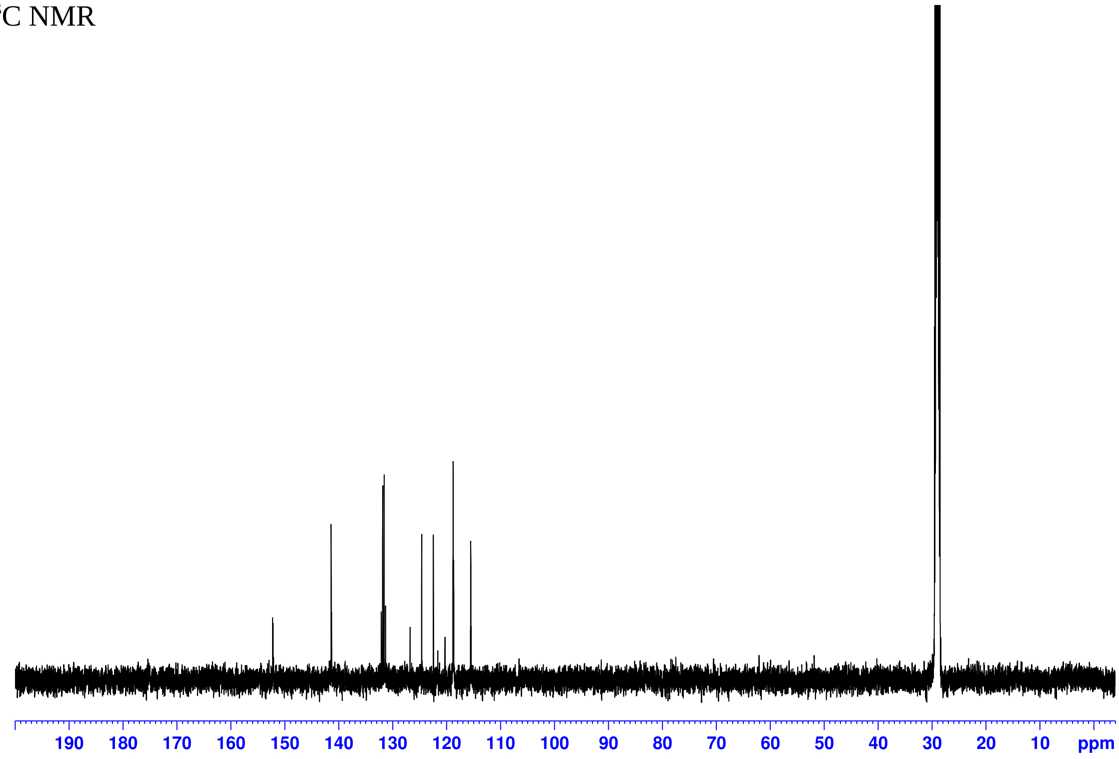

^{1}H NMR

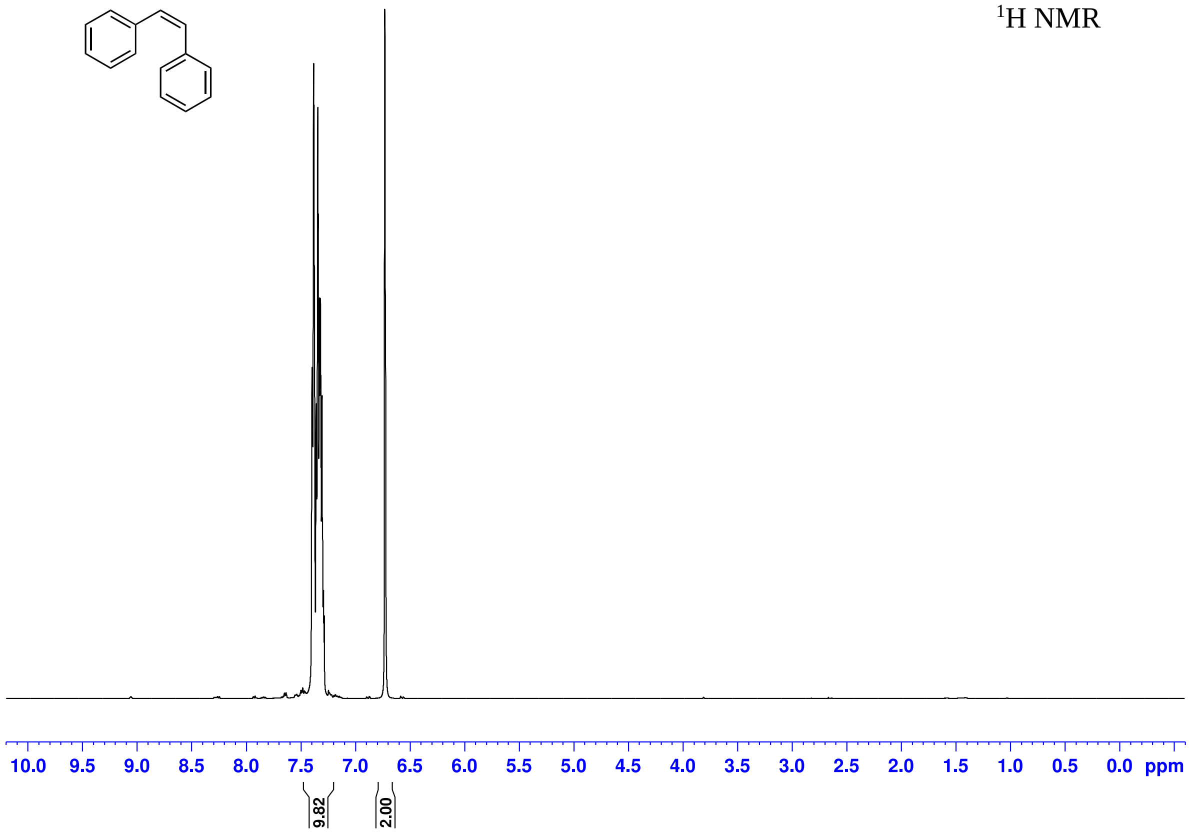

^{13}C NMR

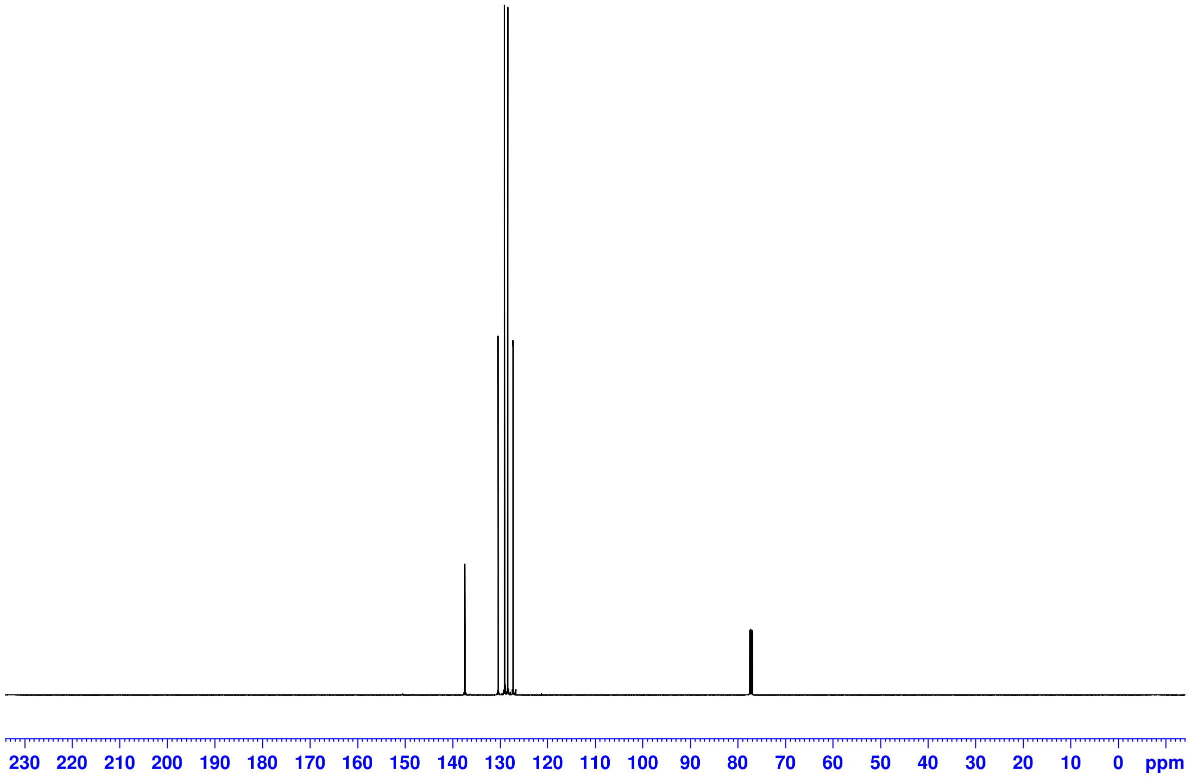

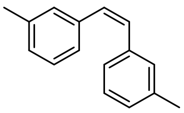

^{1}H NMR

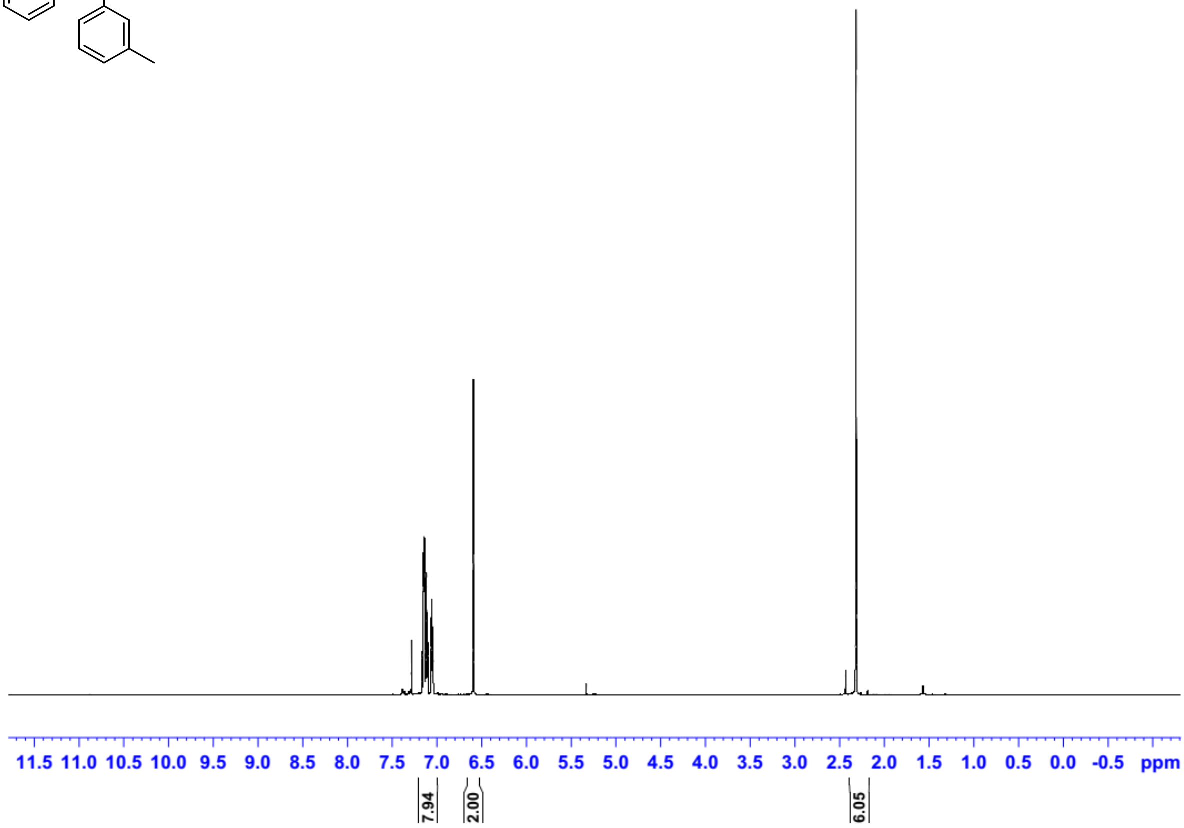

^{13}C NMR

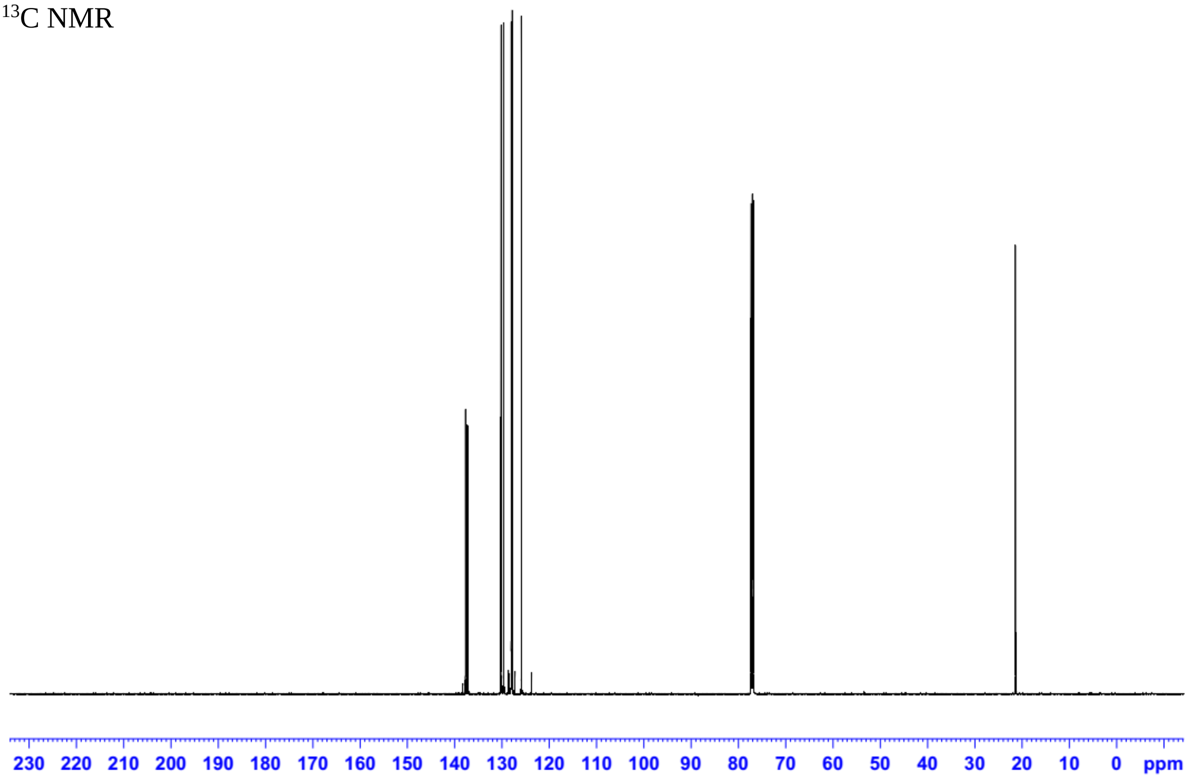

^{1}H NMR

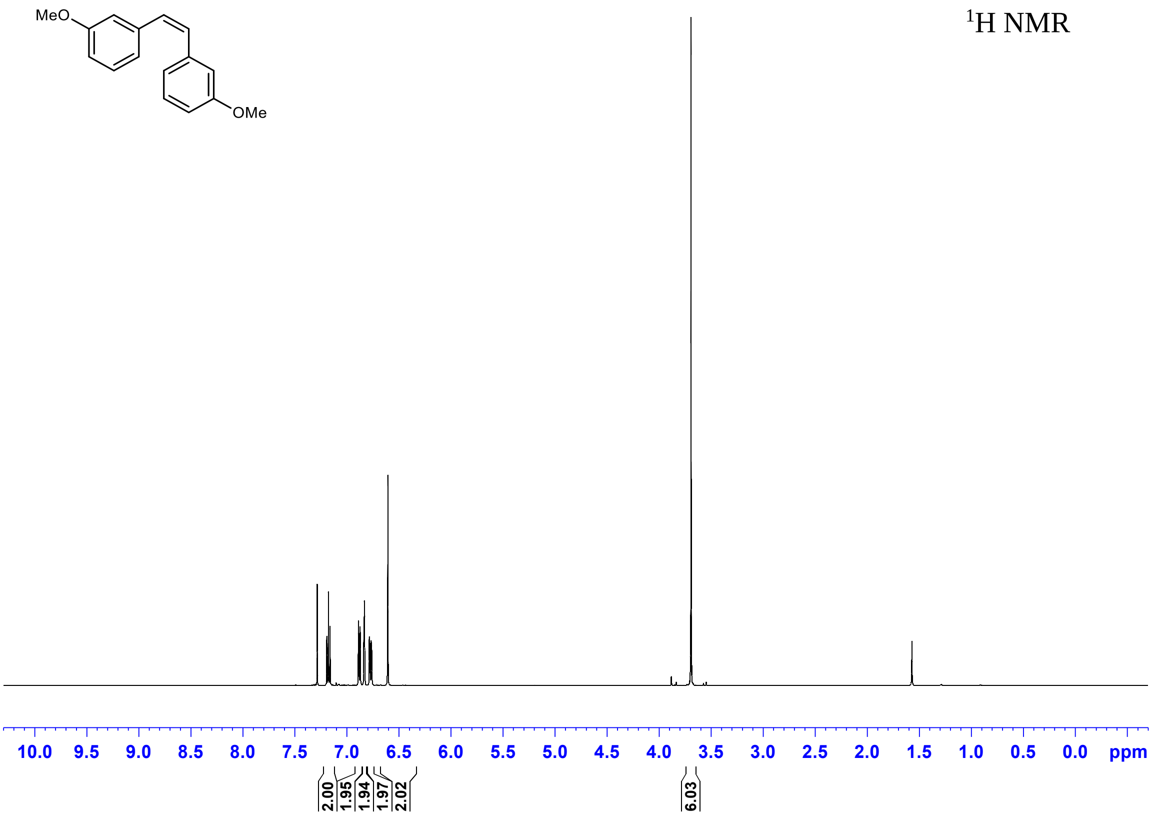

^{13}C NMR

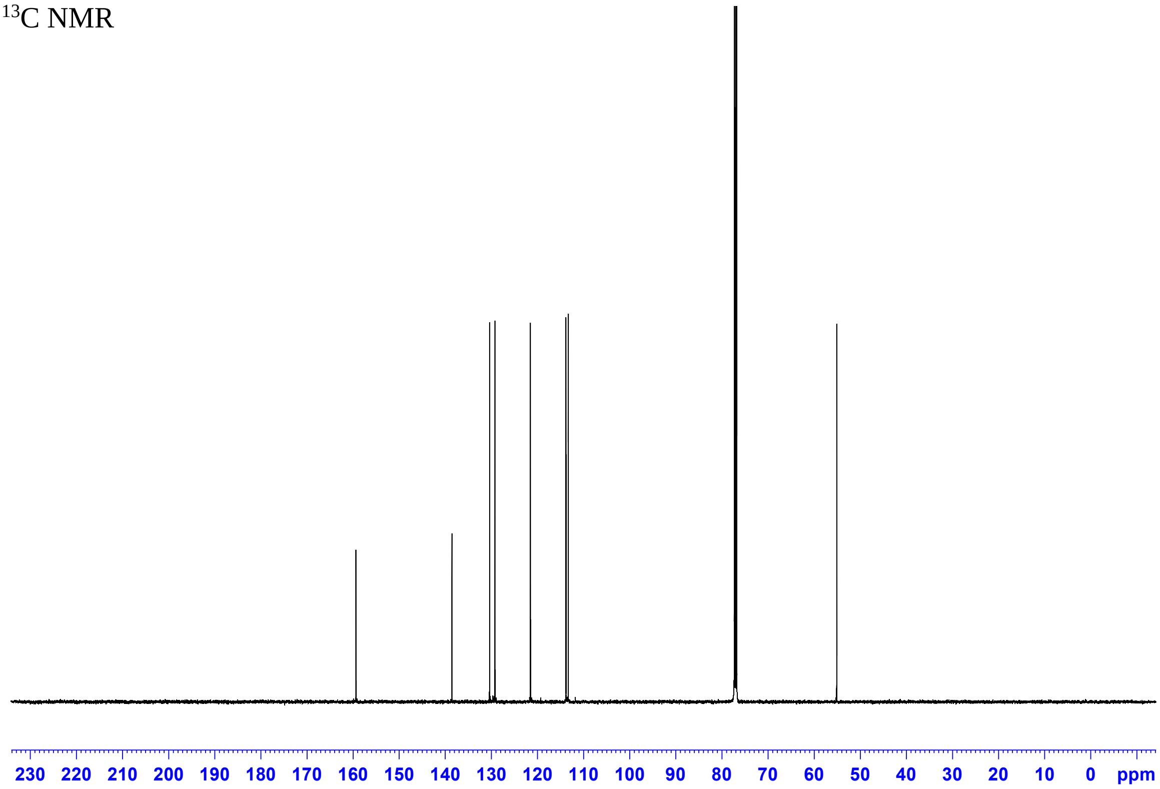

^{1}H NMR

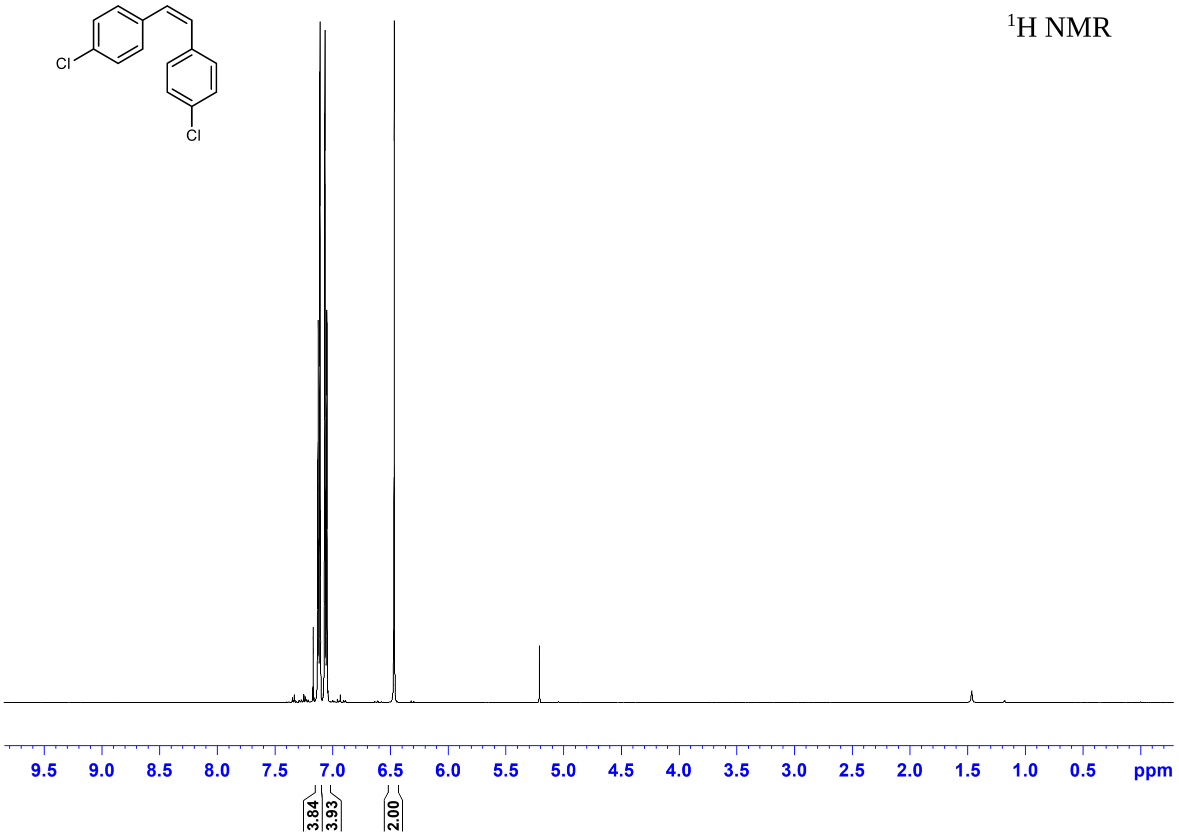

^{13}C NMR

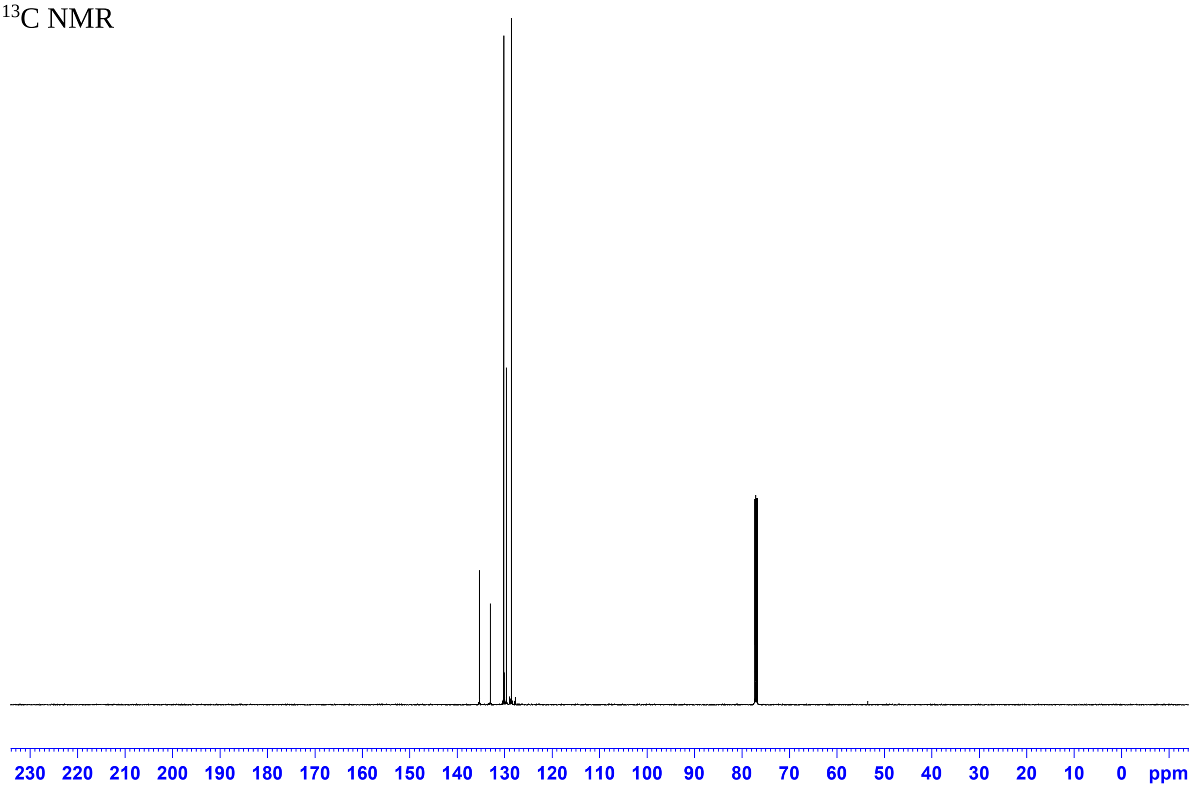

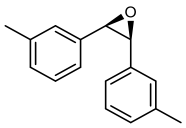

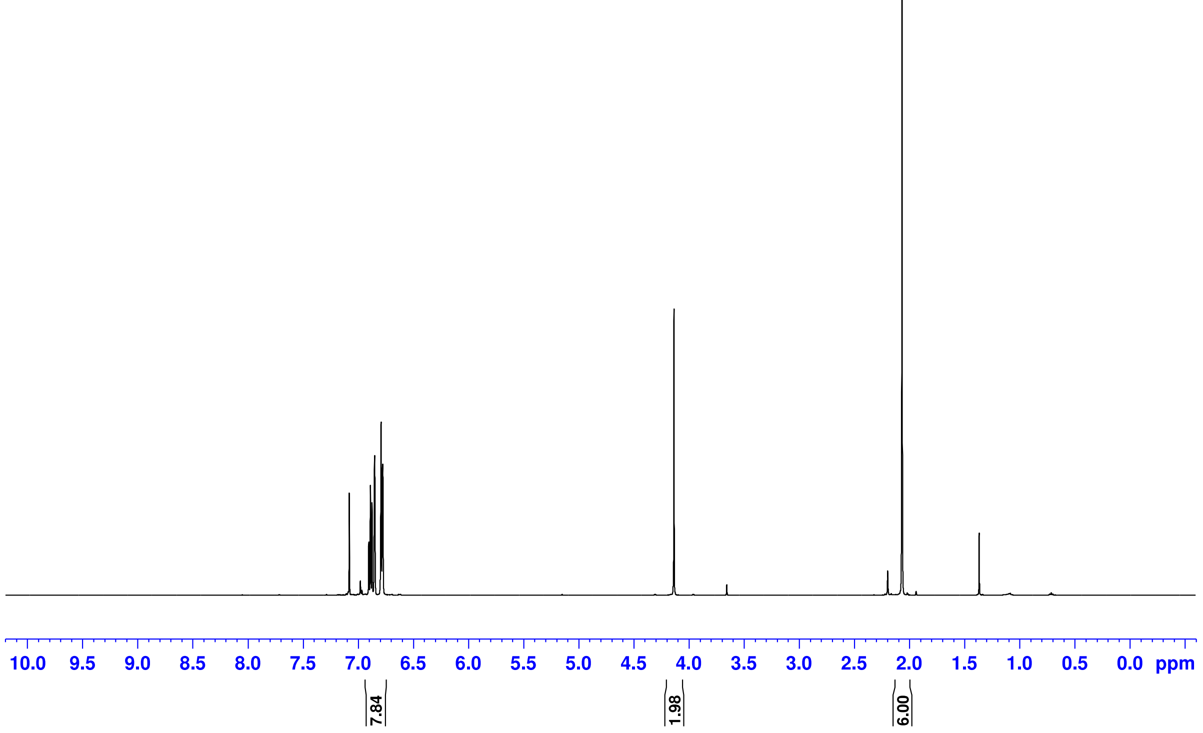

^{1}H NMR
10.0
9.5
9.0
8.5
8.0
7.5
7.0
6.5
6.0
5.5
5.0
4.5
4.0
3.5
3.0
2.5
2.0
1.5
1.0
0.5
0.0
ppm
7.84
1.98
6.00

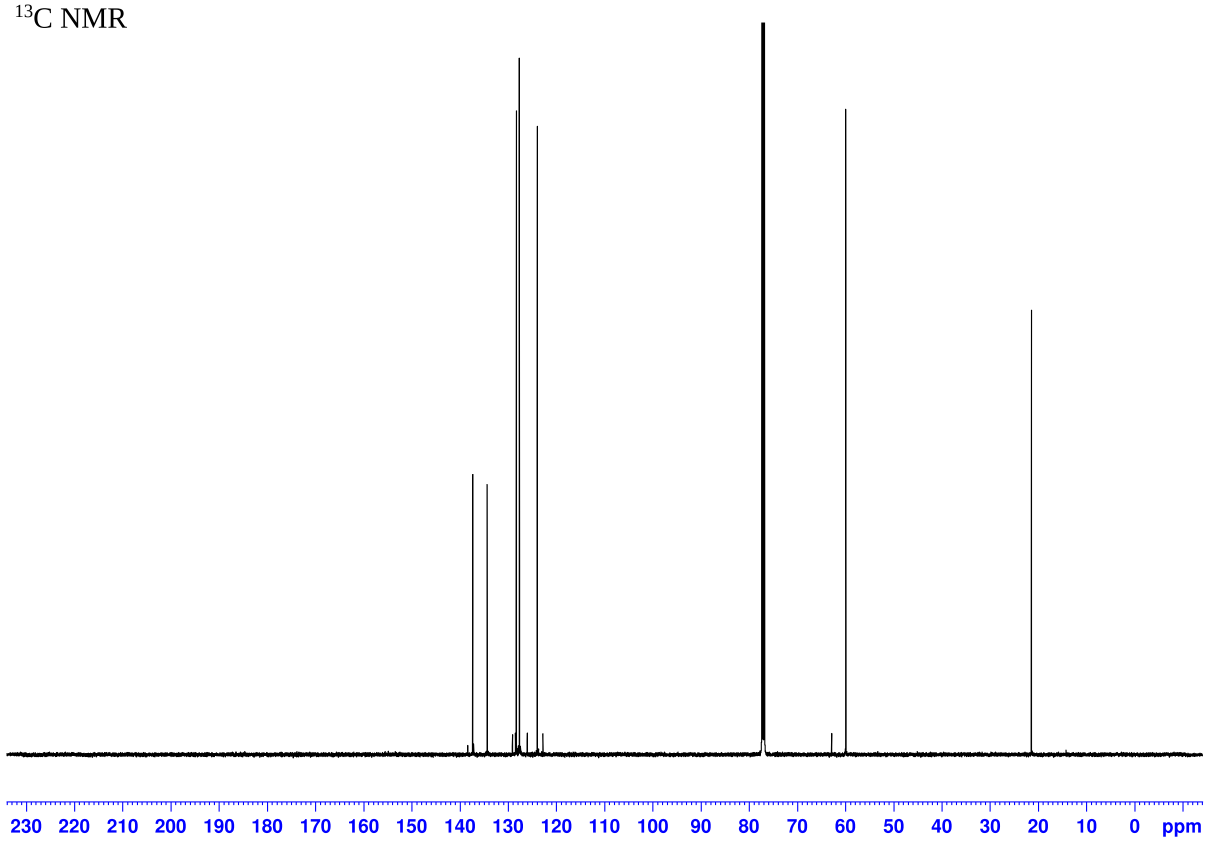

^{13}C NMR
230
220
210
200
190
180
170
160
150
140
130
120
110
100
90
80
70
60
50
40
30
20
10
0
ppm

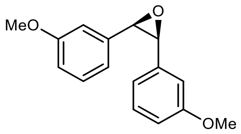

^{1}H NMR

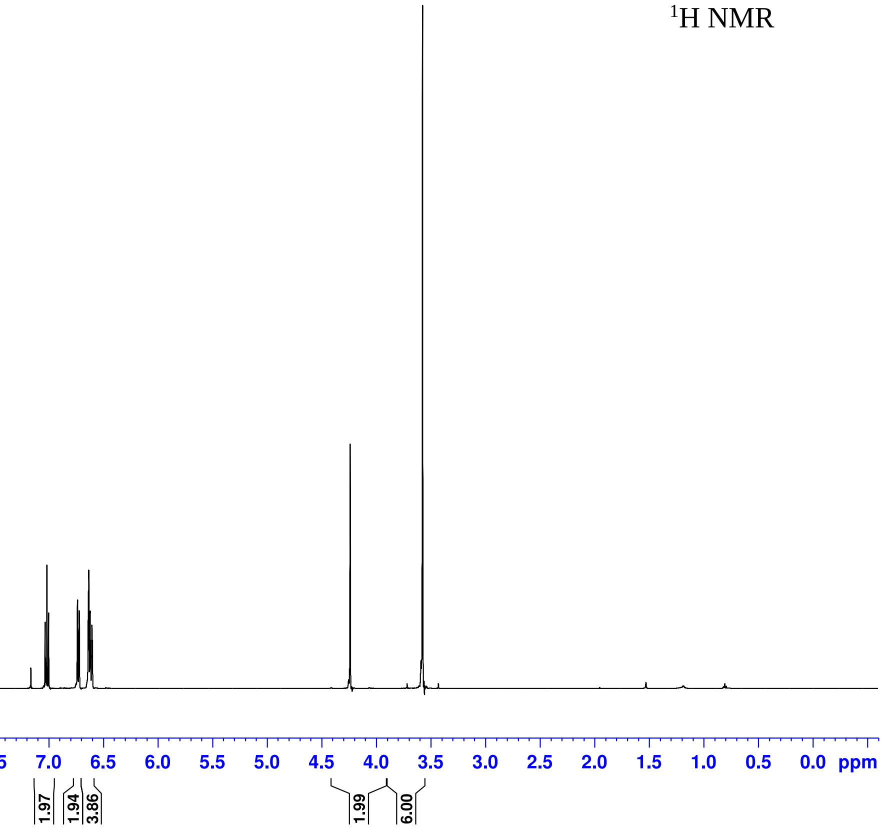

^{13}C NMR

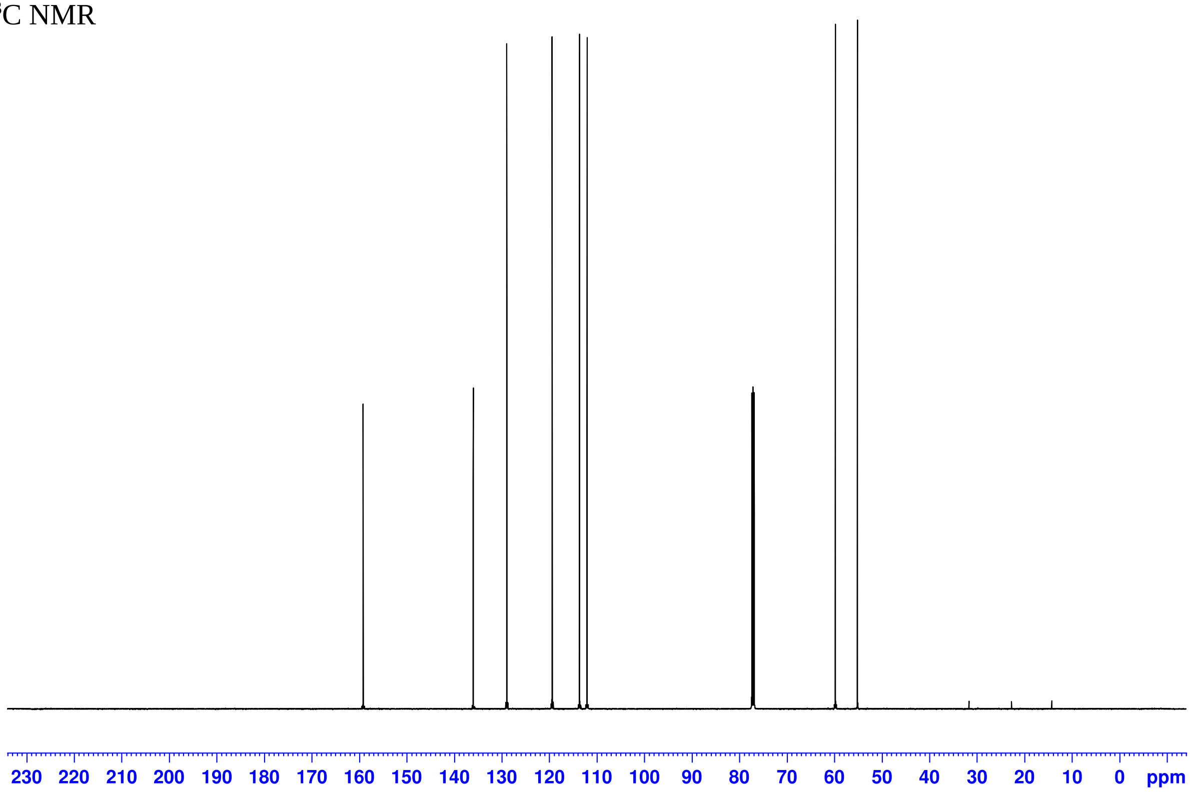

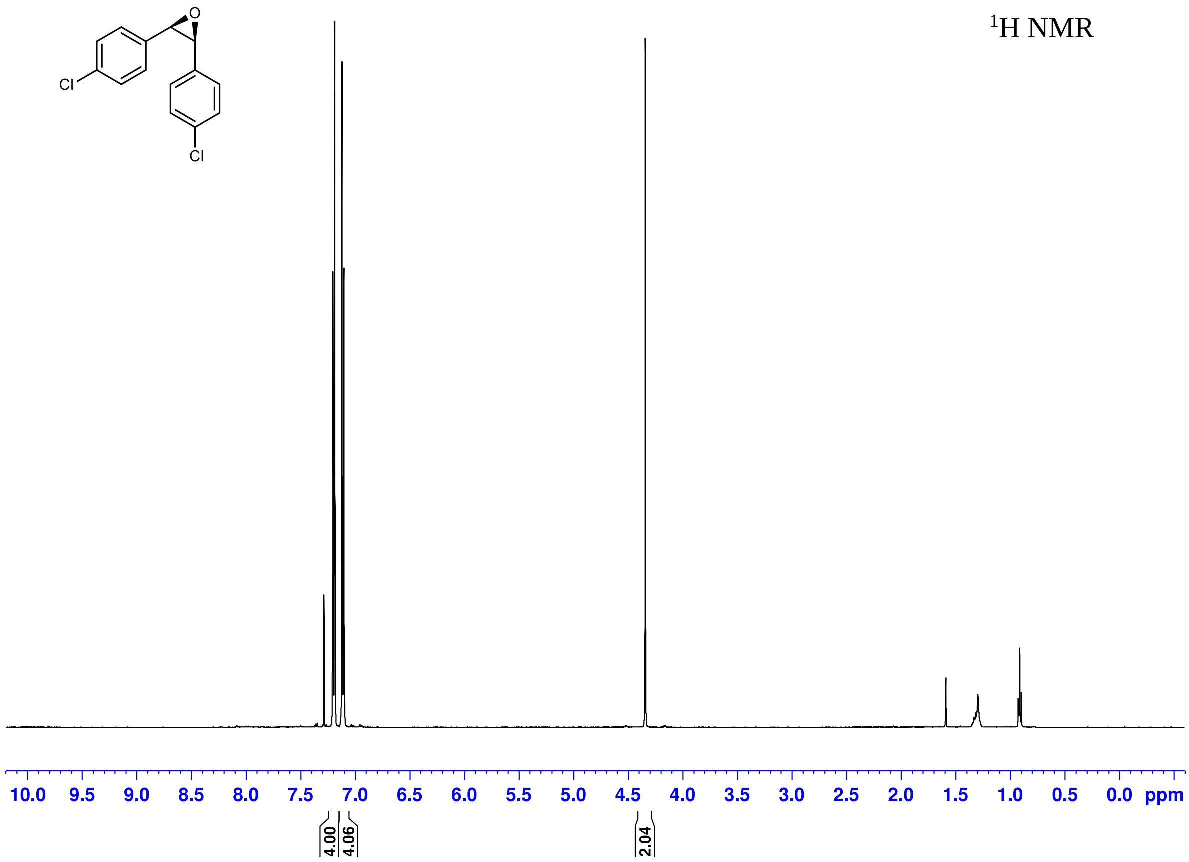
O
Cl
Cl
1H NMR
10.0 9.5 9.0 8.5 8.0 7.5 7.0 6.5 6.0 5.5 5.0 4.5 4.0 3.5 3.0 2.5 2.0 1.5 1.0 0.5 0.0 ppm
4.00
4.06
2.04

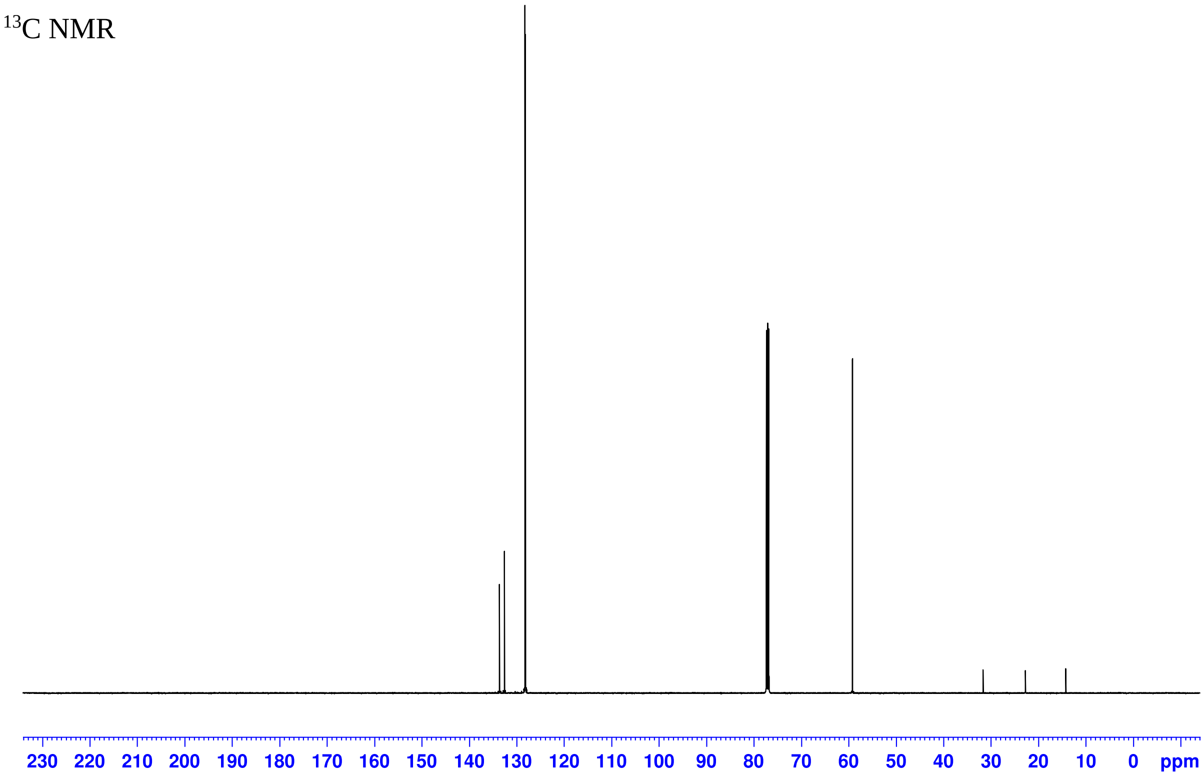
13C NMR
230 220 210 200 190 180 170 160 150 140 130 120 110 100 90 80 70 60 50 40 30 20 10 0 ppm

***rac*-2-(phenethylthio)-1,2-diphenylethan-1-ol**

^{1}H NMR

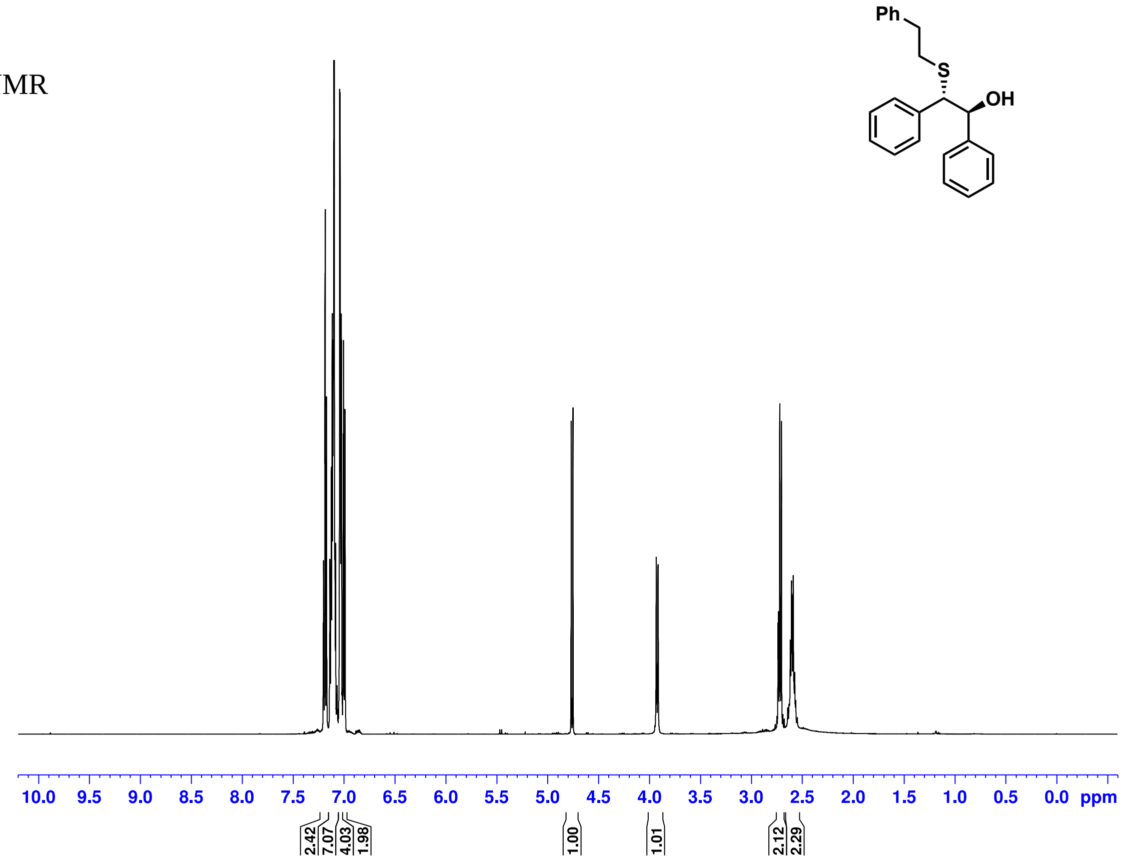

^{13}C NMR

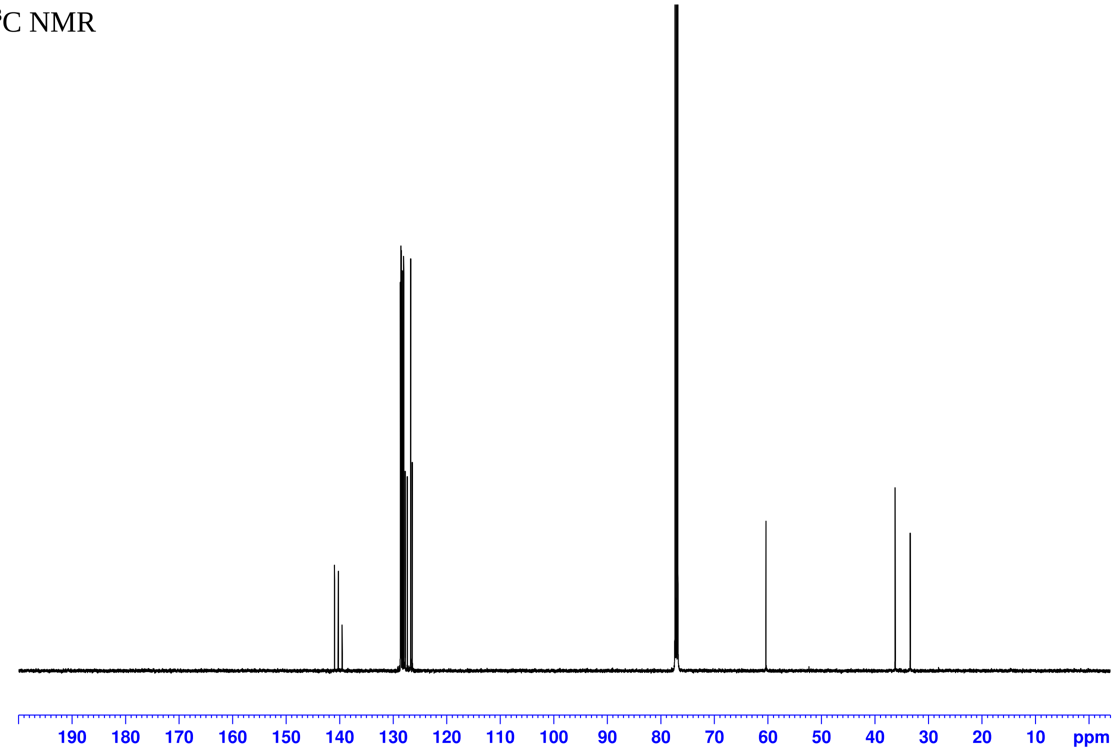

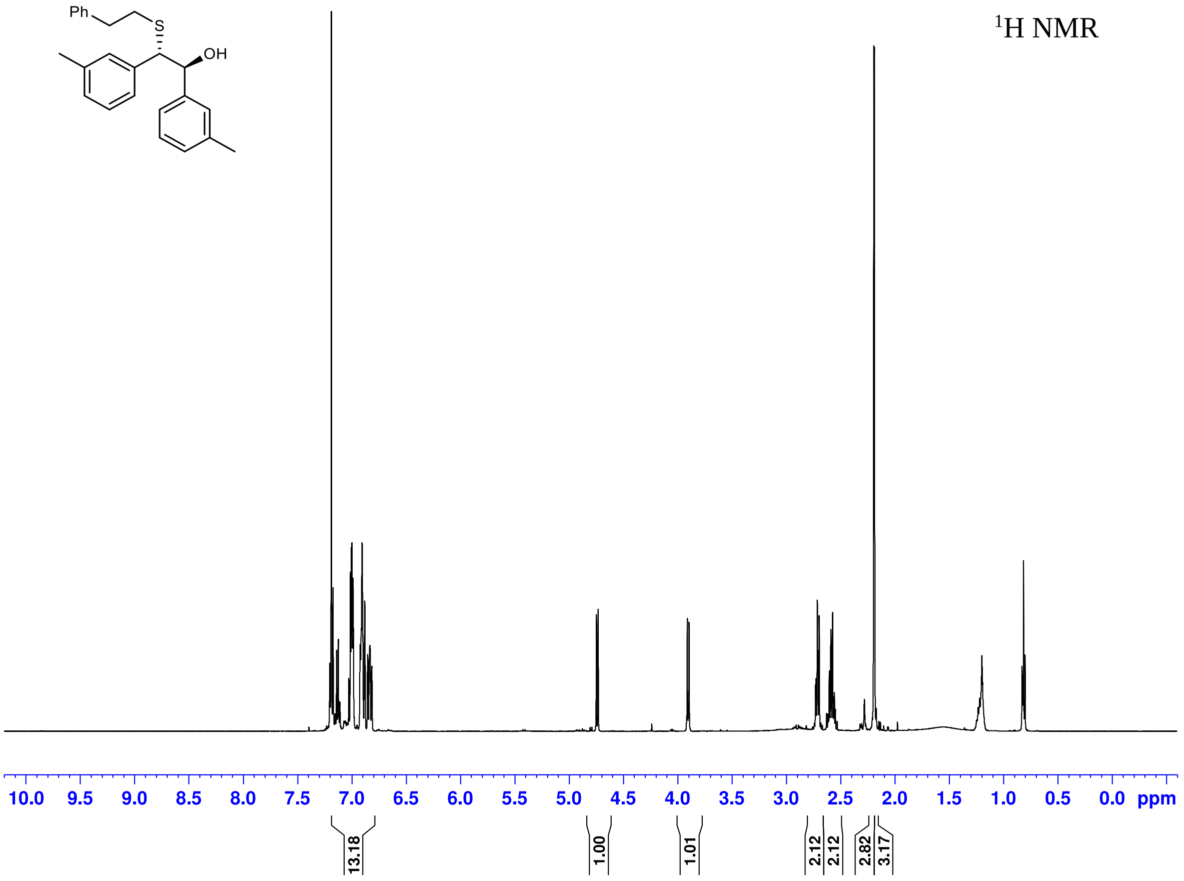

Ph
S
OH
1H NMR
10.0 9.5 9.0 8.5 8.0 7.5 7.0 6.5 6.0 5.5 5.0 4.5 4.0 3.5 3.0 2.5 2.0 1.5 1.0 0.5 0.0 ppm
13.18
1.00
1.01
2.12
2.12
2.82
3.17

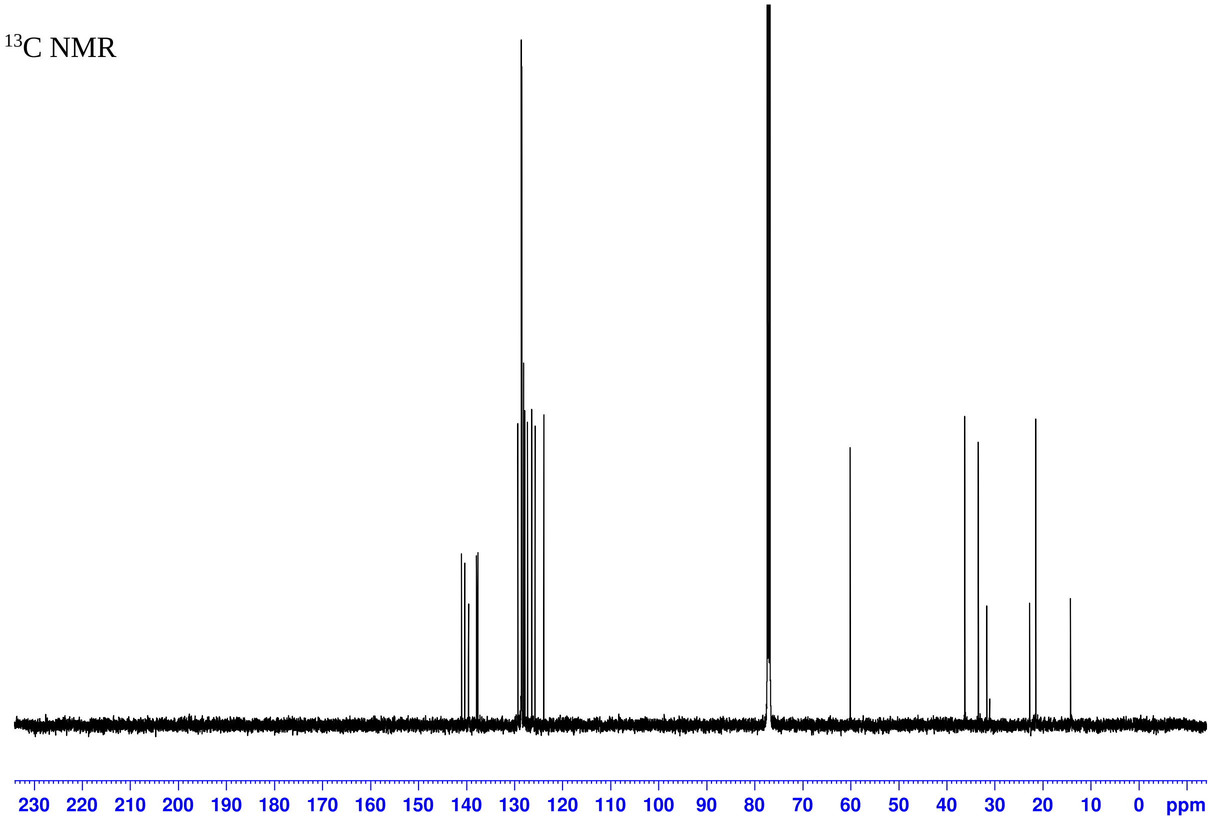

13C NMR
230 220 210 200 190 180 170 160 150 140 130 120 110 100 90 80 70 60 50 40 30 20 10 0 ppm

^{1}H NMR

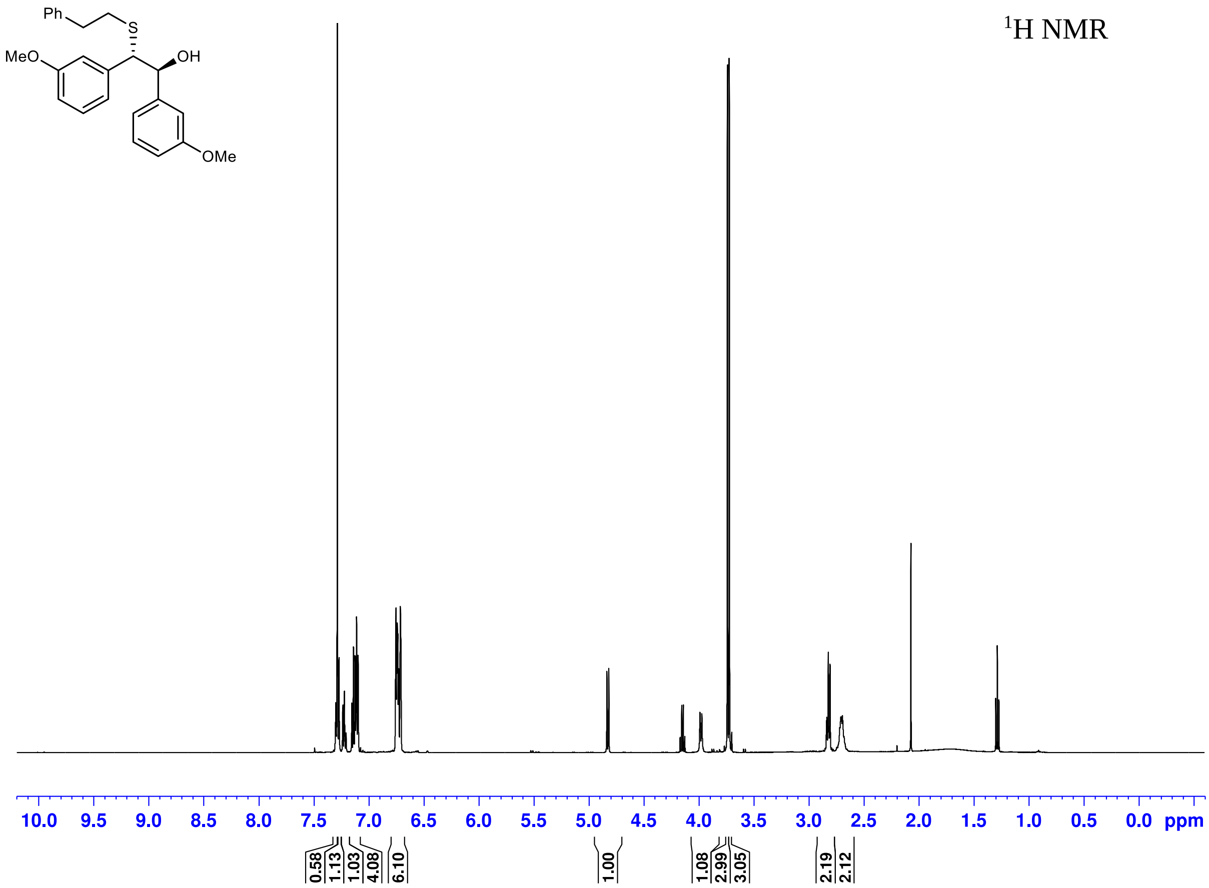

^{13}C NMR

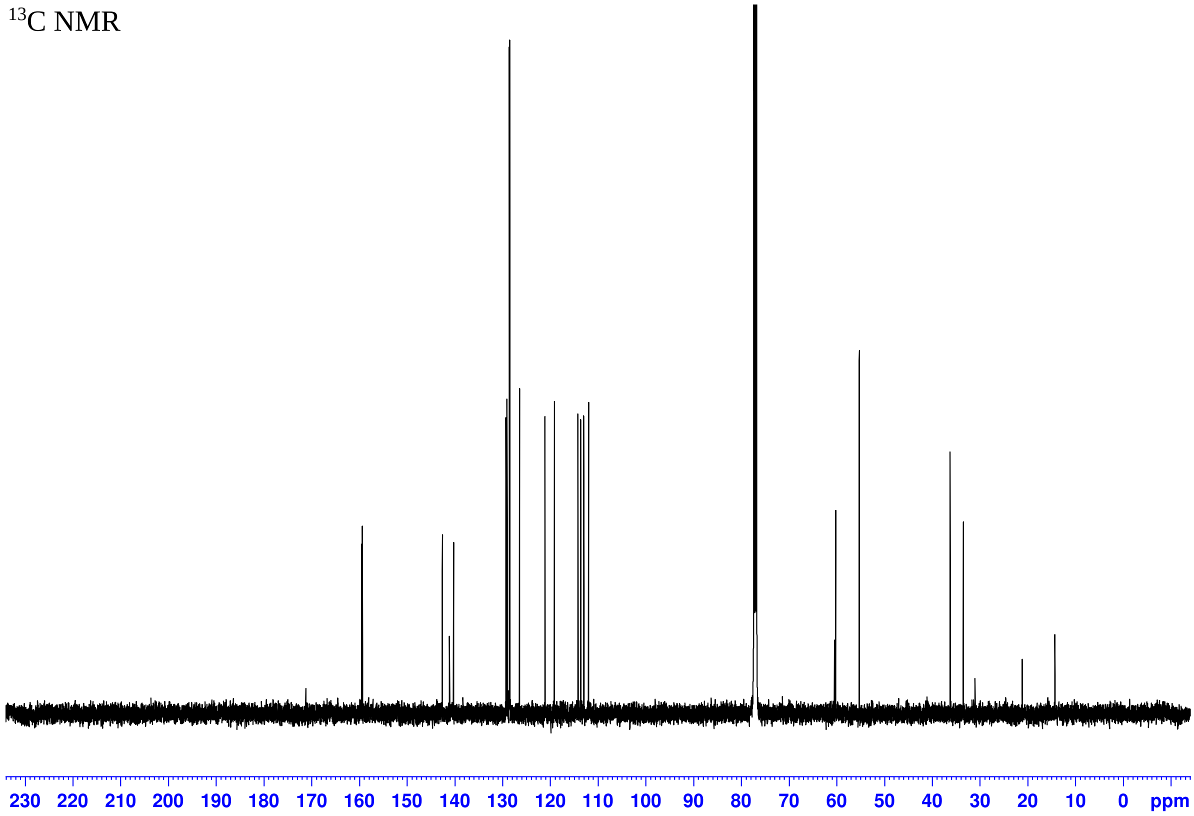

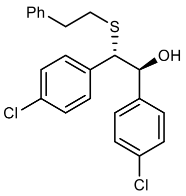

^{1}H NMR

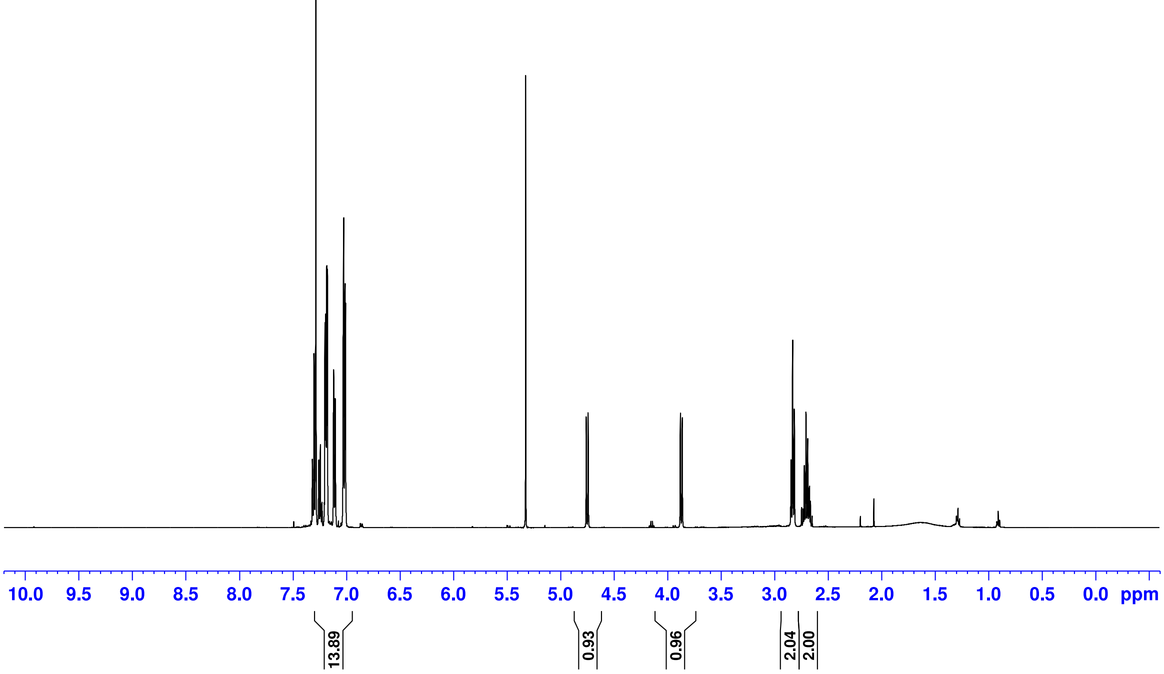

^{13}C NMR

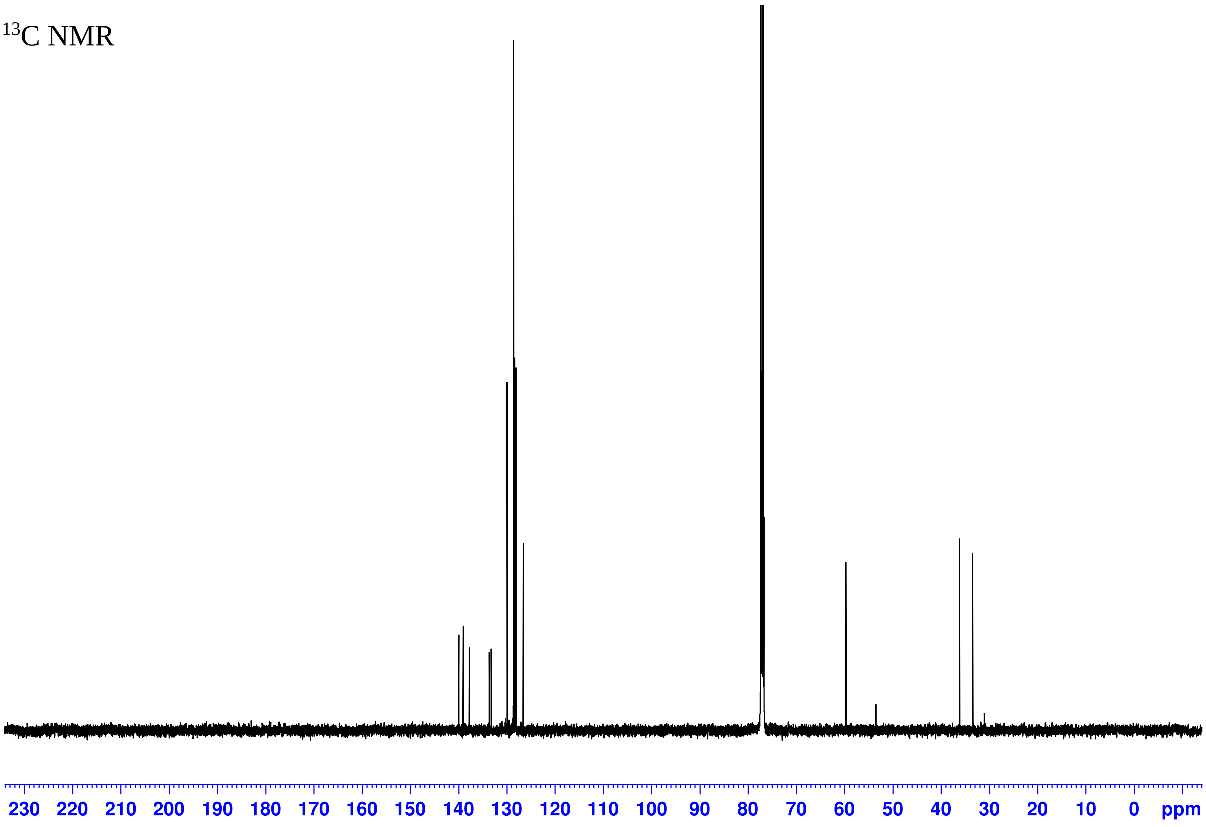

***rac*-2-bromo-1,2-diphenylethyl(phenethyl)sulfane**

^{1}H NMR

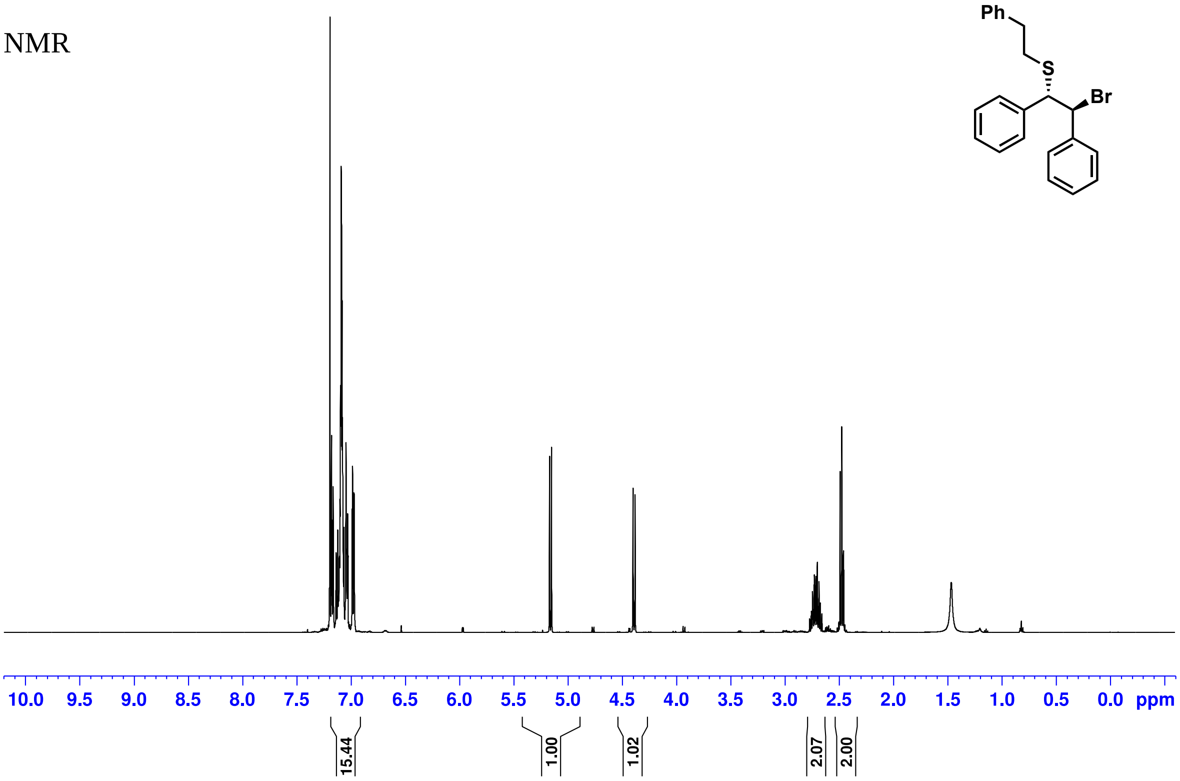

^{13}C NMR

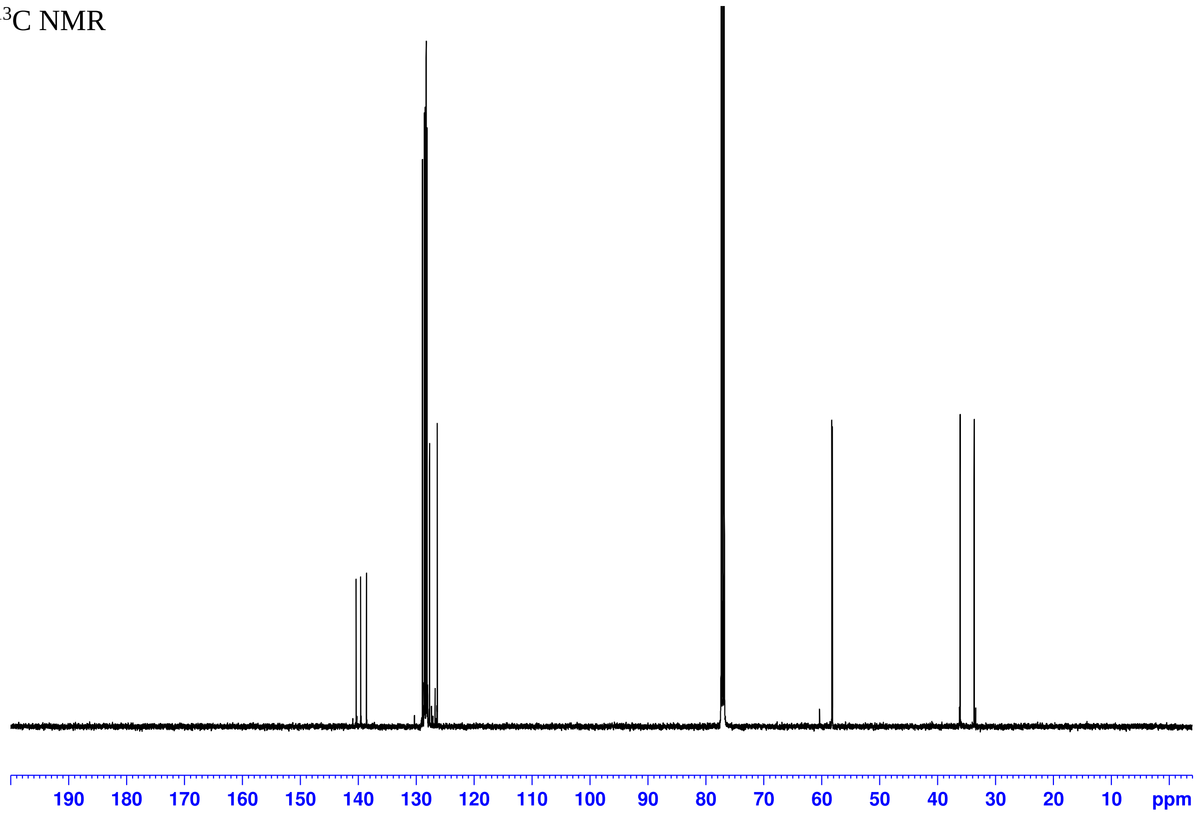

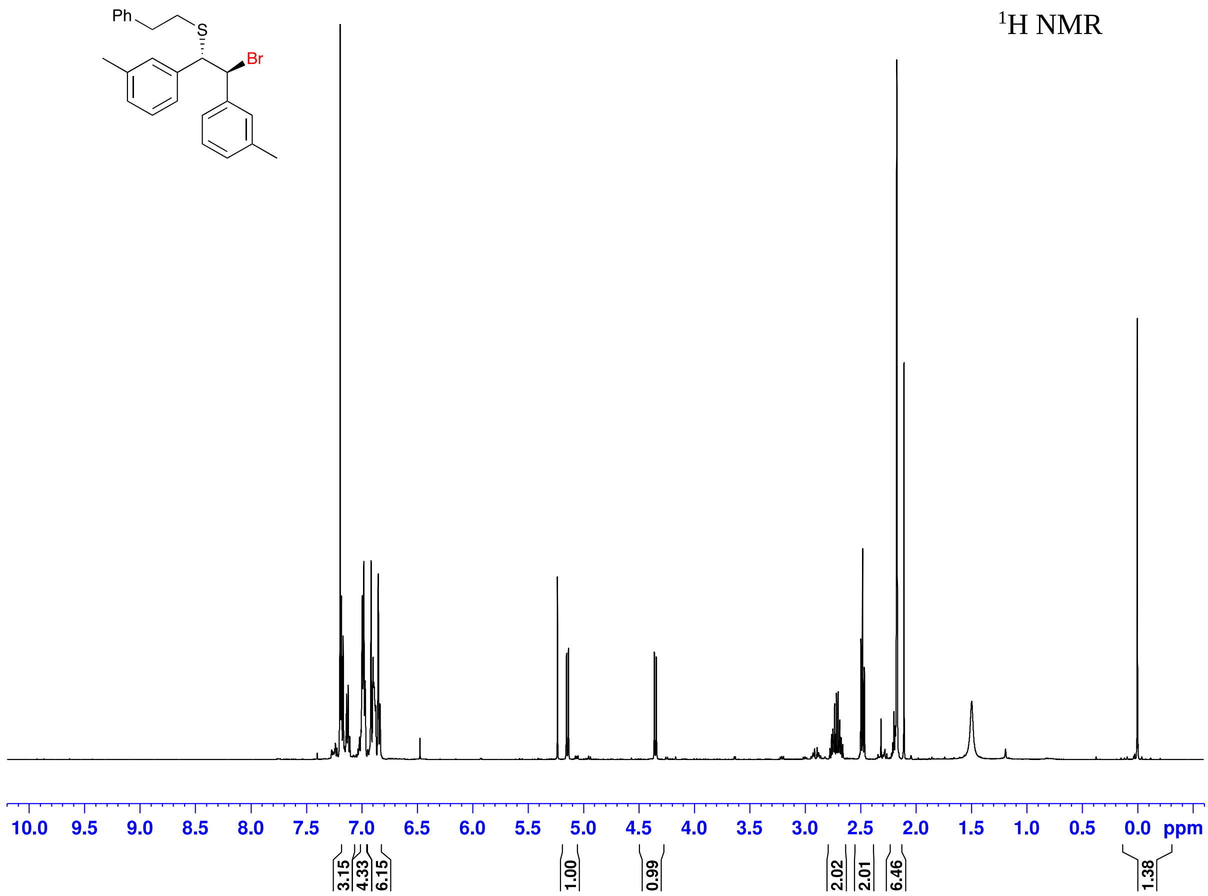
Ph
S
Br
^{1}H NMR
10.0 9.5 9.0 8.5 8.0 7.5 7.0 6.5 6.0 5.5 5.0 4.5 4.0 3.5 3.0 2.5 2.0 1.5 1.0 0.5 0.0 ppm
3.15 4.33 6.15 1.00 0.99 2.02 2.01 6.46 1.38

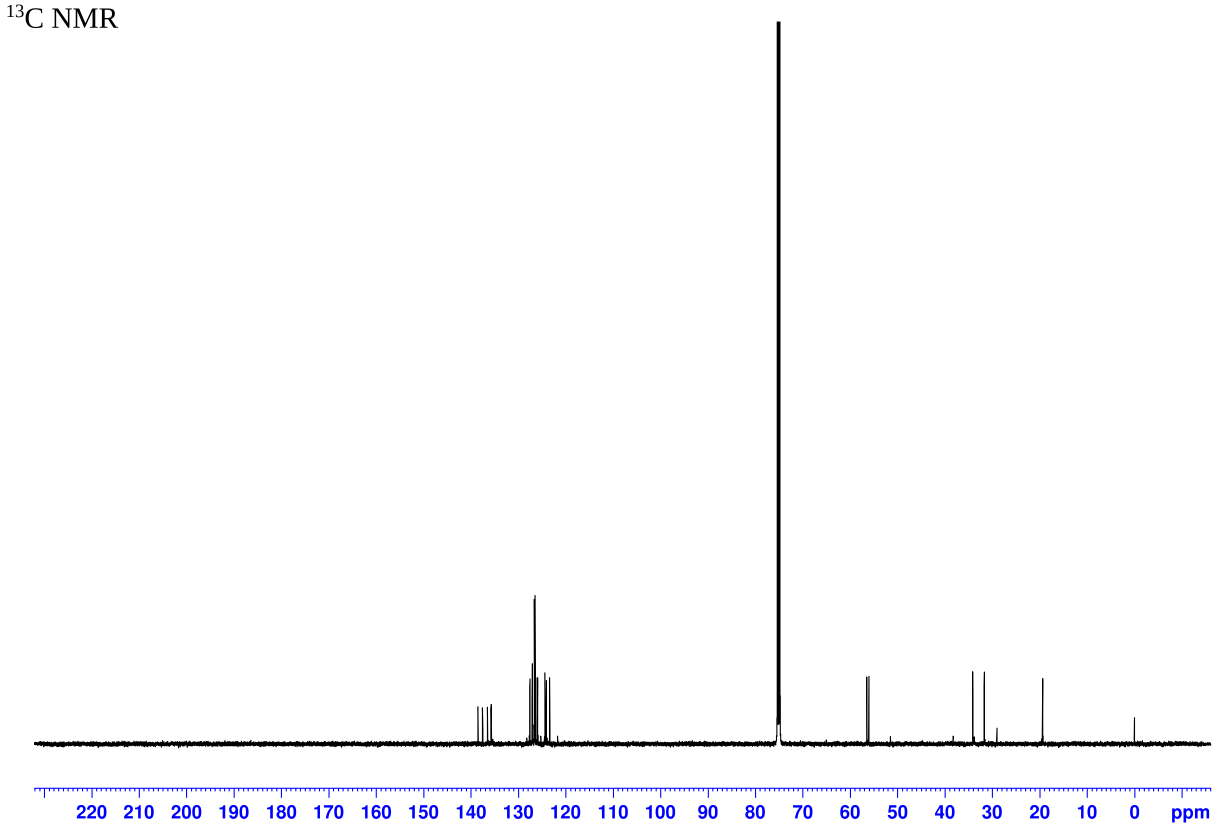
^{13}C NMR
220 210 200 190 180 170 160 150 140 130 120 110 100 90 80 70 60 50 40 30 20 10 0 ppm

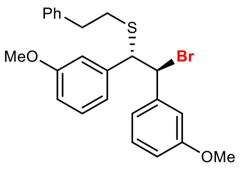

^{1}H NMR

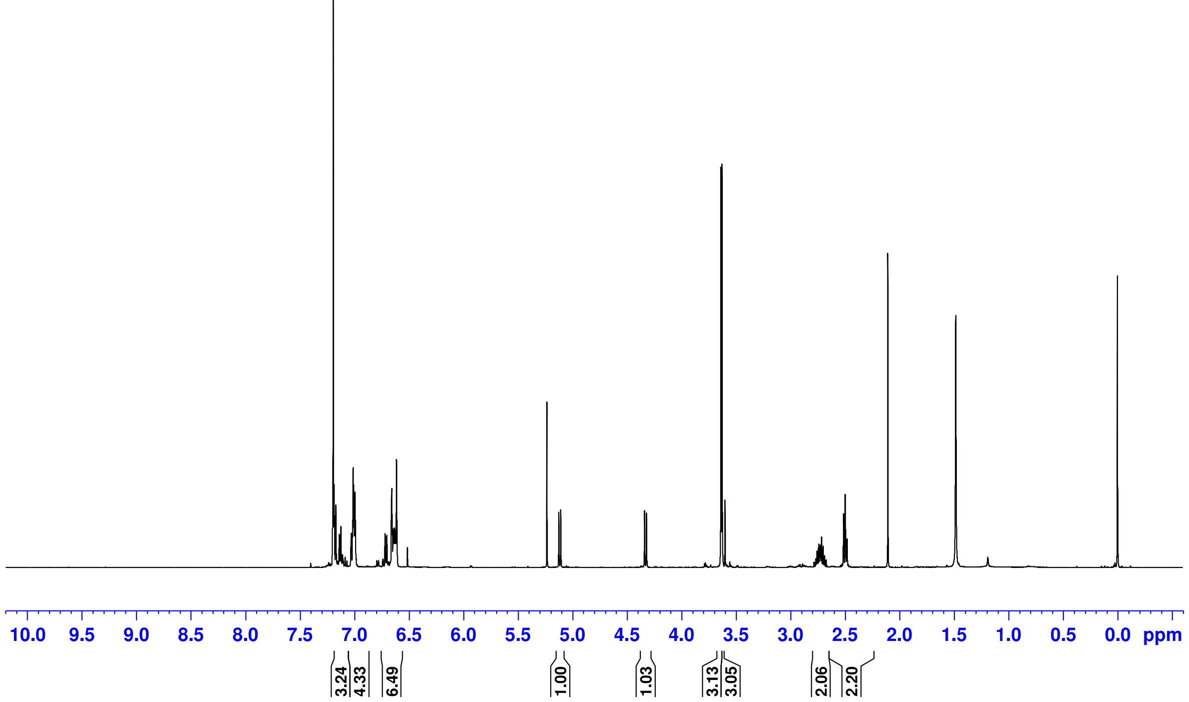

^{13}C NMR

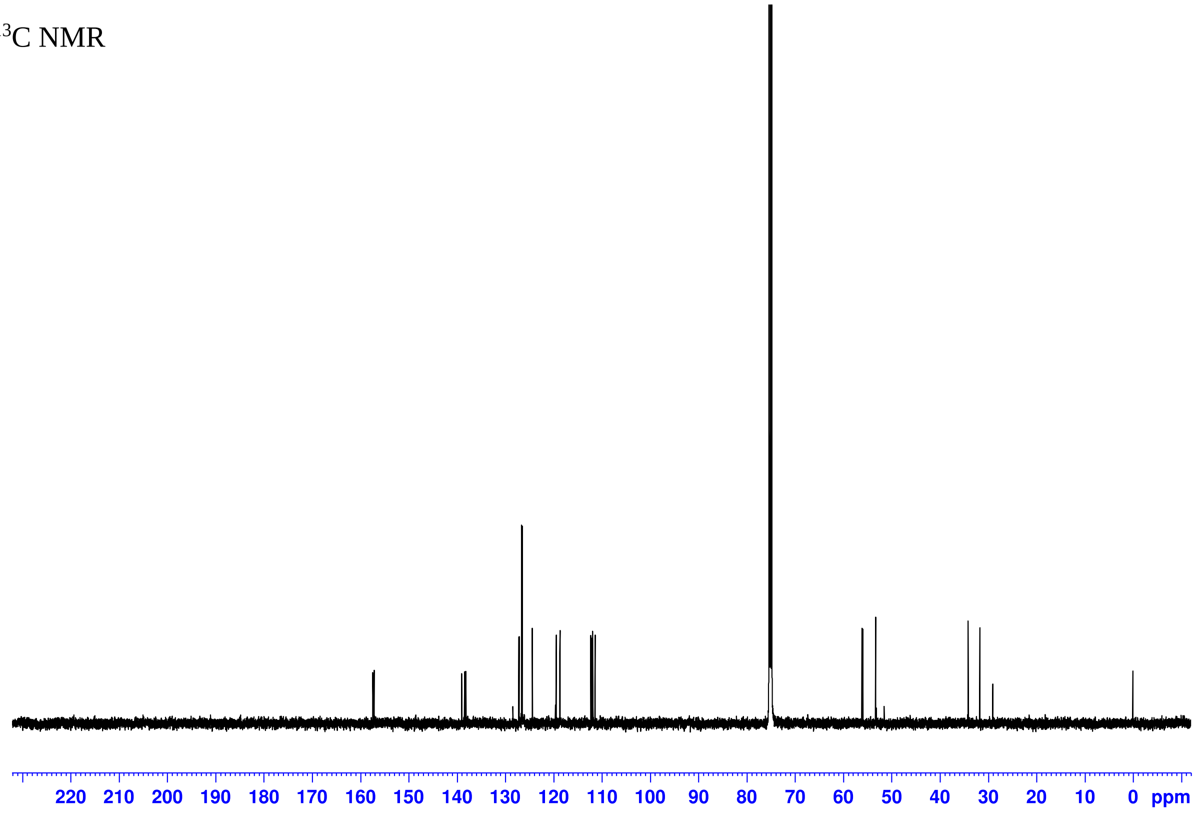

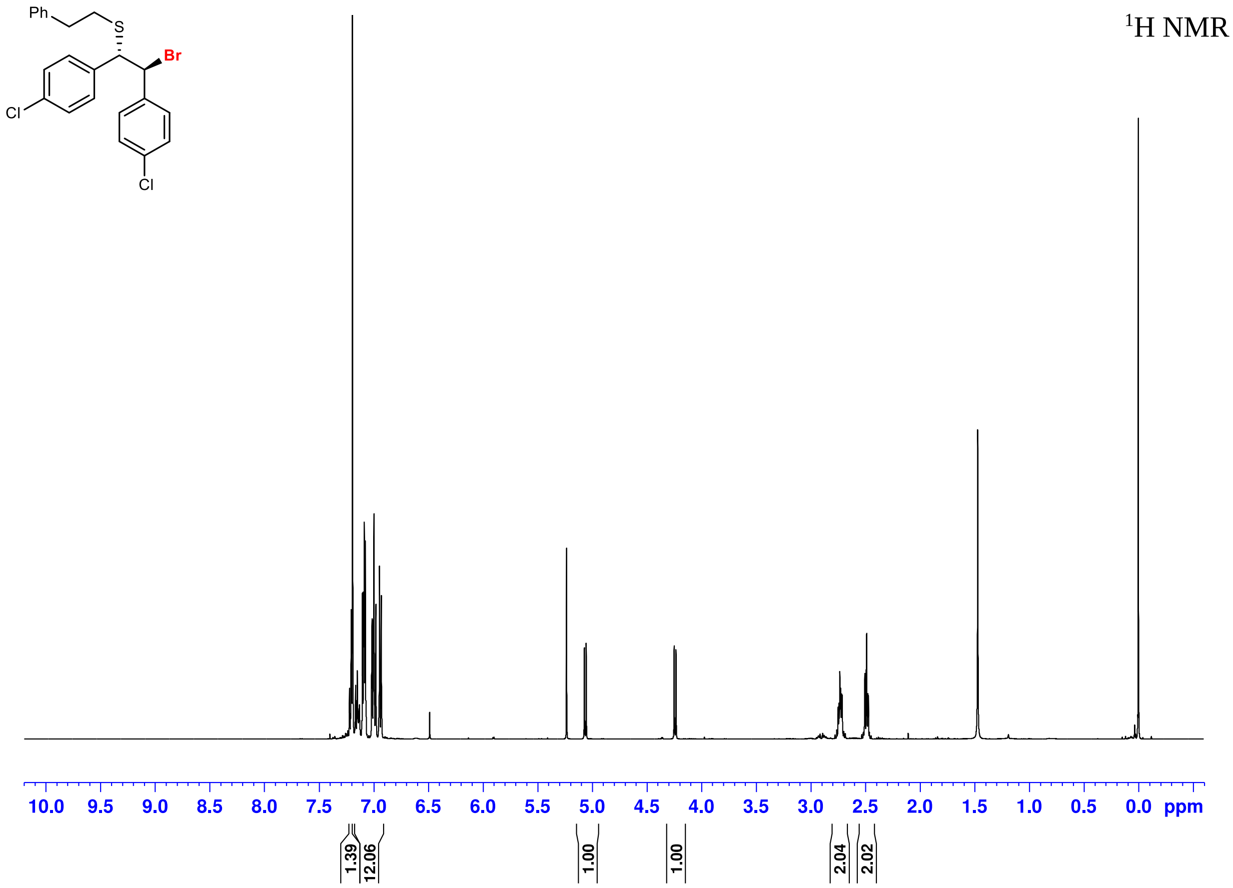
Ph
S
Br
Cl
Cl
^{1}H NMR
10.0 9.5 9.0 8.5 8.0 7.5 7.0 6.5 6.0 5.5 5.0 4.5 4.0 3.5 3.0 2.5 2.0 1.5 1.0 0.5 0.0 ppm
1.39
12.06
1.00
1.00
2.04
2.02

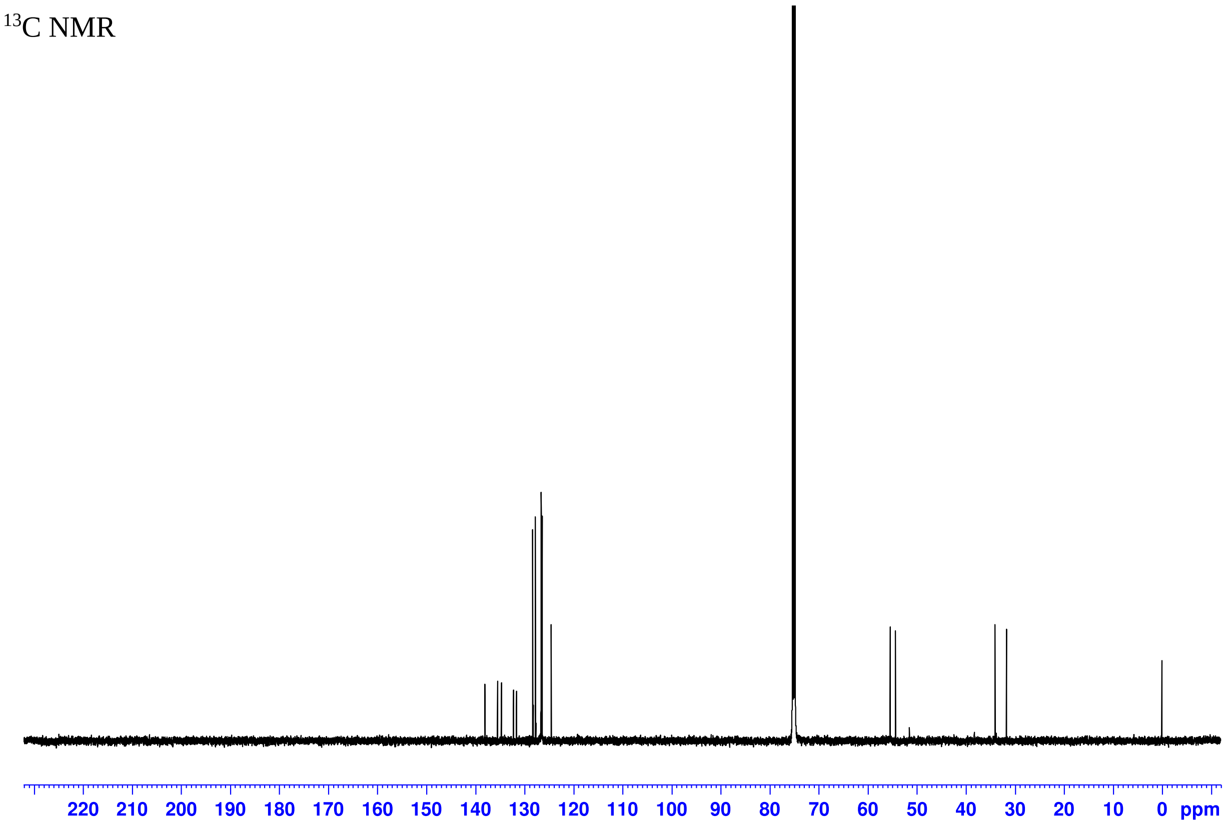
^{13}C NMR
220 210 200 190 180 170 160 150 140 130 120 110 100 90 80 70 60 50 40 30 20 10 0 ppm

***rac*-2-fluoro-1,2-diphenylethyl)(phenethyl)sulfane**

^{1}H NMR

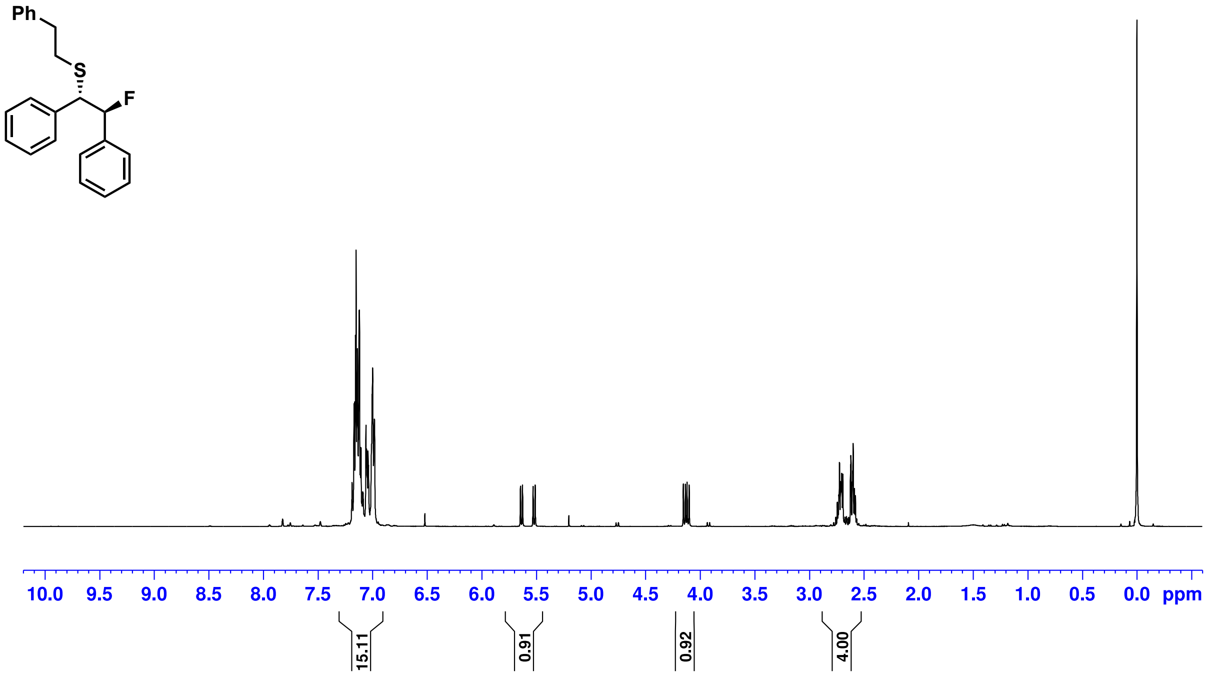

^{13}C NMR

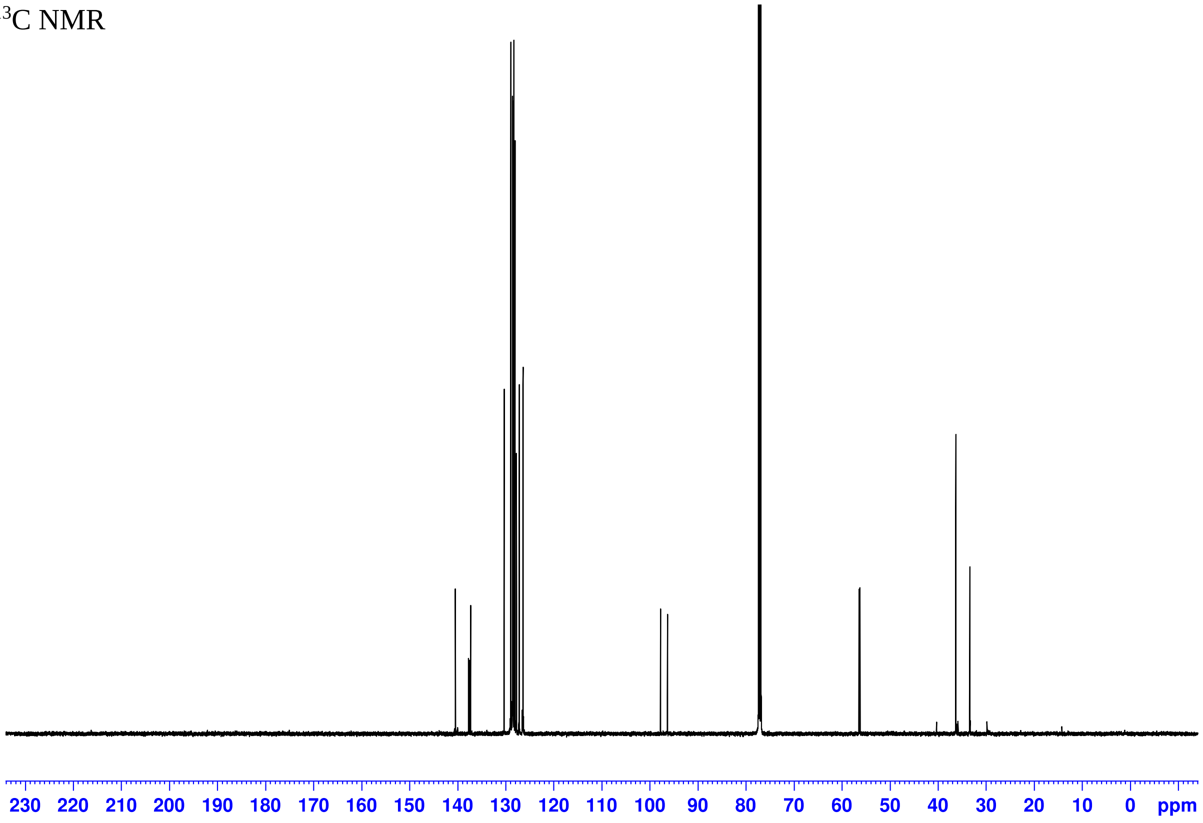

^{19}F NMR

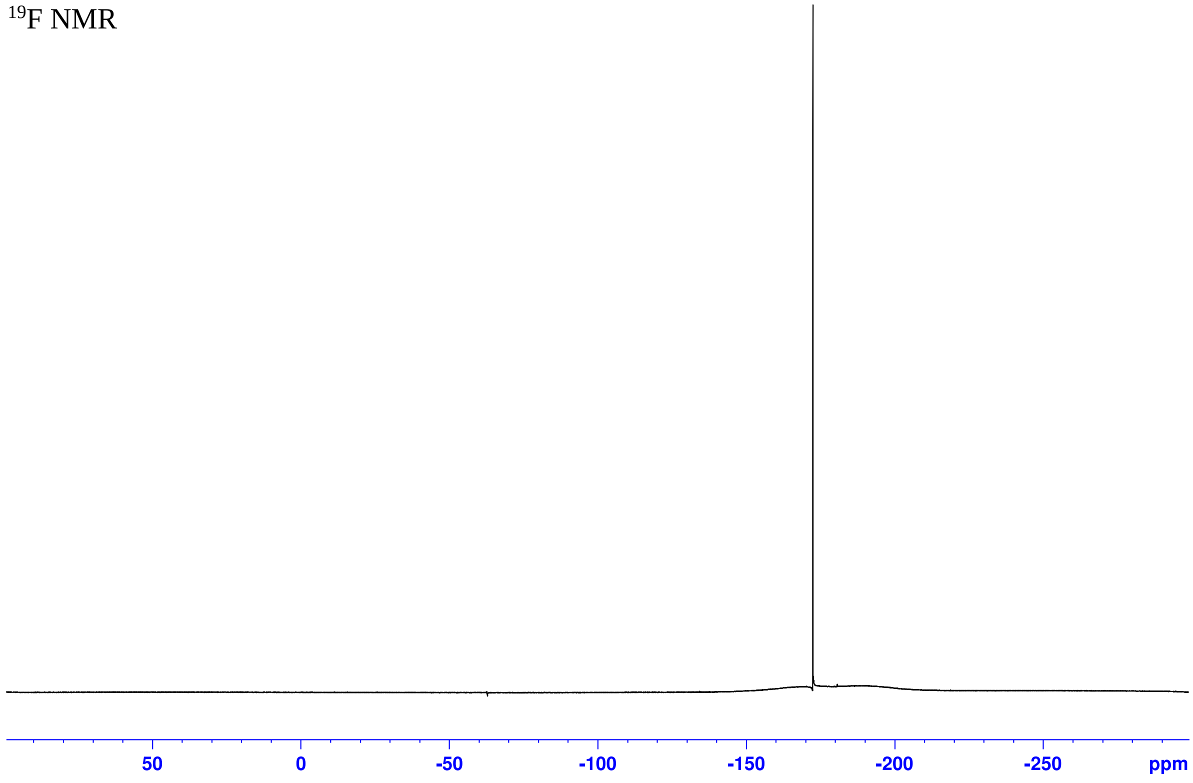

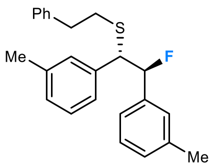

^{1}H NMR

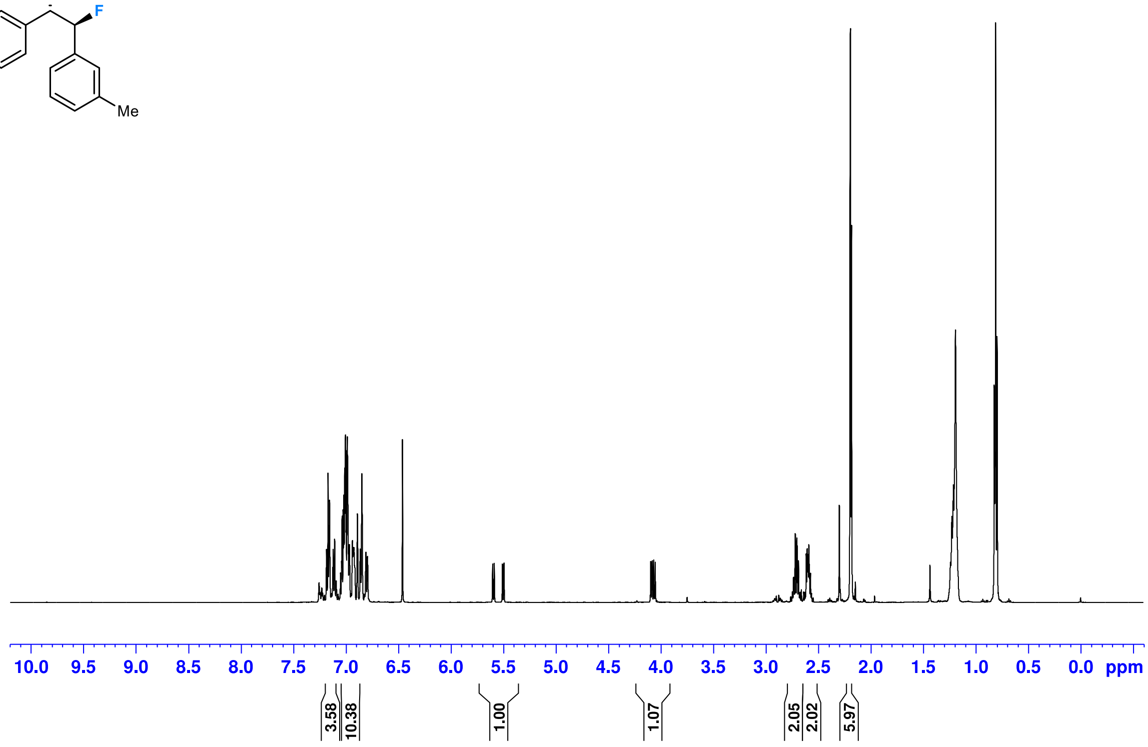

^{13}C NMR

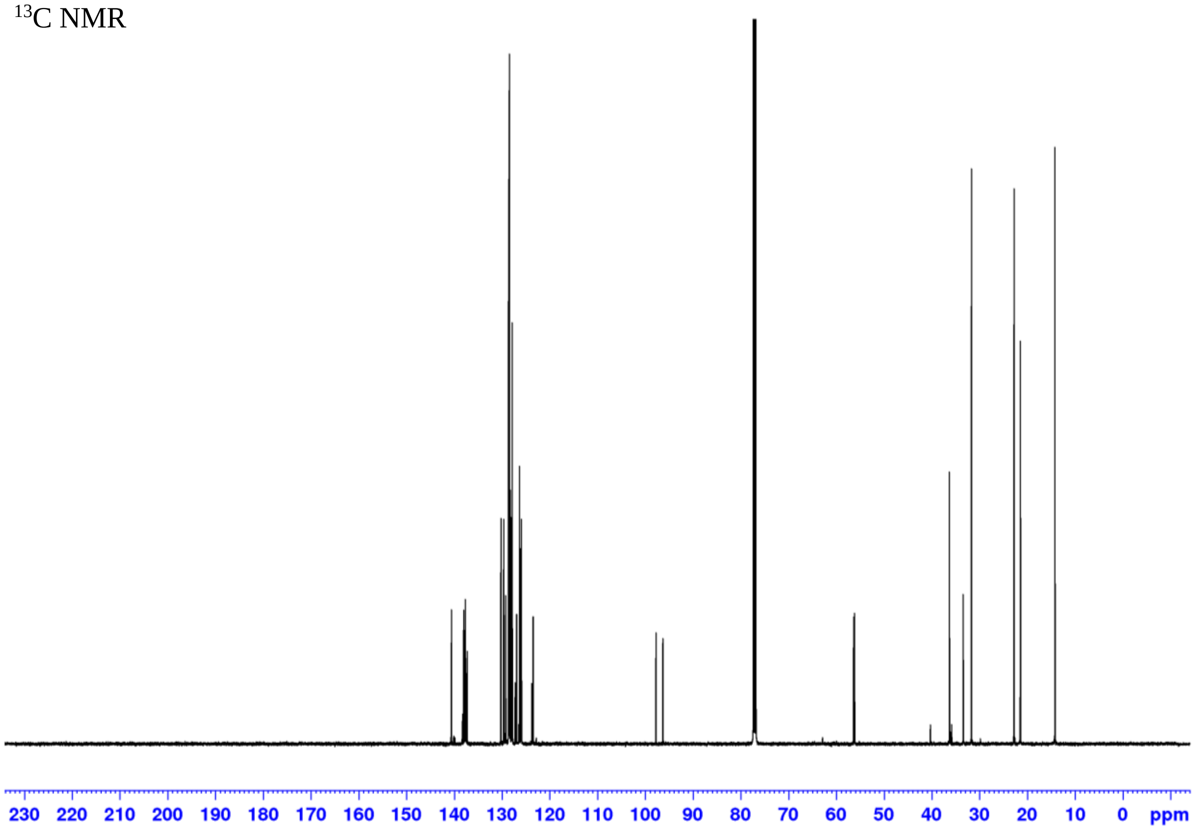

^{19}F NMR

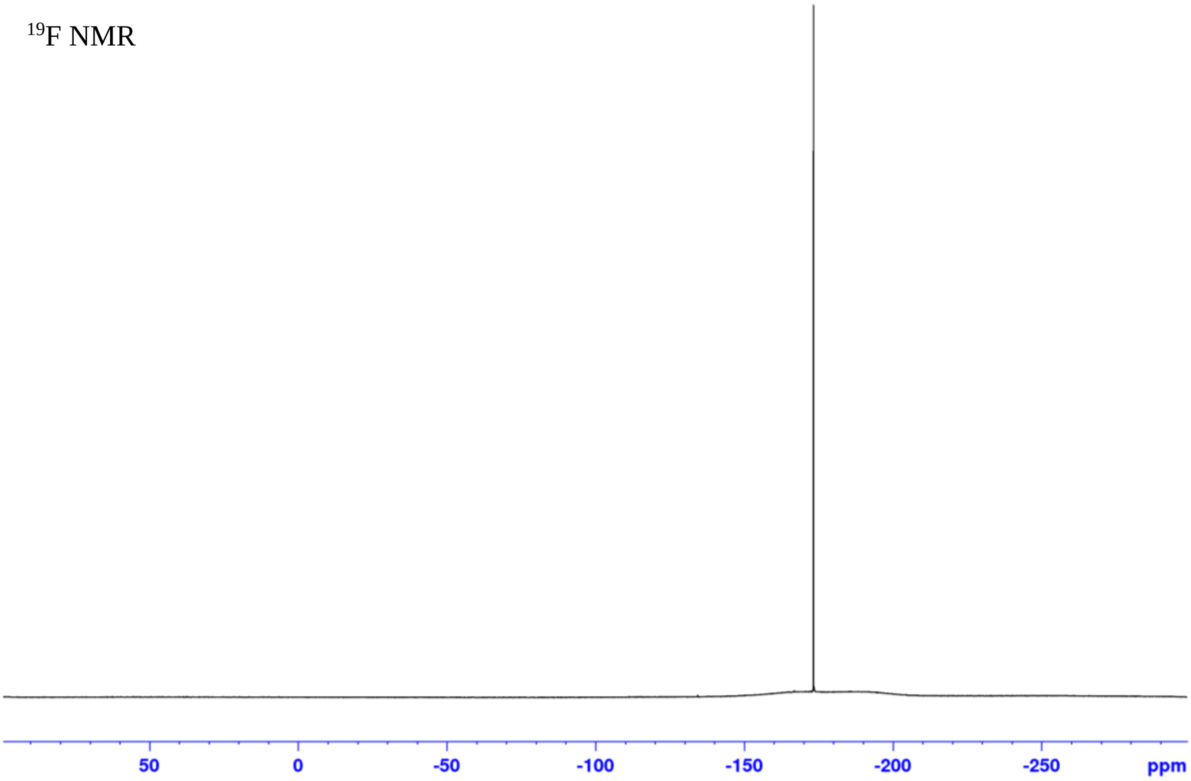

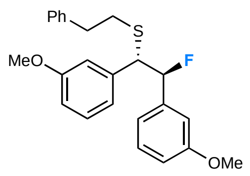
Ph
S
MeO
F
OMe

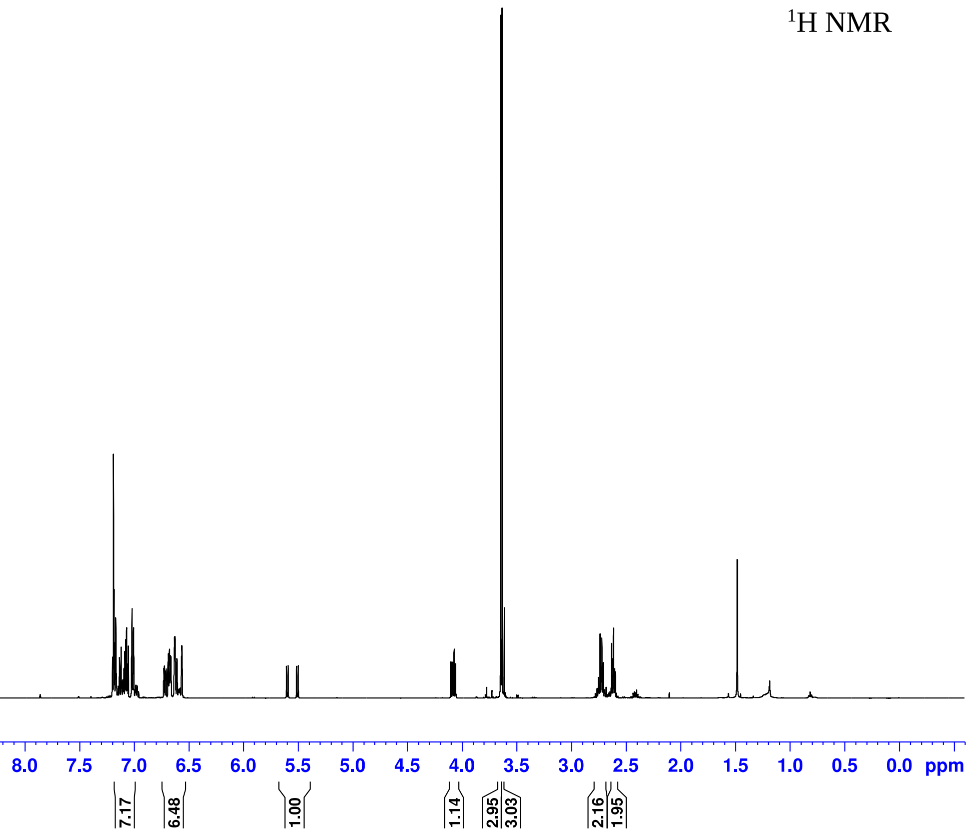
^{1}H NMR
10.0 9.5 9.0 8.5 8.0 7.5 7.0 6.5 6.0 5.5 5.0 4.5 4.0 3.5 3.0 2.5 2.0 1.5 1.0 0.5 0.0 ppm
7.17
6.48
1.00
1.14
2.95
3.03
2.16
1.95

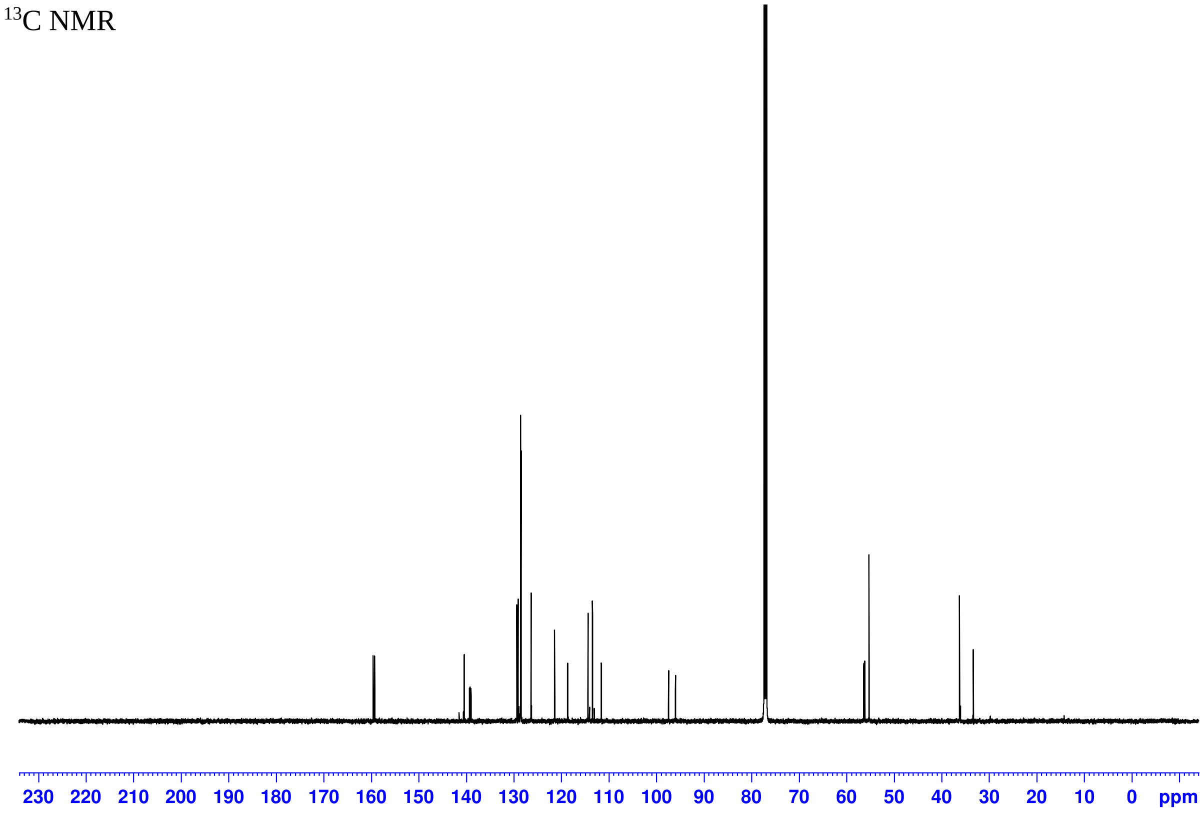
^{13}C NMR
230 220 210 200 190 180 170 160 150 140 130 120 110 100 90 80 70 60 50 40 30 20 10 0 ppm

^{19}F NMR

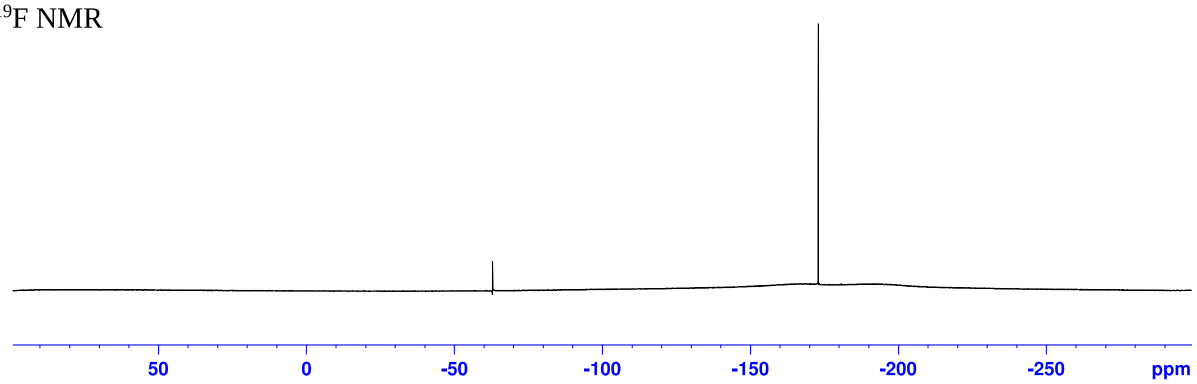

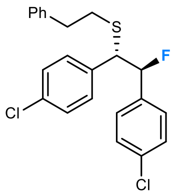

^{1}H NMR

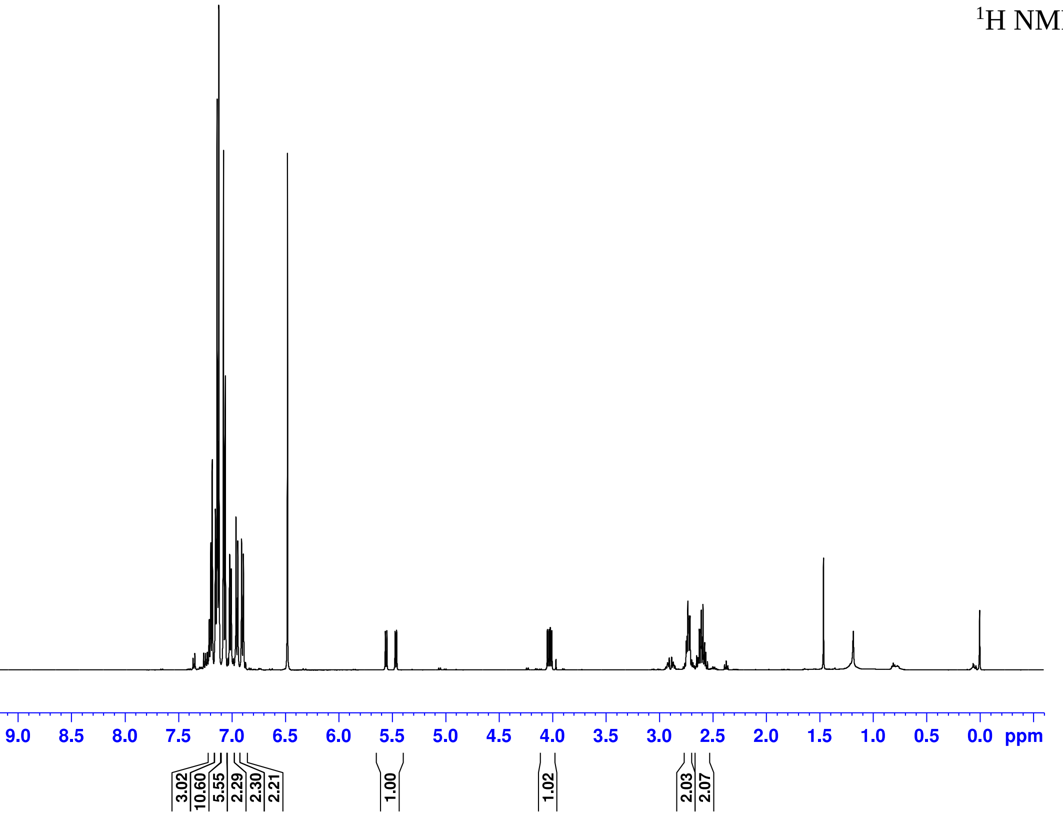

^{13}C NMR

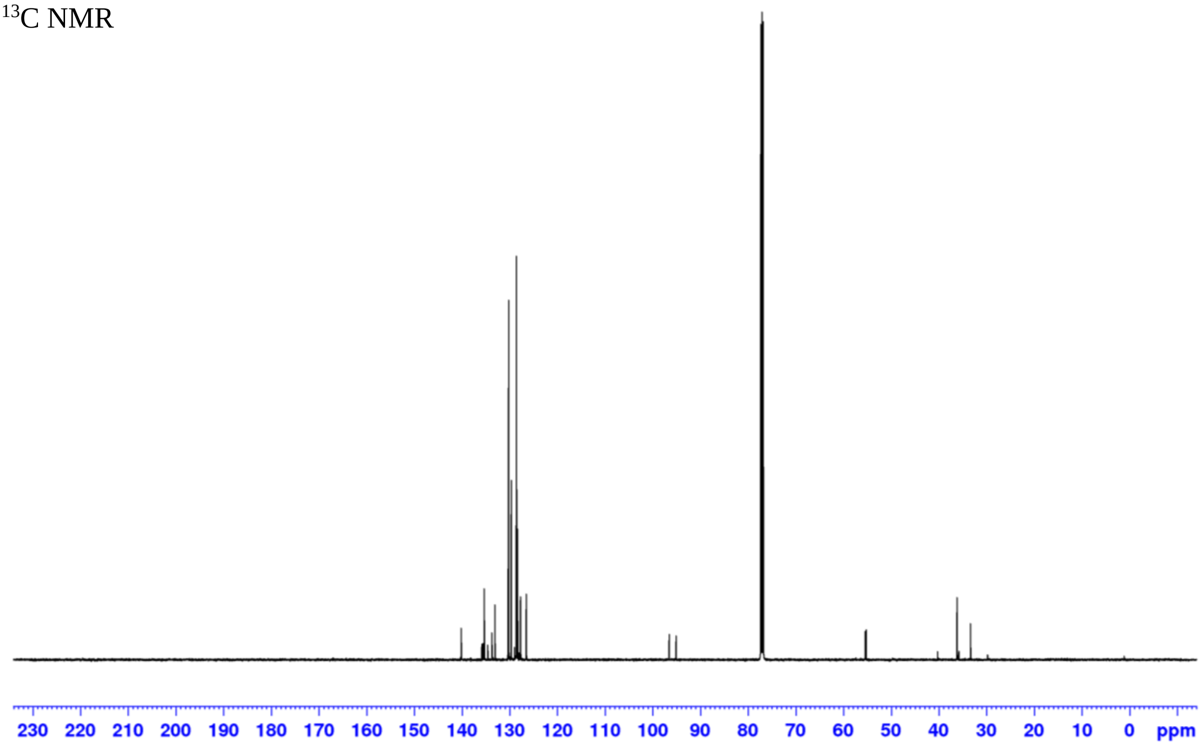

^{19}F NMR

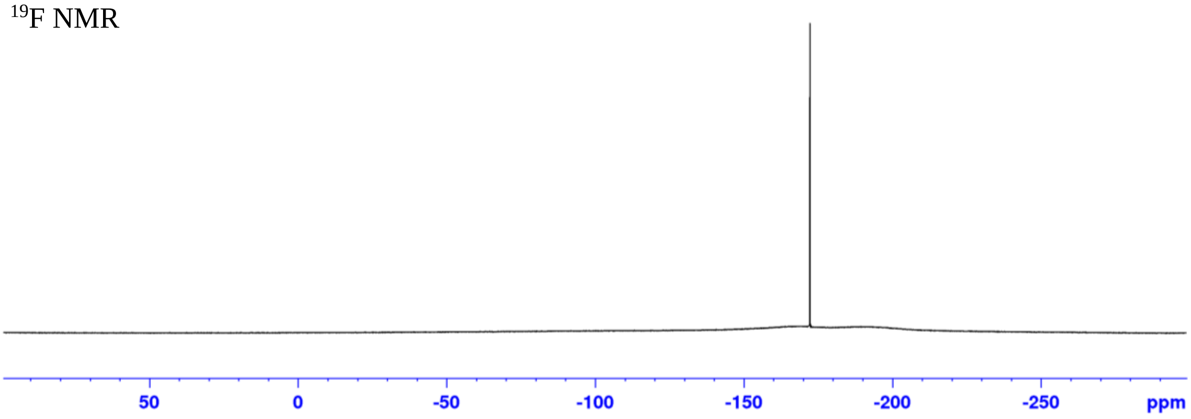

^{1}H NMR

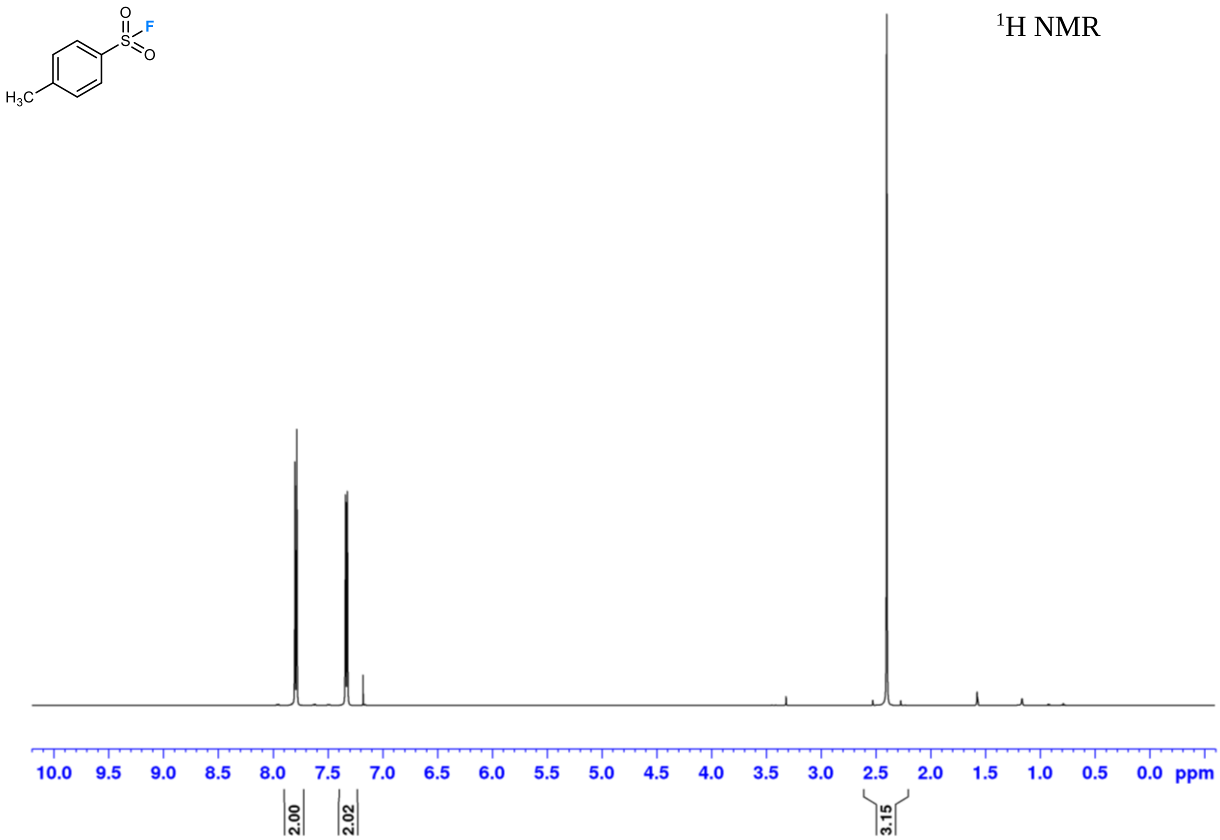

^{13}C NMR

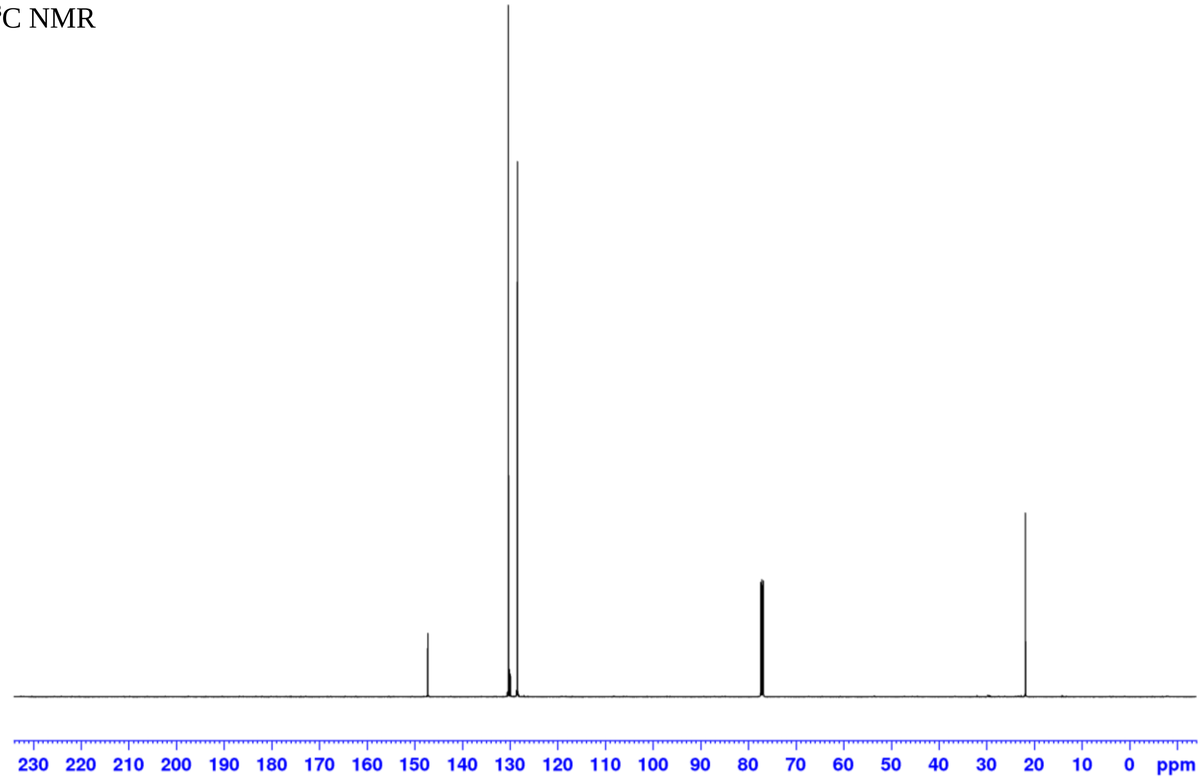

^{19}F NMR

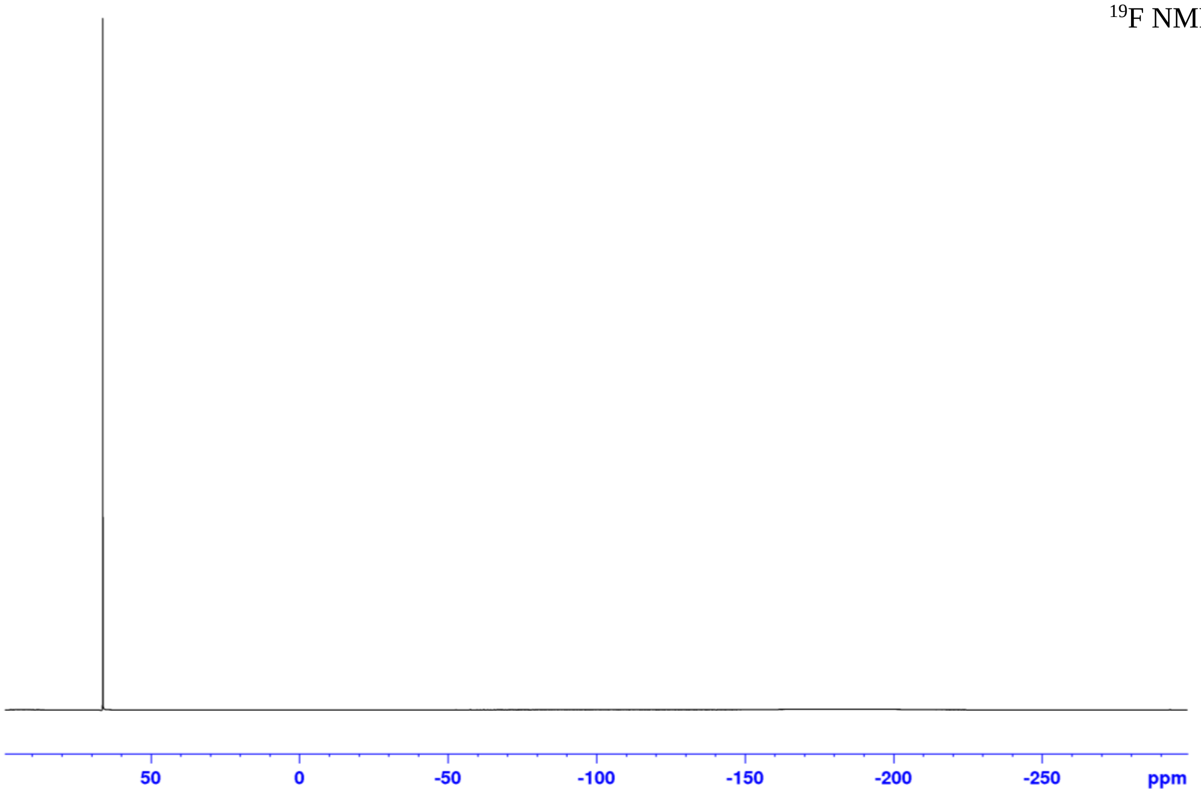

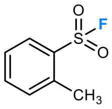

^{1}H NMR

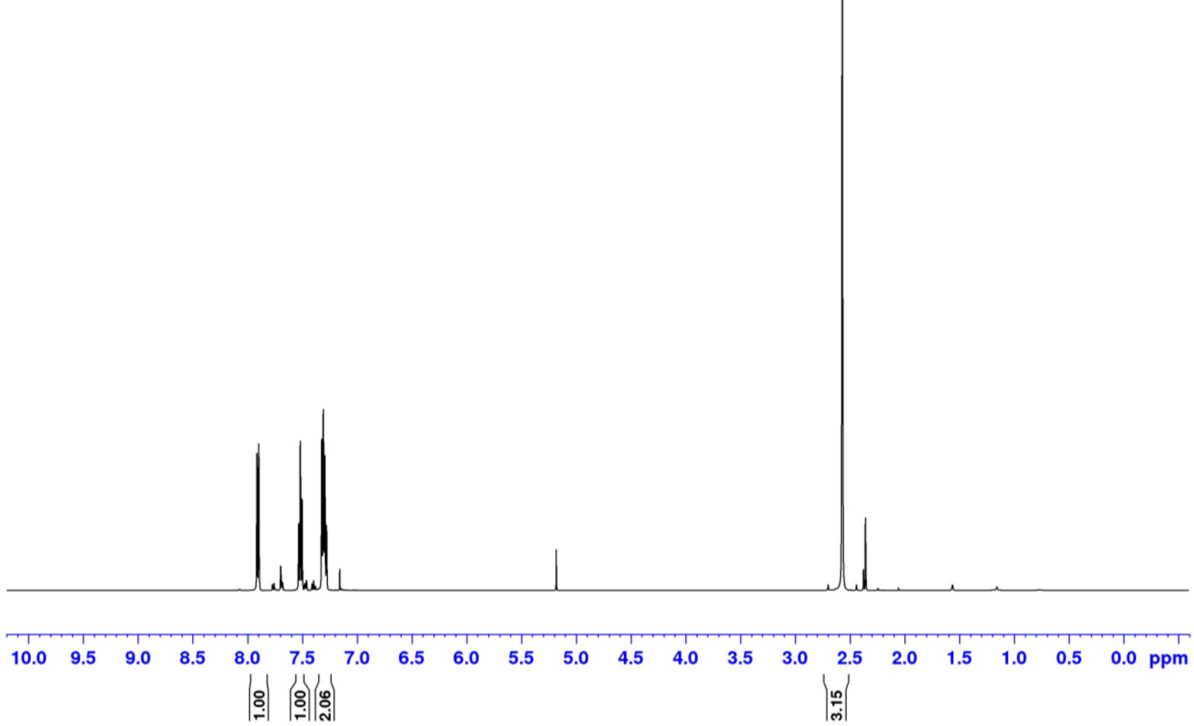

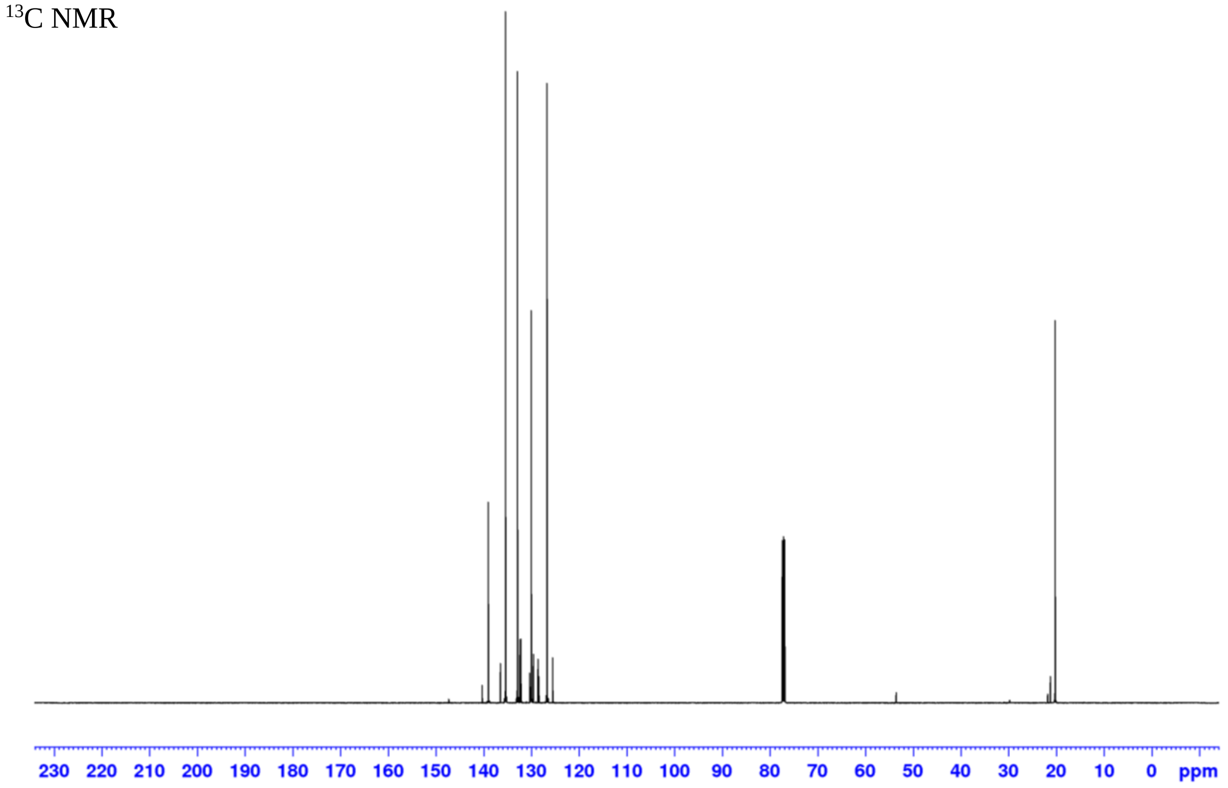
13C NMR
230
220
210
200
190
180
170
160
150
140
130
120
110
100
90
80
70
60
50
40
30
20
10
0
ppm

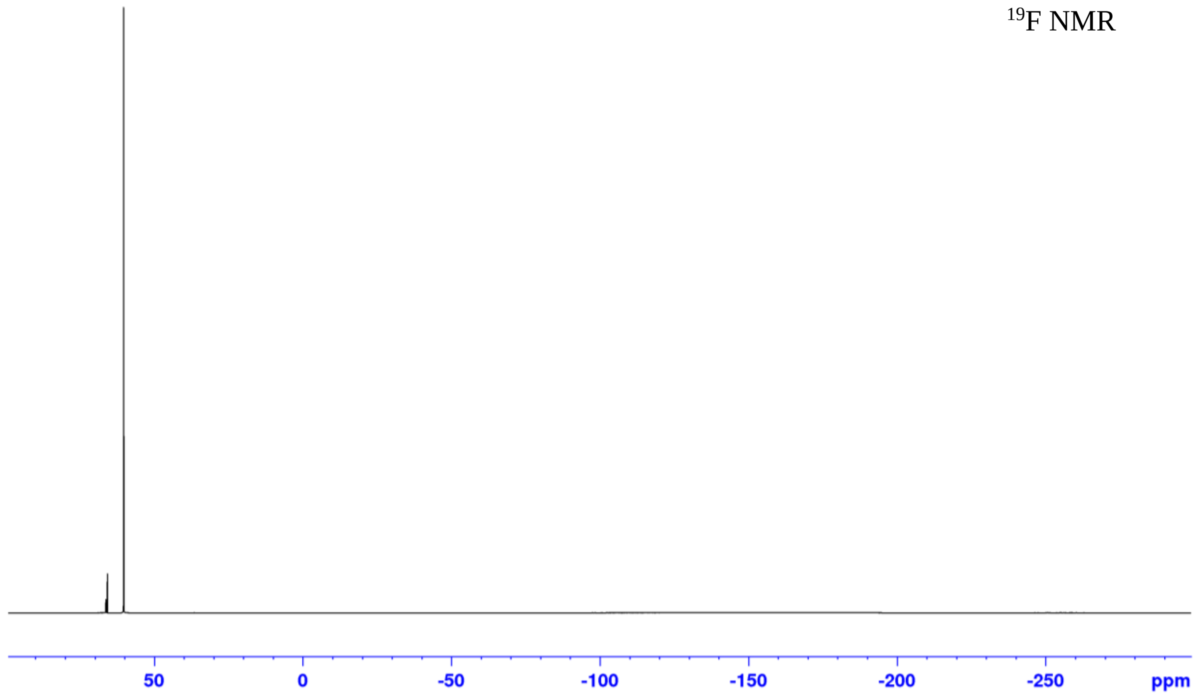
19F NMR
50
0
-50
-100
-150
-200
-250
ppm

Commercial ortho-methyl benzene sulfonyl chloride starting material

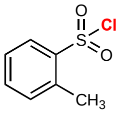

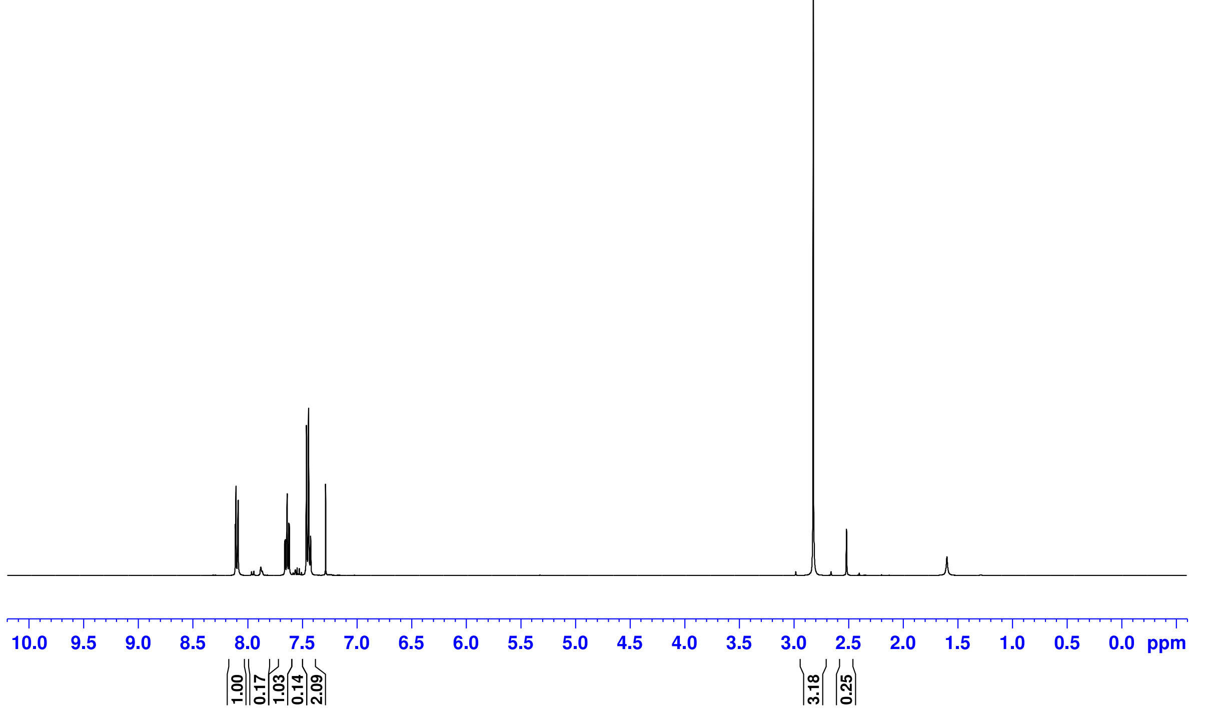

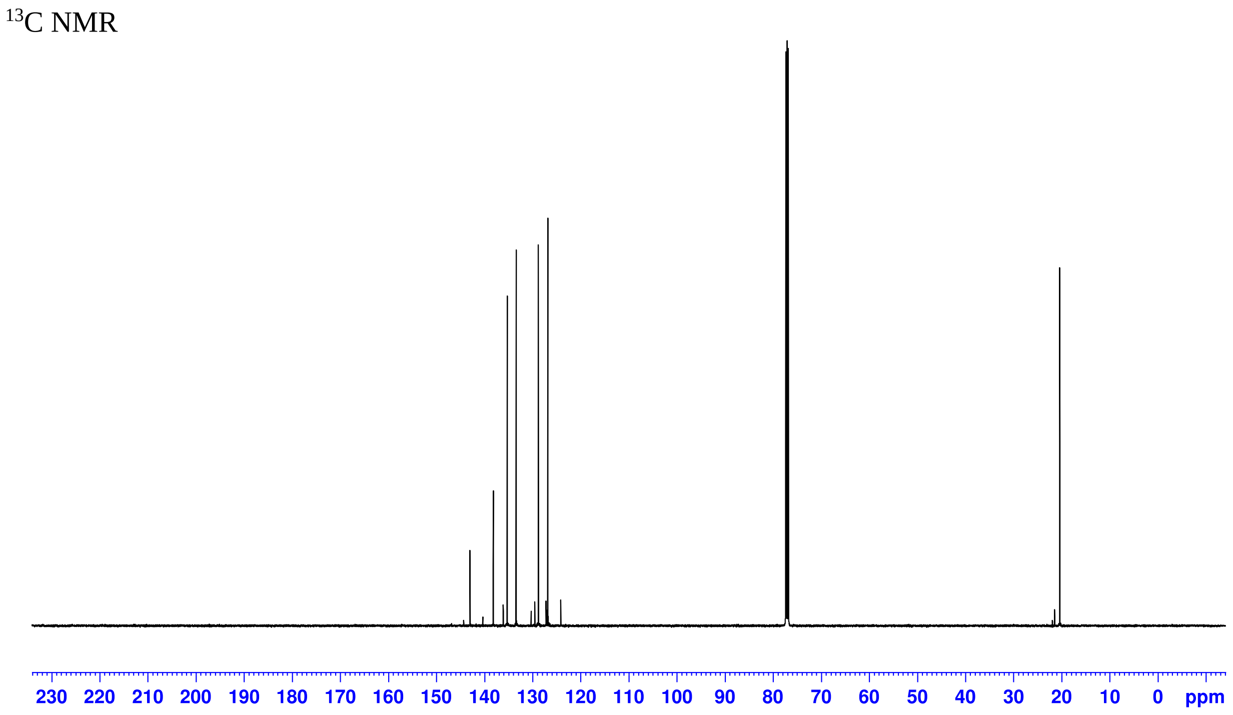

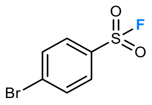

^{1}H NMR

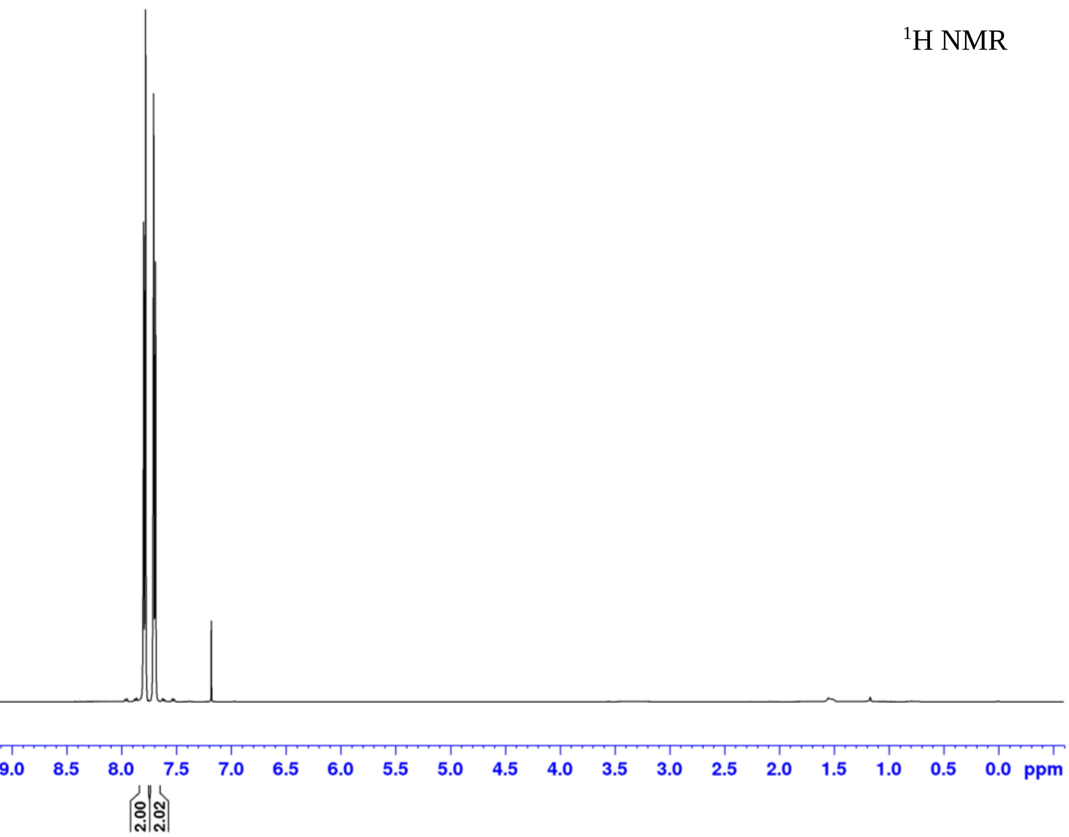

^{13}C NMR

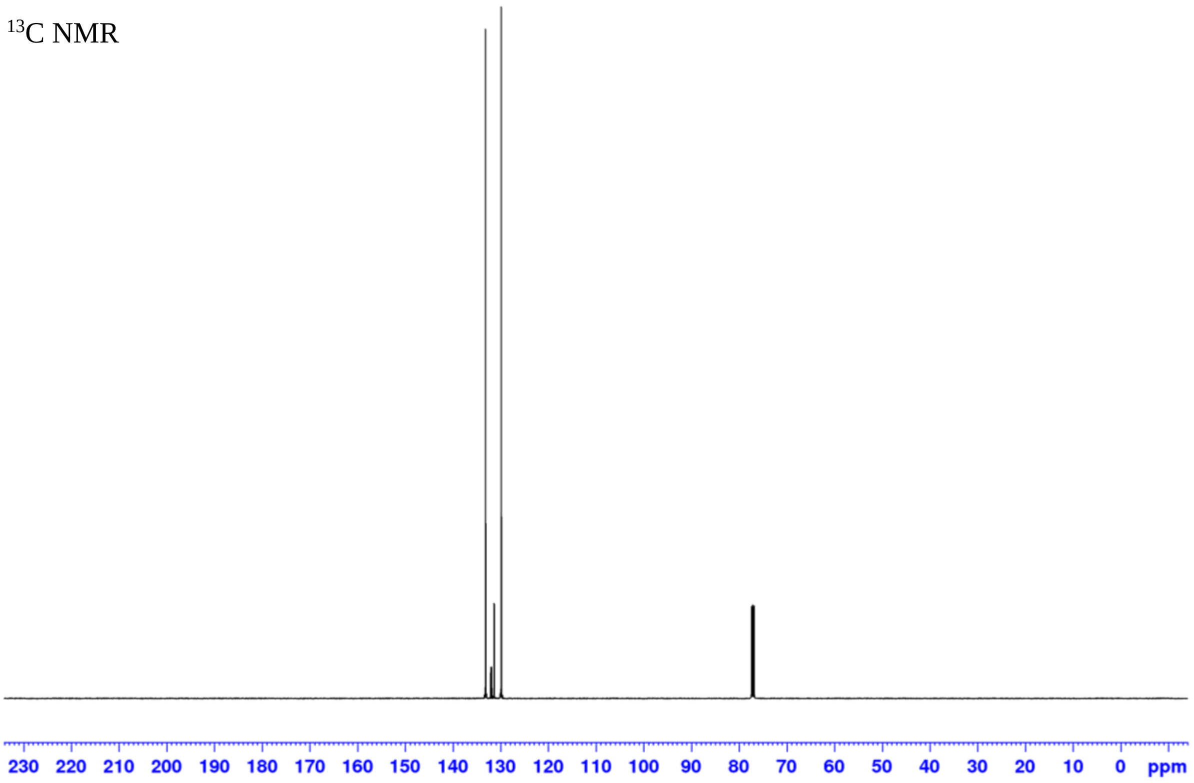

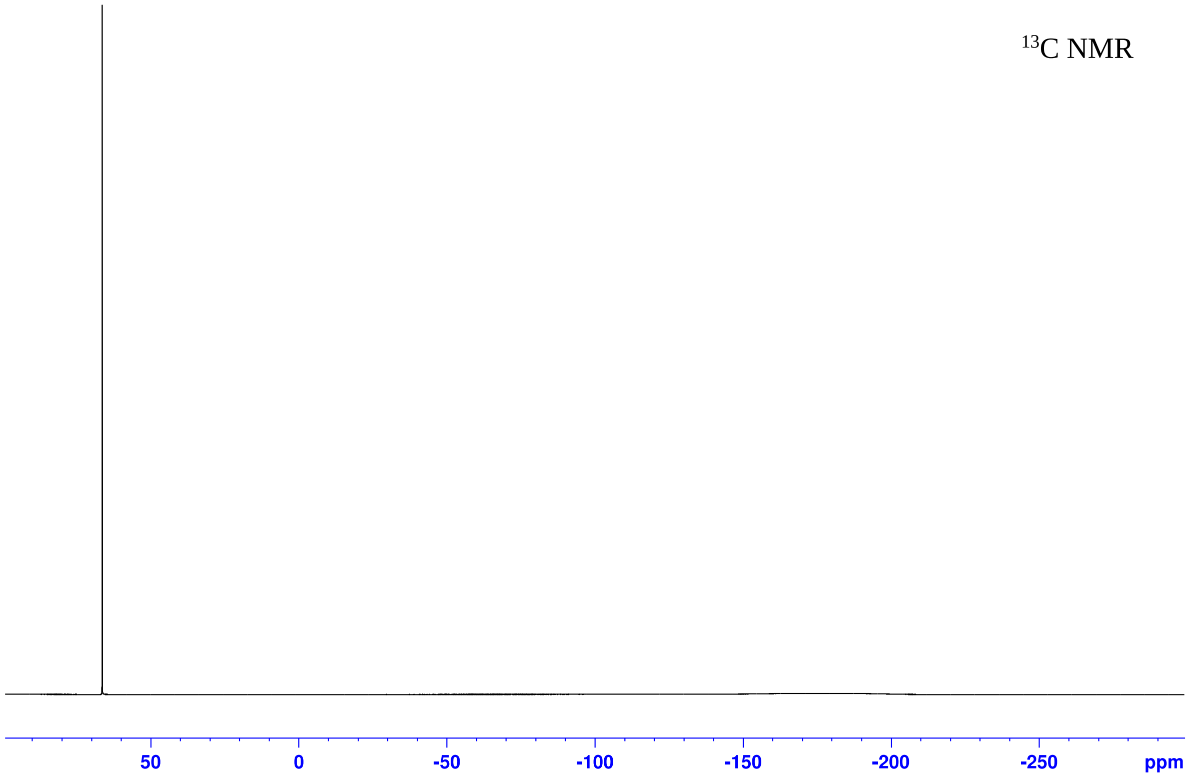

^{13}C NMR
50
0
-50
-100
-150
-200
-250
ppm

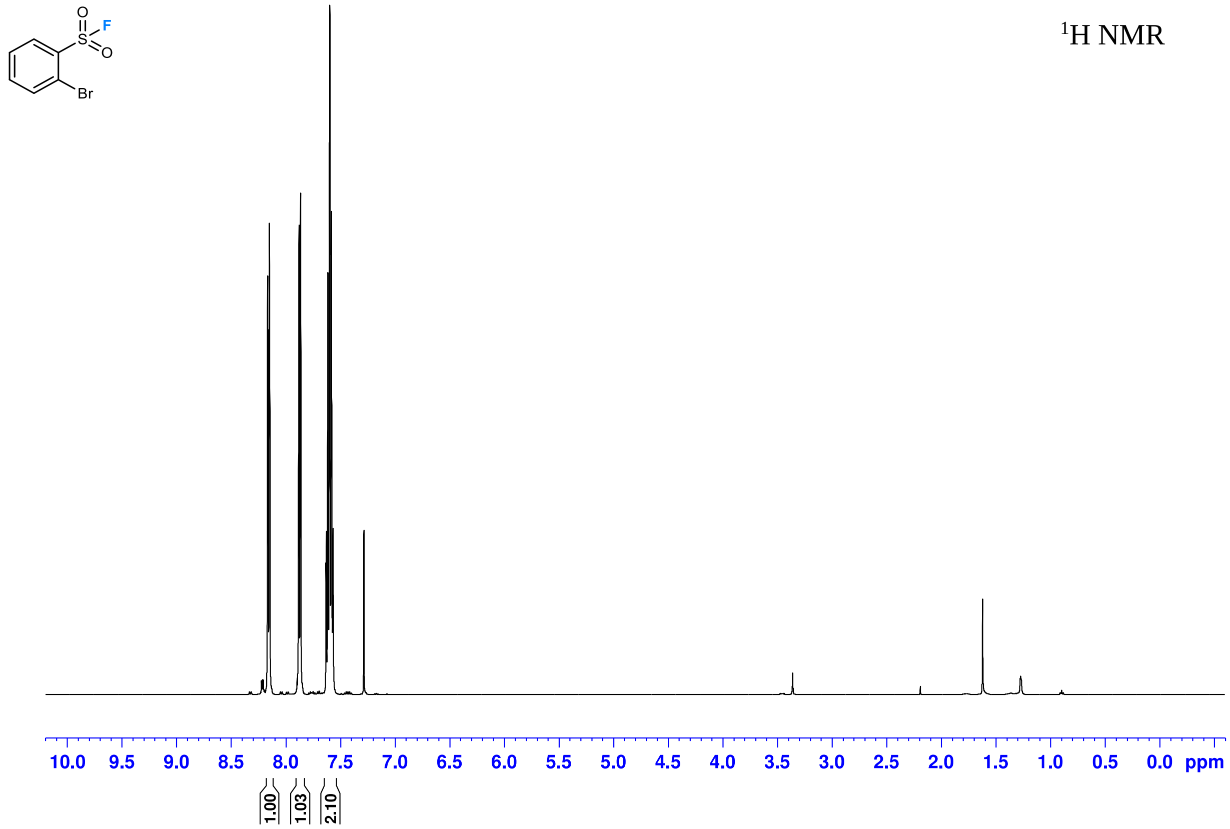

O
S
F
O
Br
^{1}H NMR
10.0
9.5
9.0
8.5
8.0
7.5
7.0
6.5
6.0
5.5
5.0
4.5
4.0
3.5
3.0
2.5
2.0
1.5
1.0
0.5
0.0
ppm
1.00
1.03
2.10

^{13}C NMR

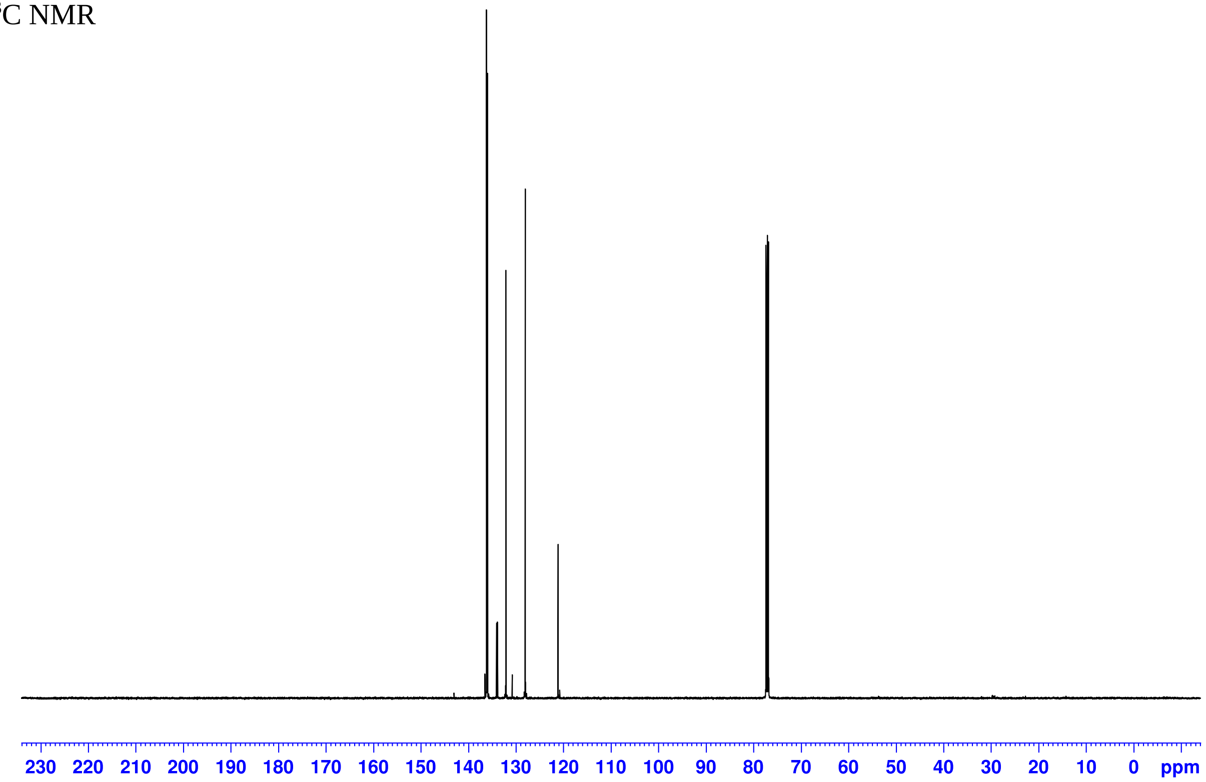

^{19}F NMR

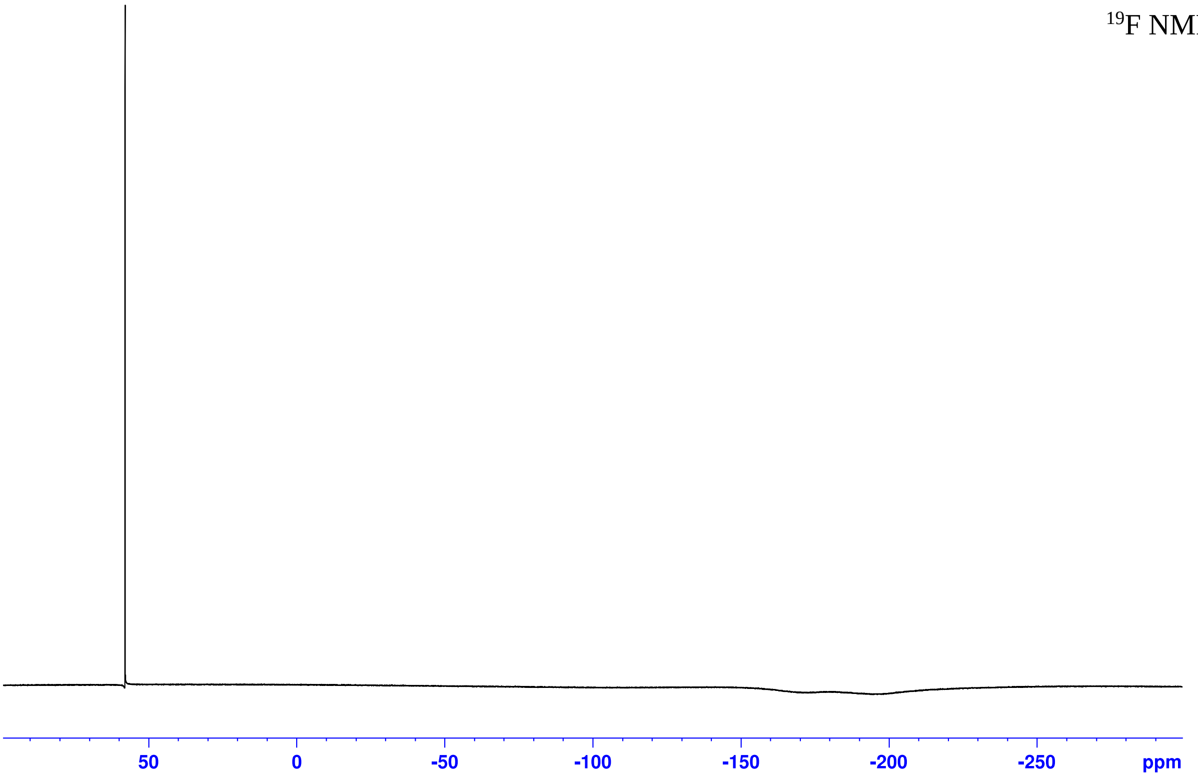

^{1}H NMR

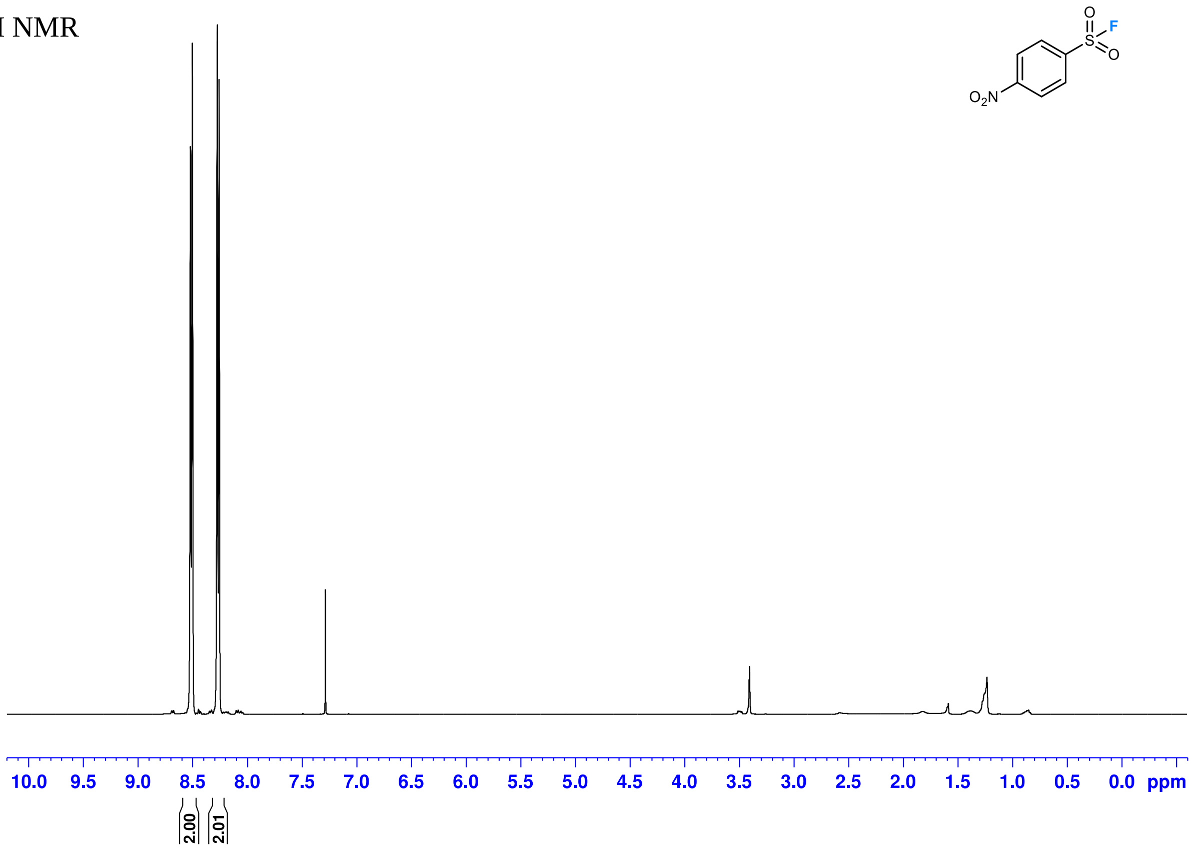

^{13}C NMR

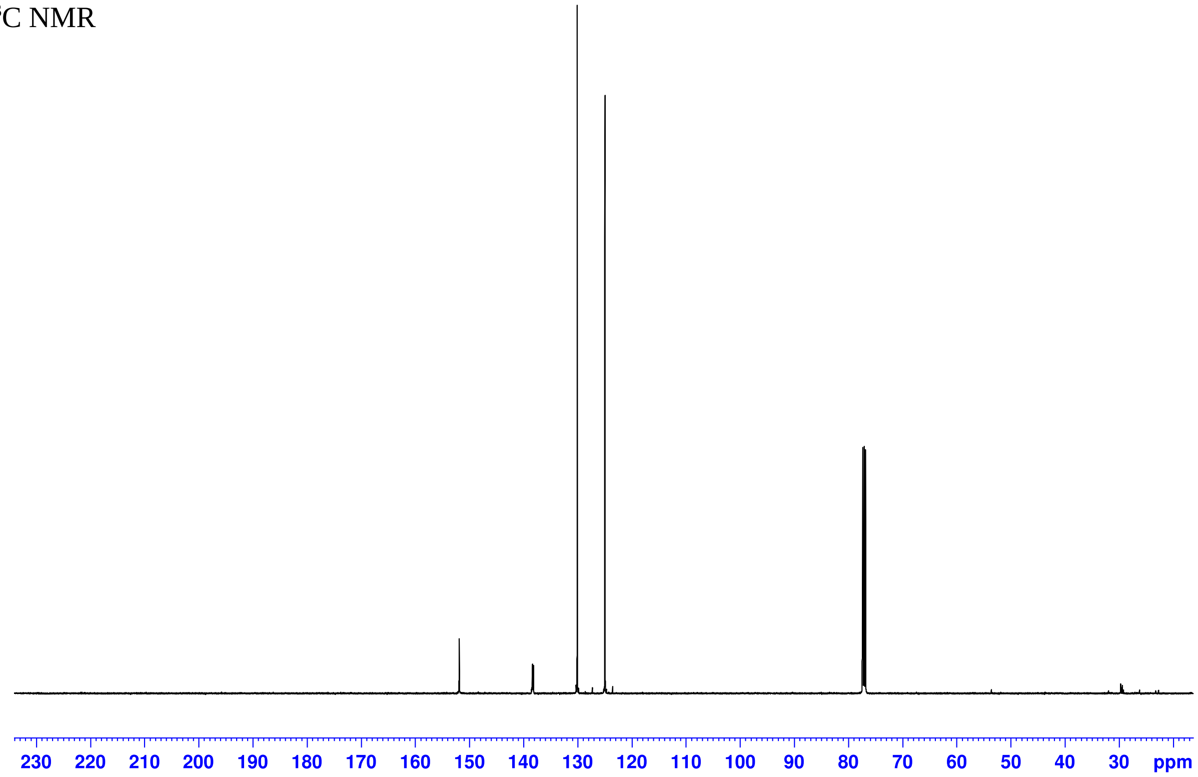

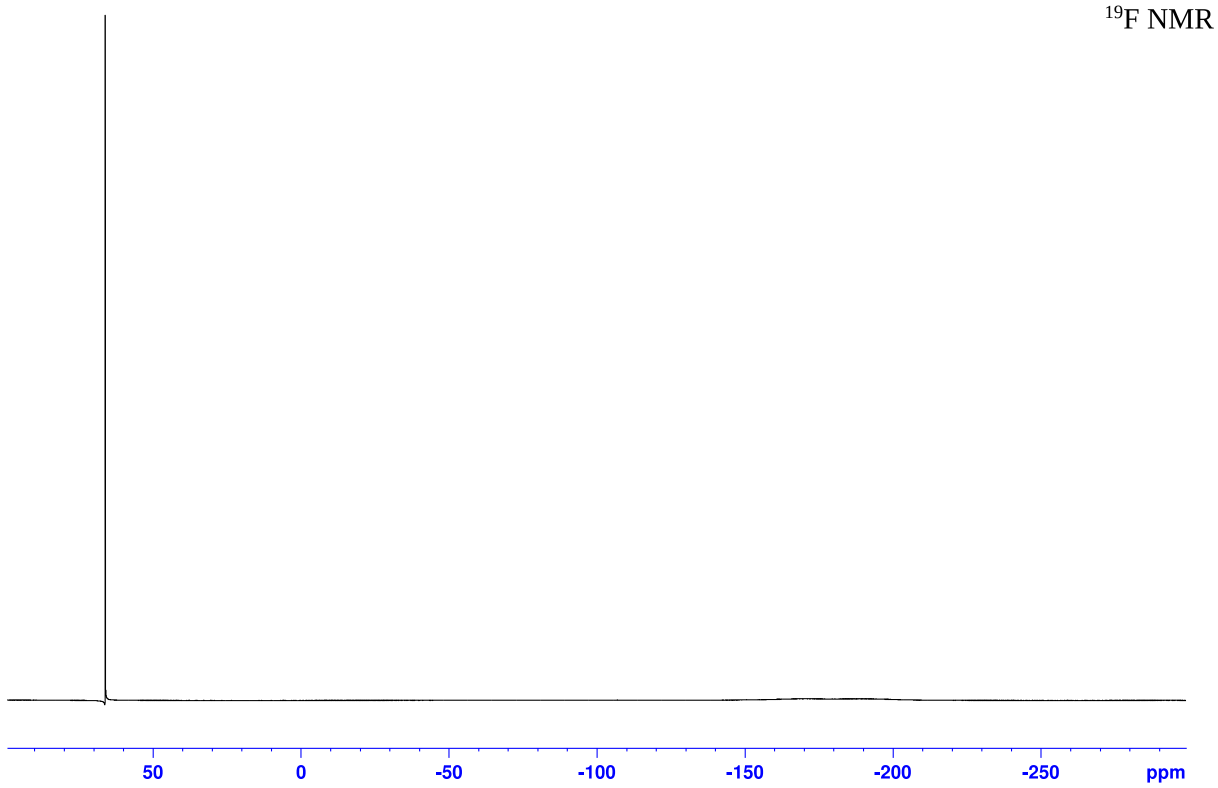
^{19}F NMR
50
0
-50
-100
-150
-200
-250
ppm

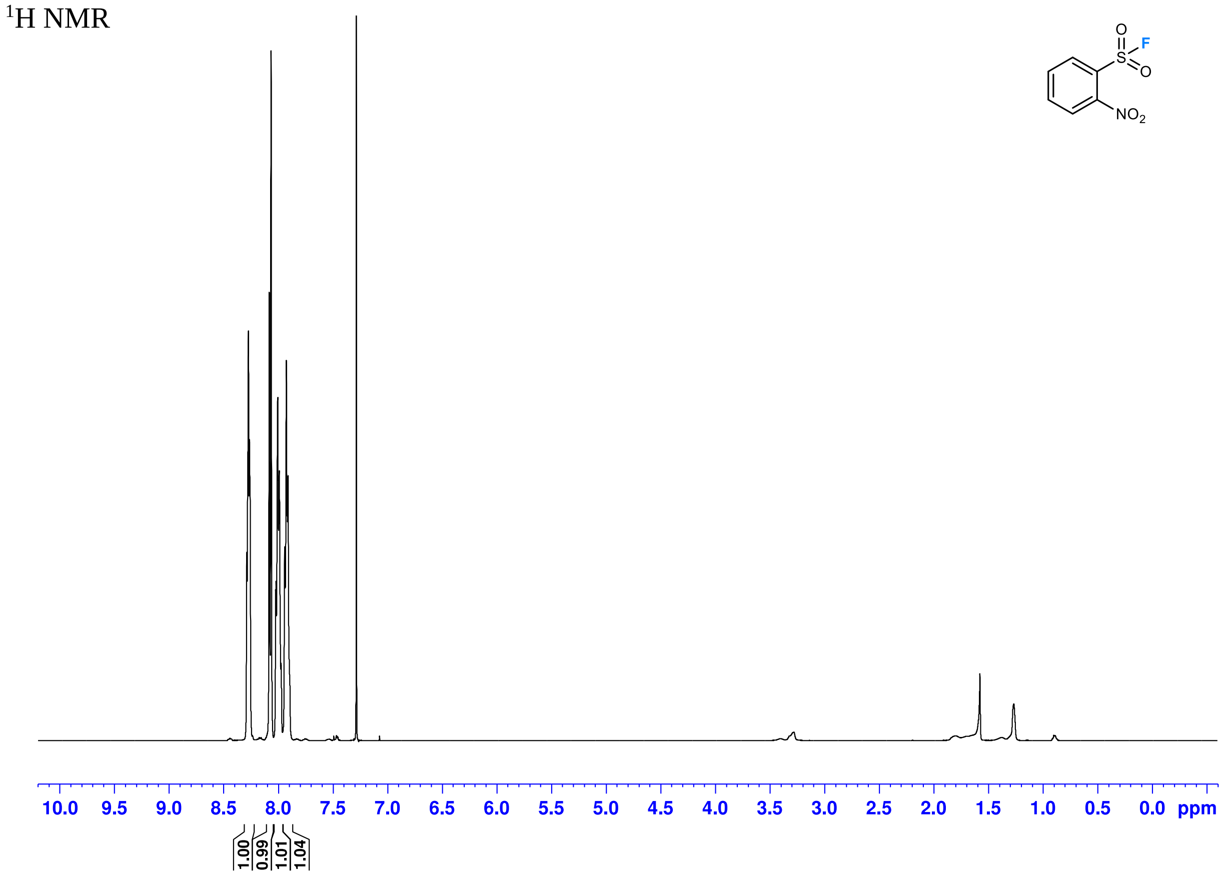
^{1}H NMR
O
S
F
O
NO$_2$
10.0 9.5 9.0 8.5 8.0 7.5 7.0 6.5 6.0 5.5 5.0 4.5 4.0 3.5 3.0 2.5 2.0 1.5 1.0 0.5 0.0 ppm
1.00
0.99
1.01
1.04

^{13}C NMR

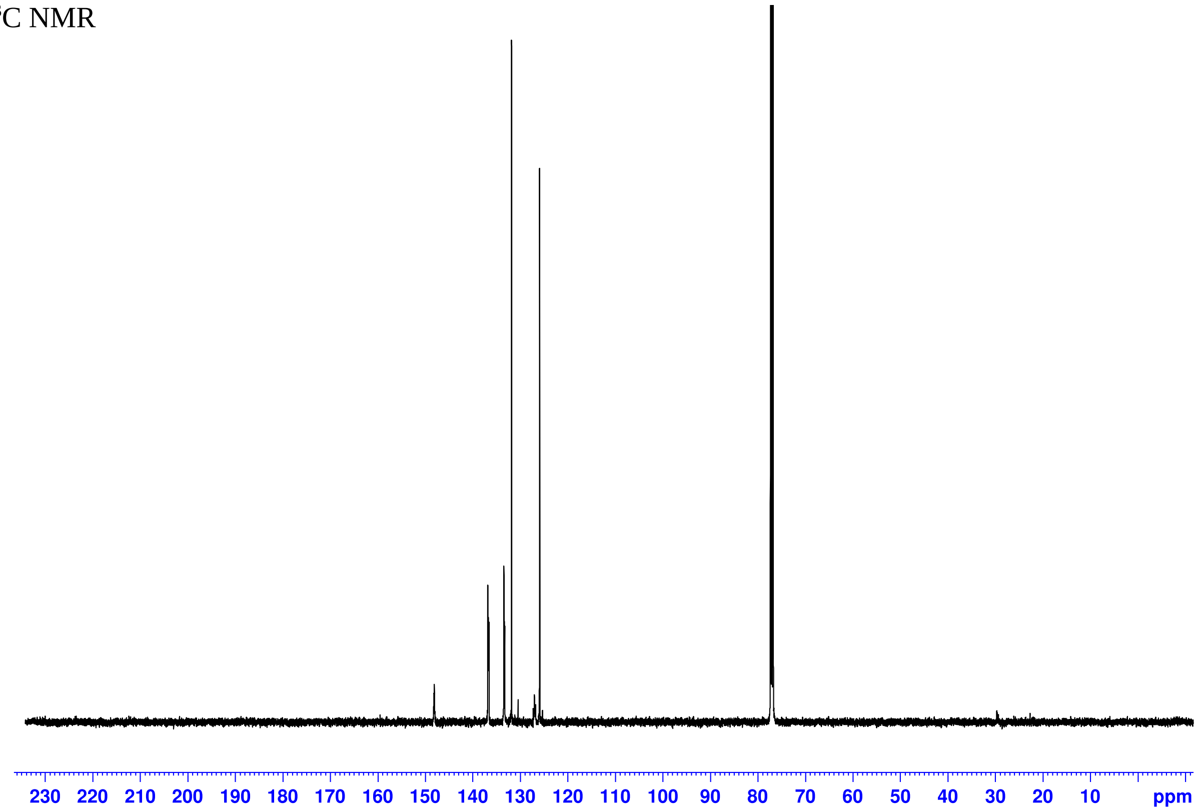

^{19}F NMR

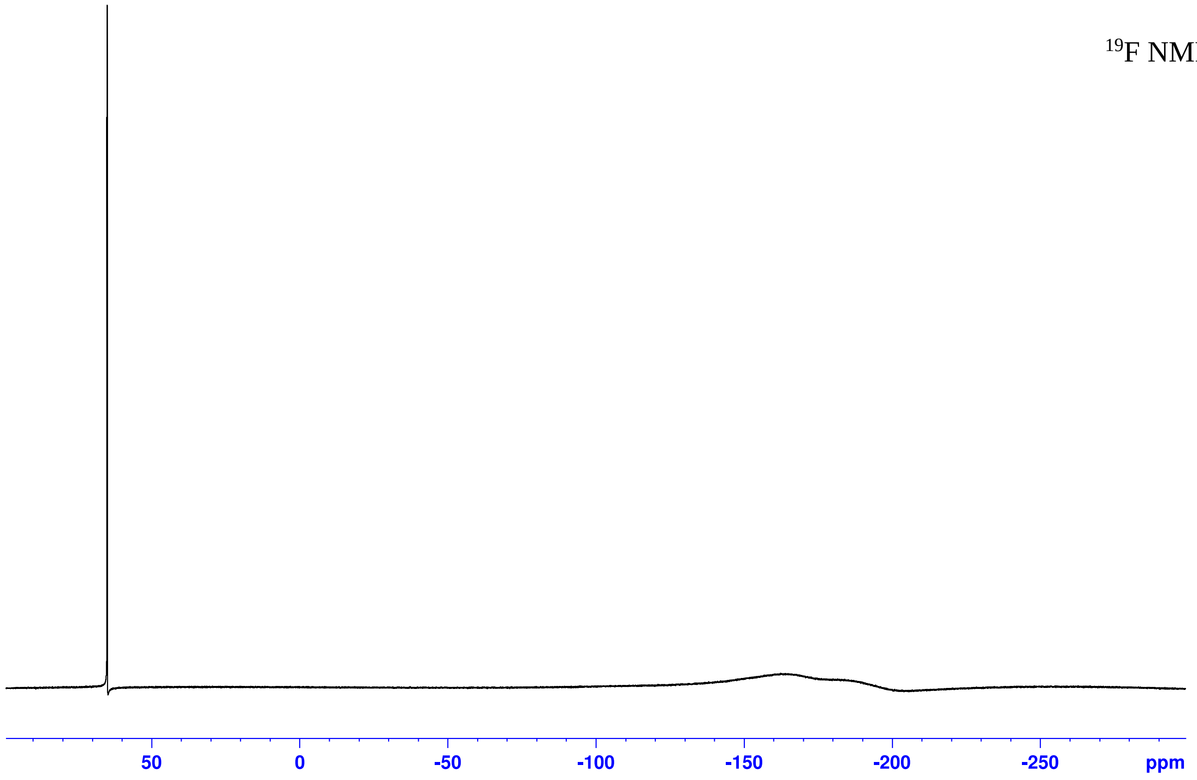

^{1}H NMR

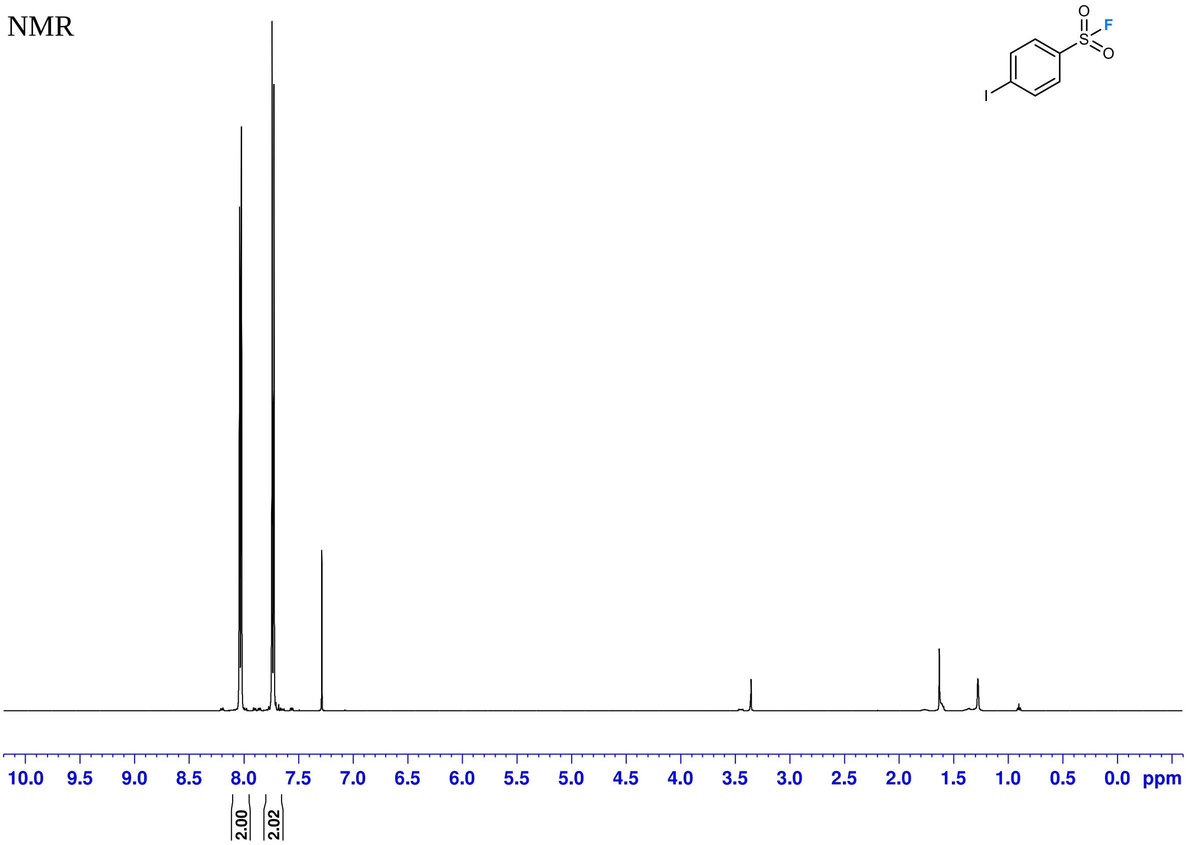

^{13}C NMR

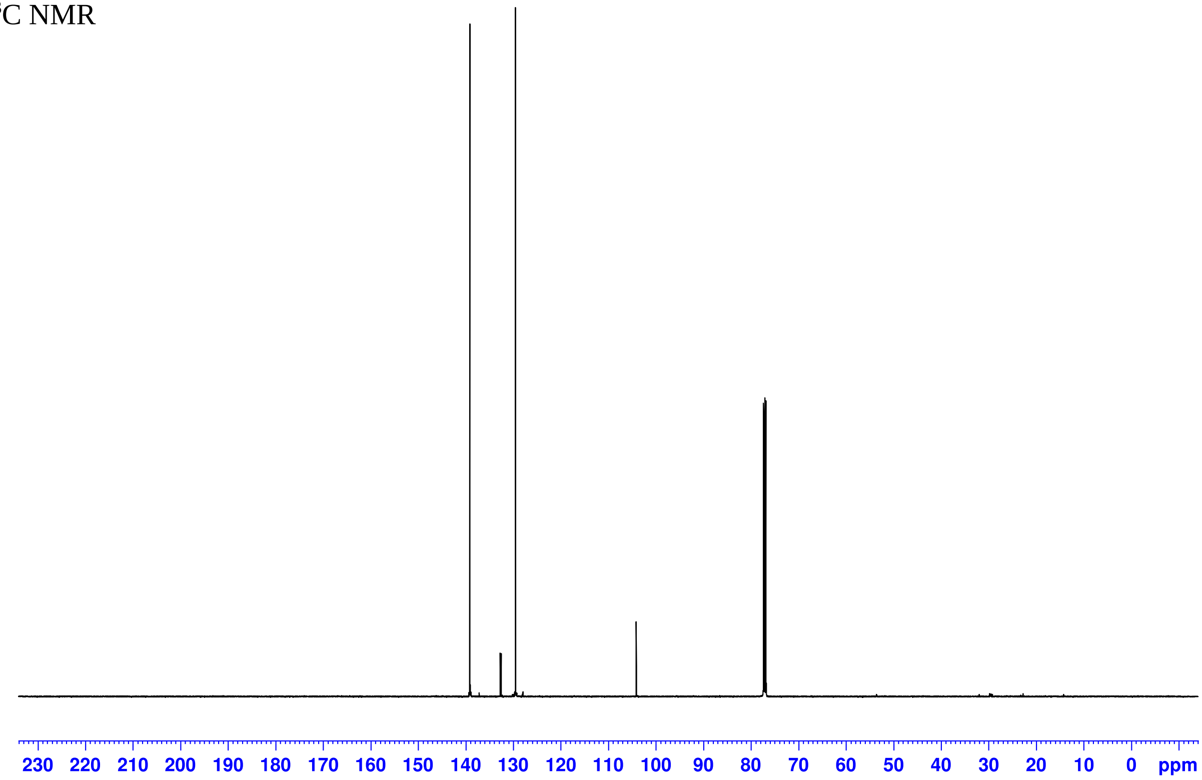

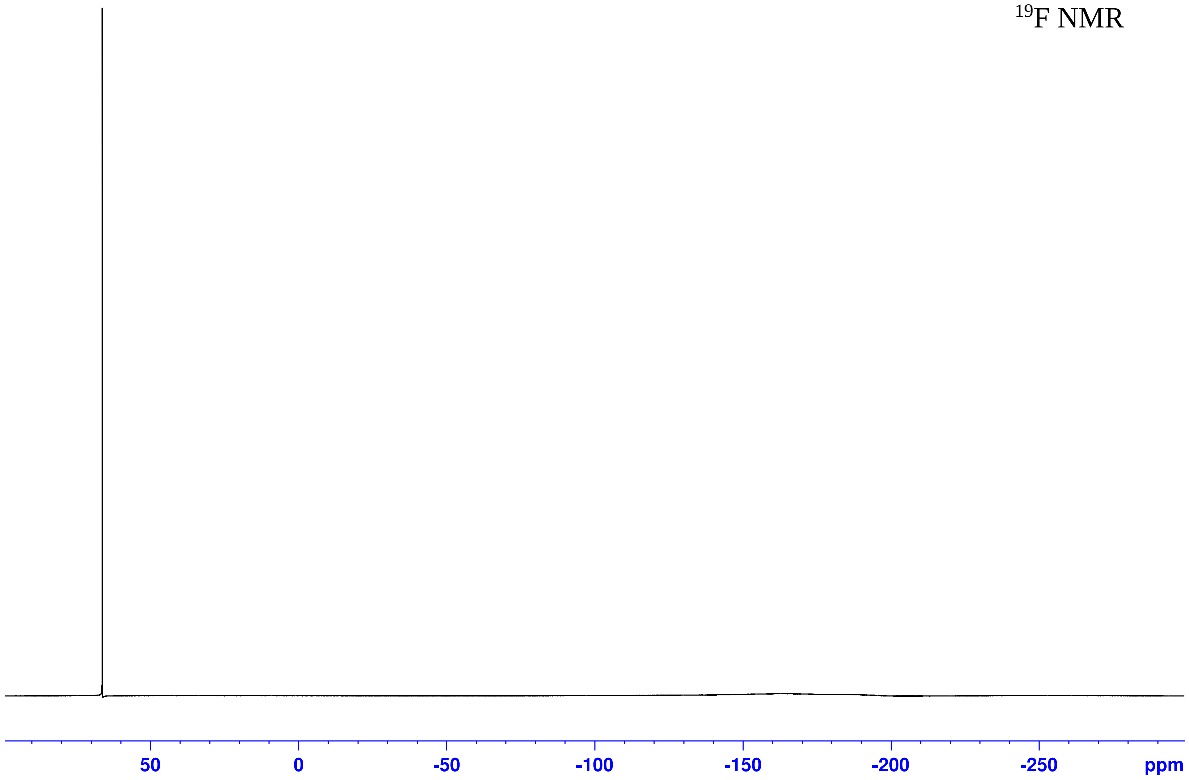
19F NMR
50
0
-50
-100
-150
-200
-250
ppm

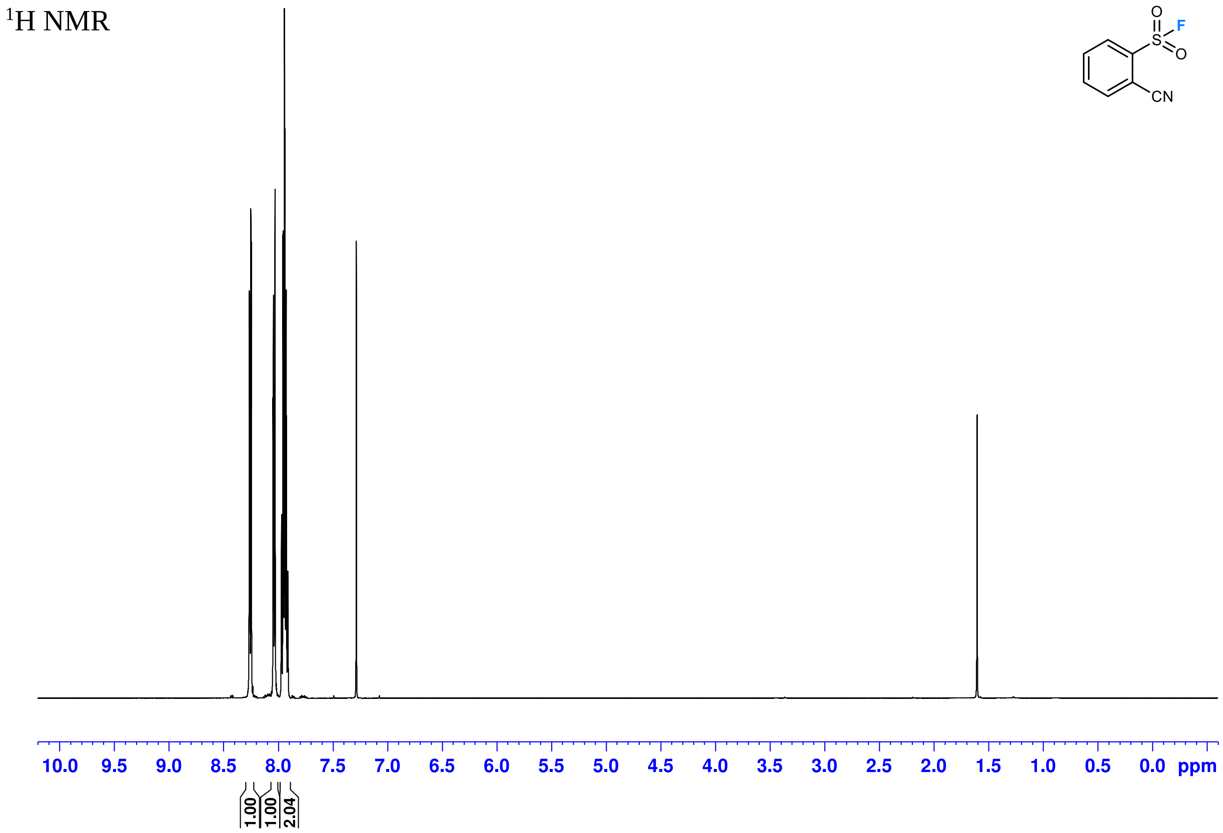
1H NMR
O
S
F
O
CN
10.0
9.5
9.0
8.5
8.0
7.5
7.0
6.5
6.0
5.5
5.0
4.5
4.0
3.5
3.0
2.5
2.0
1.5
1.0
0.5
0.0
ppm
1.00
1.00
2.04

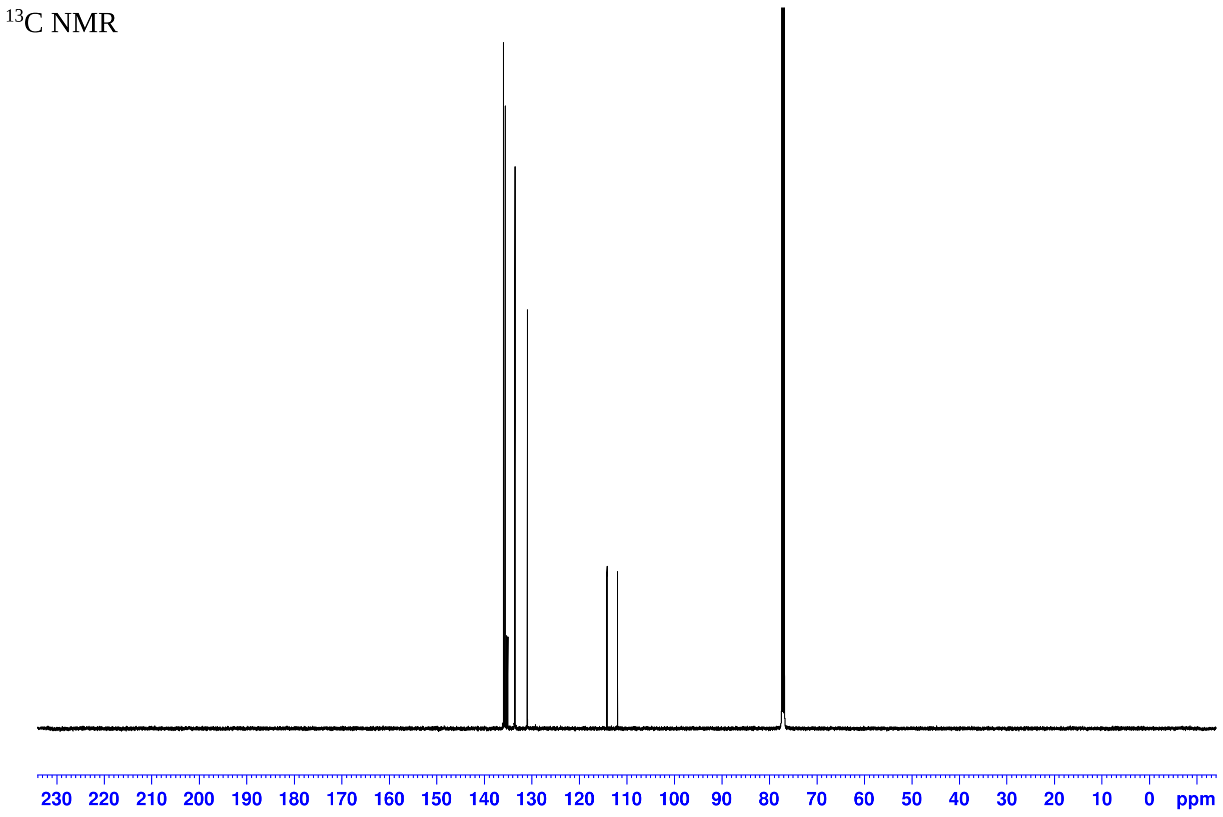
13C NMR
230 220 210 200 190 180 170 160 150 140 130 120 110 100 90 80 70 60 50 40 30 20 10 0 ppm

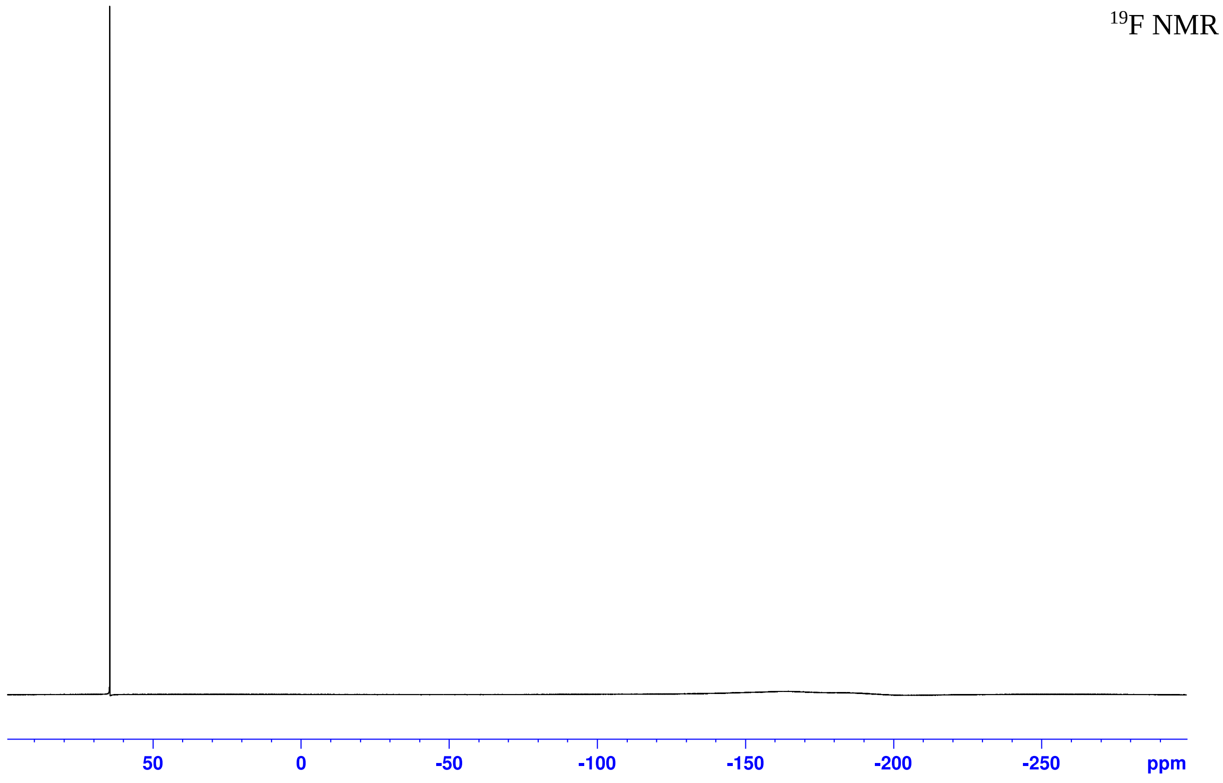
19F NMR
50 0 -50 -100 -150 -200 -250 ppm

^{1}H NMR

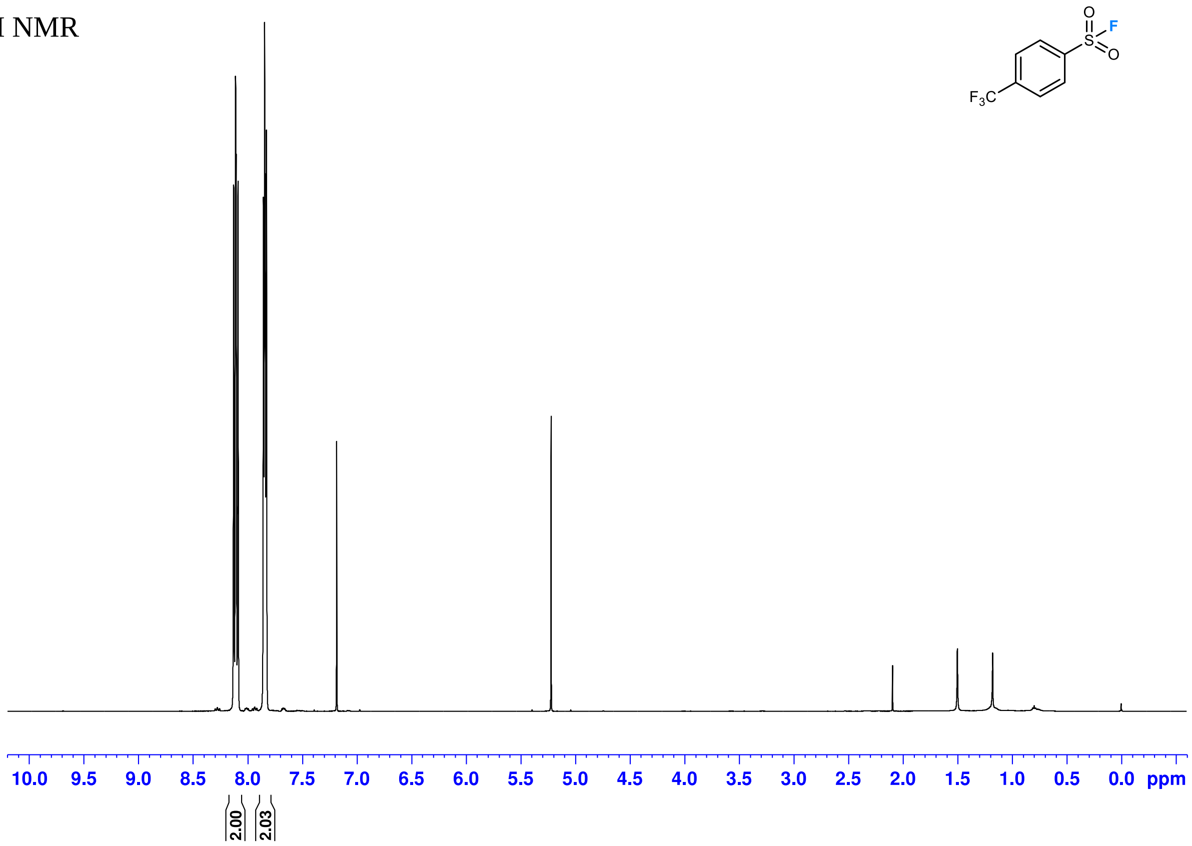

^{13}C NMR

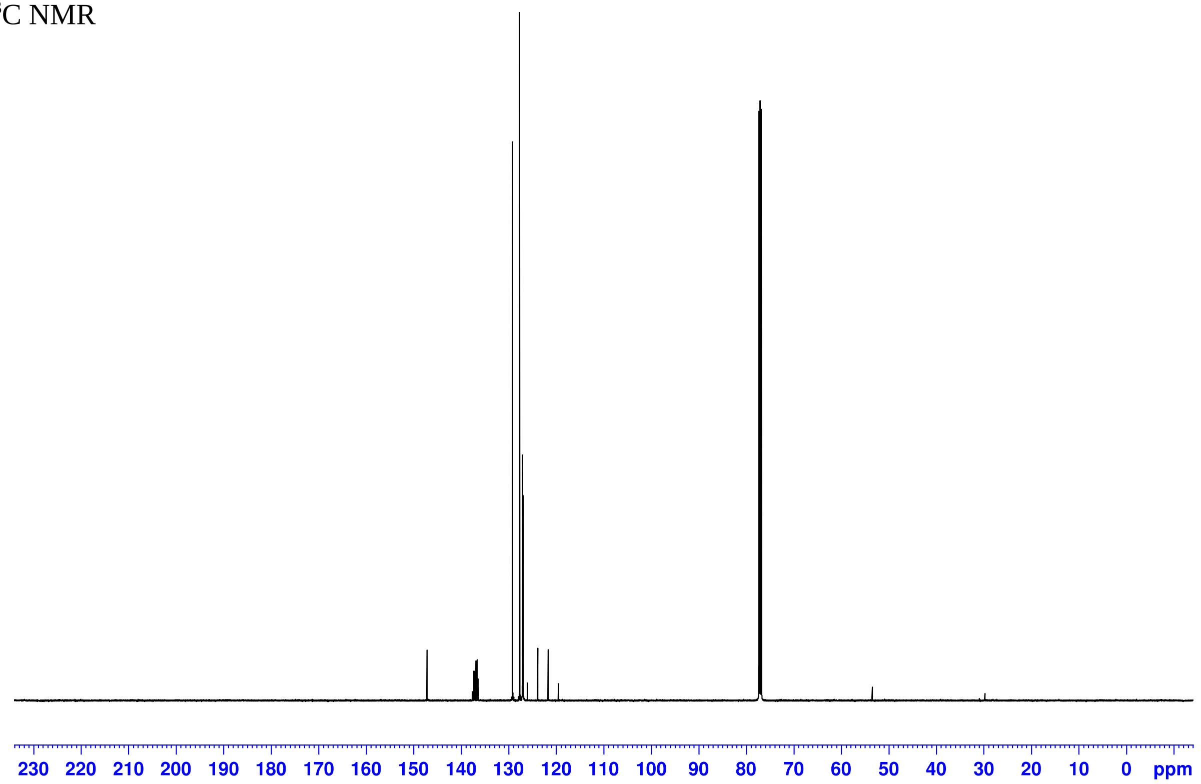

^{19}F NMR

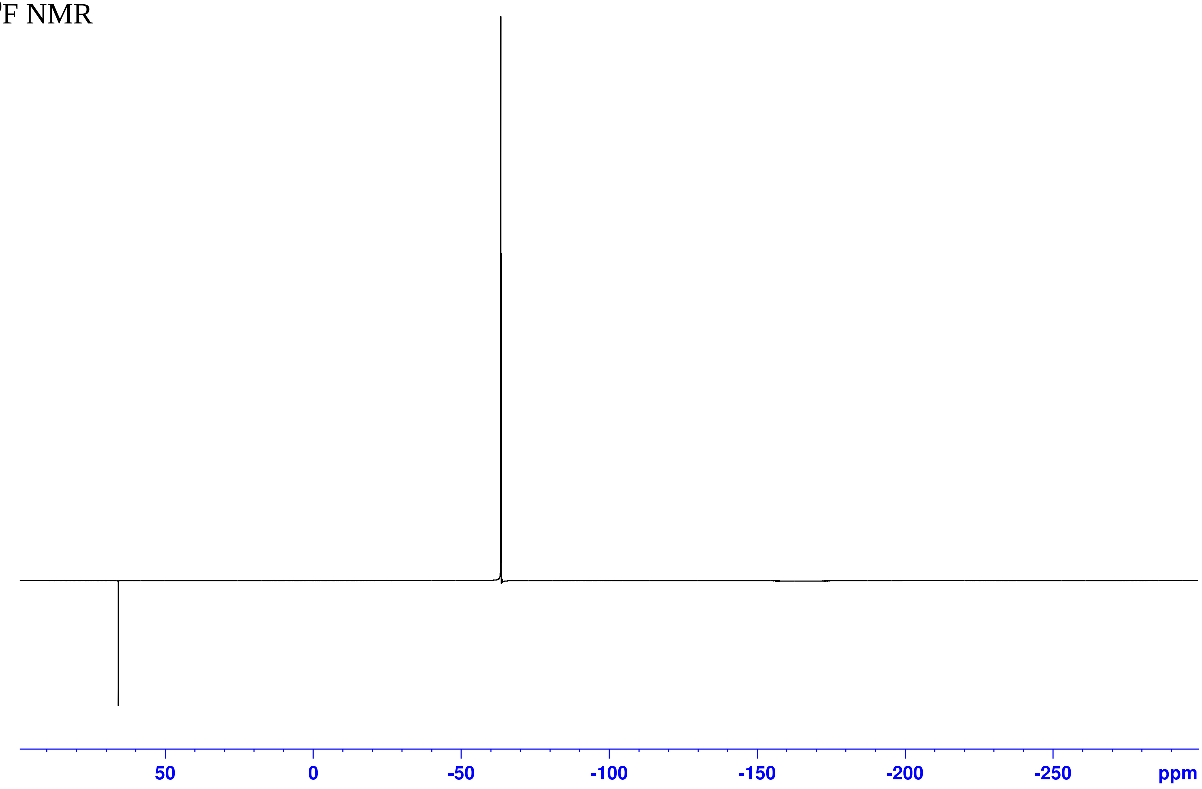

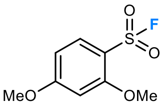

^{1}H NMR

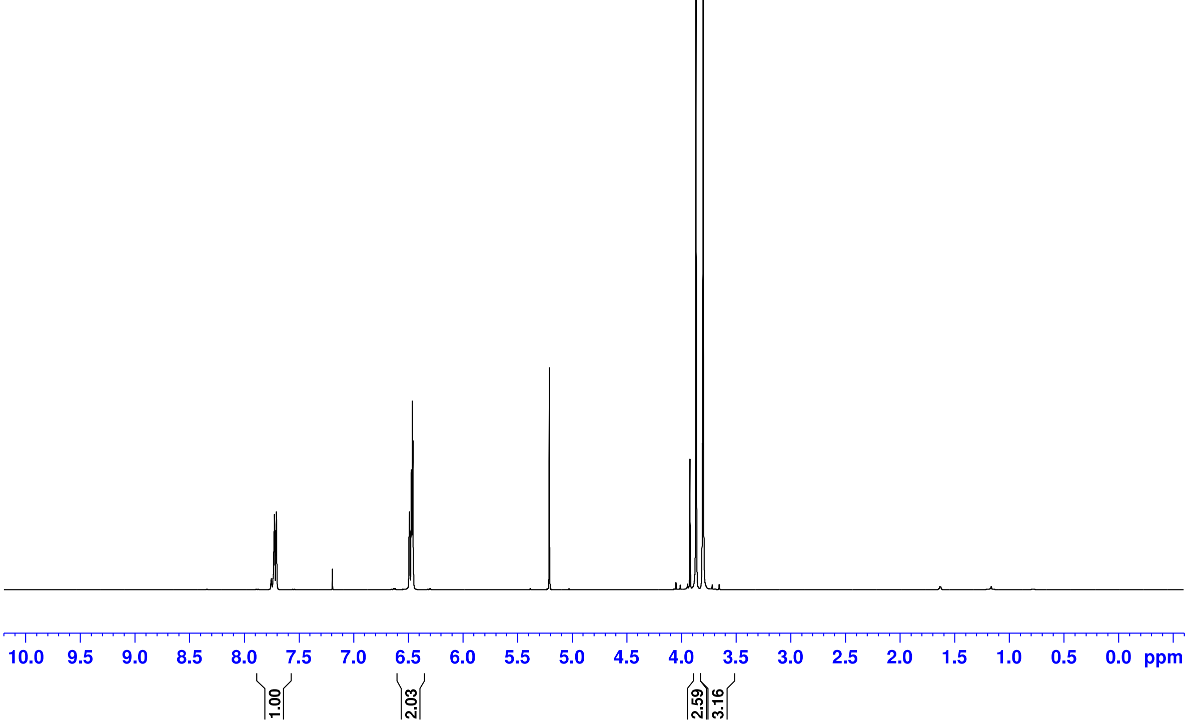

^{13}C NMR

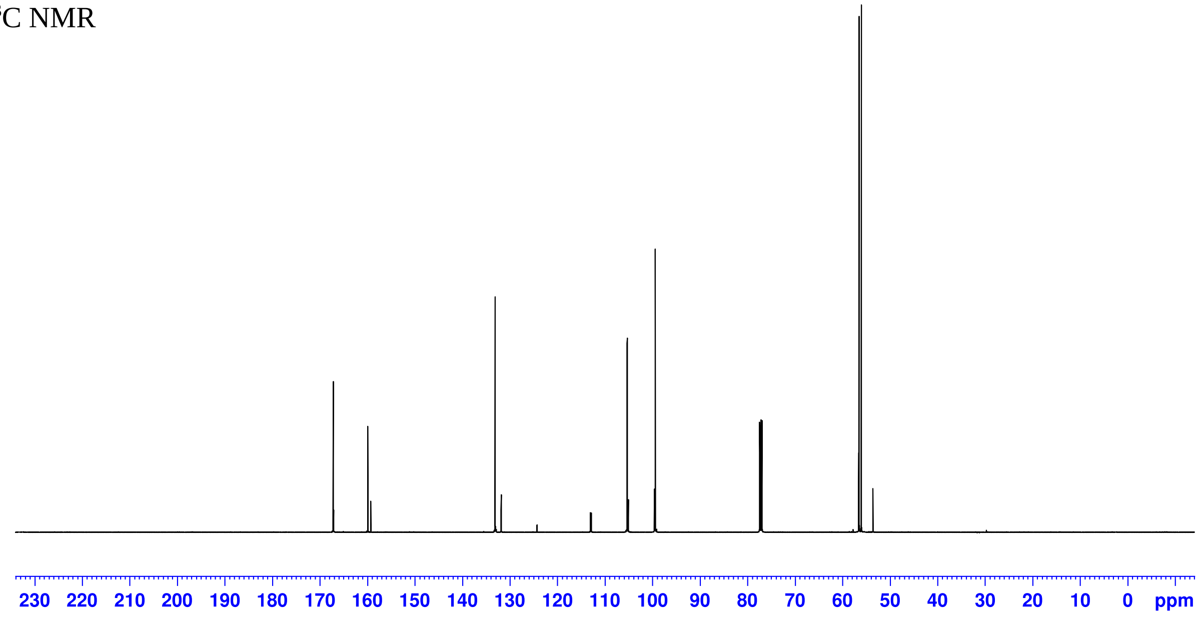

^{19}F NMR

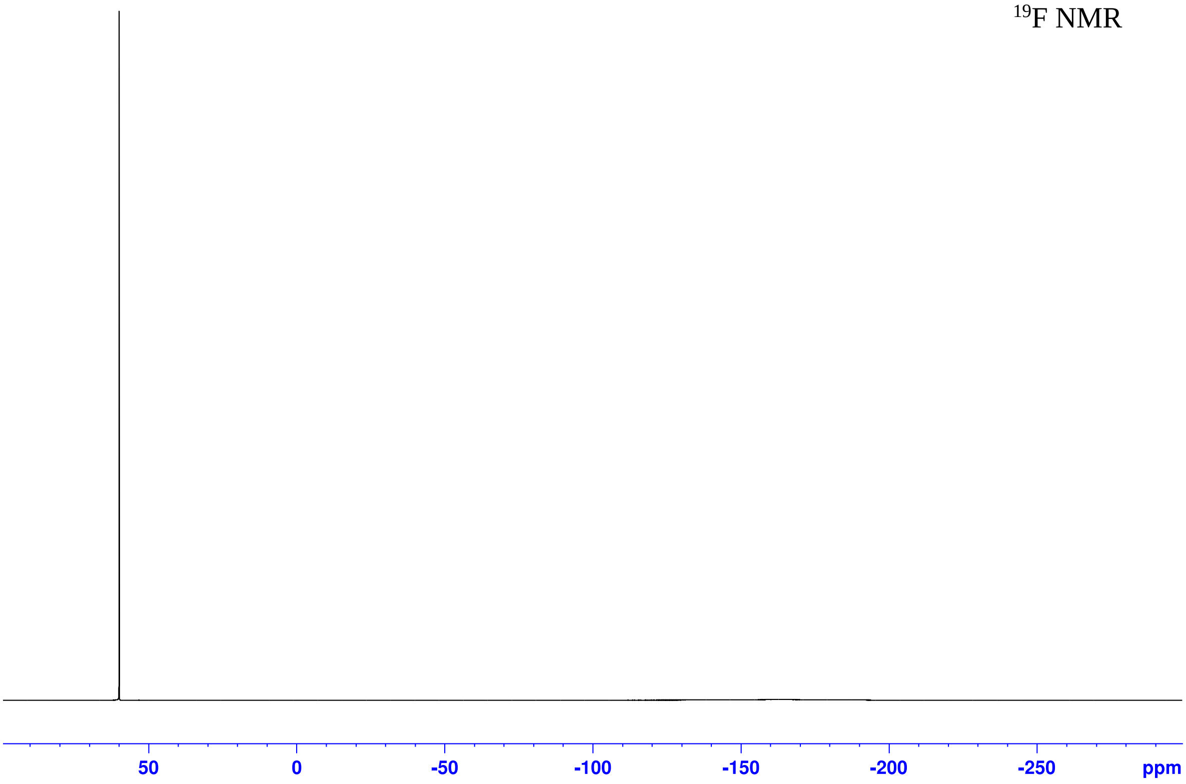

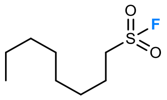

^{1}H NMR

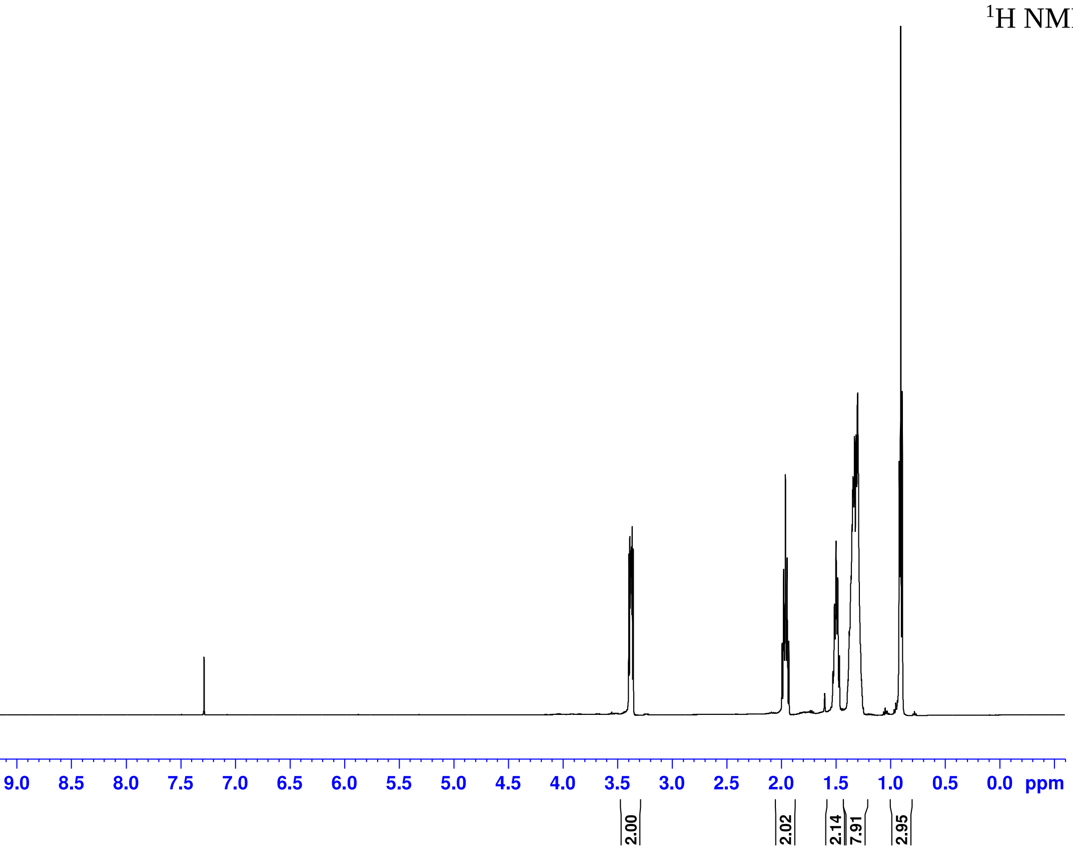

^{13}C NMR

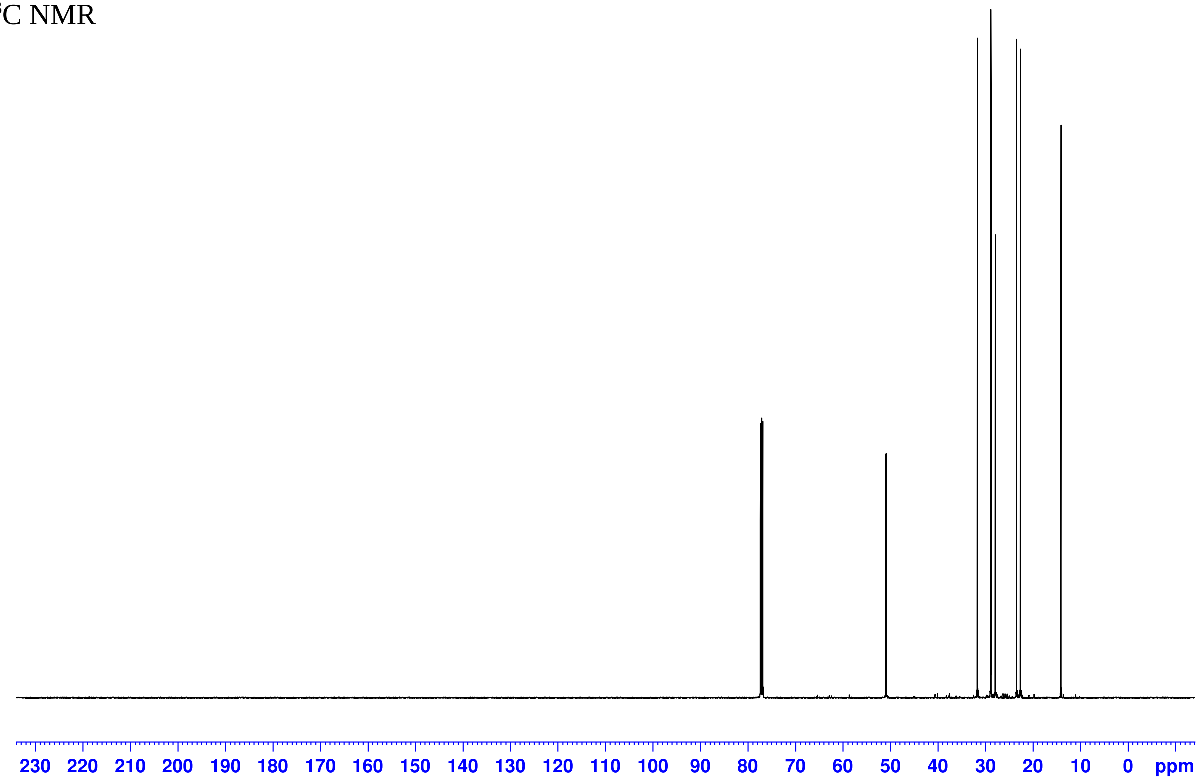

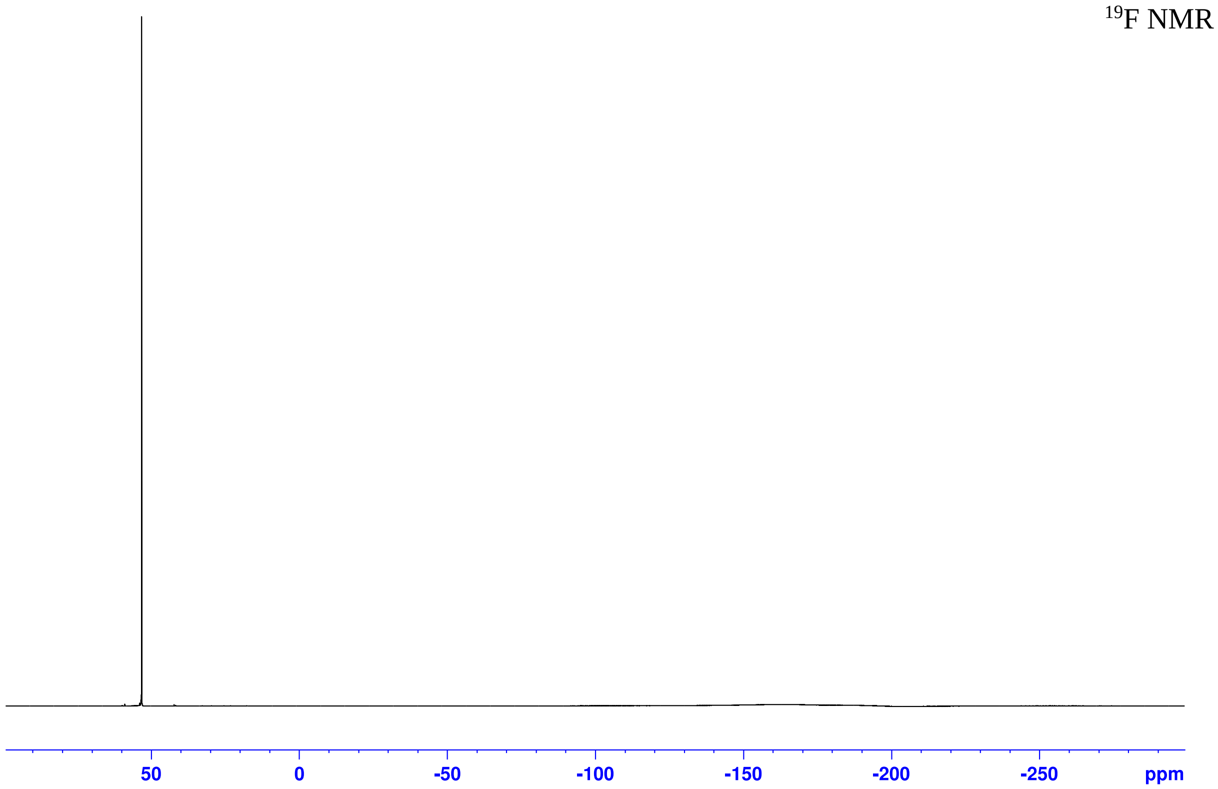

19F NMR
50
0
-50
-100
-150
-200
-250
ppm

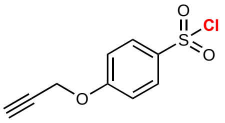

O
S
Cl
O
O

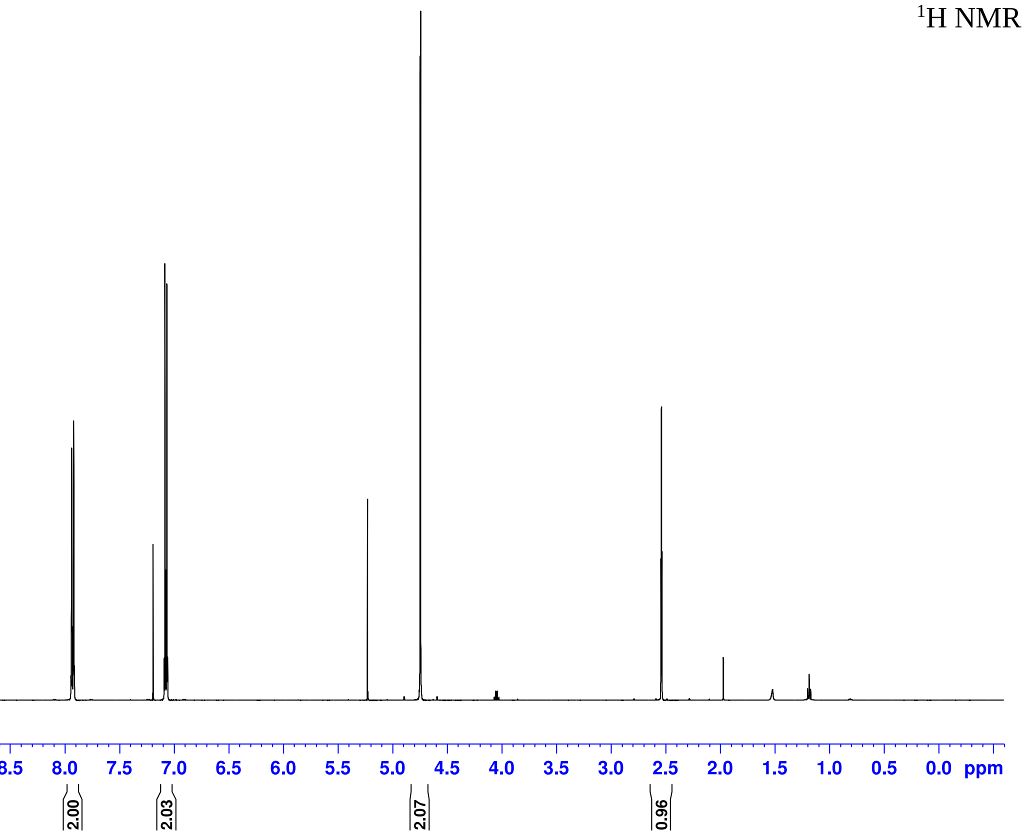

1H NMR
10.0
9.5
9.0
8.5
8.0
7.5
7.0
6.5
6.0
5.5
5.0
4.5
4.0
3.5
3.0
2.5
2.0
1.5
1.0
0.5
0.0
ppm
2.00
2.03
2.07
0.96

^{13}C NMR

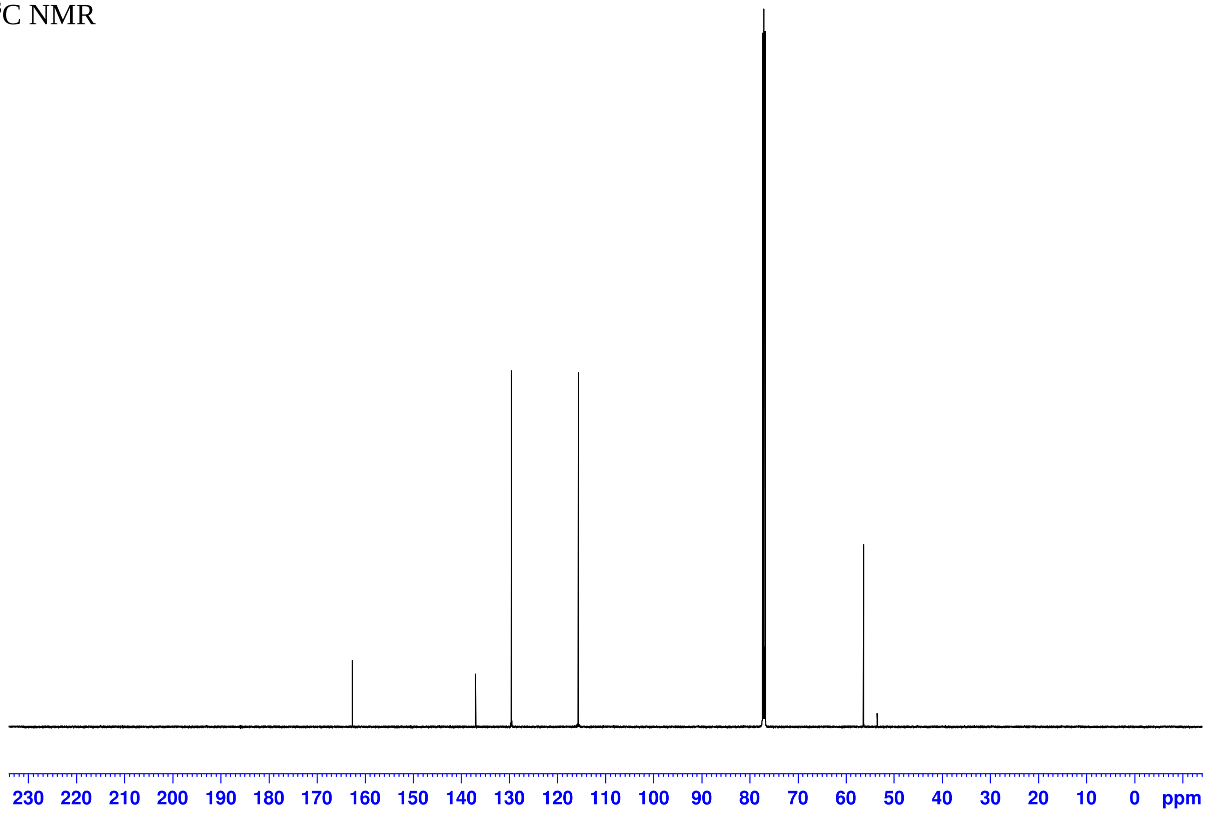

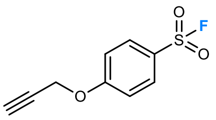

^{1}H NMR

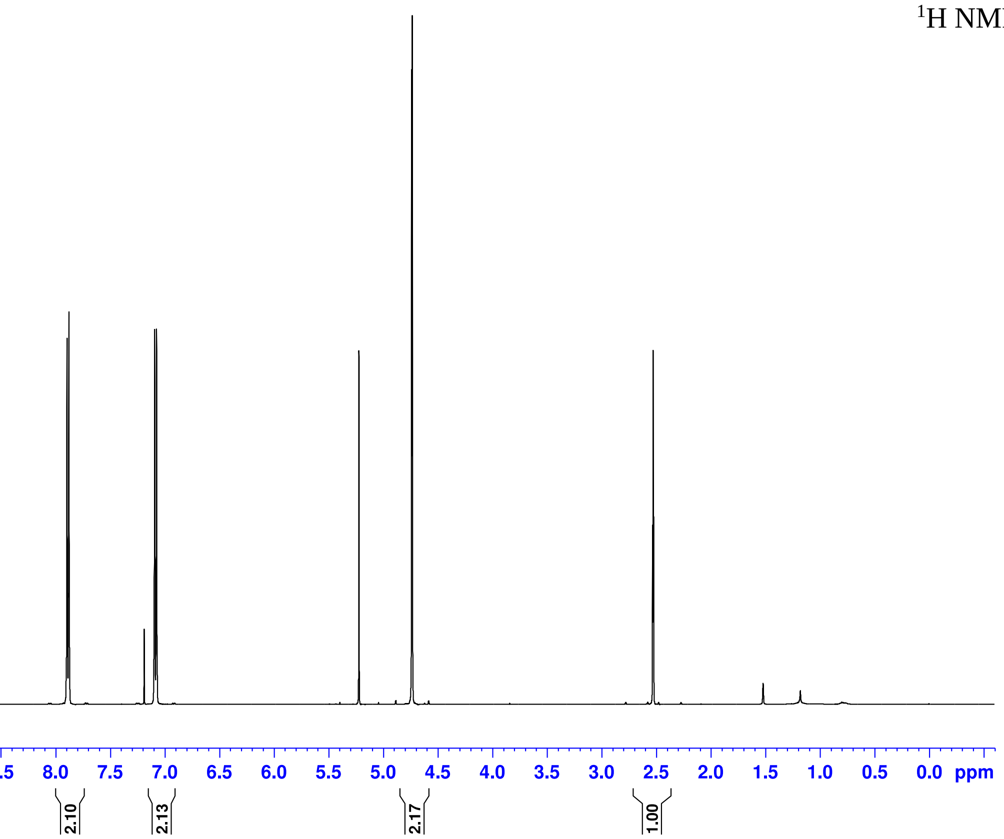

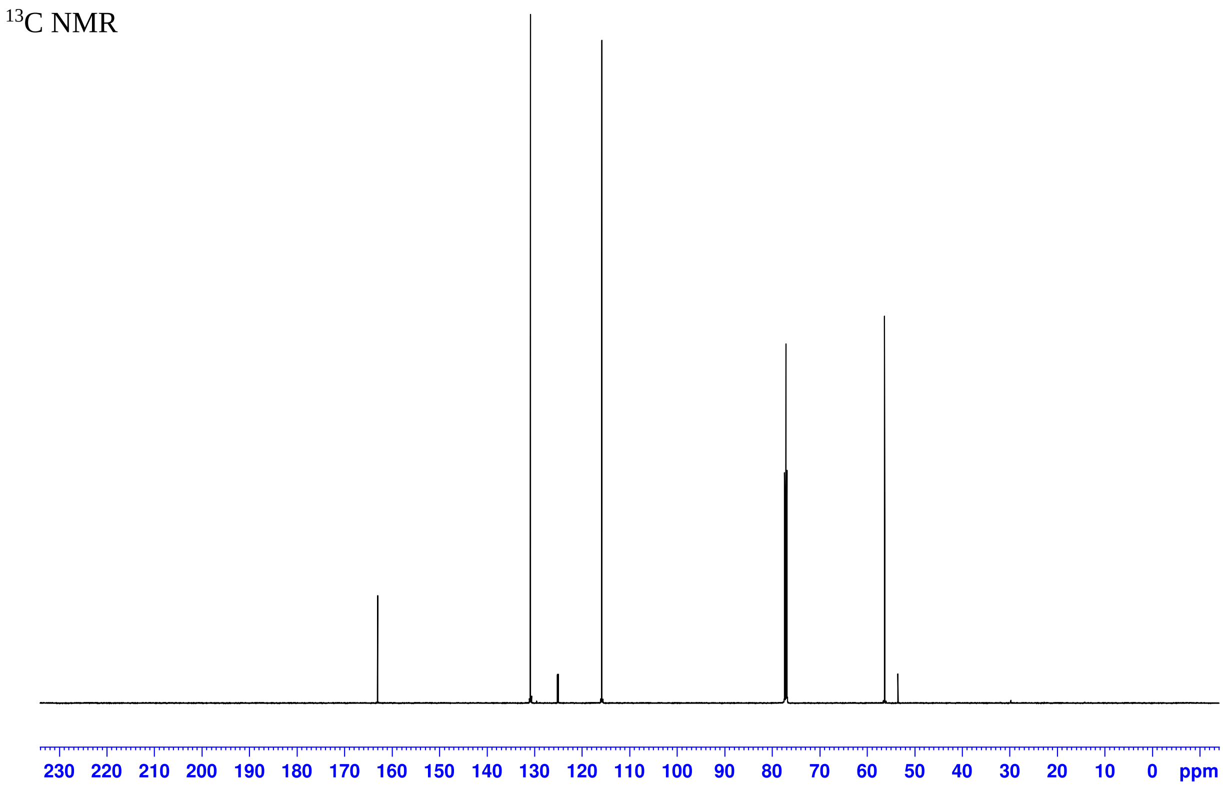

13C NMR
230 220 210 200 190 180 170 160 150 140 130 120 110 100 90 80 70 60 50 40 30 20 10 0 ppm

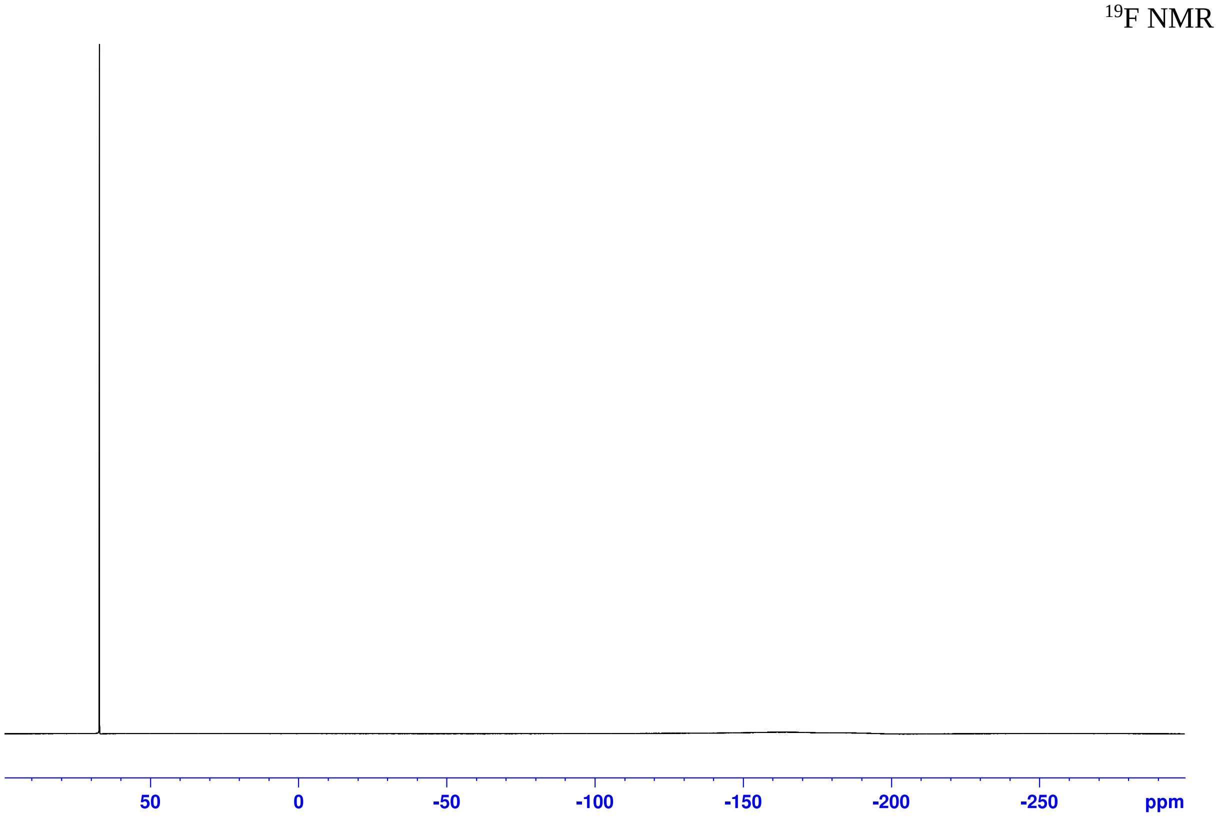

19F NMR
50 0 -50 -100 -150 -200 -250 ppm

8 References

1 N. Zoeller and D. Blankschtein, *Langmuir*, 1998, **14**, 7155–7165.

2 P. Klumphu and B. H. Lipshutz, *J. Org. Chem.*, 2014, **79**, 888–900.

3 M. Borkovec, in *Analysis and Characterisation in surface chemistry*, 2002, vol. 2, pp. 357–370.

4 J. Gapiński, J. Szymański, A. Wilk, J. Kohlbrecher, A. Patkowski and R. Hołyst, *Langmuir*, 2010, **26**, 9304–9314.

5 P. Bäverbäck, C. L. P. Oliveira, V. M. Garamus, I. Varga, P. M. Claesson and J. S. Pedersen, *Langmuir*, 2009, **25**, 7296–7303.

6 M. Ouanezar, F. Guyomarc'h and A. Bouchoux, *Langmuir*, 2012, **28**, 4915–4919.

7 Y. I. González and E. W. Kaler, *Curr. Opin. Colloid Interface Sci.*, 2005, **10**, 256–260.

8 J. Chamieh, F. Davanier, V. Jannin, F. Demarne and H. Cottet, *Int. J. Pharm.*, 2015, **492**, 46–54.

9 A. Sullo and I. T. Norton, in *Encyclopedia of Food and Health*, 2016, pp. 7–15.

10 D. F. Evans and H. Wennerström, *The colloidal domain: Where physics, chemistry, biology, and technology meet*, Wiley VHC, New York, 1999.

11 R. Shah, D. Eldridge, E. Palombo and I. Harding, *J. Phys. Sci.*, 2014, **25**, 59–75.

12 S. Samimi, N. Maghsoudnia, R. B. Eftekhari and F. Dorkoosh, *Lipid-Based Nanoparticles for Drug Delivery Systems*, Elsevier Inc., 2018.

13 A. Sze, D. Erickson, L. Ren and D. Li, *J. Colloid Interface Sci.*, 2003, **261**, 402–410.

14 RDKit, 2023, Open-Source Cheminformatics.

15 Swain Matt, Python interface for the Chemical Identifier Resolver, https://cirpy.readthedocs.io/en/latest/.

16 rdkit.Chem.AllChem module.

17 Pymol.org.

18 J. R. Lu, Z. X. Li, R. K. Thomas, E. J. Staples, I. Tucker and J. Penfold, *J. Phys. Chem.*, 1993, **97**, 8012–8020.

19 R. P. Cooney, C. G. Barraclough and T. W. Healy, *J. Phys. Chem.*, 1983, **87**, 1868–1873.

20 Steven Abbott, *Surfactant Science. Principles and Practice*, 2015.

21 D. F. Veber, S. R. Johnson, H.-Y. Cheng, B. R. Smith, K. W. Ward and K. D. Kopple, *J. Med. Chem.*, 2002, **45**, 2615–2623.

22 S. Duangjit, B. Pamornpathomkul, P. Opanasopit, T. Rojanarata, Y. Obata, K. Takayama and T. Ngawhirunpat, *Int. J. Nanomedicine*, 2014, **9**, 2005–2017.

23 L. Wang, J. Ding, L. Pan, D. Cao, H. Jiang and X. Ding, *Chemom. Intell. Lab. Syst.*, 2021, **217**, 104384.

24 J. J. P. Stewart, *J. Mol. Model.*, 2007, **13**, 1173–1213.

25 M. J. Frisch, G. W. Trucks, H. B. Schlegel, G. E. Scuseria, M. A. Robb, J. R. Cheeseman, G. Scalmani, V. Barone, G. A. Petersson, H. Nakatsuji, X. Li, M. Caricato, A. Marenich, J. Bloino, B. G. Janesko, R. Gomperts, B. Mennucci, H. P. Hratchian, J. V. Ortiz, A. F. Izmaylov, J. L. Sonnenberg, D. Williams-Young, F. Ding, F. Lipparini, F. Egidi, J. Goings, B. Peng, A. Petrone, T. Henderson, D. Ranasinghe, V. G. Zakrzewski, J. Gao, N. Rega, G. Zheng, W. Liang, M. Hada, M. Ehara, K. Toyota, R. Fukuda, J. Hasegawa, M. Ishida, T. Nakajima, Y. Honda, O. Kitao, H. Nakai, T. Vreven, K. Throssell, J. A. Montgomery, J. E. P. Jr., F. Ogliaro, M. Bearpark, J. J. Heyd, E. Brothers, K. N. Kudin, V. N. Staroverov, T. Keith, R. Kobayashi, J. Normand, K. Raghavachari, A. Rendell, J. C. Burant, S. S. Iyengar, J. Tomasi, M. Cossi, J. M. Millam, M. Klene, C. Adamo, R. Cammi, J. W. Ochterski, R. L. Martin, K. Morokuma, O. Farkas, J. B. Foresman and D. J. Fox, 2016.

26 S. Miertuš, E. Scrocco and J. Tomasi, *Chem. Phys.*, 1981, **55**, 117–129.

27 F. De Proft, C. Van Alsenoy, A. Peeters, W. Langenaeker and P. Geerlings, *J. Comput. Chem.*, 2002, **23**, 1198–1209.

28 T. Lu and F. Chen, *J. Comput. Chem.*, 2012, **33**, 580–592.

29 M. E. Tipping and C. M. Bishop, *J. R. Stat. Soc. Ser. B (Statistical Methodol.*, 1999, **61**, 611–622.

30 MATLAB, Probabilistic Principal Component Analysis - MATLAB ppca, https://www.mathworks.com/help/stats/ppca.html, (accessed 12 August 2023).

31 RDocumentation, bpca function: RDocumentation, https://www.rdocumentation.org/packages/pcaMethods/versions/1.64.0/topics/bpca, (accessed 12 August 2023).

32 S. Oba, M. Sato, I. Takemasa, M. Monden, K. Matsubara and S. Ishii, *Bioinformatics*, 2003, **19**, 2088–2096.

33 H. WOLD, ed. P. R. B. T.-M. A. KRISHNAIAH, Academic Press, 1973, pp. 383–407.

34 K. Wright, The NIPALS algorithm, https://cran.r-project.org/web/packages/nipals/vignettes/nipals_algorithm.html, (accessed 12 August 2023).

35 H. Martens and M. Martens, *Multivariate Analysis of Quality: An Introduction*, Wiley, 2001.

36 M. Scholz, F. Kaplan, C. L. Guy, J. Kopka and J. Selbig, *Bioinformatics*, 2005, **21**, 3887–3895.

37 M. A. Kramer, *AIChE J.*, 1991, 37233–243.

38 X. Hao, Z. Xu, H. Lu, X. Dai, T. Yang, X. Lin and F. Ren, *Org. Lett.*, 2015, **17**, 3382–3385.

39 C.-H. Zhang, K. A. Spasov, R. A. Reilly, K. Hollander, E. A. Stone, J. A. Ippolito, M.-E. Liosi, M. G. Deshmukh, J. Tirado-Rives, S. Zhang, Z. Liang, S. J. Miller, F. Isaacs, B. D. Lindenbach, K. S. Anderson and W. L. Jorgensen, *ACS Med. Chem. Lett.*, 2021, **12**, 1325–1332.

40 H. Y. Kim and K. Oh, *Org. Lett.*, 2011, **13**, 1306–1309.

41 Y.-J. Chen and C. Chen, *Tetrahedron: Asymmetry*, 2007, **18**, 1313–1319.

42 G. Pupo, F. Ibba, D. M. H. Ascough, A. C. Vicini, P. Ricci, K. E. Christensen, L. Pfeifer, J. R. Morphy, J. M. Brown, R. S. Paton and V. Gouverneur, *Science (80-.).*, 2018, **360**, 638–642.

43 L. Tang, Y. Yang, L. Wen, X. Yang and Z. Wang, *Green Chem.*, 2016, **18**, 1224–1228.

44 G. Laudadio, A. de A. Bartolomeu, L. M. H. M. Verwijlen, Y. Cao, K. T. de Oliveira and T. Noël, *J. Am. Chem. Soc.*, 2019, **141**, 11832–11836.

45 D. I. Bugaenko, A. A. Volkov, V. V Andreychev and A. V Karchava, *Org. Lett.*, 2023, **25**, 272–276.

46 Y. Liu, D. Yu, Y. Guo, J.-C. Xiao, Q.-Y. Chen and C. Liu, *Org. Lett.*, 2020, **22**, 2281–2286.

47 M. Pérez-Palau and J. Cornella, *European J. Org. Chem.*, 2020, **2020**, 2497–2500.

48 T. S.-B. Lou, Y. Kawamata, T. Ewing, G. A. Correa-Otero, M. R. Collins and P. S. Baran, *Angew. Chemie Int. Ed.*, 2022, **61**, e202208080.

49 X. Song, Y. He, B. Wang, S. Peng, X. Pan, M. Wei, Q. Liu, H.-L. Qin and H. Tang, *Tetrahedron*, 2022, **108**, 132657.

50 J. A. H. Inkster, K. Liu, S. Ait-Mohand, P. Schaffer, B. Guérin, T. J. Ruth and T. Storr, *Chem. – A Eur. J.*, 2012, **18**, 11079–11087.
